# The effects of aging on the BTBR mouse model of autism spectrum disorder

**DOI:** 10.3389/fnagi.2014.00225

**Published:** 2014-09-01

**Authors:** Joan M. Jasien, Caitlin M. Daimon, Rui Wang, Bruce K. Shapiro, Bronwen Martin, Stuart Maudsley

**Affiliations:** ^1^Metabolism Unit, Laboratory of Clinical Investigation, National Institutes of Health, National Institute on AgingBaltimore, MD, USA; ^2^Department of Neurology, Johns Hopkins University School of Medicine, Kennedy Krieger InstituteBaltimore, MD, USA; ^3^Receptor Pharmacology Unit, Laboratory of Neurosciences, National Institute on AgingBaltimore, MD, USA; ^4^VIB-Department of Molecular Genetics, University of AntwerpAntwerp, Belgium

**Keywords:** ASD, autism, BDNF, aging, synaptic marker, neuroprotection, neurodevelopmental disorder, BTBR

## Abstract

Autism spectrum disorder (ASD) is a complex heterogeneous neurodevelopmental disorder characterized by alterations in social functioning, communicative abilities, and engagement in repetitive or restrictive behaviors. The process of aging in individuals with autism and related neurodevelopmental disorders is not well understood, despite the fact that the number of individuals with ASD aged 65 and older is projected to increase by over half a million individuals in the next 20 years. To elucidate the effects of aging in the context of a modified central nervous system, we investigated the effects of age on the BTBR T + tf/j mouse, a well characterized and widely used mouse model that displays an ASD-like phenotype. We found that a reduction in social behavior persists into old age in male BTBR T + tf/j mice. We employed quantitative proteomics to discover potential alterations in signaling systems that could regulate aging in the BTBR mice. Unbiased proteomic analysis of hippocampal and cortical tissue of BTBR mice compared to age-matched wild-type controls revealed a significant decrease in brain derived neurotrophic factor and significant increases in multiple synaptic markers (spinophilin, Synapsin I, PSD 95, NeuN), as well as distinct changes in functional pathways related to these proteins, including “*Neural synaptic plasticity regulation”* and “*Neurotransmitter secretion regulation*.” Taken together, these results contribute to our understanding of the effects of aging on an ASD-like mouse model in regards to both behavior and protein alterations, though additional studies are needed to fully understand the complex interplay underlying aging in mouse models displaying an ASD-like phenotype.

## Introduction

Autism spectrum disorder (ASD) is a heterogeneous neurodevelopmental disorder characterized by varying degrees of altered social functioning and engagement in repetitive, stereotyped behaviors accompanied by display of narrowed interests (American Psychiatric Association, DSM-V 2012). The most recent surveillance data collected in 2008 by the Center for Disease Control's Autism and Developmental Disabilities Monitoring Network indicates that approximately 1 in 88 children has an autism spectrum disorder (CDC, 2010). The United States Census Bureau in 2006 projected a doubling of the US population aged 65 and older by 2030. Assuming the life expectancy of individuals with ASD is similar to that of the general population, based on current prevalence rates in school aged children, this population increase would result in a prevalence of approximately 700,000 individuals with ASD aged 65 in the next 20 years (Piven and Rabins, 2011).

Despite the population statistics indicating a significant number of aged individuals with ASD, studies that investigate the effects of the neurodevelopmental disabilities including ASD, in an aged physiological context are lacking. Due to the paucity and limitations of studies and the expected increase in the aged individuals presenting ASD a multidisciplinary conference was held in March 2010 to identify gaps in knowledge regarding older adults with ASD. The panel concluded that descriptive studies of aging in humans and rodent models were needed (Piven and Rabins, 2011). A limited number of human studies have investigated the persistence of the ASD core deficits into adulthood (Ballaban-Gil et al., 1996; Seltzer et al., 2003; Shattuck et al., 2007), childhood factors that are associated with prognosis (Rumsey et al., 1985), and outcome studies (Rutter et al., 1967; Howlin et al., 2004; Renty and Roeyers, 2006). Major findings from these studies suggest that language development (Kanner et al., 1972) and Intelligence Quotient (IQ) predicts outcome (Lotter, 1974; Rumsey et al., 1985; Szatmari et al., 1989). The ASD diagnosis persists with inconsistent findings about which, if any, core deficits change (Ballaban-Gil et al., 1996; Piven et al., 1996; Seltzer et al., 2003), and that the adults with ASD remain dependent on others (Rutter et al., 1967) even if they have a normal IQ (Howlin et al., 2004). Despite persistent dependence some studies found that individuals with ASD had improved symptoms over time (Szatmari et al., 1995; Piven et al., 1996; Seltzer et al., 2003). However, a few limitations hinder these early studies of aging in ASD, including study individuals being only 20–30 years old, small sample sizes, and an inability to completely generalize the study findings given the heterogeneity of the population examined (Rumsey et al., 1985; Gillberg and Wing, 1999; Fombonne et al., 2001; Howlin and Moss, 2012).

Given the heterogeneous nature of ASD, mouse models have proven to be particularly useful and reliable in elucidating the mechanism of these disorders (Crawley, 2007; Moy and Nadler, 2008). There are several approaches to ASD mouse models: targeted gene mutations, defects in neurotransmitters or brain regions, inbred strains, and models based on the comorbidity of other human diseases with autism (Crawley, 2007). One strain in particular, BTBR T + tf/j, has been shown to be an especially relevant animal model of ASD (Moy et al., 2007). Extensive behavioral characterization of the BTBR (black and tan, brachyuric) mouse model has revealed low sociability compared to wildtype strains (Bolivar et al., 2007; Moy et al., 2007), resistance to change (Moy et al., 2007; Moy and Nadler, 2008), increased display of self-grooming behavior (Pobbe et al., 2010), display of repetitive behaviors (Pearson et al., 2011), and reduced display of territorial scent marking (Wohr et al., 2011). Furthermore, unusual vocalizations have also been extensively characterized in BTBR mice (Scattoni et al., 2008, 2011), in addition to instances of social avoidance and gaze aversion (Defensor et al., 2011). While the autistic-like behavioral phenotype of the young male BTBR mouse has been studied extensively, the effects of age on this ASD-like mouse model have not been elucidated. As aged human autistic tissue samples are limited and the BTBR mouse model is well characterized and widely accepted, we selected this mouse model to study the nature of the ASD phenotype in an aged context. To enhance our understanding of the aged BTBR mouse and to gain further insight into the effects of aging of ASD, we followed our extensive behavioral characterization of the aged BTBR mouse with quantitative proteomic analyses on cortical and hippocampal tissues as these two brain regions have been strongly associated with ASD (Mundy, 2003; Nadler et al., 2006). We hypothesized that both the behavioral phenotype and proteomic profile would be different between the aged ASD-like mice [or mice with an abnormal central nervous system (CNS)] and the aged wildtype mice (or mice with a presumed normal CNS). Our results contribute to our understanding of the effects of aging on an ASD-like mouse model, and it is our hope that further research will enhance our understanding of aging in an abnormal CNS.

## Materials and methods

### Experimental animals

All experimental animal procedures were approved by the Animal Care and Use Committee of the National Institute on Aging, National Institutes of Health. Male BTBR T + tf/J mice (*n* = 8, 15 months of age) and male control wildtype (WT) C57BL6J (*n* = 5, 15 months of age) were housed in the NIA animal facility on a 12 h light and dark cycle from 6 am to 6 pm and received *ad libitum* access to food and water throughout the duration of the study. All behavioral testing described in detail below was performed between the hours of 9 AM and 5 PM. Prior to behavioral testing, animals were moved from the vivarium to an experimental room and given a minimum of 30 min to habituate to the new environment. Between each experimental animal, all apparatuses were wiped down with 70% ethanol followed by D. I. water to prevent any scent retention that could possibly bias the performances of later mice.

### Social preference assessment

Social behavior was assessed using a three-chambered sociability apparatus with a protocol modeled after previously established methods (Crawley, 2007). In brief, animals were first habituated for 10 min to the three-chambered sociability apparatus (60 × 40 × 22 cm, Stoelting, Wood Dale, IL) free of any stimuli and returned to their home cage for a minimum of 30 min. Prior to the return of the experimental mouse to the chamber for testing, another mouse of the same strain, age, and gender and an inanimate object both housed in identical wire mesh cylinders (15 cm tall, 7 cm in diameter) were placed in opposing compartments of the apparatus. Experimental animals were then returned to the center chamber of the apparatus and were free to choose to interact with either the mouse or object enclosed in the respective wire mesh containers. Interactions were recorded for 10 min and analyzed with ANY-Maze Video Tracking Software (Stoelting). A discrimination index (DI) was calculated that takes into account the relative exploration time spent with either the contained mouse or object: (animal time/total time—object time/total time) × 100. As socially-normal mice show a robust preference for another mouse over an inanimate object, a high DI is reflective of socially-normal behavior while a low DI is indicative of deficits in social functioning.

### Novel object preference test

The novel object preference test was performed as described previously (Dere et al., 2007; Park et al., 2010). In brief, mice were given 10 min to explore a 25 cm^2^ opaque walled box with two identical objects placed equidistant from each other in the center of the box. After 10 min of habituation to the two identical objects was completed, animals were returned to their home cages for a minimum of 30 min. After the habituation task, trained experimenters replaced one of the identical objects with a novel object that had never been seen by the experimental mouse. Experimental animals were then returned to the opaque box and given 10 min in which they were free to interact with either the familiar or novel object. Both the habituation session and testing session were videotaped and the animal's behavior automatically tracked by ANY-Maze Video Tracking Software (Stoelting). Discrimination indices were again calculated: (novel time/total time-same time/total time) × 100. A high discrimination index (DI) is indicative of an enhanced ability to preferentially recognize the novel object, suggesting intact memory functioning.

### Open field test

A generalized view of the behavioral characteristics of both experimental and control mice was obtained via an open field test performed as described previously (Holmes et al., 2002). In brief, each mouse was placed in a square chamber approximately 0.14 m^2^ in size (Med Associates Inc, St. Albans, VT) and given 10 min to freely explore. Activity Monitor software (Med Associates Inc) automatically recorded multiple parameters over the duration of the 10 min test. Individual data points were then compiled and subsequently analyzed to produce the following measurements: distance traveled, time spent in the peripheral areas of the chamber, time spent in the center area of the chamber, number of ambulatory episodes, and number of jumping episodes. Relative time spent in the periphery vs. the center was calculated for each mouse by dividing the time spent in either the periphery or center by the total exploration time (600 s for all animals).

### Light/dark exploration test

The light-dark exploration test was performed as previously described (Chadwick et al., 2011) as a means to assess general anxiety behavior. The same square chamber used in the Open field test was again used to complete the Light/Dark exploration test, with the additional insertion of a dark chamber taking up 50% of the area of the entire apparatus (Dark box insert for mouse open field activity, Med Associates Inc). The dark chamber contains a small arched entrance to allow mice to access the dark environment; this entryway was placed toward the center of the apparatus so mice could easily access it. Time spent in either the light or dark compartment was automatically recorded by Activity Monitor software (Med Associates Inc). Relative time spent in the light chamber vs. the dark chamber was calculated for each mouse by dividing the time spent in either the light or the dark by the total exploration time (600 s for all animals).

### Elevated plus maze

General anxiety behavior was also evaluated using an elevated plus maze test as described previously (Chadwick et al., 2011). In brief, animals were given 5 min to explore a plus shaped maze raised approximately 36 cm off of the ground. Two of the arms of the maze were enclosed on all sides; two arms remained open for the duration of the test. ANY-Maze Video Tracking Software (Stoelting) was used to record time spent in either the closed or open arms of the apparatus and the relative time spent in the closed or open arms was calculated by dividing the time spent in either arm by the total exploration time (300 s for all animals).

### Rota-Rod test

Motor coordination was evaluated using an accelerating Rota-Rod treadmill (Med. Associates, Inc) as described previously (Bohlen et al., 2009). Briefly, mice were given two training sessions on the spinning Rota-Rod apparatus on the day prior to the testing day (the first trial was administered in the morning and the second trial was administered in the afternoon). Each habituation training trial lasted 2 min [4 revolutions per minute (rpm)]. On the test day, the mice were placed on the Rota-Rod, which gradually accelerated from 4 to 40 rpm over a 5 min time interval. The test was performed twice per day and the latency to fall was measured and averaged.

### Animal euthanization and tissue collection

Following completion of behavioral analyses, animals were euthanized via isoflurane overdose (Butler Animal Health Supply, Dublin, OH) as described previously and in accord with approved animal procedures (Chadwick et al., 2011). Bodyweight data was collected immediately prior to euthanization for each animal. Hippocampal and cortical brain tissues were snap frozen on dry ice and stored at −80°C until used for further analyses.

### Protein digestion, iTRAQ labeling and western blotting

Protein digestion and iTRAQ labeling were processed according to the iTRAQ protocol (iTRAQ Reagents—8plex: amine-modifying Labeling Reagents for Multiplexed Relative and Absolute Protein Quantitation, ABSciex). All tissue samples (Hippocampus and Cortex; WT and BTBR) were prepared in parallel throughout the labeling procedures. Briefly, 100 μg of protein in each condition was acetone precipitated and resuspended in 20 μL of iTRAQ dissolution buffer (0.5 M triethylammonium bicarbonate (TEAB), ABSciex) containing 0.1% ProteaseMAX detergent (Promega) to denature the proteins. The sample was then reduced by adding iTRAQ Reducing Reagent [Tris(2-carboxyethyl) phosphine (TCEP), ABSciex] to a final concentration of 5 mM and incubated at 60°C for 1 h. Subsequently, the sample was alkylated with iTRAQ Cysteine-Blocking Reagent (10 mM methyl methanethiosulfonate (MMTS), ABSciex) for 10 min at room temperature. The protein sample was then digested with 5 μg sequencing-grade trypsin (Promega) per 100 μg protein at 37°C overnight. Labeling of the samples with iTRAQ 8-plex labels was performed at room temperature for 2 h. After labeling, the samples to be compared were mixed and underwent an off-line strong cation exchange (SCX) fractionation (ICAT Cation Exchange Buffer Pack, ABSciex) to 16 fractions to reduce sample complexity. After reversed-phase desalting (C18 tips, Pierce), the samples were re-constituted in water with 0.1% formic acid, then stored at −20°C until LC/MS/MS analysis. Western blotting procedures as described previously (Maudsley et al., 2007; Martin et al., 2009a), were performed using the same protein lysates employed for iTRAQ labeling. Antibodies used were as follows: rabbit anti-BDNF [(brain-derived neurotrophic factor) Santa Cruz Biotechnology, Santa Cruz CA], rabbit antiphosphorylated TrkB and TrkB, rabbit anti-phosphorylated Akt and Akt, rabbit antiphosphorylated synapsin 1 and synapsin 1, rabbit anti-spinophilin, rabbit anti-PSD95 and rabbit anti-synaptophysin (Cell signaling technology, Inc., Danvers, MA), mouse anti-NeuN (EMD Millipore Corporation, Billerica, MA) and mouse anti-beta actin (Sigma-Aldrich, St. Louis, MO). Results were quantified and statistical analysis was performed using an unpaired student *t*-test. *p* < 0.05 was considered statistically significant.

### LC/MS/MS analysis

Samples were analyzed using an Eksigent NanoLC Ultra 2D (Dublin, CA) and Thermo Fisher Scientific LTQ Orbitrap XL (San Jose, CA). In brief, peptides were first loaded onto a trap cartridge (Agilent), then eluted onto a reversed phase PicoFrit column (New Objective, Woburn, MA) using a linear 120 min gradient of acetonitrile (2–62%) containing 0.1% formic acid at 250 nL/min flowrate. The eluted peptides were sprayed into the LTQ Orbitrap XL. The data-dependent acquisition mode was enabled, and each FTMS MS1 scan (60,000 resolution) was followed by 6 MS2 scans (alternating CID at unit resolution and HCD at 7500 resolution on 3 precursor ions). The spray voltage and ion transfer tube temperature were set at 1.8 kV and 180°C, respectively.

### Database search and iTRAQ quantification

Proteome Discoverer 1.2 (Thermo Fisher Scientific) was used for protein identification and iTRAQ quantification using Sequest algorithms. The following criteria were followed: SwissProt mouse database; enzyme: trypsin; miscleavages: 2; static modifications: methylthio (+45.988 Da on C), iTRAQ8plex (+304.205 Da on N-terminus and K); dynamic modifications: oxidation (+15.995 Da on M), deamidation (+0.984 Da on N and Q); peptide tolerance as 25 ppm; MS2 tolerance as 0.8 Da. Peptides reported via all search engines were accepted only if they met the false discovery rate of 5%. For iTRAQ quantification, the reporter ion intensities in MS2 spectra (*m*/z 113–121, integration width tolerance 50 mmu) were used to calculate the expression ratios among the different conditions (Hippocampus and Cortex; WT and BTBR).

### Bioinformatics analysis

Functional annotational clustering of our proteomic data, i.e., gene ontology (GO), KEGG (Kyoto Encyclopedia of Genes and Genomes) pathway analysis was performed using WebGestalt (http://bioinfo.vanderbilt.edu/webgestalt/, 3/2014) as previously described (Chadwick et al., 2010a, 2011). In addition Ingenuity Pathway Analysis (http://Ingenuity.com, 3/2014) was employed for Canonical Signaling Pathway enrichment investigations. In brief, GO analysis allows for broad clustering of genes into functional groups, while KEGG pathways analysis allows for more specific clustering into signaling pathways (Maudsley et al., 2011). Inclusion criteria were set as follows: pathway groups needed to meet a minimum population of two genes from the input experimental set, and also needed to possess a probability significance of enrichment compared to a control background dataset of less than *p* < 0.05 (hypergeometric test of significance). In addition, Venn diagrams were also constructed to identify common and uniquely altered genes between hippocampus and cortex, using VennPlex (Cai et al., 2013), as described previously (Martin et al., 2009b).

### *Textrous!* latent semantic analysis-based investigation

*Textrous!* latent semantic analysis was performed as described previously (Chen et al., 2013). In brief, corresponding gene symbols were uploaded into *Textrous!* (http://textrous.irp.nia.nih.gov/, 3/2014) and then the relevant output indices, i.e., Cosine Similarity, Z-score and *p*-value were extracted. In addition to the direct list-based semantic output both the Collective processing mode (generating hierarchical word-clouds), as well the Individual processing mode (generating symbol-word heatmaps) were applied to provide more nuanced informatic appreciation of the specific input datasets.

### Statistical analysis

All statistical analyses were conducted using a Student's *t*-test (two-tailed with equal variances: GraphPad Prism, version 5.02). *p* < 0.05 was considered statistically significant throughout the study. Error bars represent 95% confidence interval. All data represent means ± s.e.m. (standard error of mean). ^*^p < 0.05; ^**^p < 0.01; ^***^p < 0.001.

## Results

### ASD-like behavioral phenotype persists in aged BTBR mice

**Figure 1:**
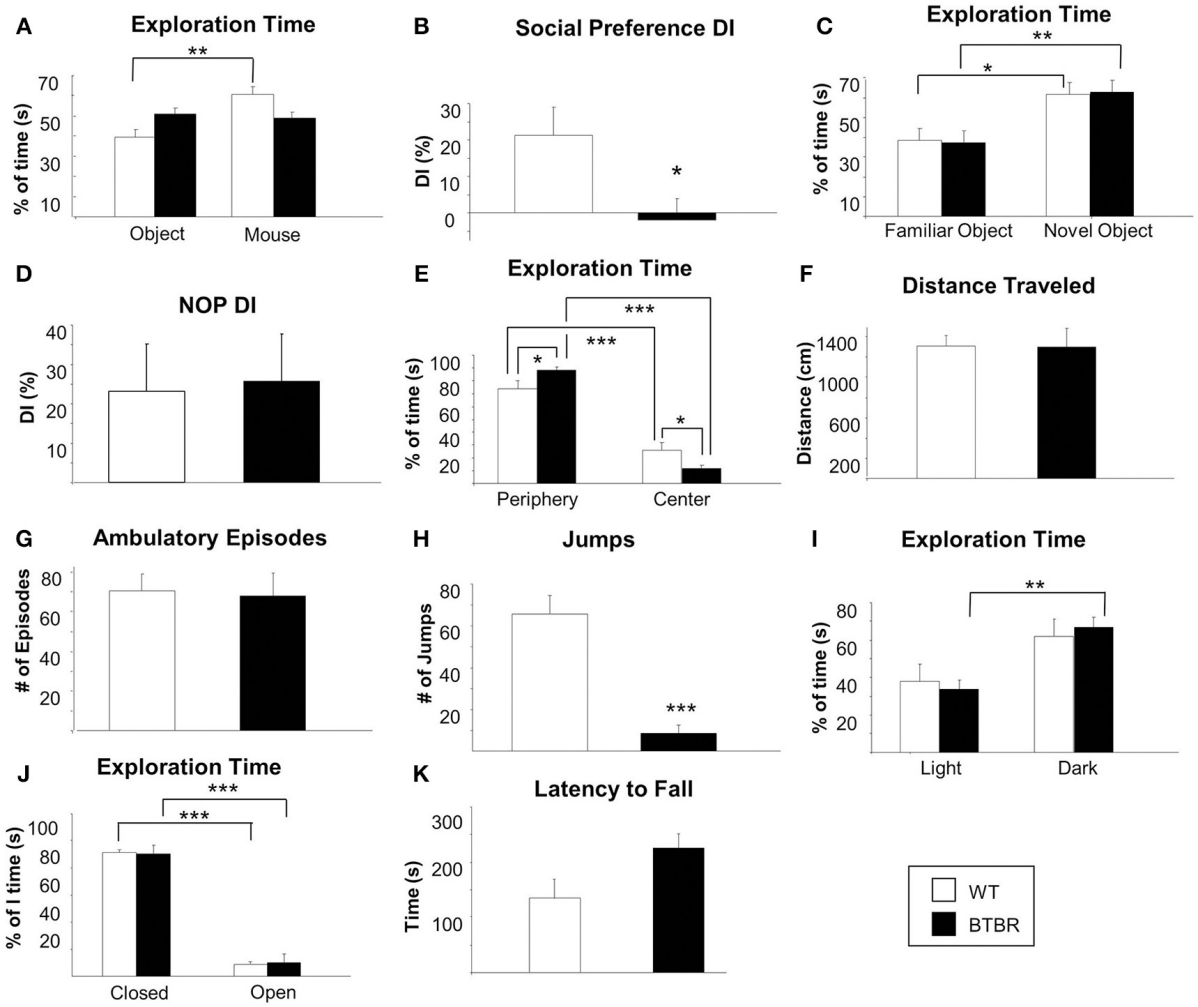
**Social function, cognitive ability, general behavior, anxiety and motor coordination in an aged ASD mouse model**. Social preference was assessed using a three-chambered sociability apparatus in aged male BTBR T + tf/j mice and age-sex-matched control mice **(A, B)**. Percentage of time spent exploring both the contained inanimate object and the contained mouse was measured for male BTBR (*n* = 8) and control mice (*n* = 5) **(A)**. A discrimination index (DI) was calculated **(B)** which represents the degree to which experimental or control animals displayed a preference for exploring the contained mouse over the contained inanimate object. Cognitive functioning was assessed via the novel object preference task (NOP) **(C, D)**. Percentage of time spent exploring either a familiar or a novel object was again measured for male BTBR (*n* = 8) and control mice (*n* = 5) **(C)** and a DI was calculated **(D)** with a higher DI indicating an increased ability to discern a novel object from a familiar object. Exploration of an open field apparatus was assessed in male BTBR and control mice was measured with regard to the following parameters: time spent exploring the peripheral or central areas of the apparatus relative to the total amount of time spent exploring **(E)**, total distance traveled **(F)**, number of ambulatory episodes **(G)**, and number of jumps **(H)**. Exploration of the open field apparatus was repeated with a Light/Dark insert add-on and relative time spent in either the light half or the dark half of the chamber was recorded **(I)**. General anxiety was assessed using the elevated plus maze task. For both BTBR T + tf/j mice and age-and sex-matched controls, relative spent in the closed vs. the open arm was recorded as a percentage of total time **(J)**. Finally, motor coordination was assessed using a rotarod apparatus latency to fall was recorded in both BTBR T + tf/j and control mice (**K**, *n* = 5). Data are expressed as means ± s.e.m. Asterisks represent *p*-values as shown: ^*^p < 0.05, ^**^p < 0.01, ^***^p < 0.001. Statistical significance was measured using a Student's *t*-test.

**Figure 2:**
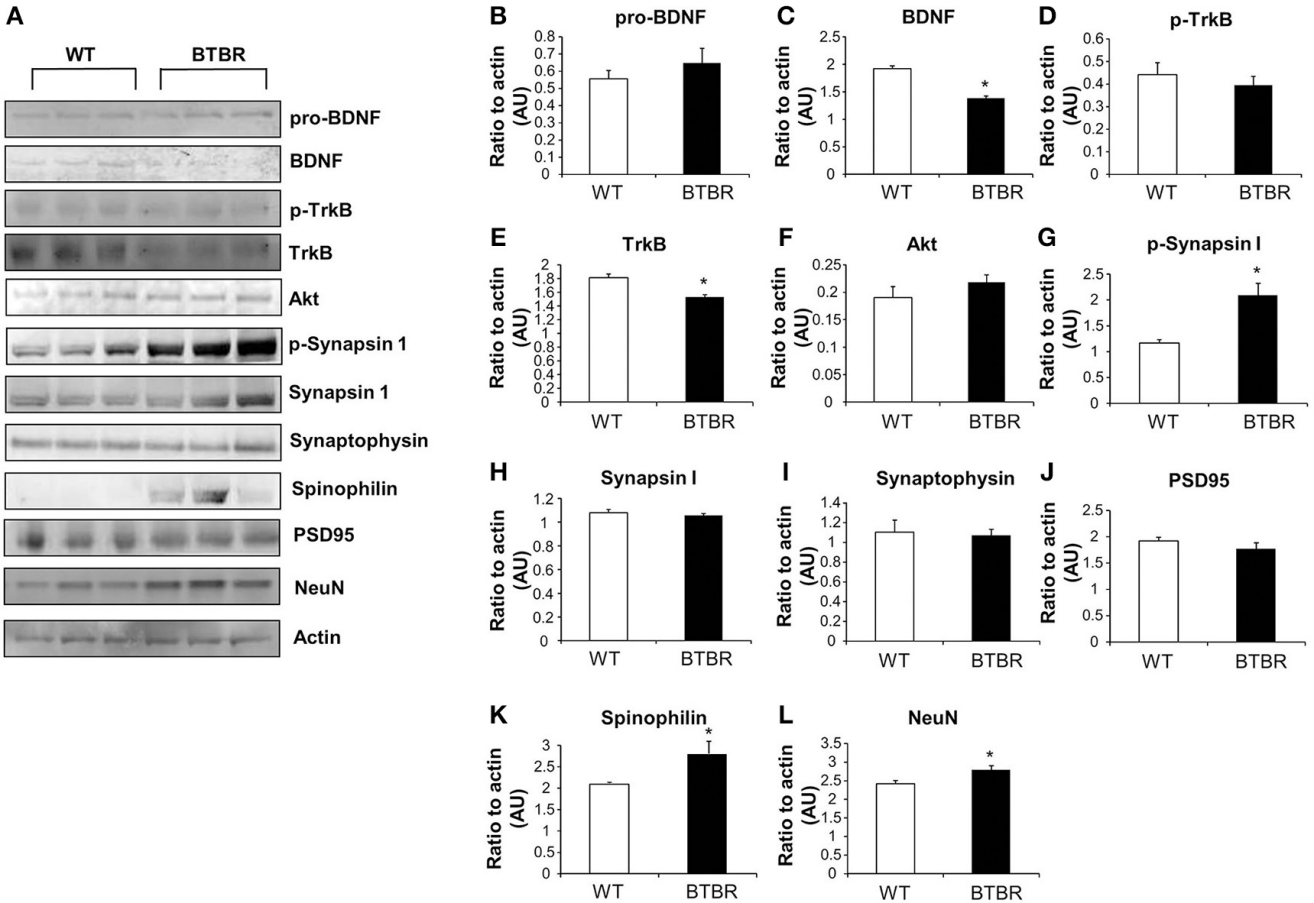
**Protein expression in wild type and BTBR mouse cortex**. Western blotting was performed using mouse cortical protein lysates **(A)**. Protein levels were measured in BTBR and control mice with respect to the following proteins in the cortex: pro-BDNF **(B)**, mature BDNF **(C)**, phospho-TrkB **(D)**, total TrkB **(E)**, Akt **(F)** phospho-synapsin 1 **(G)**, synapsin 1 **(H)** synaptophysin **(I)**, PSD95 **(J)**, spinophilin **(K)**, Neuronal cell marker NeuN **(L)**. Data are expressed as means ± s.e.m. Asterisks represent *p*-values as shown: ^*^p < 0.05. Statistical significance was measured using a Student's *t*-test, *n* = 3 for each group.

Commensurate with an extant ASD phenotype aged BTBR mice demonstrated no sociability preference between an object or another mouse, while WT controls spent significantly more time with another mouse compared to an object (Figure 1A). Relative percentage of time spent with either the mouse or the object was used to calculate a social discrimination index [DI: (novel time/total time-same time/total time) × 100] (Figure 2B). WT and BTBR mice performed similarly in the novel object preference test of learning and memory, with both groups spending significantly more time with a novel object compared to a familiar object (Figures 1C,D). Both WT and BTBR mice significantly preferred to explore the peripheral areas of the chamber over the center of the chamber, though the BTBR mice did show a significantly higher preference for the periphery over the center (Figure 1E). No significant differences in total distance traveled (Figure 1F) or number of ambulatory episodes (Figure 1G) were found. BTBR mice however demonstrated significantly less jumping activity compared to WT (Figure 1H). In light-dark exploration tests BTBR mice spent significantly more time in the dark chamber compared to the lighted chamber, while WT control mice show no statistical difference in preference for either chamber (Figure 1I). In the elevated plus maze test of anxiety, both WT and BTBR mice significantly preferred closed arms to open arms (Figure 1J). Motor coordination, assessed using a Rotarod test was similar in both groups (Figure 1K).

### Altered protein expression in CNS of aged BTBR mice

Cortical and hippocampal western blotting of proteins, selected for their ability to indicate alterations of neurosynaptic activity, was employed to detect potential expression differences between aged WT and BTBR mice. Our panel of neurosynaptic factors analyzed in cortical tissue is indicated in Figure 2A. No significant difference between WT and BTBR pro-BDNF (brain-derived neurotrophic factor) levels was detected (Figure 2B), whereas a significant decreased in BTBR mature BDNF was observed compared to WT (Figure 2C). Levels of phosphorylated TrkB receptor were similar in WT and BTBR cortex (Figure 2D) while the total TrkB expression was significantly lower in BTBR mice compared to WT (Figure 2E). No significant differences in Akt1 expression was noted in the BTBR model compared to WT (Figure 2F). Levels of phosphorylated synapsin 1 (Figure 2G), but not total synapsin 1 (Figure 2H) were significantly elevated in BTBR mice compared to WT. Expression of both synaptophysin (Figure 2I) and PSD95 (Figure 2J) was not affected in BTBR mice, while both spinophilin (Figure 2K) and NeuN (Figure 2L) levels were significantly elevated in BTBR mice compared to WT.

**Figure 3:**
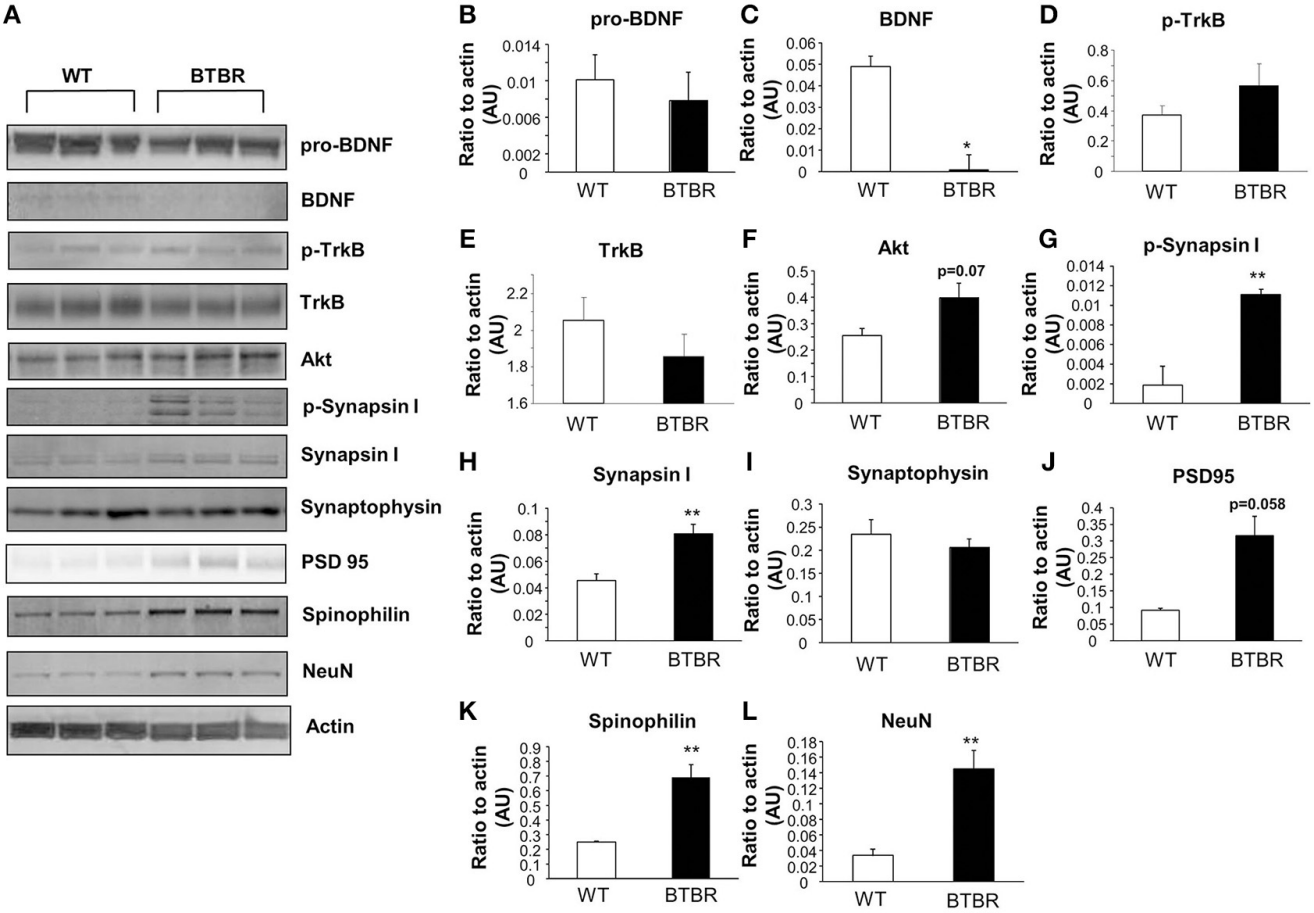
**Protein expression in wild type and BTBR mouse hippocampus**. Western blotting was performed using mouse hippocampal protein lysates **(A)**. Protein levels were measured in BTBR and control mice with respect to the following proteins in the hippocampus: pro-BDNF **(B)**, mature BDNF **(C)**, phospho-TrkB **(D)**, total TrkB **(E)**, Akt **(F)** phospho-synapsin 1 **(G)**, synapsin 1 **(H)** synaptophysin **(I)**, PSD95 **(J)**, spinophilin **(K)**, Neuronal cell marker NeuN **(L)**. Data are expressed as means ± s.e.m. Asterisks represent *p*-values as shown: ^*^p < 0.05, ^**^p < 0.01. Statistical significance was measured using a Student's *t*-test, *n* = 3 for each group.

In the hippocampus we again found no significant differences in the levels of pro-BDNF between BTBR and WT (Figure 3B) that was coincident with a significant reduction in extant BDNF levels (Figure 3C). Phosphorylated TrkB expression was unchanged in BTBR mice compared to WT (Figure 3D), while there was a trend, similar to that in the cortex, to reductions of total TrkB receptor expression in the BTBR mice (Figure 3E). As with the cortex no significant alterations in Akt1 were observed (Figure 3F). Significant potentiation of both phosphorylated (Figure 3G) and non-phosphorylated synapsin 1 (Figure 3H) expression levels was seen in the BTBR hippocampus. In accordance with the cortical data we found no significant BTBR-induced changes in the hippocampal expression of synaptophysin (Figure 3I) or PSD95 (Figure 3J). Levels of both spinophilin (Figure 3K) and NeuN (Figure 3L) were again significantly potentiated in the BTBR mice, mirroring our previous cortical data.

### Systemic analysis of BTBR central nervous protein expression

**Figure 4:**
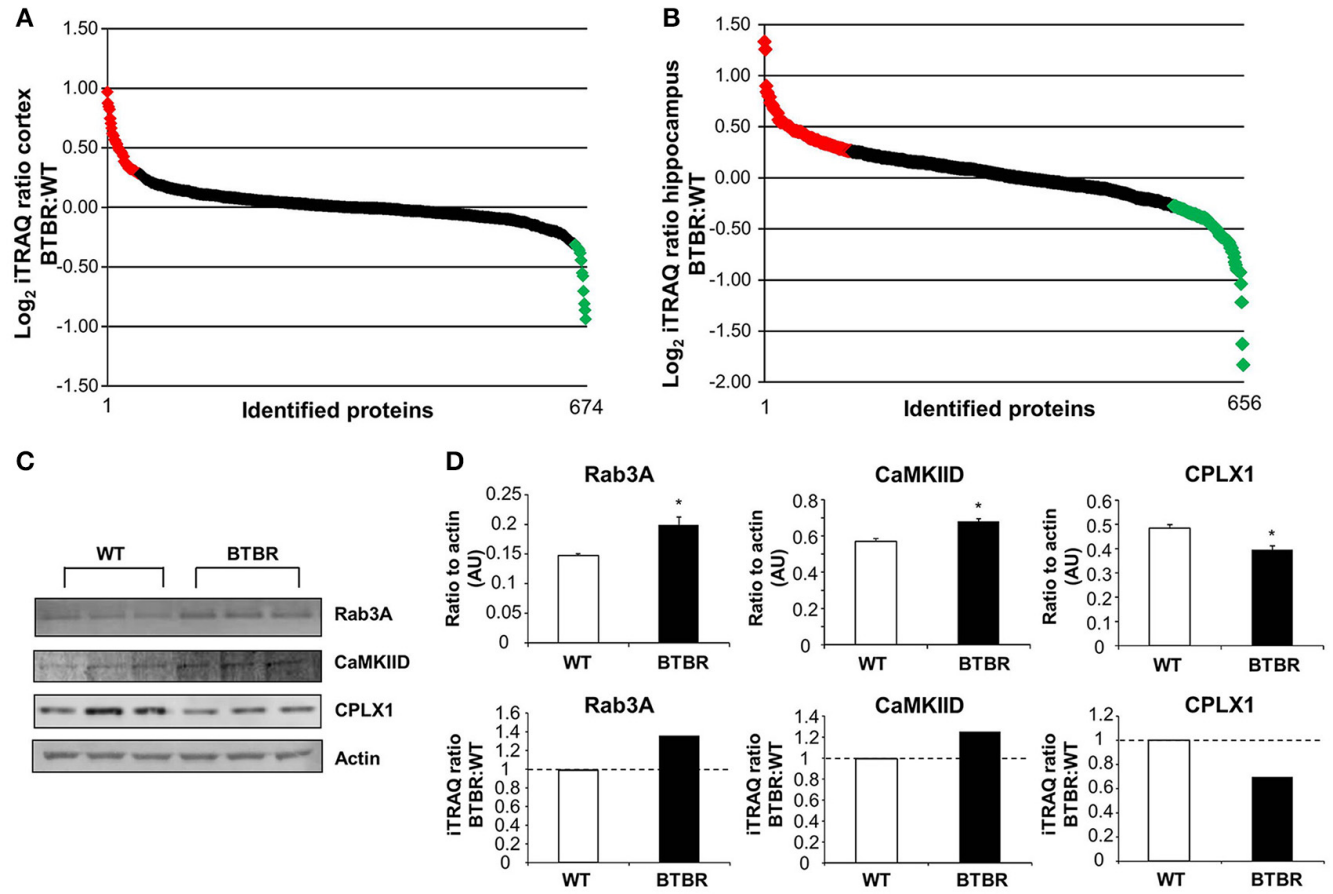
**Quantitative proteomic and bioinformatics analysis of BTBR CNS tissues**. Log_2_transformed iTRAQ ratio data (BTBR:WT) snake plot of the proteins identified in the cortex **(A)** and hippocampus **(B)** are depicted. Upregulated proteins are highlighted in red, while downregulated proteins are highlighted in green. Three proteins identified by iTRAQ were validated via western blot and results correlated to the iTRAQ results **(C, D)**. Data are expressed as means ± s.e.m. Asterisks represent *p*-values as shown: ^*^p < 0.05. Statistical significance was measured using a Student's *t*-test.

To gain an unbiased appreciation of the multiple tissue protein expression pattern changes generated in the BTBR mice compare to WT we applied quantitative isobaric mass-tag labeling (iTRAQ) to cortical and hippocampal tissue extracts. In the cortex we identified and generated relative expression profiles for 674 proteins (Figure 4A: Table S1), while in the hippocampus 656 proteins were identified and quantified in BTBR mice (Figure 4B: Table S2). Log_2_-transformed iTRAQ expression ratios were employed to generate snake plot graphs of individual proteins. To initially validate some of the iTRAQ data we chose three random proteins, Rab3A, CaMKIID and Cplx1 and found that at the western level of detection our results were comparable to our iTRAQ expression ratio data (Figures 4C,D).

### Gene ontology bioinformatic analysis of BTBR-specific altered proteins

Using a standard arbitrary cut-off of >1.2 (elevated in BTBR vs. WT) or <0.8 (reduced in BTBR vs. WT) for the iTRAQ ratios for differentially expressed proteins between BTBR and WT mice we investigated the functional clustering of the BTBR-specific protein changes into Gene Ontology term groups. Analysis of the differentially–regulated [elevated (<1.2) and reduced (<0.8)] proteins in the BTBR cortex we found a strong GO-biological process clustering of endoplasmic reticular, vesicular transport and neurotransmission related functions (Table 1). No GO-molecular function annotation was achieved with the cortical data however. With respect to GO-cellular compartment analysis we found the differential BTBR proteins were strongly associated with endocytic activity, neuronal outgrowth and architecture as well as mitochondrial metabolic activity (Table 1). GO-biological process analysis of the differential BTBR hippocampal protein set indicated a strong bias toward neuronal synaptic secretion, neuronal plasticity, metabolic activity and inter-cellular contact (Table 2). GO-cellular compartment interrogation of the hippocampal dataset revealed a strong bias for protein interactions in the synaptic cleft, clathrin-coated vesicles and mitochondrial-associated metabolic areas (Table 2). GO-molecular function annotation was evident with the hippocampal data and revealed the presence of GO groups associated with malate metabolism, redox activity, synaptic vesicle docking and structural modeling (Table 2).

**Table 1:** **GO term analysis for cortical protein alterations observed in aged BTBR compared to WT control**.

**GO group**	**GO term**	**GO ID**	***C***	***O***	***E***	***R***	***P***	***H***
Biological process	Endoplasmic reticulum tubular network organization	71786	4	2	0.02	126.12	0.0047	293.595
Biological process	Early endosome to late endosome transport	45022	21	3	0.08	36.03	0.0041	86.0114
Biological process	Vesicle-mediated transport	16192	909	14	3.6	3.88	0.0018	10.64954
Biological process	Secretion by cell	32940	693	11	2.75	4	0.0041	9.548865
Biological process	Secretion	46903	795	12	3.15	3.81	0.0037	9.265151
Biological process	Transmission of nerve impulse	19226	735	11	2.91	3.78	0.0047	8.79947
Biological process	Neurological system process	50877	1237	16	4.9	3.26	0.002	8.798642
Biological process	System process	3008	1695	19	6.72	2.83	0.002	7.638085
Biological process	Multicellular organismal signaling	35637	751	11	2.98	3.69	0.0087	7.603174
Biological process	Transport	6810	3338	30	13.23	2.27	0.0006	7.313597
Biological process	Establishment of localization	51234	3392	30	13.45	2.23	0.0006	7.184723
Biological process	Establishment of localization in cell	51649	1756	19	6.96	2.73	0.0027	7.012377
Biological process	Cellular localization	51641	1977	20	7.84	2.55	0.0035	6.262626
Biological process	Localization	51179	4167	31	16.52	1.88	0.0037	4.571781
Cellular component	Chaperonin-containing T-complex	5832	7	2	0.03	78.59	0.0039	189.3182
Cellular component	Pericentriolar material	242	12	2	0.04	45.84	0.0076	97.1435
Cellular component	Clathrin-sculpted vesicle	60198	12	2	0.04	45.84	0.0076	97.1435
Cellular component	Clathrin-coated vesicle membrane	30665	110	5	0.4	12.5	0.0011	36.98259
Cellular component	Filopodium	30175	55	3	0.2	15	0.0076	31.7878
Cellular component	Coated pit	5905	57	3	0.21	14.48	0.0082	30.20798
Cellular component	Coated vesicle membrane	30662	150	5	0.55	9.17	0.0032	22.87777
Cellular component	Lamellipodium	30027	121	4	0.44	9.09	0.0076	19.2634
Cellular component	Ruffle	1726	123	4	0.45	8.94	0.0076	18.94553
Cellular component	Neuron projection	43005	651	12	2.37	5.07	0.0002	18.75378
Cellular component	Cell leading edge	31252	257	6	0.93	6.42	0.0039	15.46537
Cellular component	Clathrin-coated vesicle	30136	207	5	0.75	6.64	0.0076	14.0714
Cellular component	Cell projection	42995	1230	16	4.47	3.58	0.0002	13.24231
Cellular component	Mitochondrion	5739	1525	18	5.54	3.25	0.0002	12.02165
Cellular component	Cytoplasmic part	44444	6772	44	24.62	1.79	7.70E-05	7.363182
Cellular component	Microtubule cytoskeleton	15630	863	10	3.14	3.19	0.0076	6.760205
Cellular component	Cytoskeleton	5856	1790	15	6.51	2.3	0.0093	4.672489
Cellular component	Cytoplasm	5737	9130	49	33.19	1.48	0.0007	4.669255
Cellular component	Cytosol	5829	2372	18	8.62	2.09	0.0093	4.245871
Cellular component	Intracellular	5622	12564	58	45.68	1.27	0.0012	3.70944
Cellular component	Intracellular part	44424	12237	55	44.49	1.24	0.0082	2.586871

GO term annotation of the cortical proteins possessing BTBR:WT iTRAQ expression ratios <1.2 or <0.8 were created using WebGestalt. Each GO term group significantly populated (p < 0.05) by at least two independent proteins are depicted. For each GO Term group the specific GO term ID, number of total proteins in the curated GO term cluster (C), the number of observed experimental proteins in the GO term group (O), the expected number of proteins in the group based on whole-genome background frequency (E), the GO term group enrichment factor (R), the GO term enrichment probability (P) and the hybrid score (R ^*^ −log_10_ P) are indicated (H).

**Table 2:** **GO term analysis for hippocampal protein alterations observed in aged BTBR compared to WT controls**.

**GO Class**	**GO Term**	**GO ID**	***C***	***E***	***O***	***R***	***P***	***H***
Biological process	Neurotransmitter secretion	7269	99	15	1.25	11.98	4.40E–10	112.0914
Biological process	Synaptic vesicle endocytosis	48488	22	6	0.28	21.57	8.46E–06	109.4166
Biological process	Regulation of neuronal synaptic plasticity	48168	47	9	0.59	15.14	4.14E–07	96.63861
Biological process	Synaptic vesicle transport	48489	68	11	0.86	12.79	5.71E–08	92.64262
Biological process	Regulation of synaptic plasticity	48167	96	13	1.21	10.71	1.68E–08	83.26694
Biological process	Neurotransmitter transport	6836	133	15	1.68	8.92	1.21E–08	70.62155
Biological process	Synaptic transmission	7268	651	41	8.23	4.98	8.14E–15	70.16509
Biological process	Regulation of neurotransmitter levels	1505	137	15	1.73	8.66	1.54E–08	67.65607
Biological process	Regulation of synaptic transmission	50804	186	18	2.35	7.65	3.76E–09	64.44981
Biological process	Regulation of neurological system process	31644	217	19	2.74	6.92	5.27E–09	57.28507
Biological process	Regulation of transmission of nerve impulse	51969	204	18	2.58	6.98	1.08E–08	55.6067
Biological process	Transmission of nerve impulse	19226	735	41	9.29	4.41	2.94E–13	55.26459
Biological process	Multicellular organismal signaling	35637	751	41	9.5	4.32	4.13E–13	53.4991
Biological process	Respiratory electron transport chain	22904	110	12	1.39	8.63	8.22E–07	52.51466
Biological process	Signal release	23061	344	23	4.35	5.29	8.37E–09	42.72878
Biological process	Generation of a signal involved in cell-cell signaling	3001	344	23	4.35	5.29	8.37E–09	42.72878
Biological process	Cellular respiration	45333	159	14	2.01	6.96	8.22E–07	42.35249
Biological process	Energy derivation by oxidation of organic compounds	15980	329	22	4.16	5.29	1.54E–08	41.32802
Biological process	Cell-cell signaling	7267	1099	48	13.9	3.45	3.98E–12	39.3304
Biological process	Generation of precursor metabolites and energy	6091	451	26	5.7	4.56	1.07E–08	36.34601
Biological process	Neurological system process	50877	1237	44	15.64	2.81	1.54E–08	21.95307
Biological process	Small molecule metabolic process	44281	2515	71	31.8	2.23	1.02E–09	20.05082
Biological process	Cell morphogenesis involved in neuron differentiation	48667	575	25	7.27	3.44	3.07E–06	18.96424
Biological process	Regulation of biological quality	65008	2511	70	31.75	2.2	2.49E–09	18.92836
Biological process	Neuron projection morphogenesis	48812	583	25	7.37	3.39	3.70E–06	18.4138
Biological process	Neuron development	48666	798	31	10.09	3.07	1.11E–06	18.28086
Biological process	Regulation of system process	44057	455	21	5.75	3.65	1.05E–05	18.17266
Biological process	Secretion by cell	32940	693	28	8.76	3.2	2.39E–06	17.98913
Biological process	Axonogenesis	7409	526	23	6.65	3.46	7.81E–06	17.67143
Biological process	Neuron projection development	31175	704	28	8.9	3.15	3.07E–06	17.36551
Biological process	Secretion	46903	795	30	10.05	2.98	3.07E–06	16.42833
Biological process	Neuron differentiation	30182	990	34	12.52	2.72	3.46E–06	14.85371
Biological process	Nervous system development	7399	1724	50	21.8	2.29	6.25E–07	14.20743
Biological process	Establishment of localization in cell	51649	1756	50	22.21	2.25	1.01E–06	13.49028
Biological process	Generation of neurons	48699	1073	35	13.57	2.58	6.71E–06	13.34706
Biological process	Cellular localization	51641	1977	53	25	2.12	2.31E–06	11.94914
Biological process	Establishment of localization	51234	3392	74	42.89	1.73	7.81E–06	8.835714
Biological process	Transport	6810	3338	73	42.21	1.73	8.58E–06	8.765067
Biological process	SinglE–multicellular organism process	44707	5612	106	70.97	1.49	4.87E–06	7.915582
Biological process	Multicellular organismal process	32501	5644	106	71.37	1.49	6.63E–06	7.715945
Molecular function	Malate dehydrogenase (oxaloacetate-decarboxylating) (NADP+) activity	4473	2	2	0.02	81.3	0.0023	214.4915
Molecular function	Malate dehydrogenase (oxaloacetate-decarboxylating) activity	16619	3	2	0.04	54.2	0.0039	130.5643
Molecular function	Thioredoxin peroxidase activity	8379	3	2	0.04	54.2	0.0039	130.5643
Molecular function	Malic enzyme activity	4470	4	2	0.05	40.65	0.0063	89.45681
Molecular function	Hydrolase activity, acting on carbon-nitrogen (but not peptide) bonds, in cyclic amides	16812	10	3	0.12	24.39	0.0023	64.34746
Molecular function	NAD binding	51287	49	7	0.6	11.61	0.0001	46.44
Molecular function	Calmodulin-dependent protein kinase activity	4683	21	4	0.26	15.49	0.0017	42.90035
Molecular function	Proline-rich region binding	70064	15	3	0.18	16.26	0.0062	35.89571
Molecular function	Syntaxin binding	19905	38	5	0.47	10.7	0.0017	29.6342
Molecular function	NADH dehydrogenase (ubiquinone) activity	8137	44	5	0.54	9.24	0.0023	24.37763
Molecular function	NADH dehydrogenase (quinone) activity	50136	44	5	0.54	9.24	0.0023	24.37763
Molecular function	NADH dehydrogenase activity	3954	44	5	0.54	9.24	0.0023	24.37763
Molecular function	SNARE binding	149	45	5	0.55	9.03	0.0023	23.8236
Molecular function	Cofactor binding	48037	262	15	3.22	4.65	7.65E–05	19.14097
Molecular function	Actin filament binding	51015	72	6	0.89	6.77	0.0033	16.79966
Molecular function	Coenzyme binding	50662	186	11	2.29	4.81	0.0005	15.87795
Molecular function	Oxidoreductase activity, acting on NADH or NADPH, quinone or similar compound as acceptor	16655	58	5	0.71	7.01	0.0062	15.47533
Molecular function	Calmodulin binding	5516	164	10	2.02	4.96	0.0008	15.36067
Molecular function	Hydrogen ion transmembrane transporter activity	15078	101	7	1.24	5.63	0.0023	14.85347
Molecular function	Cytoskeletal protein binding	8092	638	25	7.85	3.19	3.90E–05	14.0645
Molecular function	Oxidoreductase activity	16491	711	24	8.75	2.74	0.0003	9.652688
Molecular function	Actin binding	3779	356	14	4.38	3.2	0.0017	8.862563
Molecular function	GTPase activity	3924	231	10	2.84	3.52	0.0057	7.899321
Molecular function	Identical protein binding	42802	863	25	10.62	2.36	0.0012	6.893132
Molecular function	Anion binding	43168	2402	54	29.55	1.83	0.0002	6.769115
Molecular function	Small molecule binding	36094	2630	58	32.35	1.79	0.0002	6.621156
Molecular function	Protein binding	5515	7337	126	90.25	1.4	2.47E–05	6.450224
Molecular function	Nucleoside phosphate binding	1901265	2437	53	29.98	1.77	0.0005	5.842823
Molecular function	Nucleotide binding	166	2436	52	29.96	1.74	0.0007	5.489529
Molecular function	Nucleoside-triphosphatase activity	17111	760	21	9.35	2.25	0.0039	5.420105
Molecular function	Pyrophosphatase activity	16462	794	21	9.77	2.15	0.0063	4.731418
Molecular function	Hydrolase activity, acting on acid anhydrides, in phosphorus-containing anhydrides	16818	797	21	9.8	2.14	0.0063	4.709411
Molecular function	Catalytic activity	3824	5371	94	66.07	1.42	0.0005	4.687463
Molecular function	Hydrolase activity, acting on acid anhydrides	16817	802	21	9.86	2.13	0.0063	4.687405
Molecular function	Purine ribonucleoside triphosphate binding	35639	1829	38	22.5	1.69	0.0063	3.719114
Molecular function	Purine nucleoside binding	1883	1841	38	22.65	1.68	0.0063	3.697108
Molecular function	Purine ribonucleoside binding	32550	1838	38	22.61	1.68	0.0063	3.697108
Molecular function	Ribonucleoside binding	32549	1842	38	22.66	1.68	0.0063	3.697108
Molecular function	Nucleoside binding	1882	1852	38	22.78	1.67	0.0068	3.61971
Molecular function	Purine ribonucleotide binding	32555	1864	38	22.93	1.66	0.0073	3.546884
Cellular component	Synapse	45202	489	28	5.45	5.14	3.35E–11	53.84127
Cellular component	Mitochondrial respiratory chain	5746	69	8	0.77	10.41	6.79E–06	53.80024
Cellular component	Neuron projection	43005	651	33	7.25	4.55	1.02E–11	50.01087
Cellular component	Synaptic vesicle	8021	105	10	1.17	8.55	2.14E–06	48.47496
Cellular component	Axon	30424	286	19	3.19	5.96	8.47E–09	48.10981
cellular component	Respiratory chain	70469	76	8	0.85	9.45	1.32E–05	46.11058
Cellular component	Clathrin-coated vesicle membrane	30665	110	10	1.23	8.16	3.21E–06	44.82692
Cellular component	Vesicle membrane	12506	355	21	3.96	5.31	8.47E–09	42.86294
Cellular component	Cytoplasmic vesicle membrane	30659	342	20	3.81	5.25	2.52E–08	39.89265
Cellular component	Cytoplasm	5737	9130	170	101.72	1.67	1.52E–24	39.77632
Cellular component	Cytoplasmic part	44444	6772	143	75.45	1.9	1.86E–21	39.38793
Cellular component	Clathrin-coated vesicle	30136	207	14	2.31	6.07	7.60E–07	37.14346
Cellular component	Cell projection	42995	1230	45	13.7	3.28	2.51E–11	34.76907
Cellular component	Coated vesicle membrane	30662	150	11	1.67	6.58	6.79E–06	34.0063
Cellular component	Cytoplasmic vesicle part	44433	401	21	4.47	4.7	6.16E–08	33.88897
Cellular component	Cytosol	5829	2372	69	26.43	2.61	4.11E–13	32.32787
Cellular component	Coated vesicle	30135	254	15	2.83	5.3	1.44E–06	30.96068
Cellular component	Mitochondrion	5739	1525	48	16.99	2.83	5.17E–10	26.28082
Cellular component	Synapse part	44456	370	18	4.12	4.37	1.46E–06	25.50178
Cellular component	Membrane-bounded vesicle	31988	883	32	9.84	3.25	4.82E–08	23.7801
Cellular component	Mitochondrial inner membrane	5743	354	17	3.94	4.31	3.58E–06	23.47276
Cellular component	Cytoplasmic membrane-bounded vesicle	16023	862	31	9.6	3.23	8.13E–08	22.90041
Cellular component	Mitochondrial part	44429	744	27	8.29	3.26	5.82E–07	20.32635
Cellular component	Organelle inner membrane	19866	384	17	4.28	3.97	1.01E–05	19.83284
Cellular component	Vesicle	31982	970	32	10.81	2.96	3.20E–07	19.22476
Cellular component	Cytoplasmic vesicle	31410	928	31	10.34	3	3.93E–07	19.21682
Cellular component	Intracellular part	44424	12237	179	136.34	1.31	2.42E–13	16.5272
Cellular component	Intracellular	5622	12564	180	139.98	1.29	1.32E–12	15.32446
Cellular component	Organelle membrane	31090	2373	57	26.44	2.16	8.13E–08	15.3142
Cellular component	Intracellular organelle part	44446	6725	120	74.93	1.6	5.80E–10	14.77852
Cellular component	Organelle part	44422	6812	120	75.9	1.58	1.42E–09	13.97938
Cellular component	Cytoskeleton	5856	1790	45	19.94	2.26	1.06E–06	13.50281
Cellular component	Protein complex	43234	3278	70	36.52	1.92	9.89E–08	13.44922
Cellular component	Cell part	44464	14643	189	163.15	1.16	1.90E–10	11.27665
Cellular component	Cell	5623	14644	189	163.16	1.16	1.90E–10	11.27665
Cellular component	Macromolecular complex	32991	3864	76	43.05	1.77	5.44E–07	11.08799
Cellular component	Intracellular organelle	43229	10636	155	118.5	1.31	8.09E–08	9.290587
Cellular component	Organelle	43226	10651	155	118.67	1.31	8.13E–08	9.287781
Cellular component	Intracellular membrane-bounded organelle	43231	9587	138	106.82	1.29	1.32E–05	6.29446
Cellular component	Membrane-bounded organelle	43227	9598	138	106.94	1.29	1.36E–05	6.277735

GO term annotation of the cortical proteins possessing BTBR:WT iTRAQ expression ratios <1.2 or <0.8 were created using WebGestalt. Each GO term group significantly populated (p<0.05) by at least two independent proteins are depicted. For each GO Term group the specific GO term ID, number of total proteins in the curated GO term cluster (C), the number of observed experimental proteins in the GO term group (O), the expected number of proteins in the group based on whole-genome background frequency (E), the GO term group enrichment factor (R), the GO term enrichment probability (P) and the hybrid score (R ^*^ −log_10_ P) are indicated (H).

### Signaling pathway analysis of differentially regulated BTBR-specific proteins

**Figure 5:**
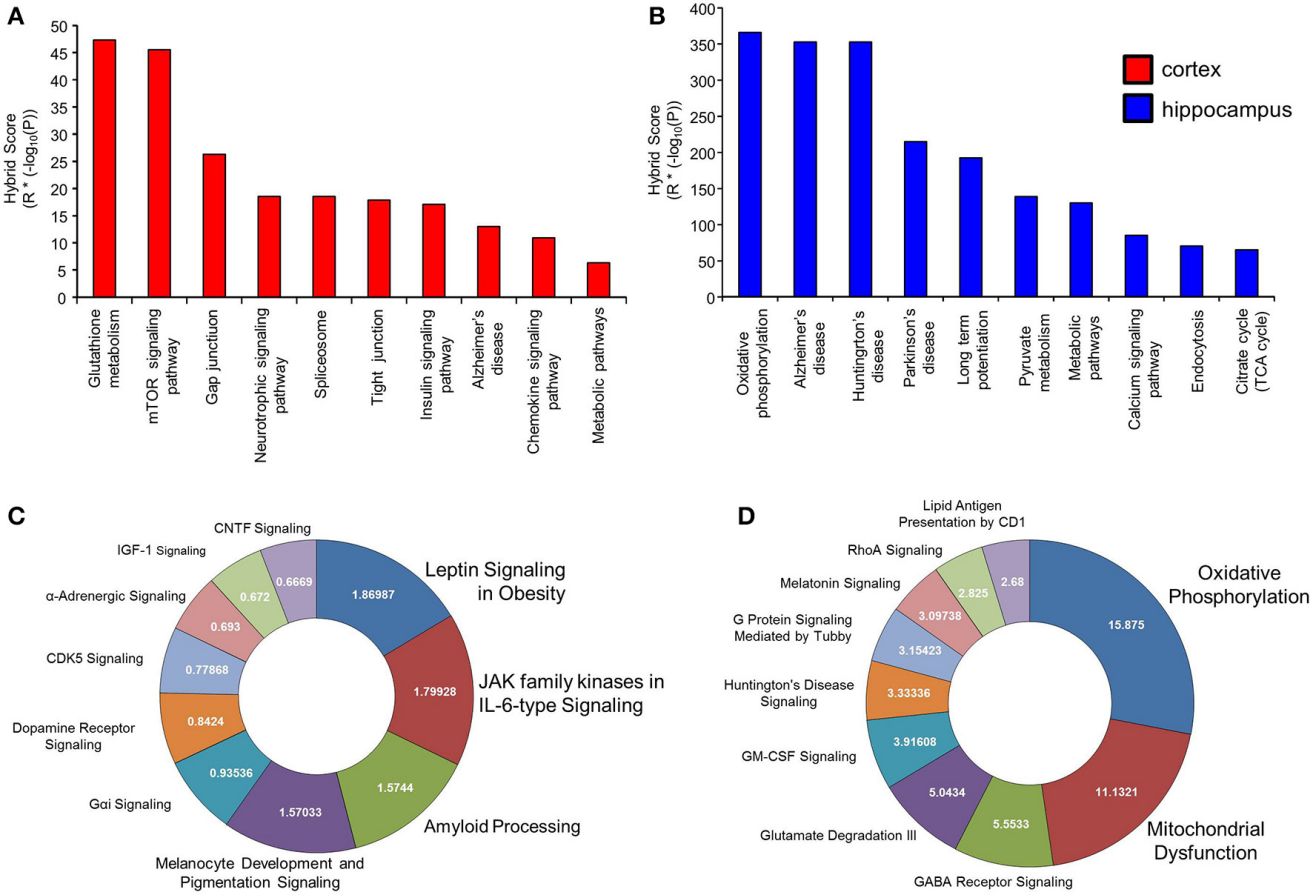
**Signaling pathway analysis of cortical and hippocampal proteins differentially regulated BTBR and WT**. Cortical and hippocampal proteins exhibiting differential regulation between BTBR and control mice were analyzed for potential functional pathway interactions using KEGG (**A**-cortex, **B**-hippocampus) and Ingenuity Canonical Signaling pathway analysis (**C**-cortex, **D**-hippocampus). For **(A,B)**, the top 10 most significantly-populated (calculated with Hybrid scores: enrichment factor (R) ^*^ (−log_10_ enrichment probability, P) neuronally-specific KEGG or Canonical signaling pathways are shown.

While GO annotation indicates some degree of functional commonality between groups of proteins, associating multiple proteins with classically-identified molecular signaling paradigms provides additional functional information regarding the potential physiological ramifications of specific group of differentially-regulated factors. We therefore performed both KEGG and Ingenuity-base pathway analysis upon our cortical and hippocampal BTBR protein sets. Inspecting the top ten highest scoring KEGG pathways from cortical proteins (Figure 5A: Table 3) a strong population of metabolic (*Glutathione metabolism*, *Insulin signaling pathway*, *Metabolic pathways*), neurotransmission/degenerative (*Neurotrophic signaling pathway*, *Alzheimer's disease*) and ultrastructural (*Gap junction*, *Tight junction*, *Spliceosome*) signaling pathways was evident. Applying the same analysis to the hippocampus an intense population of signaling systems linked to oxidative metabolism [*Oxidative phosphorylation*, *Pyruvate metabolism*, *Citrate cycle (TCA cycle)*] and central neurodegeneration (*Alzheimer's disease*, *Huntington's disease*, *Parkinson's disease*, *Long-term potentiation*) was clear (Figure 5B: Table 4). Extraction of potential physiological meaning from complex data sets is best performed using multiple informatics tools. Therefore, we also sought interpretation of the potential signaling pathways enriched in the BTBR tissues using canonical signaling pathway analysis (Ingenuity). Canonical signaling analysis of the cortex revealed a strong bias toward cytokine signaling (*Leptin signaling in obesity*, *JAK family kinases in IL-6-type signaling*), neurodegenerative disease related activity (*Amyloid processing*, *CDK5 signaling*, *CNTF signaling*) and energy metabolism (IGF-1 signaling) (Figure 5C: Table 5). Similar pathway processing of hippocampal data again revealed a strong prediction of metabolism-related activity (*Oxidative phosphorylation*, *Mitochondrial dysfunction*, *G protein signaling mediated by Tubby*), neurodegenerative activity (*Huntington's disease signaling*) and interestingly, given the strong presentation of, *Melanocyte development and pigmentation signaling'* in the cortex (Figure 5C), melatonin-related activity (*Melatonin signaling*) (Figure 5D: Table 6).

**Table 3:** **KEGG Pathway analysis for cortical proteins altered in BTBR mice compared to WT controls**.

**KEGG pathway**	***C***	***E***	***O***	***R***	***P***	***H***
Glutathione metabolism	50	2	0.07	27.38	0.0188	47.25356
MTOR signaling pathway	52	2	0.08	26.33	0.0188	45.44142
Arginine and proline metabolism	54	2	0.08	25.35	0.0188	43.7501
Shigellosis	61	2	0.09	22.44	0.0188	38.7279
Renal cell carcinoma	70	2	0.1	19.56	0.0188	33.75747
Chronic myeloid leukemia	73	2	0.11	18.75	0.0188	32.35954
Progesterone-mediated oocyte maturation	86	2	0.13	15.92	0.0188	27.47541
Gap junction	90	2	0.13	15.21	0.0188	26.25006
Melanogenesis	101	2	0.15	13.56	0.0192	23.27844
GnRH signaling pathway	101	2	0.15	13.56	0.0192	23.27844
Oocyte meiosis	112	2	0.16	12.22	0.0192	20.97806
Leukocyte transendothelial migration	116	2	0.17	11.8	0.0192	20.25705
Vascular smooth muscle contraction	116	2	0.17	11.8	0.0192	20.25705
Neurotrophin signaling pathway	127	2	0.19	10.78	0.0192	18.50601
Spliceosome	127	2	0.19	10.78	0.0192	18.50601
Tight junction	132	2	0.19	10.37	0.0192	17.80217
Hepatitis C	134	2	0.2	10.22	0.0192	17.54466
Natural killer cell mediated cytotoxicity	136	2	0.2	10.07	0.0192	17.28716
Insulin signaling pathway	138	2	0.2	9.92	0.0192	17.02965
Alzheimer's disease	167	2	0.24	8.2	0.0261	12.98355
Chemokine signaling pathway	189	2	0.28	7.24	0.0313	10.89226
Metabolic pathways	1130	6	1.65	3.63	0.0188	6.264807

KEGG signaling pathway annotation of the cortical proteins possessing BTBR:WT iTRAQ expression ratios < 1.2 or < 0.8 was created using WebGestalt. Each KEGG pathway significantly populated (p < 0.05) by at least two independent proteins are depicted. For each populated KEGG pathway the number of total proteins in the curated KEGG pathway (C), the number of observed experimental proteins in the KEGG pathway (O), the expected number of proteins in the pathway based on whole-genome background frequency (E), the KEGG pathway enrichment factor (R), the KEGG pathway enrichment probability (P) and the hybrid score (R ^*^ −log_10_ P) are indicated (H).

**Table 4:** **KEGG Pathway analysis for hippocampal proteins altered in BTBR mice compared to WT controls**.

**KEGG pathway**	***C***	***E***	***O***	***R***	***P***	***H***
Oxidative phosphorylation	132	15	0.61	24.63	1.42E–15	365.6991
Alzheimer's disease	167	17	0.77	22.06	1.03E–16	352.6768
Huntington's disease	183	18	0.84	21.32	2.92E–17	352.518
Parkinson's disease	130	12	0.6	20	1.88E–11	214.5168
Long–term potentiation	70	8	0.32	24.77	1.74E–08	192.2016
Gastric acid secretion	74	8	0.34	23.43	2.34E–08	178.7893
Collecting duct acid secretion	27	4	0.12	32.11	4.01E–05	141.183
Pyruvate metabolism	40	5	0.18	27.09	7.57E–06	138.7253
Metabolic pathways	1130	37	5.21	7.1	5.38E–19	129.7114
GnRH signaling pathway	101	8	0.47	17.17	2.19E–07	114.3446
Oocyte meiosis	112	8	0.52	15.48	4.04E–07	98.97322
Calcium signaling pathway	177	10	0.82	12.24	1.14E–07	84.98348
Vasopressin-regulated water reabsorption	44	4	0.2	19.7	0.0002	72.86971
Endocytosis	201	10	0.93	10.78	3.03E–07	70.27005
Citrate cycle (TCA cycle)	30	3	0.14	21.67	0.001	65.01
Butanoate metabolism	30	3	0.14	21.67	0.001	65.01
Propanoate metabolism	32	3	0.15	20.32	0.001	60.96
Alanine, aspartate and glutamate metabolism	32	3	0.15	20.32	0.001	60.96
Melanogenesis	101	6	0.47	12.87	4.15E–05	56.39572
Vibrio cholerae infection	54	4	0.25	16.05	0.0004	54.53694
Shigellosis	61	4	0.28	14.21	0.0007	44.83116
Wnt signaling pathway	150	7	0.69	10.11	4.01E–05	44.45221
Salivary secretion	89	5	0.41	12.18	0.0003	42.90866
Gap junction	90	5	0.42	12.04	0.0003	42.41546
Beta-Alanine metabolism	22	2	0.1	19.7	0.0071	42.33021
Glioma	65	4	0.3	13.34	0.0007	42.08639
Neurotrophin signaling pathway	127	6	0.59	10.24	0.0001	40.96
Valine, leucine and isoleucine degradation	44	3	0.2	14.78	0.0023	38.99366
Proteasome	44	3	0.2	14.78	0.0023	38.99366
Epithelial cell signaling in Helicobacter pylori infection	68	4	0.31	12.75	0.0009	38.83341
PPAR signaling pathway	70	4	0.32	12.38	0.0009	37.70648
Long-term depression	70	4	0.32	12.38	0.0009	37.70648
Bile secretion	71	4	0.33	12.21	0.0009	37.1887
Cardiac muscle contraction	77	4	0.36	11.26	0.0012	32.88842
Phosphatidylinositol signaling system	78	4	0.36	11.11	0.0012	32.4503
Pentose phosphate pathway	27	2	0.12	16.05	0.0103	31.89396
Amyotrophic lateral sclerosis (ALS)	53	3	0.24	12.27	0.0036	29.98417
ErbB signaling pathway	87	4	0.4	9.96	0.0016	27.84696
Inositol phosphate metabolism	57	3	0.26	11.41	0.0043	27.00212
Regulation of actin cytoskeleton	213	7	0.98	7.12	0.0003	25.0829
Glycolysis/Gluconeogenesis	65	3	0.3	10	0.0057	22.44125
Prion diseases	35	2	0.16	12.38	0.0158	22.30063
African trypanosomiasis	35	2	0.16	12.38	0.0158	22.30063
Pancreatic secretion	101	4	0.47	8.58	0.0026	22.17953
Adipocytokine signaling pathway	68	3	0.31	9.56	0.0064	20.97292
Bacterial invasion of epithelial cells	70	3	0.32	9.29	0.0066	20.25644
Renal cell carcinoma	70	3	0.32	9.29	0.0066	20.25644
Vascular smooth muscle contraction	116	4	0.54	7.47	0.0039	17.99475
Antigen processing and presentation	76	3	0.35	8.55	0.0081	17.88245
Purine metabolism	162	5	0.75	6.69	0.0023	17.65004
Protein processing in endoplasmic reticulum	165	5	0.76	6.57	0.0023	17.33345
Lysosome	121	4	0.56	7.16	0.0044	16.87288
Spliceosome	127	4	0.59	6.83	0.005	15.71603
Chemokine signaling pathway	189	5	0.87	5.73	0.0036	14.00239
Rheumatoid arthritis	91	3	0.42	7.14	0.0127	13.53884
Fc gamma R-mediated phagocytosis	94	3	0.43	6.92	0.0136	12.91591
Taste transduction	52	2	0.24	8.34	0.0313	12.54716
MAPK signaling pathway	268	6	1.24	4.85	0.0032	12.10002
Arginine and proline metabolism	54	2	0.25	8.03	0.0325	11.94958
Pathogenic Escherichia coli infection	56	2	0.26	7.74	0.0342	11.34664
Chagas disease (American trypanosomiasis)	104	3	0.48	6.25	0.017	11.05969
Amoebiasis	106	3	0.49	6.13	0.0177	10.73992
Toxoplasmosis	132	3	0.61	4.93	0.0309	7.444505
Natural killer cell mediated cytotoxicity	136	3	0.63	4.78	0.0324	7.119595
Phagosome	153	3	0.71	4.25	0.0416	5.868853

KEGG signaling pathway annotation of the hippocampal proteins possessing BTBR:WT iTRAQ expression ratios < 1.2 or < 0.8 was created using WebGestalt. Each KEGG pathway significantly populated (p < 0.05) by at least two independent proteins are depicted. For each populated KEGG pathway the number of total proteins in the curated KEGG pathway (C), the number of observed experimental proteins in the KEGG pathway (O), the expected number of proteins in the pathway based on whole-genome background frequency (E), the KEGG pathway enrichment factor (R), the KEGG pathway enrichment probability (P) and the hybrid score (R ^*^ −log_10_ P) are indicated (H).

**Table 5:** **Ingenuity Canonical Signaling Pathway analysis of proteins differentially regulated in the cortex of aged BTBR compared to WT controls**.

**Canonical pathways**	**log(*p*-value)**	**Ratio**	**10 × Hybrid**
Leptin Signaling in Obesity	3.97E + 00	4.71E–02	1.86987
Role of JAK family kinases in IL-6-type Cytokine Signaling	2.52E + 00	7.14E–02	1.79928
Amyloid Processing	3.20E + 00	4.92E–02	1.5744
Melanocyte Development and Pigmentation Signaling	3.73E + 00	4.21E–02	1.57033
Gαi Signaling	3.16E + 00	2.96E–02	0.93536
Dopamine Receptor Signaling	2.70E + 00	3.12E–02	0.8424
CDK5 Signaling	2.52E + 00	3.09E–02	0.77868
α-Adrenergic Signaling	2.52E + 00	2.75E–02	0.693
IGF-1 Signaling	2.40E + 00	2.80E–02	0.672
CNTF Signaling	1.90E + 00	3.51E–02	0.6669
Neuropathic Pain Signaling In Dorsal Horn Neurons	2.36E + 00	2.75E–02	0.649
G Beta Gamma Signaling	2.50E + 00	2.48E–02	0.62
IL-2 Signaling	1.88E + 00	3.28E–02	0.61664
Thrombopoietin Signaling	1.85E + 00	3.12E–02	0.5772
Renin-Angiotensin Signaling	2.26E + 00	2.38E–02	0.53788
Gαs Signaling	2.22E + 00	2.40E–02	0.5328
Role of JAK1 and JAK3 in γc Cytokine Signaling	1.75E + 00	2.94E–02	0.5145
GM-CSF Signaling	1.74E + 00	2.94E–02	0.51156
Clathrin-mediated Endocytosis Signaling	2.49E + 00	2.02E–02	0.50298
Regulation of Cellular Mechanics by Calpain Protease	1.82E + 00	2.74E–02	0.49868
CREB Signaling in Neurons	2.58E + 00	1.93E–02	0.49794
Glutamate Receptor Signaling	1.79E + 00	2.78E–02	0.49762
Synaptic Long Term Potentiation	2.14E + 00	2.31E–02	0.49434
Antiproliferative Role of Somatostatin Receptor 2	1.73E + 00	2.78E–02	0.48094
Agrin Interactions at Neuromuscular Junction	1.68E + 00	2.86E–02	0.48048
JAK/Stat Signaling	1.69E + 00	2.82E–02	0.47658
Corticotropin Releasing Hormone Signaling	2.21E + 00	2.07E–02	0.45747
Neurotrophin/TRK Signaling	1.68E + 00	2.63E–02	0.44184
P2Y Purigenic Receptor Signaling Pathway	2.12E + 00	2.08E–02	0.44096
Renal Cell Carcinoma Signaling	1.65E + 00	2.53E–02	0.41745
Insulin Receptor Signaling	2.03E + 00	2.01E–02	0.40803
Melatonin Signaling	1.65E + 00	2.47E–02	0.40755
FLT3 Signaling in Hematopoietic Progenitor Cells	1.60E + 00	2.53E–02	0.4048
GNRH Signaling	2.05E + 00	1.96E–02	0.4018
cAMP-mediated signaling	2.23E + 00	1.77E–02	0.39471
Ephrin B Signaling	1.61E + 00	2.44E–02	0.39284
Prolactin Signaling	1.62E + 00	2.38E–02	0.38556
BMP signaling pathway	1.59E + 00	2.33E–02	0.37047
Relaxin Signaling	1.98E + 00	1.83E–02	0.36234
FGF Signaling	1.49E + 00	2.13E–02	0.31737
Gap Junction Signaling	1.83E + 00	1.66E–02	0.30378
Apoptosis Signaling	1.47E + 00	2.00E–02	0.294
Acute Phase Response Signaling	1.74E + 00	1.66E–02	0.28884
G-Protein Coupled Receptor Signaling	1.99E + 00	1.45E–02	0.28855
Mouse Embryonic Stem Cell Pluripotency	1.41E + 00	2.02E–02	0.28482
Neuregulin Signaling	1.47E + 00	1.92E–02	0.28224
FAK Signaling	1.48E + 00	1.89E–02	0.27972
Chronic Myeloid Leukemia Signaling	1.42E + 00	1.89E–02	0.26838
VEGF Signaling	1.43E + 00	1.83E–02	0.26169
IL-1 Signaling	1.42E + 00	1.83E–02	0.25986
Sertoli Cell-Sertoli Cell Junction Signaling	1.69E + 00	1.52E–02	0.25688
PPARα/RXRα Activation	1.69E + 00	1.50E–02	0.2535
HGF Signaling	1.37E + 00	1.80E–02	0.2466
Mitochondrial Dysfunction	1.76E + 00	1.40E–02	0.2464
Ephrin Receptor Signaling	1.70E + 00	1.43E–02	0.2431
Role of NFAT in Cardiac Hypertrophy	1.66E + 00	1.44E–02	0.23904
Calcium Signaling	1.67E + 00	1.38E–02	0.23046
Integrin Signaling	1.57E + 00	1.44E-02	0.22608
Leukocyte Extravasation Signaling	1.56E + 00	1.43E-02	0.22308
Breast Cancer Regulation by Stathmin1	1.59E + 00	1.40E–02	0.2226
Fc Epsilon RI Signaling	1.30E + 00	1.71E–02	0.2223
Nitric Oxide Signaling in the Cardiovascular System	1.36E + 00	1.60E–02	0.2176
NGF Signaling	1.32E + 00	1.64E–02	0.21648
Axonal Guidance Signaling	1.86E + 00	1.03E–02	0.19158
Cardiac Hypertrophy Signaling	1.42E + 00	1.20E–02	0.1704
Molecular Mechanisms of Cancer	1.58E + 00	1.03E–02	0.16274
Colorectal Cancer Metastasis Signaling	1.34E + 00	1.12E–02	0.15008
Protein Kinase A Signaling	1.43E + 00	9.78E–03	0.139854

Canonical signaling pathway annotation of the cortical proteins possessing BTBR:WT iTRAQ expression ratios <1.2 or <0.8 was created using Ingenuity Pathway Analysis. Each signaling pathway significantly populated (p < 0.05) by at least two independent proteins are depicted. For each populated signaling pathway the −log_10_ of the enrichment probability (p-value), the enrichment ratio and the calculated hybrid score [10 ^*^ (enrichment ratio ^*^ −log_10_(p-value))] are depicted.

**Table 6:** **Ingenuity Canonical Signaling Pathway analysis of proteins differentially regulated in the hippocampus of aged BTBR compared to WT controls**.

**Canonical pathways**	**−log(*p*-value)**	**Ratio**	**10 × Hybrid**
Oxidative phosphorylation	1.27E + 01	1.25E–01	15.875
Mitochondrial dysfunction	1.33E + 01	8.37E–02	11.1321
GABA receptor signaling	5.19E + 00	1.07E–01	5.5533
Glutamate degradation III (via 4-aminobutyrate)	3.02E + 00	1.67E–01	5.0434
GM-CSF signaling	4.44E + 00	8.82E–02	3.91608
Huntington's disease signaling	6.46E + 00	5.16E–02	3.33336
G protein signaling mediated by Tubby	3.47E + 00	9.09E–02	3.15423
Melatonin signaling	4.18E + 00	7.41E–02	3.09738
RhoA signaling	4.52E + 00	6.25E–02	2.825
Lipid antigen presentation by CD1	2.68E + 00	1.00E–01	2.68
Regulation of actin-based motility by rho	3.77E + 00	6.52E–02	2.45804
Role of NFAT in cardiac hypertrophy	4.87E + 00	4.78E–02	2.32786
CTLA4 Signaling in cytotoxic T lymphocytes	3.63E + 00	6.25E–02	2.26875
TCA Cycle II (Eukaryotic)	2.90E + 00	7.50E–02	2.175
Breast cancer regulation by stathmin1	4.65E + 00	4.67E–02	2.17155
Chemokine signaling	3.22E + 00	6.67E–02	2.14774
Synaptic long term potentiation	3.72E + 00	5.38E–02	2.00136
Guanine and guanosine salvage I	1.71E + 00	1.11E–01	1.8981
CREB signaling in neurons	4.21E + 00	4.35E–02	1.83135
β-alanine degradation I	1.71E + 00	1.00E–01	1.71
Axonal guidance signaling	5.19E + 00	3.29E–02	1.70751
GNRH signaling	3.51E + 00	4.58E–02	1.60758
Actin nucleation by ARP-WASP complex	2.65E + 00	5.97E–02	1.58205
4-Aminobutyrate degradation I	1.53E + 00	1.00E–01	1.53
Superpathway of inositol phosphate compounds	3.94E + 00	3.85E–02	1.5169
Creatine-phosphate biosynthesis	1.32E + 00	1.11E–01	1.4652
Pyruvate fermentation to lactate	1.32E + 00	1.11E–01	1.4652
D-myo-inositol-5-phosphate metabolism	3.32E + 00	4.32E–02	1.43424
fMLP signaling in neutrophils	3.13E + 00	4.55E–02	1.42415
Methylglyoxal degradation I	1.53E + 00	9.09E–02	1.39077
Clathrin-mediated endocytosis signaling	3.29E + 00	4.04E–02	1.32916
Ephrin receptor signaling	3.45E + 00	3.81E–02	1.31445
Protein kinase A signaling	3.95E + 00	3.18E–02	1.2561
Thrombin signaling	3.20E + 00	3.79E–02	1.2128
3-phosphoinositide biosynthesis	3.10E + 00	3.87E–02	1.1997
D-myo-inositol (1,4,5,6)-tetrakisphosphate biosynthesis	2.86E + 00	4.17E–02	1.19262
D-myo-inositol (3,4,5,6)-tetrakisphosphate biosynthesis	2.86E + 00	4.17E–02	1.19262
Methylmalonyl pathway	1.41E + 00	8.33E–02	1.17453
Neuropathic pain signaling in dorsal horn neurons	2.50E + 00	4.59E–02	1.1475
G beta gamma signaling	2.73E + 00	4.13E–02	1.12749
nNOS signaling in neurons	1.95E + 00	5.77E–02	1.12515
Signaling by rho family GTPases	3.24E + 00	3.36E–02	1.08864
Arginine degradation I (arginase pathway)	1.41E + 00	7.69E–02	1.08429
Ephrin B signaling	2.21E + 00	4.88E–02	1.07848
Purine nucleotides de novo biosynthesis II	2.22E + 00	4.76E–02	1.05672
Leptin signaling in obesity	2.19E + 00	4.71E–02	1.03149
Semaphorin signaling in neurons	1.83E + 00	5.56E–02	1.01748
Inosine-5′-phosphate biosynthesis II	1.53E + 00	6.25E–02	0.95625
Actin cytoskeleton signaling	2.88E + 00	3.31E–02	0.95328
3-phosphoinositide degradation	2.57E + 00	3.70E–02	0.9509
Rac signaling	2.43E + 00	3.91E–02	0.95013
iCOS-iCOSL signaling in T helper cells	2.38E + 00	3.97E–02	0.94486
Dopamine receptor signaling	2.17E + 00	4.17E–02	0.90489
Molybdenum cofactor biosynthesis	1.41E + 00	6.25E–02	0.88125
Aldosterone signaling in epithelial cells	2.38E + 00	3.55E–02	0.8449
CD28 signaling in T helper cells	2.23E + 00	3.68E–02	0.82064
B cell receptor signaling	2.30E + 00	3.43E–02	0.7889
2-oxobutanoate degradation I	1.32E + 00	5.88E–02	0.77616
PKCθ signaling in T lymphocytes	2.23E + 00	3.47E–02	0.77381
Virus entry via endocytic pathways	1.93E + 00	3.96E–02	0.76428
P2Y purigenic receptor signaling pathway	2.14E + 00	3.47E–02	0.74258
D-myo-inositol (1,4,5)-trisphosphate biosynthesis	1.48E + 00	5.00E–02	0.74
Dopamine-DARPP32 feedback in cAMP signaling	2.25E + 00	3.21E–02	0.72225
PI3K signaling in B lymphocytes	2.05E + 00	3.50E–02	0.7175
α-Adrenergic signaling	1.95E + 00	3.67E–02	0.71565
Protein ubiquitination pathway	2.41E + 00	2.96E–02	0.71336
Gluconeogenesis I	1.67E + 00	4.26E–02	0.71142
Glutamate receptor signaling	1.69E + 00	4.17E–02	0.70473
G-Protein coupled receptor signaling	2.38E + 00	2.90E–02	0.6902
IL-1 signaling	1.87E + 00	3.67E–02	0.68629
Antiproliferative role of somatostatin receptor 2	1.60E + 00	4.17E–02	0.6672
Remodeling of epithelial adherens junctions	1.53E + 00	4.29E–02	0.65637
JAK/stat signaling	1.54E + 00	4.23E–02	0.65142
Glioma signaling	1.84E + 00	3.54E–02	0.65136
Role of NFAT in regulation of the immune response	2.15E + 00	3.00E–02	0.645
Fatty acid β-oxidation I	1.45E + 00	4.44E–02	0.6438
RhoGDI signaling	2.11E + 00	2.97E–02	0.62667
Cardiac β-adrenergic signaling	1.96E + 00	3.16E–02	0.61936
Cardiac hypertrophy signaling	2.15E + 00	2.80E–02	0.602
Relaxin signaling	1.93E + 00	3.05E–02	0.58865
Renal cell carcinoma signaling	1.50E + 00	3.80E–02	0.57
Calcium signaling	2.05E + 00	2.76E–02	0.5658
Phospholipase C signaling	2.06E + 00	2.64E–02	0.54384
Gαq signaling	1.80E + 00	2.92E–02	0.5256
Gαs signaling	1.60E + 00	3.20E–02	0.512
Molecular mechanisms of cancer	2.15E + 00	2.32E–02	0.4988
Role of tissue factor in cancer	1.61E + 00	3.08E–02	0.49588
Gap junction signaling	1.71E + 00	2.76E–02	0.47196
p70S6K signaling	1.51E + 00	3.03E-02	0.45753
Androgen signaling	1.60E + 00	2.76E–02	0.4416
Gαi signaling	1.48E + 00	2.96E–02	0.43808
cAMP-mediated signaling	1.65E + 00	2.65E–02	0.43725
PPARα/RXRα activation	1.51E + 00	2.50E–02	0.3775
Insulin receptor signaling	1.38E + 00	2.68E–02	0.36984
eNOS signaling	1.39E + 00	2.58E–02	0.35862
Glucocorticoid receptor signaling	1.35E + 00	2.01E–02	0.27135

Canonical signaling pathway annotation of the hippocampal proteins possessing BTBR:WT iTRAQ expression ratios < 1.2 or < 0.8 was created using Ingenuity Pathway Analysis. Each signaling pathway significantly populated (p < 0.05) by at least two independent proteins are depicted. For each populated signaling pathway the −log_10_ of the enrichment probability (p-value), the enrichment ratio and the calculated hybrid score [10 ^*^ (enrichment ratio ^*^ −log_10_(p-value))] are depicted.

### BTBR-regulated coherent protein regulation patterns

**Figure 6:**
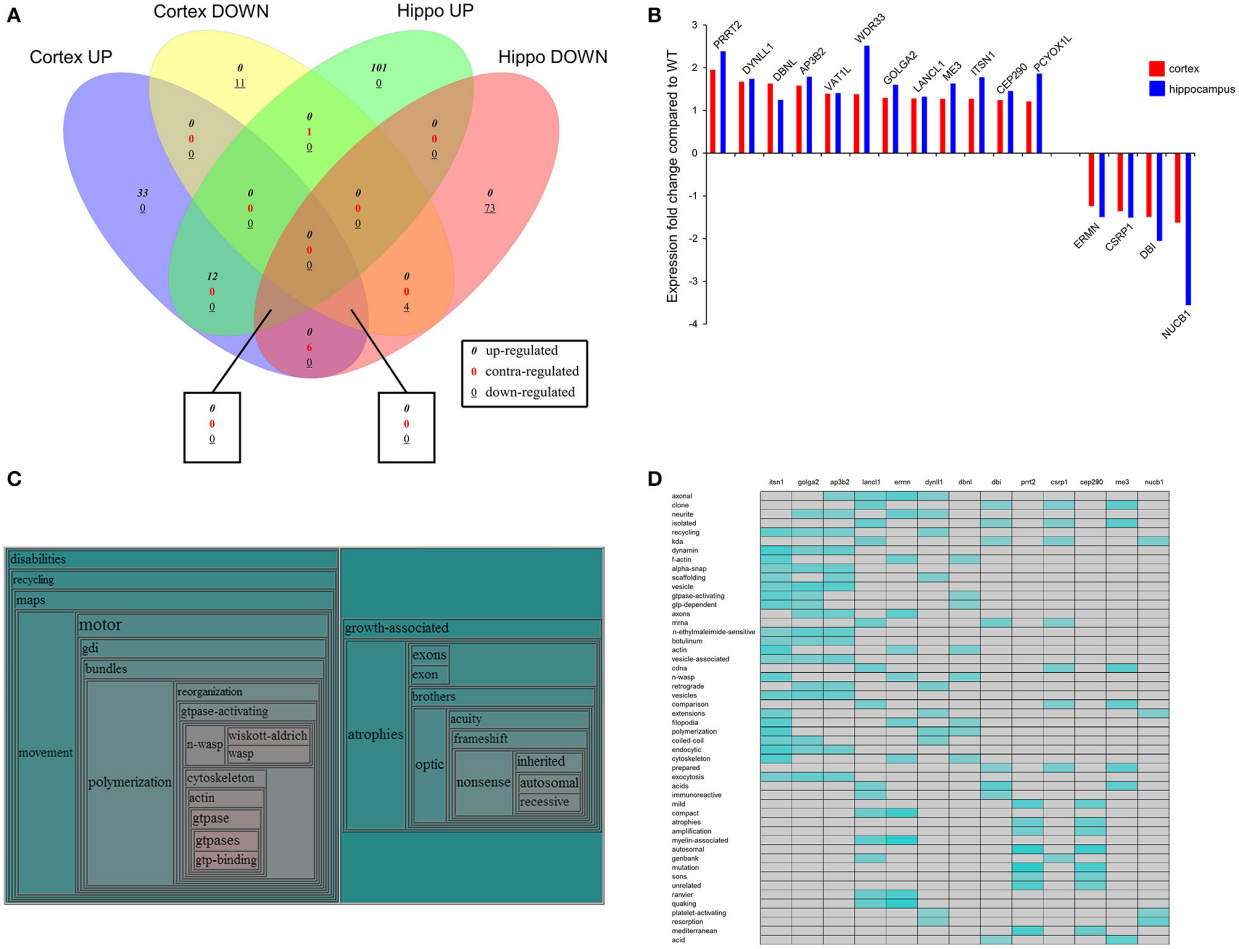
**Identification of coherent regulatory protein signatures in BTBR CNS tissues**. Vennplex diagram analysis of the upregulated (BTBR:WT iTRAQ ratio > 1.2) or downregulated (iTRAQ ratio < 0.8) proteins in both the hippocampus and cortex are depicted **(A)**. A small coherently-regulated protein subset across both tissues was evident and comprised a focused protein set strongly linked to human ASD-like conditions **(B)**. *Textrous!*-based collective processing interrogation of the coherently-regulated BTBR protein set revealed, in the resultant hierarchical word-cloud, a strong potential role of skeletal modeling activity in this protein set **(C)**. This skeletal signaling dependence of ASD was confirmed using the individual processing module of *Textrous!* **(D)**. In this heatmap output correlation strength between protein and functional term is indicated by the intensity of the teal-colored blocks.

Along with our global analysis of protein set data from the two tissues we also investigated the predicted functional nature of the divergent expression polarity subsets of BTBR-specific proteins uniquely regulated in either the cortex or hippocampus. Using VennPlex (Figure 6A: Table 7) (Cai et al., 2013) we were able to identify multiple groups of coherently-controlled proteins across multiple CNS tissues. Using our previously developed informatics application, *Textrous!*, we generated physiological predictions extracted from multiple databases using latent semantic analysis (Chen et al., 2013). Using the collective processing capacity of *Textrous!*. which generates interactive hierarchical word-clouds, we found that the tissue-unique up or down regulated sets of proteins generated quite distinct functional signatures in the cortex or hippocampus. For example, the specifically upregulated proteins in the cortex were strongly focused into activity controlling excitatory amino acid synaptic activity (Figure S1A: Table S3), while the downregulated cortical only proteins demonstrated a strong link to a reduction of protein kinase activity (Figure S1B: Table S4). With respect to the hippocampus, the upregulated protein set appeared to be linked to increases in heat-shock factors and synaptic pathophysiology (Figure S2A: Table S5), while the downregulated hippocampal protein set suggested a reduction in oligodendrocyte generation and CNS myelination (Figure S2B: Table S6).

**Table 7:** **VennPlex Venn diagram analysis of up and downregulated proteins across the cortex and hippocampus of BTBR mice compared to WT controls**.

**Protein**	**Cortex up**	**Cortex down**	**Hippo up**	**Hippo down**
PRRT2	1.951168338		2.381953722	
DYNLL1	1.672920918		1.737545789	
DBNL	1.626693344		1.24548243	
AP3B2	1.581785753		1.789312776	
VAT1L	1.391942674		1.401775589	
WDR33	1.379833997		2.510567518	
GOLGA2	1.292500223		1.60040989	
LANCL1	1.279121599		1.320074872	
ME3	1.270939623		1.62730561	
ITSN1	1.269638936		1.774926636	
CEP290	1.238621608		1.454207016	
PCYOX1L	1.209317219		1.860882083	
ERMN		−1.243267187		−1.500264003
CSRP1		−1.365689412		−1.509867503
DBI		−1.496288514		−2.056916039
NUCB1		−1.631205729		−3.561025937
EIF5A	1.517756095			−1.65673365
MMAA	1.391228352			−1.887879989
PTPN11	1.247745108			−3.093767449
ZXDB	1.214676409			−1.393599577
SLC17A7	1.204082729			−1.315805498
DPYSL4	1.195193712			−1.314442062
OAT		−1.307905257	1.362629072	
Ptcd	1.828557315			
PCBP2	1.792502849			
QDPR	1.7625157			
SRSF4	1.533013648			
ADCY2	1.511246891			
NDUFS4	1.47998035			
SLC9A3R1	1.473396618			
LETM1	1.455678341			
PRDX3	1.44243043			
IDH2	1.441010435			
EXOC5	1.41342868			
HNRNPUL2	1.405691244			
PTCD1	1.371753507			
DDX5	1.365841661			
PPP1R9B	1.346708939			
DPP6	1.340020376			
LAP3	1.303333413			
KCNAB2	1.296085587			
SLC12A5	1.252190507			
HOOK3	1.250023514			
COX5A	1.247002966			
WDR1	1.244912709			
GRM2	1.243617897			
CAB39	1.2340405			
RTN3	1.233971862			
TF	1.233243832			
FBXO41	1.231290747			
RAB7A	1.226744118			
RTN4	1.216638369			
TCP1	1.215498024			
PRKAR2B	1.215235782			
CCT3	1.20748828			
PRDM5	1.198502382			
COQ9		−1.24961385		
CLDN11		−1.260614004		
NCALD		−1.266374541		
F5		−1.269955566		
EXOC6B		−1.275380087		
CTTN		−1.287911813		
VBP1		−1.361766656		
PICALM		−1.466374966		
CAPNS1		−1.756445493		
TOMM20		−1.819905488		
MAPK3		−1.918813356		
NUDC			1.732009523	
RAP2B			1.726355193	
KIF2A			1.663032474	
SETDB1			1.648556725	
ATP6V1D			1.640964877	
SYNGR1			1.58532711	
DPYSL3			1.582802831	
NCAN			1.571585688	
CTNND2			1.562506309	
CNRIP1			1.554254382	
UTP20			1.544855143	
ME1			1.478800627	
PFN1			1.477145399	
PPME1			1.455765369	
APP			1.455465641	
LANCL2			1.455132031	
GNAQ			1.453050806	
ARPC5L			1.451224208	
PRDX2			1.448929334	
CYFIP1			1.432347642	
HSPA2			1.431503207	
SFPQ			1.427372534	
TXLNG			1.42119969	
GRB2			1.4157442	
CPNE6			1.414165286	
PDXP			1.410098436	
GNB2			1.408516442	
GLOD4			1.391614359	
OXR1			1.380069445	
VAMP2			1.373709575	
HSPA1A			1.373028035	
HSPA1B			1.373028035	
PSD3			1.371411227	
BSG			1.369172074	
PFN2			1.368469072	
ATP5J2			1.368321641	
GNB1			1.366417736	
DLG4			1.363981591	
SH3GLB2			1.36363306	
KTN1			1.362629072	
RAB3A			1.362173026	
CCT7			1.3567651	
SDHC			1.347654394	
BCAN			1.342898167	
HSPH1			1.337373319	
ADD2			1.336930818	
AP2A2			1.335723865	
DYNLL2			1.322647505	
GDA			1.312825433	
TKT			1.3105561	
COX7A2			1.308575621	
SNCA			1.305570043	
BAIAP2			1.303596608	
SH3BGRL3			1.302583328	
NPM1			1.301978732	
NIPSNAP1			1.299090793	
ARPC4			1.296939524	
NAPA			1.292138971	
CALB1			1.290893833	
CAMKV			1.286413462	
COX6C			1.286388519	
COX5B			1.285714649	
NDUFA9			1.285358088	
ATP6V0D1			1.275603048	
NSFL1C			1.274526051	
LDHA			1.269135901	
ATP6V1F			1.266693513	
CLTB			1.265664013	
SRSF4			1.261593119	
LRGUK			1.260891479	
NDUFS8			1.260447912	
PPP2CB			1.259397109	
COX4I1			1.256849924	
CAMK2D			1.256848229	
DNAJC6			1.255985647	
COL6A2			1.253698992	
CRYM			1.251866833	
PPP3CA			1.249125255	
SLC2A1			1.24839376	
PPFIA3			1.244596343	
CAMK2A			1.241519397	
SYP			1.237859198	
AP2B1			1.237413188	
USP24			1.233820239	
FABP5			1.233314773	
SEPT9			1.226916013	
EHD1			1.226344591	
CAP2			1.223807885	
SLC25A3			1.221941931	
CAMK2B			1.22058499	
PTGES3			1.216665914	
PRDX1			1.215985447	
ATP5I			1.215521395	
ATIC			1.213849668	
GSTM5			1.213704207	
NDUFS7			1.211966939	
DCLK1			1.207992461	
GPD1L			1.207286149	
HNRNPL			1.205847041	
NAPB			1.202833155	
RTN4			1.201798491	
PCSK1N				−1.245474191
WDR7				−1.245967263
MAG				−1.248958157
DDB1				−1.255013637
MOBP				−1.256321905
GAD2				−1.26014124
INA				−1.265874043
TPT1				−1.268669401
IDH3G				−1.270175029
PCCB				−1.272883353
MAPRE2				−1.274095945
MRPS36				−1.275342647
MARCKS				−1.280485807
PSMC6				−1.281124693
ABAT				−1.283265073
NTM				−1.284285221
CA2				−1.284359839
HPCAL1				−1.284463003
MOG				−1.295178624
PSMA6				−1.297701354
SDHA				−1.300775393
COPB1				−1.300938943
NONO				−1.301095086
TUFM				−1.311237761
ADSS				−1.311542644
IGSF8				−1.312784192
COX5A				−1.313555144
UQCRFS1				−1.316410553
CKB				−1.318123142
PIP4K2B				−1.324477494
NEFM				−1.330302859
PSMD6				−1.330311752
NEFL				−1.344530231
NDUFA8				−1.348309239
BIN1				−1.354260702
PTMS				−1.379162453
PHB2				−1.381012946
NDUFS2				−1.382654847
DSTN				−1.396469687
CCT5				−1.396711507
S100B				−1.403153034
CLASP2				−1.403192685
EPB41L1				−1.419486383
PFKL				−1.424253294
PSAP				−1.429458675
CPLX1				−1.437904339
SERPINA3K				−1.462482626
AHSA1				−1.482509362
SET				−1.494218612
ATL1				−1.49652324
PRKACB				−1.501341659
NME2				−1.502355851
ITPKA				−1.515605888
NCS1				−1.519434163
EEF1A1				−1.528884517
PLCB1				−1.530645112
ENPP6				−1.542384655
SYNE1				−1.544411246
PCP4				−1.554348855
MBP				−1.583600552
NCEH1				−1.612308156
ACSL6				−1.612342183
GLO1				−1.659621805
PPP1R1B				−1.668571078
HSD17B10				−1.719780931
SIRT2				−1.776727737
GPHN				−1.810243901
MORC1				−1.853643692
SLC6A11				−1.877132951
PDHX				−1.878732243
PTK6				−1.89800334
DPYSL5				−1.903465278
HPRT1				−2.333095423

*For each specific protein studied fold change values, indicated in the table below, were generated from the iTRAQ expression ratios of (>1.2 or <0.8) proteins differentially regulated in the BTBR tissues [Cortex, Hippocampus (Hippo)] compared to WT*.

In addition to our global data set informatics interpretation we also sought to investigate the function of coherently-regulated groups of BTBR-associated proteins observed across multiple tissues. We therefore focused on the subset of proteins (12 upregulated; 4 downregulated: Table 7) coherently altered in a BTBR-specific manner in both the cortex and hippocampus (Figure 6B). To explore the potential functional connection between these select proteins we applied our novel informatic platform *Textrous!* (Chen et al., 2013). *Textrous!* allows the extraction of functional data, using latent semantic analysis (LSA), from even the smallest of datasets. *Textrous!* is a unique LSA tool as it possesses two forms of functional data output, i.e., collective and individual processing. Collective processing of the coherent BTBR protein set results in the creation of a hierarchical word-cloud revealing a strong and novel link between the BTBR ASD phenotype and N-WASP (neuronal Wiskott–Aldrich Syndrome protein) mediated activity (Figure 6C: Table 8). In addition there was a second, less strong demonstration of the importance of inherited genomic activity in ASD phenotypes. The individual processing mode in *Textrous!* also investigates the strongest individual protein-term relationships within a specific protein subset (Figure 6D). Again with this distinct analytical output we observed a strong relationship between cytoskeletal remodeling activity (f-actin, scaffolding, n-wasp, filopodia) and proteins such as Itsn1 (intersectin 1), Ap3b2 (adaptor-related protein complex 3, beta 2 subunit), Dbnl (drebrinlike) and Ermn (Ermin, ERM-like protein) with the BTBR phenotype. Interestingly other tight relationships between BTBR coherently-regulated factors were found, e.g., myelin-associated activity [Lancl1 (LanC bacterial lantibiotic synthetase component C)-like 1 and Ermn] and inherited neuronal atrophy and impairment [Prrt2 (proline-rich transmembrane protein **2**) and Cep290 (centrosomal protein 290)] (Figure 6D).

**Table 8:** ***Textrous!*-based collective analysis of coherently-regulated BTBR-specific proteins**.

**Word**	**Cosine similarity**	***Z*-score**	***p*-Value**
Motor	0.524476314	1.869598279	0.030741909
Nonsense	0.478414897	1.693752796	0.045132642
Atrophies	0.4700895	1.661969501	0.048256387
Gtpases	0.468206051	1.65477919	0.048962249
Exons	0.466743056	1.649194013	0.049573817
Growth-associated	0.455961506	1.608034038	0.053917589
Inherited	0.455845768	1.607592194	0.053917589
Gtpase	0.455708175	1.607066915	0.054027184
Autosomal	0.453900108	1.600164382	0.054799292
Polymerization	0.453672232	1.599294435	0.054910301
Optic	0.444664336	1.564905613	0.058791455
Exon	0.437675556	1.538225034	0.062024307
Gtp-binding	0.435679912	1.5306064	0.062884696
Maps	0.427384101	1.498936057	0.066936816
Bundles	0.426675363	1.496230358	0.067326828
Disabilities	0.423470554	1.483995579	0.06890446
Gdi	0.419726975	1.469703976	0.070780877
Brothers	0.419534775	1.46897023	0.070916395
Movement	0.417518799	1.461273977	0.072007721
Acuity	0.417184158	1.45999644	0.072145037
Cytoskeleton	0.414147875	1.448405032	0.073808525
n-wasp	0.412898356	1.443634832	0.074369488
Frameshift	0.411123648	1.436859653	0.075358997
Recessive	0.409375594	1.430186227	0.07635851
Wiskott-aldrich	0.407779814	1.424094126	0.077223236
Actin	0.406644694	1.419760659	0.077803841
Wasp	0.405802073	1.416543842	0.078241464
Gtpase-activating	0.402282931	1.403109057	0.080308419
Recycling	0.395770067	1.378245348	0.084101644
Reorganization	0.391991869	1.363821581	0.086283783
Prenylation	0.389968302	1.356096352	0.087549584
Splice	0.388453273	1.350312529	0.088507991
Missense	0.388195935	1.349330109	0.088668483
Streaming	0.387806369	1.347842888	0.088829191
Apparatus	0.3874916	1.346641219	0.088990116
Pericentriolar	0.387010855	1.344805911	0.089312617
Families	0.386042261	1.341108175	0.089960226
Plexiform	0.385574635	1.339322953	0.090285336
Polysomes	0.383798601	1.332542712	0.091265902
Guanosine	0.383107191	1.329903161	0.091759136
Splicing	0.382666221	1.328219703	0.092089053
Cytoskeletal	0.381993528	1.325651608	0.092419849
Mutational	0.381893517	1.325269803	0.092585576
Coiled-coil	0.381550118	1.323958833	0.092751522
gaps	0.380853905	1.321300948	0.093250682
Depolymerizing	0.380444571	1.319738261	0.093417509
Intellectual	0.378479462	1.312236202	0.094760067
Mutations	0.378323187	1.311639602	0.094760067
Protrusions	0.377199886	1.307351254	0.095606356
Neurofibromin	0.376699229	1.305439931	0.095946424
Actin-binding	0.375976678	1.302681498	0.096287381
Atrophy	0.375377983	1.3003959	0.096800485
Dynamics	0.373862886	1.29461182	0.097660115
Pigmentary	0.373853033	1.294574204	0.097660115
Neuron	0.370913941	1.283353835	0.099746038
Multidisciplinary	0.369651835	1.27853558	0.100448528
Destination	0.369566192	1.278208627	0.100624713
Cilia	0.368620901	1.274599856	0.101154621
Gtp-dependent	0.367048826	1.268598253	0.102220532
Phosphatidic	0.366734218	1.267397195	0.102577645
Identification	0.366608729	1.266918125	0.102577645
Disability	0.365764506	1.263695196	0.103115013
Muscular	0.365741802	1.26360852	0.103115013
Ciliary	0.364952202	1.260594116	0.103654424
Meshwork	0.364710238	1.259670389	0.103834681
Partners	0.364390606	1.258450149	0.104195878
Inheritance	0.364260066	1.257951798	0.104195878
Scanning	0.363734162	1.255944089	0.104557985
f-actin	0.363367304	1.254543564	0.10473938
Beta-galactoside	0.36316993	1.253790062	0.104921003
Anonymous	0.361144496	1.2460577	0.106382198
Protrusion	0.359889734	1.241267484	0.107302874
Sialyltransferase	0.359831442	1.241044947	0.107302874
Neurofibromatosis	0.359791096	1.24089092	0.107302874
Lipofuscin	0.359746522	1.240720753	0.107302874
Radixin	0.35878958	1.237067503	0.108043541
Proteins	0.358631641	1.236464548	0.108229282
Squid	0.358474059	1.23586296	0.108229282
Screen	0.356102317	1.226808525	0.109911295
Filopodia	0.355914644	1.226092057	0.110099338
Extensions	0.355265349	1.223613288	0.110476114
Lowe	0.355018906	1.222672462	0.110664848
Pre-mrna	0.354335273	1.220062601	0.111232437
Leber	0.354284348	1.219868188	0.111232437
Counseling	0.354132651	1.219289065	0.111422096
x-linked	0.354046801	1.218961323	0.111422096
Cones	0.353920096	1.218477608	0.111611986
Contig	0.353576868	1.217167292	0.111802108
Cryomicroscopy	0.35310173	1.215353389	0.112183046
Amaurosis	0.353046083	1.215140949	0.112183046
Methanothermobacter	0.352545212	1.21322881	0.11256491
Neurofibromas	0.35222224	1.211995824	0.11275619
Endocytic	0.352220725	1.211990038	0.11275619
Pumping	0.351073392	1.207609946	0.113523631
Tail	0.350586481	1.205751097	0.113908745
Dynamin	0.350367829	1.204916365	0.114101651
Syndrome	0.349521708	1.20168619	0.114681764
Endosomes	0.347991562	1.195844656	0.11584828
Blindness	0.34724489	1.19299414	0.116434687
Visual	0.347190286	1.192785679	0.116434687

*Cosine similarity scores, Z-scores and probability values (p-Value) were calculated using collective processing of the 16 coherently-regulated BTBR-specific proteins in the cortex and hippocampal datasets*.

## Discussion

In the present study we evaluated the behavior, synaptic protein alterations and targeted proteomic signatures in the hippocampus and cortex of 15 month old BTBR and WT mice to determine whether the ASD-like phenotype of BTBR mice was resistant to neuronal aging. Thus, we assessed both the behavioral phenotype and proteomic profiles between aged ASD-like mice (abnormal CNS) and aged wild type mice (with a relatively normal CNS). We found that several neurosynaptic proteins were significantly increased in both the hippocampus and cortex of the aged ASD-like mice in comparison to the aged wild type. In particular, the phosphorylated form of the pre-synaptic marker synapsin 1, and the post-synaptic marker spinophilin were increased in both the aged BTBR hippocampus and cortex; PSD95 was significantly elevated in aged BTBR hippocampus. Aged BTBR mice still presented a behavioral phenotype typically observed in young autistic mice, suggesting that the physiological actions underlying the ASD-like condition of the BTBR mice is relatively resistant to aging.

The maintenance of the ASD-phenotype into advanced age in BTBR mice is intriguing because in the normal developing CNS synapses are overproduced but then become pruned in response to neuronal activity during post-natal development (Petit, 1988; Cohen and Greenberg, 2008; Ebert and Greenberg, 2013). It is proposed that autism is caused by dysregulation of the machinery controlling synaptic messenger RNA translation leading to increased synthesis of synaptic proteins and lack of synaptic pruning, in turn inducing a hyperconnectivity and over-reinforcement of neuronal circuits. In fact it is one of the hallmark characteristics of ASD observed in the brains of both mouse models and humans with ASD (Kelleher and Bear, 2008; Gkogkas et al., 2013). Fragile X syndrome, for example which is the leading cause of inherited intellectual disability and ASD (Bhakar et al., 2012), is caused by mutations in FMR1 gene resulting in decreased expression of fragile X mental retardation protein (FMRP). This protein modulates the rate of translational elongation of mRNAs that encode synaptic proteins (Darnell et al., 2011). A decrease in FMRP impairs regulation of synaptic protein expression (Bhakar et al., 2012) and post-natal synapse elimination (Harlow et al., 2010).

As increased synaptic marker expression is associated with the autistic-like mouse phenotype it is not surprising that we found that the BTBR T + tf/j mice maintained their reduced social behavior into old age (Figure 1). This is consistent with the limited human literature that found that social impairments in individuals with ASD persist into adulthood (Ballaban-Gil et al., 1996; Szatmari, 1998; Howlin and Moss, 2012). Although increased synaptic markers have been found to be deleterious for the developing CNS, it is possible that the increased synapses and hyperconnected neuronal circuits be an effective advantage in the aging process where typically nervous system connectivity is degraded through accumulated damage. BDNF and TrkB mRNAs are localized in dendritic spines, thus age-related spine density decreases in older individuals suggest a link between BDNF and TrKB mRNA expression and impaired synaptic connections with aging (Tapia-Arancibia et al., 2008). Indeed decreases in both BDNF and TrkB expression have been documented in the hippocampus and in the neocortex during physiological aging (Phillips et al., 1991; Murray et al., 1994; Connor et al., 1997; Ferrer et al., 1999; Quartu et al., 1999; Hock et al., 2000; Holsinger et al., 2000; Fahnestock et al., 2002; Romanczyk et al., 2002; Tapia-Arancibia et al., 2008; Zhao et al., 2010).

We found that levels of mature BDNF were significantly decreased in the aged BTBR hippocampus and cortex compared with the aged WT (Figures 2A, 3A), suggesting an accelerated aging phenomena, but did not find a significant change in the overall level of active TrkB receptor (Figures 2D, 3D), in the BTBR mice. In addition, we also found that there was a non-significant trend for the increase in the expression in both cortical and hippocampal levels of Akt1, one of the most important downstream targets of TrkB signaling. Thus, via a potentially complex compensatory mechanism, the BTBR mice may have increased their BDNF signaling efficiency and downstream signal transduction capacity compared to WT mice.

Our findings of reduced BDNF in both the hippocampus and cortex in old BTBR mice compared to old WT mice despite seemingly intact memory functioning may be related to this efficiency potentiation of the BDNF-TrkB system in the BTBR mice. These effects may be related to changes in endocytic proteins such as Itsn1, Golga2 and Ap3b2 (Table 7) that may affect the signaling dynamics of the TrkB receptor (Mattson et al., 2004). In addition it is possible that the elevated levels of various synaptic markers served to protect the aging BTBR brain against age-related declines in cognition often associated with decreased levels of BDNF.

Given the complexity of the ASD, it is unlikely that BDNF signaling and various neuronal cell markers are the only protein products altered in the presence of ASD; thus we performed global quantitative iTRAQ proteomics, coupled to advanced bioinformatics in order to gain a comprehensive, systems-level understanding of potential changes in aged BTBR mice compared to WT counterparts. With respect to current relevant literature we discovered that many of these proteins differentially regulated in the brain of the old BTBR mice demonstrated strong correlations with those altered in humans with neurodevelopmental disabilities (Table S7). Nearly twenty percent of the coherently-regulated proteins across the hippocampus and cortex of aged BTBR mice also are strongly linked to human ASD phenotypes, suggesting that future investigation of the remaining factors may prove extremely fruitful for autism-related research (Figure 6B). With respect to the highly-focused, coherently-regulated protein set (Figure 6B: Table 7) Prrt2 was coherently altered in both the hippocampus and cortex of aged BTBR mice compared to aged WT (Table 7). Alterations in activity of Prrt2 have been found in the frontal cortex of individuals with autism (Ji et al., 2012) and have also been genetically-associated with ASD (Weber et al., 2013). Vat1l was up-regulated in both the hippocampus and cortex of the old BTBR mice.

We performed functional informatics data clustering to gain a wider appreciation of the signaling phenotype present in aged BTBR mice. Using GO term annotation it was evident in the cortex that considerable alterations in ER (endoplasmic reticulum), vesicular transport, cytoskeletal remodeling, and energy generation were apparent (Figures 4E,F). The functional GO term clustering of differentially expressed proteins in the BTBR hippocampus also indicated a role of energy generation, neurotransmitter release and synaptic activity but did not display a robust skeletal remodeling component (Table 2). We also employed KEGG and Ingenuity Canonical Signaling pathway analysis for the differentially-regulated cortex and hippocampal protein sets. At this higher level of functional interrogation (compared to GO term clustering) we also observed a subtle divergence in tissue behavior, i.e., cortical KEGG pathway analysis demonstrated a strong bias toward energy metabolic and neurotrophic/neuroprotective mechanisms (Figure 5A) while the hippocampus demonstrated a more degenerative phenotype associated with metabolic instabilities and calcium management factors (Figure 5B). Interestingly and to highlight the need for multidimensional informatics analyses for physiological interpretation, the canonical signaling pathway analysis of both tissue datasets revealed additional processes that could affect the ASD phenotype, i.e., the prominence of cytokine signaling activity in the cortex and the identification of the potential role of melatonin function in both CNS tissues (Figures 5C,D).

To further reinforce the importance of applying unbiased informatics for the analysis of complex data associated with a highly nuanced phenotype such as ASD we found that analysis of different subgroups of the data yielded interesting, tissue-specific insights into the effects of aging on ASD. Using our novel *Textrous!* platform we were able to identify differential tissue activity, i.e., enhanced glutamatergic cortical activity coincident with attenuated kinase signaling (Figure S1). These cortical changes occurred contemporaneously with an elevation of hippocampal protein misfolding/aggregation coincident with an attenuated CNS myelination and oligodendrocyte functionality (Figure S2). Both of these hippocampal processes have been recently associated with dysfunctional brain development (Mitew et al., 2013).

When approaching complex issues such as ASD with a systems-biology approach it is important to maintain this concept of multiple levels of protein interaction, function and hierarchical architecture, i.e., functions can be tissue specific, expression polarity specific while also existing contemporaneously at a level above tissue-or polarity-dependence. For example we found that inspection of the coherently-regulated multi-tissue protein set revealed an exciting and potentially novel functional association of neuronal Wasp (Wiskott-Aldrich syndrome protein) activity and ASD (Figures 6C,D). To our current knowledge this is the first example of such a functional correlation, however it is relatively simple to predict that the molecular activities of this skeletal remodeling factor could impact a disorder such as ASD in which a less synaptically plastic neurological system is present.

In conclusion, we found that both the social behavioral phenotype and the CNS proteomic profile of this ASD model are relatively age-resistant. Aged BTBR mice possessed elevations in hippocampal and cortical synaptic markers that likely play a role in the etiology of the autistic-like phenotype. Low levels of BDNF have been found inconsistently in the ASD literature but more consistently in the aging and Alzheimer Disease literature (Chadwick et al., 2010b). Diminished mature BDNF in the aged BTBR hippocampus/cortex were seen along with complementary alterations in the downstream signaling pathways compared to WT aged mice. These findings in-part reinforce the potential long-term neuroprotective capacity of the ASD phenotype with respect to cognitive aging. It is therefore interesting to posit that despite lower levels of neurosynaptic plasticity that the more robust and reinforced neuronal network in ASD phenotypes may indeed act prophylactically over long periods of time compared to normal CNS networks that degrade due to unrepaired damage and synaptic loss. The complex proteotype of the BTBR mice revealed molecular and functional signatures highly reminiscent of those found in humans with neurodevelopmental disabilities including ASD. Many of these identified processes play a role in neurodevelopment based on studies in murine models, and give more evidence for proteins that are playing a role in neuroprotection allowing for maintenance of cognition in the aged BTBR mice. Therefore, it is possible that the elevated levels of various synaptic markers and proteins identified proteomically such as Picalm, Itsn1, Mobp, Ina, as well as signaling actions involving Wasp functionality served to protect the aging BTBR brain against age-related declines in cognition often associated with decreased BDNF. In the current study we observed a robust maintenance of cognitive ability despite advanced age that may be due to unique neurosynaptic architecture and activity in the ASD phenotype. The role of the associated proteins and synaptic markers provide a foundation for further investigation into this intriguing mechanism.

### Conflict of interest statement

The authors declare that the research was conducted in the absence of any commercial or financial relationships that could be construed as a potential conflict of interest.

## Supplementary material

Figure S1 **Collective *Textrous!* analysis of differentially-regulated cortical protein subsets**. Functional hierarchical word-clouds were generated from the upregulated (BTBR:WT iTRAQ ratio > 1.2) (A) and downregulated (BTBR:WT iTRAQ ratio < 0.8) cortical protein data sets.

Figure S2 **Collective *Textrous!* analysis of differentially-regulated hippocampal protein subsets**. Functional hierarchical word-clouds were generated from the upregulated (BTBR:WT iTRAQ ratio >1.2) (A) and downregulated (BTBR:WT iTRAQ ratio < 0.8) hippocampal protein data sets.

## Abbreviations

BDNF: brain derived neurotrophic factor
BTBR: black and tan, brachyuric
ASD: autism spectrum disorder.
