# Supplementary figures and images for: The effects of aging on the BTBR mouse model of autism spectrum disorder
Source: Front Aging Neurosci. 2014 Sep 1;6:225. doi: 10.3389/fnagi.2014.00225 (PMC4150363; doi:10.3389/fnagi.2014.00225)

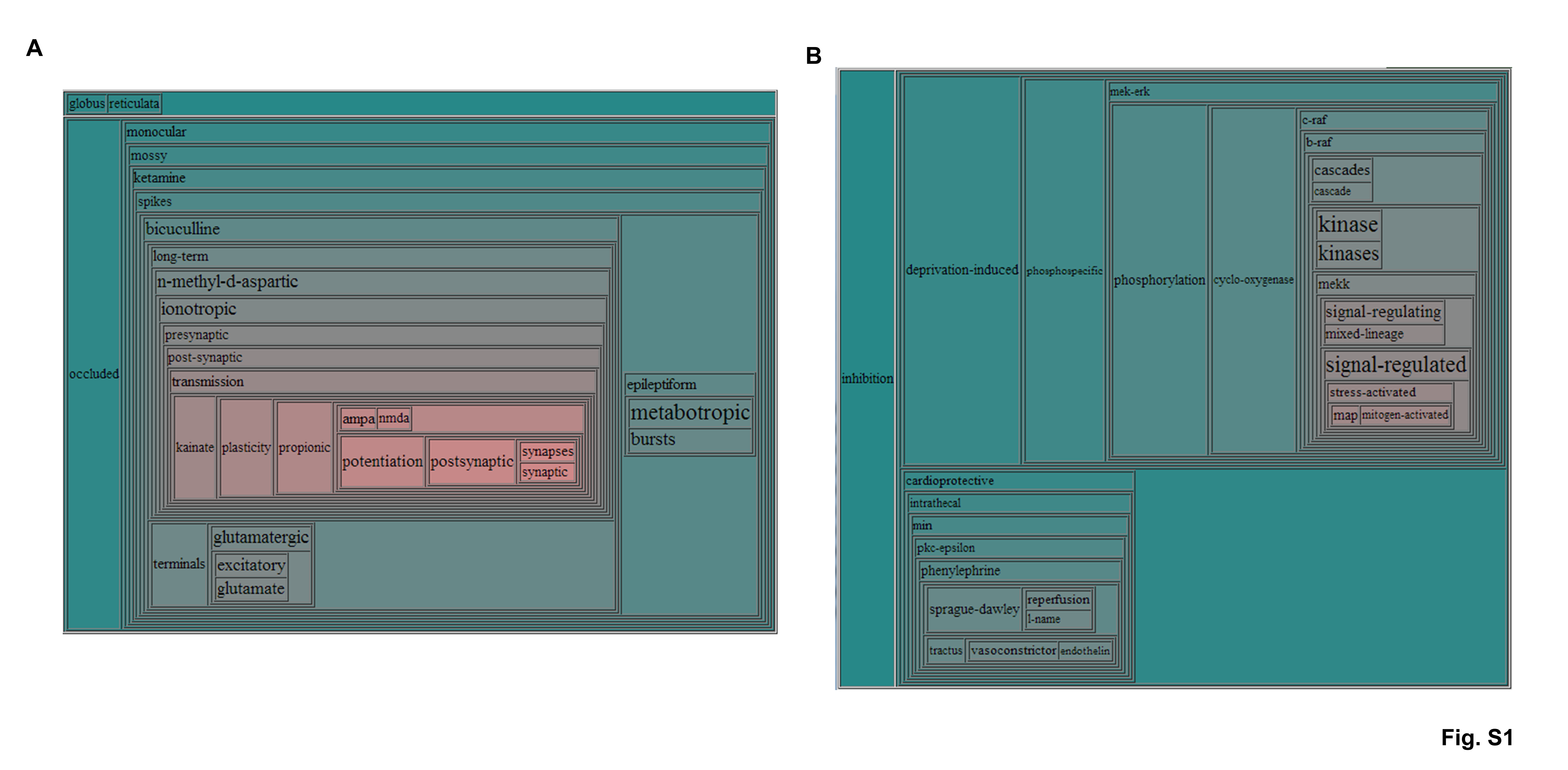

Supplement: Figure S1 — Collective Textrous! analysis of differentially-regulated cortical protein subsets. Functional hierarchical word-clouds were generated from the upregulated (BTBR:WT iTRAQ ratio > 1.2) (A) and downregulated (BTBR:WT iTRAQ ratio < 0.8) cortical protein data sets. [file DataSheet1.ZIP › Figure-S1.tif]

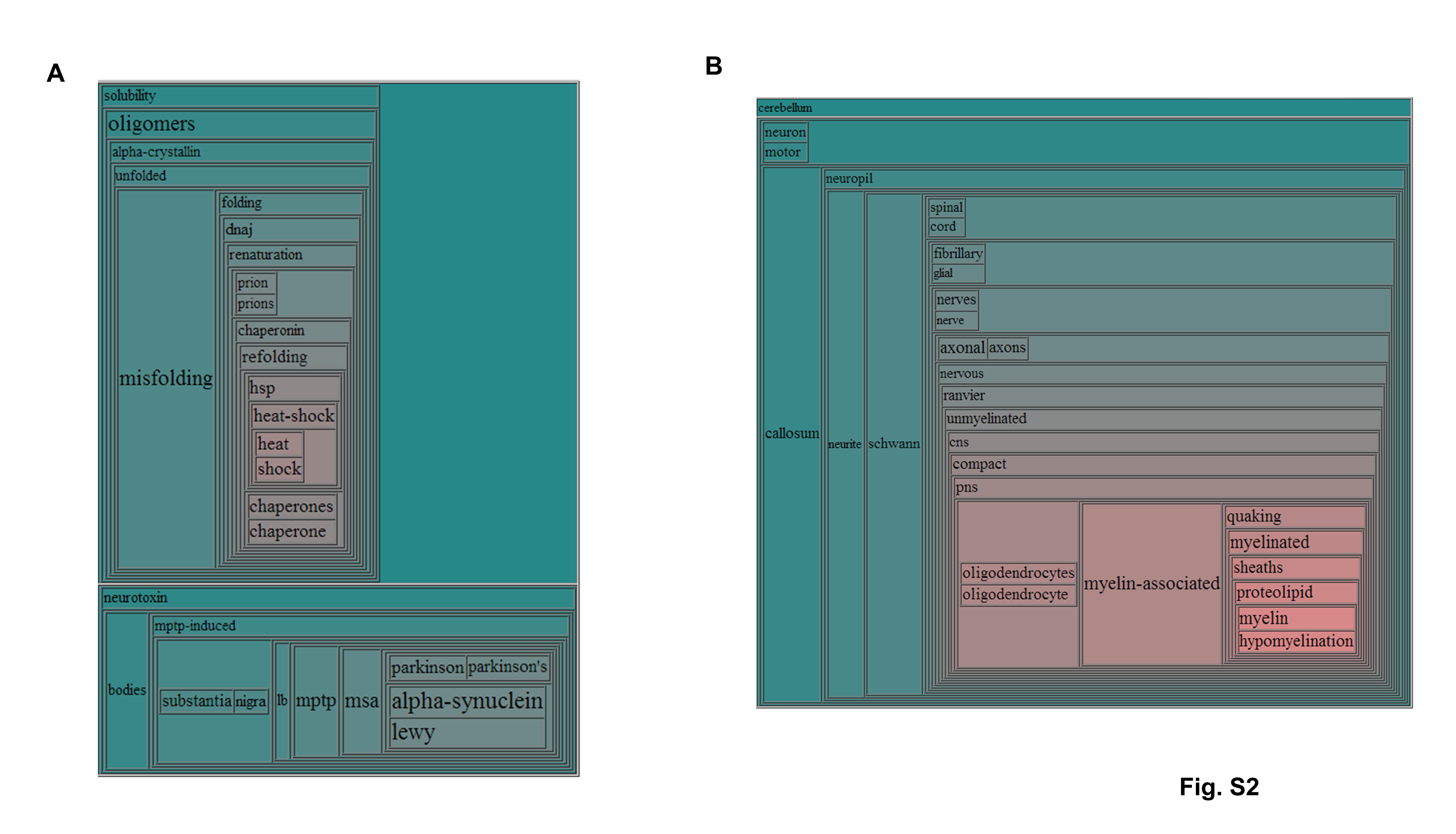

Supplement: Figure S2 — Collective Textrous! analysis of differentially-regulated hippocampal protein subsets. Functional hierarchical word-clouds were generated from the upregulated (BTBR:WT iTRAQ ratio >1.2) (A) and downregulated (BTBR:WT iTRAQ ratio < 0.8) hippocampal protein data sets. [file DataSheet2.ZIP › Figure-S2.tif]
