# Supplementary material for: The effects of aging on the BTBR mouse model of autism spectrum disorder
Source: Front Aging Neurosci. 2014 Sep 1;6:225. doi: 10.3389/fnagi.2014.00225 (PMC4150363; doi:10.3389/fnagi.2014.00225)
Supplement: Supplementary file 3 [file Table1.DOCX]

**Table S1. Identified and quantified proteins from BTBR cortex.** iTRAQ expression ratios (BTBR:WT), Fold Changes and Log_2_ transformed iTRAQ ratios were generated for proteins extracted from cortical BTBR tissues compared to WT controls.

| **Accession** | **Symbol** | **Σ# Peptides** | **Cortex BTBR:WT** | **Fold Change** | **Log2 ratio** | **Description** |
| --- | --- | --- | --- | --- | --- | --- |
| gi378522303 | PRRT2 | 2 | 1.951168338 | 1.951168338 | 0.964338252 | RecName: Full=Proline-rich transmembrane protein 2; AltName: Full=Dispanin subfamily B member 3 |
| gi29428127 | SLC32A1 | 2 | 1.828557315 | 1.828557315 | 0.870705847 | RecName: Full=Vesicular inhibitory amino acid transporter; AltName: Full=GABA and glycine transporter; AltName: Full=Solute carrier family 32 member 1; AltName: Full=Vesicular GABA transporter; Short=mVGAT; Short=mVIAAT |
| gi2500577 | PCBP2 | 3 | 1.792502849 | 1.792502849 | 0.841975412 | RecName: Full=Poly(rC)-binding protein 2; AltName: Full=Alpha-CP2; AltName: Full=CTBP; Short=CBP; AltName: Full=Putative heterogeneous nuclear ribonucleoprotein X; Short=hnRNP X |
| gi62510641 | QDPR | 2 | 1.7625157 | 1.7625157 | 0.817636109 | RecName: Full=Dihydropteridine reductase; AltName: Full=HDHPR; AltName: Full=Quinoid dihydropteridine reductase |
| gi52783579 | DYNLL1 | 2 | 1.672920918 | 1.672920918 | 0.742369248 | RecName: Full=Dynein light chain 1, cytoplasmic; AltName: Full=8 kDa dynein light chain; Short=DLC8; AltName: Full=Dynein light chain LC8-type 1; AltName: Full=Protein inhibitor of neuronal nitric oxide synthase; Short=PIN; Short=mPIN |
| gi51315842 | DBNL | 4 | 1.626693344 | 1.626693344 | 0.701942308 | RecName: Full=Drebrin-like protein; AltName: Full=Actin-binding protein 1; AltName: Full=SH3 domain-containing protein 7 |
| gi61219108 | AP3B2 | 2 | 1.581785753 | 1.581785753 | 0.661554205 | RecName: Full=AP-3 complex subunit beta-2; AltName: Full=Adapter-related protein complex 3 subunit beta-2; AltName: Full=Adaptor protein complex AP-3 subunit beta-2; AltName: Full=Beta-3B-adaptin; AltName: Full=Clathrin assembly protein complex 3 beta-2 large chain |
| gi20978758 | SRSF4 | 3 | 1.533013648 | 1.533013648 | 0.616370541 | RecName: Full=Serine/arginine-rich splicing factor 4; AltName: Full=Splicing factor, arginine/serine-rich 4 |
| gi54037410 | EIF5A | 2 | 1.517756095 | 1.517756095 | 0.601939967 | RecName: Full=Eukaryotic translation initiation factor 5A-1; Short=eIF-5A-1; Short=eIF-5A1; AltName: Full=Eukaryotic initiation factor 5A isoform 1; Short=eIF-5A; AltName: Full=eIF-4D |
| gi56748753 | ADCY2 | 2 | 1.511246891 | 1.511246891 | 0.595739371 | RecName: Full=Adenylate cyclase type 2; AltName: Full=ATP pyrophosphate-lyase 2; AltName: Full=Adenylate cyclase type II; AltName: Full=Adenylyl cyclase 2 |
| gi47117304 | NDUFS4 | 2 | 1.47998035 | 1.47998035 | 0.565578021 | RecName: Full=NADH dehydrogenase [ubiquinone] iron-sulfur protein 4, mitochondrial; AltName: Full=Complex I-18 kDa; Short=CI-18 kDa; AltName: Full=Complex I-AQDQ; Short=CI-AQDQ; AltName: Full=NADH-ubiquinone oxidoreductase 18 kDa subunit; Flags: Precursor |
| gi41688568 | SLC9A3R1 | 2 | 1.473396618 | 1.473396618 | 0.559145836 | RecName: Full=Na(+)/H(+) exchange regulatory cofactor NHE-RF1; Short=NHERF-1; AltName: Full=Ezrin-radixin-moesin-binding phosphoprotein 50; Short=EBP50; AltName: Full=Regulatory cofactor of Na(+)/H(+) exchanger; AltName: Full=Sodium-hydrogen exchanger regulatory factor 1; AltName: Full=Solute carrier family 9 isoform A3 regulatory factor 1 |
| gi62510940 | LETM1 | 2 | 1.455678341 | 1.455678341 | 0.541691601 | RecName: Full=LETM1 and EF-hand domain-containing protein 1, mitochondrial; AltName: Full=Leucine zipper-EF-hand-containing transmembrane protein 1; Flags: Precursor |
| gi126986 | PRDX3 | 2 | 1.44243043 | 1.44243043 | 0.528501738 | RecName: Full=Thioredoxin-dependent peroxide reductase, mitochondrial; AltName: Full=Antioxidant protein 1; Short=AOP-1; AltName: Full=PRX III; AltName: Full=Perioredoxin-3; AltName: Full=Protein MER5; Flags: Precursor |
| gi158518416 | IDH2 | 2 | 1.441010435 | 1.441010435 | 0.527080783 | RecName: Full=Isocitrate dehydrogenase [NADP], mitochondrial; Short=IDH; AltName: Full=ICD-M; AltName: Full=IDP; AltName: Full=NADP(+)-specific ICDH; AltName: Full=Oxalosuccinate decarboxylase; Flags: Precursor |
| gi341940668 | EXOC5 | 2 | 1.41342868 | 1.41342868 | 0.499199088 | RecName: Full=Exocyst complex component 5; AltName: Full=Exocyst complex component Sec10 |
| gi123778087 | HNRNPUL2 | 2 | 1.405691244 | 1.405691244 | 0.491279745 | RecName: Full=Heterogeneous nuclear ribonucleoprotein U-like protein 2; AltName: Full=MLF1-associated nuclear protein |
| gi52783066 | VAT1L | 2 | 1.391942674 | 1.391942674 | 0.477099796 | RecName: Full=Synaptic vesicle membrane protein VAT-1 homolog-like |
| gi38258077 | MMAA | 2 | 1.391228352 | 1.391228352 | 0.476359239 | RecName: Full=Methylmalonic aciduria type A homolog, mitochondrial; Flags: Precursor |
| WDR33_MOUSE | WDR33 | 3 | 1.379833997 | 1.379833997 | 0.464494711 | (Q8K4P0) pre-mRNA 3' end processing protein WDR33 |
| gi341941747 | PTCD1 | 2 | 1.371753507 | 1.371753507 | 0.456021264 | RecName: Full=Pentatricopeptide repeat-containing protein 1 |
| gi341940436 | DDX5 | 3 | 1.365841661 | 1.365841661 | 0.449790244 | RecName: Full=Probable ATP-dependent RNA helicase DDX5; AltName: Full=DEAD box RNA helicase DEAD1; Short=mDEAD1; AltName: Full=DEAD box protein 5; AltName: Full=RNA helicase p68 |
| gi81892818 | PPP1R9B | 2 | 1.346708939 | 1.346708939 | 0.429438079 | RecName: Full=Neurabin-2; AltName: Full=Neurabin-II; AltName: Full=Protein phosphatase 1 regulatory subunit 9B; AltName: Full=Spinophilin |
| gi22653715 | DPP6 | 2 | 1.340020376 | 1.340020376 | 0.422254938 | RecName: Full=Dipeptidyl aminopeptidase-like protein 6; AltName: Full=DPPX; AltName: Full=Dipeptidyl aminopeptidase-related protein; AltName: Full=Dipeptidyl peptidase 6; AltName: Full=Dipeptidyl peptidase IV-like protein; AltName: Full=Dipeptidyl peptidase VI; Short=DPP VI |
| gi124028616 | LAP3 | 2 | 1.303333413 | 1.303333413 | 0.382206195 | RecName: Full=Cytosol aminopeptidase; AltName: Full=Leucine aminopeptidase 3; Short=LAP-3; AltName: Full=Leucyl aminopeptidase; AltName: Full=Proline aminopeptidase; AltName: Full=Prolyl aminopeptidase |
| gi60392581 | KCNAB2 | 2 | 1.296085587 | 1.296085587 | 0.374160989 | RecName: Full=Voltage-gated potassium channel subunit beta-2; AltName: Full=K(+) channel subunit beta-2; AltName: Full=Kv-beta-2; AltName: Full=Neuroimmune protein F5 |
| gi294862512 | GOLGA2 (includes EG:2801) | 2 | 1.292500223 | 1.292500223 | 0.370164529 | RecName: Full=Golgin subfamily A member 2; AltName: Full=130 kDa cis-Golgi matrix protein; Short=GM130 |
| gi47115588 | LANCL1 | 2 | 1.279121599 | 1.279121599 | 0.35515342 | RecName: Full=LanC-like protein 1; AltName: Full=40 kDa erythrocyte membrane protein; Short=p40 |
| gi341940931 | ME3 | 2 | 1.270939623 | 1.270939623 | 0.345895495 | RecName: Full=NADP-dependent malic enzyme, mitochondrial; Short=NADP-ME; AltName: Full=Malic enzyme 3; Flags: Precursor |
| gi20138800 | ITSN1 | 3 | 1.269638936 | 1.269638936 | 0.344418277 | RecName: Full=Intersectin-1; AltName: Full=EH and SH3 domains protein 1 |
| gi182636954 | SLC12A5 | 7 | 1.252190507 | 1.252190507 | 0.324454069 | RecName: Full=Solute carrier family 12 member 5; AltName: Full=Electroneutral potassium-chloride cotransporter 2; AltName: Full=K-Cl cotransporter 2; Short=mKCC2; AltName: Full=Neuronal K-Cl cotransporter |
| gi41688584 | HOOK3 | 2 | 1.250023514 | 1.250023514 | 0.321955233 | RecName: Full=Protein Hook homolog 3; Short=mHK3 |
| gi84028249 | PTPN11 | 2 | 1.247745108 | 1.247745108 | 0.319323248 | RecName: Full=Tyrosine-protein phosphatase non-receptor type 11; AltName: Full=Protein-tyrosine phosphatase SYP; AltName: Full=SH-PTP2; Short=SHP-2; Short=Shp2 |
| gi166897986 | COX5A (includes EG:12858) | 4 | 1.247002966 | 1.247002966 | 0.318464897 | RecName: Full=Cytochrome c oxidase subunit 5A, mitochondrial; AltName: Full=Cytochrome c oxidase polypeptide Va; Flags: Precursor |
| gi12230747 | WDR1 | 2 | 1.244912709 | 1.244912709 | 0.316044587 | RecName: Full=WD repeat-containing protein 1; AltName: Full=Actin-interacting protein 1; Short=AIP1 |
| gi158705915 | GRM2 | 3 | 1.243617897 | 1.243617897 | 0.314543284 | RecName: Full=Metabotropic glutamate receptor 2; Short=mGluR2; Flags: Precursor |
| gi118595720 | CEP290 | 2 | 1.238621608 | 1.238621608 | 0.30873552 | RecName: Full=Centrosomal protein of 290 kDa; Short=Cep290; AltName: Full=Bardet-Biedl syndrome 14 protein homolog; AltName: Full=Nephrocystin-6 |
| gi341940297 | CAB39 | 2 | 1.2340405 | 1.2340405 | 0.303389743 | RecName: Full=Calcium-binding protein 39; AltName: Full=MO25alpha; AltName: Full=Protein Mo25 |
| gi134047901 | RTN3 | 2 | 1.233971862 | 1.233971862 | 0.303309498 | RecName: Full=Reticulon-3 |
| gi21363012 | TF | 2 | 1.233243832 | 1.233243832 | 0.302458072 | RecName: Full=Serotransferrin; Short=Transferrin; AltName: Full=Beta-1 metal-binding globulin; AltName: Full=Siderophilin; Flags: Precursor |
| gi51701397 | FBXO41 | 2 | 1.231290747 | 1.231290747 | 0.300171468 | RecName: Full=F-box only protein 41 |
| gi46397834 | RAB7A | 3 | 1.226744118 | 1.226744118 | 0.294834354 | RecName: Full=Ras-related protein Rab-7a |
| gi94730421 | RTN4 (includes EG:57142) | 2 | 1.216638369 | 1.216638369 | 0.282900409 | RecName: Full=Reticulon-4; AltName: Full=Neurite outgrowth inhibitor; Short=Nogo protein |
| gi1729865 | TCP1 | 3 | 1.215498024 | 1.215498024 | 0.281547548 | RecName: Full=T-complex protein 1 subunit alpha; Short=TCP-1-alpha; AltName: Full=CCT-alpha; AltName: Full=Tailless complex polypeptide 1A; Short=TCP-1-A; AltName: Full=Tailless complex polypeptide 1B; Short=TCP-1-B |
| gi54041237 | PRKAR2B | 2 | 1.215235782 | 1.215235782 | 0.281236255 | RecName: Full=cAMP-dependent protein kinase type II-beta regulatory subunit |
| gi150416330 | ZXDB | 2 | 1.214676409 | 1.214676409 | 0.28057203 | RecName: Full=Zinc finger X-linked protein ZXDA/ZXDB |
| gi81876946 | PCYOX1L | 2 | 1.209317219 | 1.209317219 | 0.274192731 | RecName: Full=Prenylcysteine oxidase-like; Flags: Precursor |
| gi549059 | CCT3 | 2 | 1.20748828 | 1.20748828 | 0.272009187 | RecName: Full=T-complex protein 1 subunit gamma; Short=TCP-1-gamma; AltName: Full=CCT-gamma; AltName: Full=Matricin; AltName: Full=mTRiC-P5 |
| gi342187017 | SLC17A7 | 4 | 1.204082729 | 1.204082729 | 0.267934518 | RecName: Full=Vesicular glutamate transporter 1; Short=VGluT1; AltName: Full=Brain-specific Na(+)-dependent inorganic phosphate cotransporter; AltName: Full=Solute carrier family 17 member 7 |
| gi251757307 | PRDM5 | 2 | 1.198502382 | 1.198502382 | 0.261232776 | RecName: Full=PR domain zinc finger protein 5; AltName: Full=PR domain-containing protein 5 |
| gi3122044 | DPYSL4 | 5 | 1.195193712 | 1.195193712 | 0.257244463 | RecName: Full=Dihydropyrimidinase-related protein 4; Short=DRP-4; AltName: Full=Collapsin response mediator protein 3; Short=CRMP-3; AltName: Full=UNC33-like phosphoprotein 4; Short=ULIP-4 |
| gi342187059 | TOMM70A | 2 | 1.187885389 | 1.187885389 | 0.248395647 | RecName: Full=Mitochondrial import receptor subunit TOM70; AltName: Full=Mitochondrial precursor proteins import receptor; AltName: Full=Translocase of outer membrane 70 kDa subunit |
| gi146291087 | TMOD2 | 6 | 1.18041645 | 1.18041645 | 0.239295932 | RecName: Full=Tropomodulin-2; AltName: Full=Neuronal tropomodulin; Short=N-Tmod |
| gi46576352 | CACNA2D1 | 2 | 1.177970865 | 1.177970865 | 0.236303857 | RecName: Full=Voltage-dependent calcium channel subunit alpha-2/delta-1; AltName: Full=Voltage-gated calcium channel subunit alpha-2/delta-1; Contains: RecName: Full=Voltage-dependent calcium channel subunit alpha-2-1; Contains: RecName: Full=Voltage-dependent calcium channel subunit delta-1; Flags: Precursor |
| gi152031595 | DMXL2 | 8 | 1.17103257 | 1.17103257 | 0.227781202 | RecName: Full=DmX-like protein 2; AltName: Full=Rabconnectin-3 |
| gi42559420 | PCBP1 | 2 | 1.170888347 | 1.170888347 | 0.227603511 | RecName: Full=Poly(rC)-binding protein 1; AltName: Full=Alpha-CP1; AltName: Full=Heterogeneous nuclear ribonucleoprotein E1; Short=hnRNP E1 |
| gi37077864 | AUH (includes EG:11992) | 2 | 1.170793243 | 1.170793243 | 0.227486325 | RecName: Full=Methylglutaconyl-CoA hydratase, mitochondrial; AltName: Full=AU-specific RNA-binding enoyl-CoA hydratase; Short=AU-binding enoyl-CoA hydratase; Short=muAUH; Flags: Precursor |
| gi399833 | SLC2A3 | 3 | 1.167283716 | 1.167283716 | 0.22315526 | RecName: Full=Solute carrier family 2, facilitated glucose transporter member 3; AltName: Full=Glucose transporter type 3, brain; Short=GLUT-3 |
| gi30315914 | AHSA1 | 2 | 1.164480484 | 1.164480484 | 0.21968646 | RecName: Full=Activator of 90 kDa heat shock protein ATPase homolog 1; Short=AHA1 |
| gi81910752 | GIT1 (includes EG:216963) | 2 | 1.163590666 | 1.163590666 | 0.218583628 | RecName: Full=ARF GTPase-activating protein GIT1; Short=ARF GAP GIT1; AltName: Full=G protein-coupled receptor kinase-interactor 1; AltName: Full=GRK-interacting protein 1 |
| gi46576640 | GMFB | 2 | 1.157439146 | 1.157439146 | 0.210936344 | RecName: Full=Glia maturation factor beta; Short=GMF-beta |
| gi1703188 | SLC25A6 | 12 | 1.153649437 | 1.153649437 | 0.206204895 | RecName: Full=ADP/ATP translocase 2; AltName: Full=ADP,ATP carrier protein 2; AltName: Full=Adenine nucleotide translocator 2; Short=ANT 2; AltName: Full=Solute carrier family 25 member 5 |
| gi81873719 | ENPP6 | 2 | 1.153015073 | 1.153015073 | 0.205411373 | RecName: Full=Ectonucleotide pyrophosphatase/phosphodiesterase family member 6; Short=E-NPP 6; Short=NPP-6; Contains: RecName: Full=Ectonucleotide pyrophosphatase/phosphodiesterase family member 6 soluble form; Flags: Precursor |
| gi8134322 | ATP8A1 | 3 | 1.153012805 | 1.153012805 | 0.205408535 | RecName: Full=Probable phospholipid-transporting ATPase IA; AltName: Full=ATPase class I type 8A member 1; AltName: Full=Chromaffin granule ATPase II |
| gi51704193 | VAMP2 | 3 | 1.152275324 | 1.152275324 | 0.204485474 | RecName: Full=Vesicle-associated membrane protein 2; Short=VAMP-2; AltName: Full=Synaptobrevin-2 |
| gi1351942 | ANXA5 | 3 | 1.149909124 | 1.149909124 | 0.201519851 | RecName: Full=Annexin A5; AltName: Full=Anchorin CII; AltName: Full=Annexin V; AltName: Full=Annexin-5; AltName: Full=Calphobindin I; Short=CBP-I; AltName: Full=Endonexin II; AltName: Full=Lipocortin V; AltName: Full=Placental anticoagulant protein 4; Short=PP4; AltName: Full=Placental anticoagulant protein I; Short=PAP-I; AltName: Full=Thromboplastin inhibitor; AltName: Full=Vascular anticoagulant-alpha; Short=VAC-alpha |
| gi1345696 | CAPZA2 | 4 | 1.147311862 | 1.147311862 | 0.198257598 | RecName: Full=F-actin-capping protein subunit alpha-2; AltName: Full=CapZ alpha-2 |
| gi47606029 | PIP4K2B | 2 | 1.143499126 | 1.143499126 | 0.193455263 | RecName: Full=Phosphatidylinositol-5-phosphate 4-kinase type-2 beta; AltName: Full=1-phosphatidylinositol-5-phosphate 4-kinase 2-beta; AltName: Full=Diphosphoinositide kinase 2-beta; AltName: Full=Phosphatidylinositol-5-phosphate 4-kinase type II beta; Short=PI(5)P 4-kinase type II beta; Short=PIP4KII-beta; AltName: Full=PtdIns(5)P-4-kinase isoform 2-beta |
| gi24638218 | SH3BGRL3 | 3 | 1.14203221 | 1.14203221 | 0.191603341 | RecName: Full=SH3 domain-binding glutamic acid-rich-like protein 3 |
| gi50402237 | PPP2CB | 5 | 1.140882938 | 1.140882938 | 0.19015077 | RecName: Full=Serine/threonine-protein phosphatase 2A catalytic subunit beta isoform; Short=PP2A-beta |
| gi24418394 | ALDH1L1 | 2 | 1.138605594 | 1.138605594 | 0.187268093 | RecName: Full=Cytosolic 10-formyltetrahydrofolate dehydrogenase; Short=10-FTHFDH; Short=FDH; AltName: Full=Aldehyde dehydrogenase family 1 member L1 |
| gi341941123 | SLC2A1 | 2 | 1.136439701 | 1.136439701 | 0.184521138 | RecName: Full=Solute carrier family 2, facilitated glucose transporter member 1; AltName: Full=Glucose transporter type 1, erythrocyte/brain; Short=GLUT-1; Short=GT1 |
| gi44888293 | PDXP | 3 | 1.136312878 | 1.136312878 | 0.184360128 | RecName: Full=Pyridoxal phosphate phosphatase; Short=PLP phosphatase; AltName: Full=Chronophin |
| gi78099814 | ATP6V1H | 7 | 1.135095946 | 1.135095946 | 0.18281425 | RecName: Full=V-type proton ATPase subunit H; Short=V-ATPase subunit H; AltName: Full=Vacuolar proton pump subunit H |
| gi32469711 | GCC2 | 2 | 1.134621127 | 1.134621127 | 0.182210632 | RecName: Full=GRIP and coiled-coil domain-containing protein 2; AltName: Full=185 kDa Golgi coiled-coil protein; Short=GCC185 |
| gi14916635 | PYGM | 5 | 1.13393413 | 1.13393413 | 0.181336837 | RecName: Full=Glycogen phosphorylase, muscle form; AltName: Full=Myophosphorylase |
| gi231557 | APOA1 | 2 | 1.133181903 | 1.133181903 | 0.180379467 | RecName: Full=Apolipoprotein A-I; Short=Apo-AI; Short=ApoA-I; AltName: Full=Apolipoprotein A1; Flags: Precursor |
| gi76364091 | BAG6 | 2 | 1.132916099 | 1.132916099 | 0.180041022 | RecName: Full=Large proline-rich protein BAG6; AltName: Full=BAG family molecular chaperone regulator 6; AltName: Full=BCL2-associated athanogene 6; Short=BAG-6; Short=BAG6; AltName: Full=HLA-B-associated transcript 3; AltName: Full=Protein Scythe |
| gi81862370 | CYFIP2 | 6 | 1.130940535 | 1.130940535 | 0.177523074 | RecName: Full=Cytoplasmic FMR1-interacting protein 2; AltName: Full=p53-inducible protein 121 |
| gi9910790 | TAGLN3 | 2 | 1.12937064 | 1.12937064 | 0.175519032 | RecName: Full=Transgelin-3; AltName: Full=Neuronal protein NP25 |
| gi18203578 | EHD1 | 2 | 1.128953991 | 1.128953991 | 0.174986692 | RecName: Full=EH domain-containing protein 1; AltName: Full=PAST homolog 1; Short=mPAST1 |
| gi117949769 | PCSK1N | 4 | 1.125108398 | 1.125108398 | 0.170064004 | RecName: Full=ProSAAS; AltName: Full=IA-4; AltName: Full=Proprotein convertase subtilisin/kexin type 1 inhibitor; Short=Proprotein convertase 1 inhibitor; AltName: Full=pro-SAAS; Contains: RecName: Full=KEP; Contains: RecName: Full=Big SAAS; Short=b-SAAS; Contains: RecName: Full=Little SAAS; Short=l-SAAS; Contains: RecName: Full=Big PEN-LEN; Short=b-PEN-LEN; AltName: Full=SAAS CT(1-49); Contains: RecName: Full=PEN; Contains: RecName: Full=PEN-20; Contains: RecName: Full=PEN-19; Contains: RecName: Full=Little LEN; Short=l-LEN; Contains: RecName: Full=Big LEN; Short=b-LEN; AltName: Full=SAAS CT(25-40); Flags: Precursor |
| gi3183025 | HSD17B10 | 2 | 1.124088743 | 1.124088743 | 0.168755936 | RecName: Full=3-hydroxyacyl-CoA dehydrogenase type-2; AltName: Full=17-beta-hydroxysteroid dehydrogenase 10; Short=17-beta-HSD 10; AltName: Full=3-hydroxy-2-methylbutyryl-CoA dehydrogenase; AltName: Full=3-hydroxyacyl-CoA dehydrogenase type II; AltName: Full=Endoplasmic reticulum-associated amyloid beta-peptide-binding protein; AltName: Full=Mitochondrial ribonuclease P protein 2; Short=Mitochondrial RNase P protein 2; AltName: Full=Type II HADH |
| gi223634791 | ANK2 | 7 | 1.121053856 | 1.121053856 | 0.164855588 | RecName: Full=Ankyrin-2; Short=ANK-2; AltName: Full=Brain ankyrin |
| gi205830867 | NFU1 | 2 | 1.120541545 | 1.120541545 | 0.164196139 | RecName: Full=NFU1 iron-sulfur cluster scaffold homolog, mitochondrial; AltName: Full=HIRA-interacting protein 5; Short=mHIRIP5; Flags: Precursor |
| gi62901088 | RAB35 | 2 | 1.120263019 | 1.120263019 | 0.163837493 | RecName: Full=Ras-related protein Rab-35 |
| gi20141319 | GNAZ | 2 | 1.118892333 | 1.118892333 | 0.162071218 | RecName: Full=Guanine nucleotide-binding protein G(z) subunit alpha; AltName: Full=G(x) alpha chain; AltName: Full=Gz-alpha |
| gi461586 | ATP5I | 3 | 1.116124631 | 1.116124631 | 0.158498134 | RecName: Full=ATP synthase subunit e, mitochondrial; Short=ATPase subunit e |
| gi54037163 | GANAB | 2 | 1.115907481 | 1.115907481 | 0.15821742 | RecName: Full=Neutral alpha-glucosidase AB; AltName: Full=Alpha-glucosidase 2; AltName: Full=Glucosidase II subunit alpha; Flags: Precursor |
| gi59798430 | PTMS | 2 | 1.114938397 | 1.114938397 | 0.156964 | RecName: Full=Parathymosin |
| gi49036421 | NCS1 | 2 | 1.113980473 | 1.113980473 | 0.155723944 | RecName: Full=Neuronal calcium sensor 1; Short=NCS-1; AltName: Full=Frequenin homolog |
| gi29427667 | SIRPA | 4 | 1.113924972 | 1.113924972 | 0.155652064 | RecName: Full=Tyrosine-protein phosphatase non-receptor type substrate 1; Short=SHP substrate 1; Short=SHPS-1; AltName: Full=Brain Ig-like molecule with tyrosine-based activation motifs; Short=Bit; AltName: Full=CD172 antigen-like family member A; AltName: Full=Inhibitory receptor SHPS-1; AltName: Full=MyD-1 antigen; AltName: Full=Signal-regulatory protein alpha-1; Short=Sirp-alpha-1; Short=mSIRP-alpha1; AltName: Full=p84; AltName: CD_antigen=CD172a; Flags: Precursor |
| gi117502 | CALR | 6 | 1.110444852 | 1.110444852 | 0.151137746 | RecName: Full=Calreticulin; AltName: Full=CRP55; AltName: Full=Calregulin; AltName: Full=Endoplasmic reticulum resident protein 60; Short=ERp60; AltName: Full=HACBP; Flags: Precursor |
| gi22095581 | DCTN2 | 3 | 1.110042003 | 1.110042003 | 0.150614268 | RecName: Full=Dynactin subunit 2; AltName: Full=50 kDa dynein-associated polypeptide; AltName: Full=Dynactin complex 50 kDa subunit; Short=DCTN-50; AltName: Full=Growth cone membrane protein 23-48K; Short=GMP23-48K; AltName: Full=p50 dynamitin |
| gi47117242 | NDUFS8 | 4 | 1.109951125 | 1.109951125 | 0.150496151 | RecName: Full=NADH dehydrogenase [ubiquinone] iron-sulfur protein 8, mitochondrial; AltName: Full=Complex I-23kD; Short=CI-23kD; AltName: Full=NADH-ubiquinone oxidoreductase 23 kDa subunit; Flags: Precursor |
| gi118105 | PPIA | 11 | 1.109092681 | 1.109092681 | 0.149379929 | RecName: Full=Peptidyl-prolyl cis-trans isomerase A; Short=PPIase A; AltName: Full=Cyclophilin A; AltName: Full=Cyclosporin A-binding protein; AltName: Full=Rotamase A; AltName: Full=SP18 |
| gi59798463 | LIN7A | 3 | 1.108554258 | 1.108554258 | 0.148679384 | RecName: Full=Protein lin-7 homolog A; Short=Lin-7A; Short=mLin-7; AltName: Full=Mammalian lin-seven protein 1; Short=MALS-1; AltName: Full=Vertebrate lin-7 homolog 1; Short=Veli-1 |
| gi160419228 | PSD3 | 2 | 1.10739085 | 1.10739085 | 0.147164506 | RecName: Full=PH and SEC7 domain-containing protein 3; AltName: Full=Exchange factor for ADP-ribosylation factor guanine nucleotide factor 6; AltName: Full=Pleckstrin homology and SEC7 domain-containing protein 3 |
| gi59799776 | PRKACB | 4 | 1.10626258 | 1.10626258 | 0.145693861 | RecName: Full=cAMP-dependent protein kinase catalytic subunit beta; Short=PKA C-beta |
| gi47117296 | NDUFC2 | 4 | 1.10592158 | 1.10592158 | 0.145249089 | RecName: Full=NADH dehydrogenase [ubiquinone] 1 subunit C2; AltName: Full=Complex I-B14.5b; Short=CI-B14.5b; AltName: Full=NADH-ubiquinone oxidoreductase subunit B14.5b |
| gi81916660 | CADM3 | 2 | 1.10449497 | 1.10449497 | 0.143386849 | RecName: Full=Cell adhesion molecule 3; AltName: Full=Immunoglobulin superfamily member 4B; Short=IgSF4B; AltName: Full=Nectin-like protein 1; Short=NECL-1; AltName: Full=Synaptic cell adhesion molecule 3; AltName: Full=TSLC1-like protein 1; Flags: Precursor |
| gi32130449 | RPL12 | 2 | 1.103967724 | 1.103967724 | 0.142697994 | RecName: Full=60S ribosomal protein L12 |
| gi90110719 | IGSF8 | 2 | 1.10353763 | 1.10353763 | 0.142135825 | RecName: Full=Immunoglobulin superfamily member 8; Short=IgSF8; AltName: Full=CD81 partner 3; AltName: Full=Glu-Trp-Ile EWI motif-containing protein 2; Short=EWI-2; AltName: Full=Keratinocyte-associated transmembrane protein 4; Short=KCT-4; AltName: Full=Prostaglandin regulatory-like protein; AltName: CD_antigen=CD316; Flags: Precursor |
| gi81881319 | RUFY3 | 3 | 1.10139475 | 1.10139475 | 0.139331637 | RecName: Full=Protein RUFY3; AltName: Full=Rap2-interacting protein x; Short=RIPx; AltName: Full=Single axon-regulated protein; Short=Singar |
| gi2506246 | SPTB | 6 | 1.100120549 | 1.100120549 | 0.13766162 | RecName: Full=Spectrin beta chain, erythrocyte; AltName: Full=Beta-I spectrin |
| gi17380314 | NAPG | 6 | 1.099841932 | 1.099841932 | 0.137296196 | RecName: Full=Gamma-soluble NSF attachment protein; Short=SNAP-gamma; AltName: Full=N-ethylmaleimide-sensitive factor attachment protein gamma |
| gi160409932 | SH3GLB2 | 3 | 1.099255387 | 1.099255387 | 0.136526603 | RecName: Full=Endophilin-B2; AltName: Full=SH3 domain-containing GRB2-like protein B2 |
| gi342187144 | ATP6V0A1 | 10 | 1.098959732 | 1.098959732 | 0.136138524 | RecName: Full=V-type proton ATPase 116 kDa subunit a isoform 1; Short=V-ATPase 116 kDa isoform a1; AltName: Full=Clathrin-coated vesicle/synaptic vesicle proton pump 116 kDa subunit; AltName: Full=Vacuolar adenosine triphosphatase subunit Ac116; AltName: Full=Vacuolar proton pump subunit 1; AltName: Full=Vacuolar proton translocating ATPase 116 kDa subunit a isoform 1 |
| gi56757667 | HSPA1A | 9 | 1.098781437 | 1.098781437 | 0.135904442 | RecName: Full=Heat shock 70 kDa protein 1A; AltName: Full=Heat shock 70 kDa protein 3; Short=HSP70.3; AltName: Full=Hsp68 |
| gi2851596 | TALDO1 | 4 | 1.09851893 | 1.09851893 | 0.135559731 | RecName: Full=Transaldolase |
| gi215273994 | HNRNPL | 3 | 1.098065922 | 1.098065922 | 0.134964669 | RecName: Full=Heterogeneous nuclear ribonucleoprotein L; Short=hnRNP L |
| gi81862978 | KTN1 | 2 | 1.097751294 | 1.097751294 | 0.134551235 | RecName: Full=Kinectin |
| gi54038837 | PHB | 6 | 1.097671832 | 1.097671832 | 0.134446801 | RecName: Full=Prohibitin; AltName: Full=B-cell receptor-associated protein 32; Short=BAP 32 |
| gi2500582 | NPM1 | 3 | 1.096903347 | 1.096903347 | 0.133436409 | RecName: Full=Nucleophosmin; Short=NPM; AltName: Full=Nucleolar phosphoprotein B23; AltName: Full=Nucleolar protein NO38; AltName: Full=Numatrin |
| gi1350769 | RPL7 | 2 | 1.096431705 | 1.096431705 | 0.132815952 | RecName: Full=60S ribosomal protein L7 |
| gi2499469 | PRDX2 | 5 | 1.096275658 | 1.096275658 | 0.132610609 | RecName: Full=Peroxiredoxin-2; AltName: Full=Thiol-specific antioxidant protein; Short=TSA; AltName: Full=Thioredoxin peroxidase 1; AltName: Full=Thioredoxin-dependent peroxide reductase 1 |
| gi20141656 | STX1A | 7 | 1.091747278 | 1.091747278 | 0.126638933 | RecName: Full=Syntaxin-1A; AltName: Full=Neuron-specific antigen HPC-1 |
| gi76363295 | PHB2 | 3 | 1.090671831 | 1.090671831 | 0.125217078 | RecName: Full=Prohibitin-2; AltName: Full=B-cell receptor-associated protein BAP37; AltName: Full=Repressor of estrogen receptor activity |
| gi68566057 | SFPQ | 5 | 1.08884891 | 1.08884891 | 0.122803778 | RecName: Full=Splicing factor, proline- and glutamine-rich; AltName: Full=DNA-binding p52/p100 complex, 100 kDa subunit; AltName: Full=Polypyrimidine tract-binding protein-associated-splicing factor; Short=PSF; Short=PTB-associated-splicing factor |
| gi21263432 | ATP5C1 | 3 | 1.086941393 | 1.086941393 | 0.120274154 | RecName: Full=ATP synthase subunit gamma, mitochondrial; AltName: Full=F-ATPase gamma subunit; Flags: Precursor |
| gi61229841 | PDXK | 3 | 1.086938028 | 1.086938028 | 0.120269687 | RecName: Full=Pyridoxal kinase; AltName: Full=Pyridoxine kinase |
| gi94730394 | CAMK2B | 15 | 1.086777935 | 1.086777935 | 0.12005718 | RecName: Full=Calcium/calmodulin-dependent protein kinase type II subunit beta; Short=CaM kinase II subunit beta; Short=CaMK-II subunit beta |
| gi9978489 | HAPLN1 | 2 | 1.085529698 | 1.085529698 | 0.118399195 | RecName: Full=Hyaluronan and proteoglycan link protein 1; AltName: Full=Cartilage-linking protein 1; Short=Cartilage-link protein; AltName: Full=Proteoglycan link protein; Flags: Precursor |
| gi9910829 | PSMA6 | 2 | 1.085054434 | 1.085054434 | 0.11776742 | RecName: Full=Proteasome subunit alpha type-6; AltName: Full=Macropain iota chain; AltName: Full=Multicatalytic endopeptidase complex iota chain; AltName: Full=Proteasome iota chain |
| gi62510597 | UTP20 | 2 | 1.084481095 | 1.084481095 | 0.117004904 | RecName: Full=Small subunit processome component 20 homolog; AltName: Full=Down-regulated in metastasis protein |
| gi341941160 | NDUFS1 | 9 | 1.084443618 | 1.084443618 | 0.116955047 | RecName: Full=NADH-ubiquinone oxidoreductase 75 kDa subunit, mitochondrial; AltName: Full=Complex I-75kD; Short=CI-75kD; Flags: Precursor |
| gi13626886 | GDI2 | 8 | 1.080969263 | 1.080969263 | 0.112325502 | RecName: Full=Rab GDP dissociation inhibitor beta; Short=Rab GDI beta; AltName: Full=GDI-3; AltName: Full=Guanosine diphosphate dissociation inhibitor 2; Short=GDI-2 |
| gi17380315 | NAPA (includes EG:108124) | 3 | 1.080413652 | 1.080413652 | 0.111583775 | RecName: Full=Alpha-soluble NSF attachment protein; Short=SNAP-alpha; AltName: Full=N-ethylmaleimide-sensitive factor attachment protein alpha |
| gi14916536 | BIN1 | 9 | 1.079999965 | 1.079999965 | 0.111031265 | RecName: Full=Myc box-dependent-interacting protein 1; AltName: Full=Amphiphysin II; AltName: Full=Amphiphysin-like protein; AltName: Full=Bridging integrator 1; AltName: Full=SH3 domain-containing protein 9 |
| gi3914438 | PSMA3 | 2 | 1.078409366 | 1.078409366 | 0.108904932 | RecName: Full=Proteasome subunit alpha type-3; AltName: Full=Macropain subunit C8; AltName: Full=Multicatalytic endopeptidase complex subunit C8; AltName: Full=Proteasome component C8; AltName: Full=Proteasome subunit K |
| gi1352217 | GAD2 | 3 | 1.077795511 | 1.077795511 | 0.108083484 | RecName: Full=Glutamate decarboxylase 2; AltName: Full=65 kDa glutamic acid decarboxylase; Short=GAD-65; AltName: Full=Glutamate decarboxylase 65 kDa isoform |
| gi47116926 | LANCL2 | 2 | 1.077658401 | 1.077658401 | 0.107899941 | RecName: Full=LanC-like protein 2; AltName: Full=Testis-specific adriamycin sensitivity protein |
| gi38257686 | GDAP1L1 | 2 | 1.076851677 | 1.076851677 | 0.10681955 | RecName: Full=Ganglioside-induced differentiation-associated protein 1-like 1; Short=GDAP1-L1 |
| gi25453322 | VPS35 | 2 | 1.076480193 | 1.076480193 | 0.106321775 | RecName: Full=Vacuolar protein sorting-associated protein 35; AltName: Full=Maternal-embryonic 3; AltName: Full=Vesicle protein sorting 35 |
| gi51316976 | AP2S1 | 4 | 1.075665947 | 1.075665947 | 0.105230112 | RecName: Full=AP-2 complex subunit sigma; AltName: Full=Adapter-related protein complex 2 sigma subunit; AltName: Full=Adaptor protein complex AP-2 subunit sigma; AltName: Full=Clathrin assembly protein 2 small chain; AltName: Full=Clathrin coat assembly protein AP17; AltName: Full=Clathrin coat-associated protein AP17; AltName: Full=Plasma membrane adaptor AP-2 17 kDa protein; AltName: Full=Sigma-adaptin 3b; AltName: Full=Sigma2-adaptin |
| gi266414 | CKMT1A/CKMT1B | 14 | 1.075452686 | 1.075452686 | 0.104944055 | RecName: Full=Creatine kinase U-type, mitochondrial; AltName: Full=Acidic-type mitochondrial creatine kinase; Short=Mia-CK; AltName: Full=Ubiquitous mitochondrial creatine kinase; Short=U-MtCK; Flags: Precursor |
| gi6093768 | VDAC2 | 4 | 1.07540828 | 1.07540828 | 0.104884485 | RecName: Full=Voltage-dependent anion-selective channel protein 2; Short=VDAC-2; Short=mVDAC2; AltName: Full=Outer mitochondrial membrane protein porin 2; AltName: Full=Voltage-dependent anion-selective channel protein 6; Short=VDAC-6; Short=mVDAC6 |
| gi81881275 | MOBP | 2 | 1.075311685 | 1.075311685 | 0.104754894 | RecName: Full=Myelin-associated oligodendrocyte basic protein |
| gi109818808 | DNAJC6 | 3 | 1.075081261 | 1.075081261 | 0.104445712 | RecName: Full=Putative tyrosine-protein phosphatase auxilin; AltName: Full=DnaJ homolog subfamily C member 6 |
| gi1352004 | ATP1B2 | 2 | 1.073882205 | 1.073882205 | 0.102835751 | RecName: Full=Sodium/potassium-transporting ATPase subunit beta-2; AltName: Full=AMOG; AltName: Full=Glial cell adhesion molecule; AltName: Full=Sodium/potassium-dependent ATPase subunit beta-2 |
| gi18203410 | UCHL1 | 8 | 1.073532354 | 1.073532354 | 0.102365672 | RecName: Full=Ubiquitin carboxyl-terminal hydrolase isozyme L1; Short=UCH-L1; AltName: Full=Neuron cytoplasmic protein 9.5; AltName: Full=PGP 9.5; Short=PGP9.5; AltName: Full=Ubiquitin thioesterase L1 |
| gi81913084 | NECAB1 | 2 | 1.073517574 | 1.073517574 | 0.102345808 | RecName: Full=N-terminal EF-hand calcium-binding protein 1; Short=EF-hand calcium-binding protein 1 |
| gi76364169 | DDX17 | 3 | 1.073391994 | 1.073391994 | 0.102177033 | RecName: Full=Probable ATP-dependent RNA helicase DDX17; AltName: Full=DEAD box protein 17 |
| gi325530087 | KIF7 | 2 | 1.073049911 | 1.073049911 | 0.101717182 | RecName: Full=Kinesin-like protein KIF7 |
| gi47117649 | ACTR2 | 3 | 1.072434792 | 1.072434792 | 0.10088993 | RecName: Full=Actin-related protein 2; AltName: Full=Actin-like protein 2 |
| gi47117306 | RPS19 | 2 | 1.07209088 | 1.07209088 | 0.100427206 | RecName: Full=40S ribosomal protein S19 |
| gi121716 | GSTM5 | 13 | 1.071992777 | 1.071992777 | 0.100295185 | RecName: Full=Glutathione S-transferase Mu 1; AltName: Full=GST 1-1; AltName: Full=GST class-mu 1; AltName: Full=Glutathione S-transferase GT8.7; AltName: Full=pmGT10 |
| gi342187361 | WDR7 | 2 | 1.070382605 | 1.070382605 | 0.098126576 | RecName: Full=WD repeat-containing protein 7; AltName: Full=TGF-beta resistance-associated protein TRAG |
| gi341940231 | AP2A2 | 12 | 1.07004513 | 1.07004513 | 0.097671644 | RecName: Full=AP-2 complex subunit alpha-2; AltName: Full=100 kDa coated vesicle protein C; AltName: Full=Adapter-related protein complex 2 alpha-2 subunit; AltName: Full=Adaptor protein complex AP-2 subunit alpha-2; AltName: Full=Alpha-adaptin C; AltName: Full=Alpha2-adaptin; AltName: Full=Clathrin assembly protein complex 2 alpha-C large chain; AltName: Full=Plasma membrane adaptor HA2/AP2 adaptin alpha C subunit |
| gi55584163 | PPP3CB | 7 | 1.069809257 | 1.069809257 | 0.097353593 | RecName: Full=Serine/threonine-protein phosphatase 2B catalytic subunit beta isoform; AltName: Full=CAM-PRP catalytic subunit; AltName: Full=Calmodulin-dependent calcineurin A subunit beta isoform |
| gi57015413 | NCKIPSD | 2 | 1.069627427 | 1.069627427 | 0.097108364 | RecName: Full=NCK-interacting protein with SH3 domain; AltName: Full=54 kDa VacA-interacting protein; Short=VIP54; AltName: Full=90 kDa N-WASP-interacting protein; AltName: Full=90 kDa SH3 protein interacting with Nck; AltName: Full=SH3 adapter protein SPIN90; AltName: Full=WASP-interacting SH3-domain protein; Short=WISH; AltName: Full=Wiskott-Aldrich syndrome protein-binding protein; Short=N-WASP-binding protein |
| gi341940423 | DYNC1I1 | 4 | 1.069177875 | 1.069177875 | 0.096501888 | RecName: Full=Cytoplasmic dynein 1 intermediate chain 1; AltName: Full=Cytoplasmic dynein intermediate chain 1; AltName: Full=Dynein intermediate chain 1, cytosolic; Short=DH IC-1 |
| gi13626388 | EEF1G | 2 | 1.068940062 | 1.068940062 | 0.09618096 | RecName: Full=Elongation factor 1-gamma; Short=EF-1-gamma; AltName: Full=eEF-1B gamma |
| gi13124070 | COPB1 | 2 | 1.068702986 | 1.068702986 | 0.095860955 | RecName: Full=Coatomer subunit beta; AltName: Full=Beta-coat protein; Short=Beta-COP |
| gi10719959 | CPNE6 | 3 | 1.068347306 | 1.068347306 | 0.095380724 | RecName: Full=Copine-6; AltName: Full=Copine VI; AltName: Full=Neuronal-copine; Short=N-copine |
| gi21362536 | DPYSL5 | 2 | 1.06670146 | 1.06670146 | 0.093156463 | RecName: Full=Dihydropyrimidinase-related protein 5; Short=DRP-5; AltName: Full=Collapsin response mediator protein 5; Short=CRMP-5 |
| gi52783085 | PPA2 (includes EG:27068) | 3 | 1.065635648 | 1.065635648 | 0.09171425 | RecName: Full=Inorganic pyrophosphatase 2, mitochondrial; AltName: Full=Pyrophosphate phospho-hydrolase 2; Short=PPase 2; Flags: Precursor |
| gi47606041 | PHYHIP | 4 | 1.065308011 | 1.065308011 | 0.091270615 | RecName: Full=Phytanoyl-CoA hydroxylase-interacting protein; AltName: Full=Phytanoyl-CoA hydroxylase-associated protein 1; Short=PAHX-AP1; Short=PAHXAP1 |
| gi51338697 | VSNL1 | 8 | 1.064910204 | 1.064910204 | 0.090731784 | RecName: Full=Visinin-like protein 1; Short=VILIP; AltName: Full=Neural visinin-like protein 1; Short=NVL-1; Short=NVP-1 |
| gi399310 | CTNNB1 | 6 | 1.06295566 | 1.06295566 | 0.088081417 | RecName: Full=Catenin beta-1; AltName: Full=Beta-catenin |
| gi57012721 | CS | 12 | 1.062669903 | 1.062669903 | 0.087693522 | RecName: Full=Citrate synthase, mitochondrial; Flags: Precursor |
| gi341942254 | PGM1 | 5 | 1.060557808 | 1.060557808 | 0.084823261 | RecName: Full=Phosphoglucomutase-1; Short=PGM 1; AltName: Full=Glucose phosphomutase 1 |
| gi341941131 | PFKL | 11 | 1.060142781 | 1.060142781 | 0.084258581 | RecName: Full=6-phosphofructokinase, liver type; AltName: Full=Phosphofructo-1-kinase isozyme B; Short=PFK-B; AltName: Full=Phosphofructokinase 1; AltName: Full=Phosphohexokinase |
| gi67460489 | CAND1 | 6 | 1.059200503 | 1.059200503 | 0.082975713 | RecName: Full=Cullin-associated NEDD8-dissociated protein 1; AltName: Full=Cullin-associated and neddylation-dissociated protein 1; AltName: Full=p120 CAND1 |
| gi83305642 | SEPT5 | 8 | 1.059165585 | 1.059165585 | 0.082928151 | RecName: Full=Septin-5; AltName: Full=Cell division control-related protein 1; Short=CDCrel-1; AltName: Full=Peanut-like protein 1 |
| gi341941065 | HSP90AB1 | 23 | 1.058635667 | 1.058635667 | 0.082206166 | RecName: Full=Heat shock protein HSP 90-beta; AltName: Full=Heat shock 84 kDa; Short=HSP 84; Short=HSP84; AltName: Full=Tumor-specific transplantation 84 kDa antigen; Short=TSTA |
| gi292630943 | SYNE2 | 2 | 1.058278919 | 1.058278919 | 0.081719912 | RecName: Full=Nesprin-2; AltName: Full=Nuclear envelope spectrin repeat protein 2; AltName: Full=Nucleus and actin connecting element protein; Short=Protein NUANCE; AltName: Full=Synaptic nuclear envelope protein 2; Short=Syne-2 |
| gi38372295 | NFASC | 7 | 1.058218144 | 1.058218144 | 0.081637059 | RecName: Full=Neurofascin; Flags: Precursor |
| gi3023203 | 2010107E04Rik | 2 | 1.057759218 | 1.057759218 | 0.081011258 | RecName: Full=6.8 kDa mitochondrial proteolipid |
| gi46395721 | ACTN1 | 14 | 1.057509381 | 1.057509381 | 0.080670461 | RecName: Full=Alpha-actinin-1; AltName: Full=Alpha-actinin cytoskeletal isoform; AltName: Full=F-actin cross-linking protein; AltName: Full=Non-muscle alpha-actinin-1 |
| gi341940229 | AP1B1 | 10 | 1.057174629 | 1.057174629 | 0.080213707 | RecName: Full=AP-1 complex subunit beta-1; AltName: Full=Adapter-related protein complex 1 subunit beta-1; AltName: Full=Adaptor protein complex AP-1 subunit beta-1; AltName: Full=Beta-1-adaptin; AltName: Full=Beta-adaptin 1; AltName: Full=Clathrin assembly protein complex 1 beta large chain; AltName: Full=Golgi adaptor HA1/AP1 adaptin beta subunit |
| gi131884 | NRAS | 2 | 1.056139832 | 1.056139832 | 0.078800859 | RecName: Full=GTPase NRas; AltName: Full=Transforming protein N-Ras; Flags: Precursor |
| gi543921 | CANX | 10 | 1.056036601 | 1.056036601 | 0.078659837 | RecName: Full=Calnexin; Flags: Precursor |
| gi34222626 | SLC25A22 | 4 | 1.056035102 | 1.056035102 | 0.078657789 | RecName: Full=Mitochondrial glutamate carrier 1; Short=GC-1; AltName: Full=Glutamate/H(+) symporter 1; AltName: Full=Solute carrier family 25 member 22 |
| gi3041732 | SOD2 | 6 | 1.055637426 | 1.055637426 | 0.078114405 | RecName: Full=Superoxide dismutase [Mn], mitochondrial; Flags: Precursor |
| gi391359280 | GLSK | 4 | 1.055472256 | 1.055472256 | 0.077888656 | RecName: Full=Glutaminase kidney isoform, mitochondrial; Short=GLS; Flags: Precursor |
| gi13629369 | PFKP | 13 | 1.055216977 | 1.055216977 | 0.07753968 | RecName: Full=6-phosphofructokinase type C; AltName: Full=Phosphofructo-1-kinase isozyme C; Short=PFK-C; AltName: Full=Phosphofructokinase 1; AltName: Full=Phosphohexokinase |
| gi20137942 | C14orf166 | 2 | 1.054394777 | 1.054394777 | 0.076415129 | RecName: Full=UPF0568 protein C14orf166 homolog |
| gi45476974 | DDAH1 | 2 | 1.054077927 | 1.054077927 | 0.075981528 | RecName: Full=N(G),N(G)-dimethylarginine dimethylaminohydrolase 1; Short=DDAH-1; Short=Dimethylarginine dimethylaminohydrolase 1; AltName: Full=DDAHI; AltName: Full=Dimethylargininase-1 |
| gi81910611 | ACTR3B | 3 | 1.05395681 | 1.05395681 | 0.075815749 | RecName: Full=Actin-related protein 3B; AltName: Full=ARP3-beta; AltName: Full=Actin-like protein 3B |
| gi83305135 | NDUFS6 | 3 | 1.053764966 | 1.053764966 | 0.075553121 | RecName: Full=NADH dehydrogenase [ubiquinone] iron-sulfur protein 6, mitochondrial; AltName: Full=Complex I-13kD-A; Short=CI-13kD-A; AltName: Full=NADH-ubiquinone oxidoreductase 13 kDa-A subunit; Flags: Precursor |
| gi46396175 | NRGN | 4 | 1.053583579 | 1.053583579 | 0.075304766 | RecName: Full=Neurogranin; Short=Ng; AltName: Full=RC3; Contains: RecName: Full=NEUG(55-78) |
| gi158518420 | TNIK | 3 | 1.052609143 | 1.052609143 | 0.073969831 | RecName: Full=Traf2 and NCK-interacting protein kinase |
| gi134034087 | DDB1 | 2 | 1.051670478 | 1.051670478 | 0.072682733 | RecName: Full=DNA damage-binding protein 1; AltName: Full=DDB p127 subunit; AltName: Full=Damage-specific DNA-binding protein 1; AltName: Full=UV-damaged DNA-binding factor |
| gi6093647 | PAK1 | 6 | 1.051354125 | 1.051354125 | 0.07224869 | RecName: Full=Serine/threonine-protein kinase PAK 1; AltName: Full=Alpha-PAK; AltName: Full=CDC42/RAC effector kinase PAK-A; AltName: Full=p21-activated kinase 1; Short=PAK-1; AltName: Full=p65-PAK |
| gi29427692 | IMMT | 9 | 1.050751899 | 1.050751899 | 0.071422064 | RecName: Full=Mitochondrial inner membrane protein; AltName: Full=Mitofilin |
| gi341942263 | PRPS1 | 3 | 1.049344641 | 1.049344641 | 0.069488587 | RecName: Full=Ribose-phosphate pyrophosphokinase 1; AltName: Full=Phosphoribosyl pyrophosphate synthase I; Short=PRS-I |
| gi46577639 | RAB10 | 3 | 1.04905618 | 1.04905618 | 0.069091941 | RecName: Full=Ras-related protein Rab-10 |
| gi125306 | CKM | 3 | 1.048887277 | 1.048887277 | 0.068859641 | RecName: Full=Creatine kinase M-type; AltName: Full=Creatine kinase M chain; AltName: Full=M-CK |
| gi55977306 | PSMC6 | 2 | 1.048737147 | 1.048737147 | 0.06865313 | RecName: Full=26S protease regulatory subunit 10B; AltName: Full=26S proteasome AAA-ATPase subunit RPT4; AltName: Full=Proteasome 26S subunit ATPase 6; AltName: Full=Proteasome subunit p42 |
| gi128101 | GAP43 | 5 | 1.048585372 | 1.048585372 | 0.068444325 | RecName: Full=Neuromodulin; AltName: Full=Axonal membrane protein GAP-43; AltName: Full=Calmodulin-binding protein P-57; AltName: Full=Growth-associated protein 43 |
| gi22654291 | CCT2 | 4 | 1.048324205 | 1.048324205 | 0.068084954 | RecName: Full=T-complex protein 1 subunit beta; Short=TCP-1-beta; AltName: Full=CCT-beta |
| gi81871239 | HIST1H2AC | 5 | 1.047047022 | 1.047047022 | 0.066326234 | RecName: Full=Histone H2A type 1-H |
| gi32363403 | NDUFB10 | 3 | 1.046825313 | 1.046825313 | 0.066020716 | RecName: Full=NADH dehydrogenase [ubiquinone] 1 beta subcomplex subunit 10; AltName: Full=Complex I-PDSW; Short=CI-PDSW; AltName: Full=NADH-ubiquinone oxidoreductase PDSW subunit |
| gi22256950 | PACSIN1 | 17 | 1.046266999 | 1.046266999 | 0.065251063 | RecName: Full=Protein kinase C and casein kinase substrate in neurons protein 1 |
| gi47117840 | NAPB | 11 | 1.045778936 | 1.045778936 | 0.064577917 | RecName: Full=Beta-soluble NSF attachment protein; Short=SNAP-beta; AltName: Full=Brain protein I47; AltName: Full=N-ethylmaleimide-sensitive factor attachment protein beta |
| gi114393 | ATP1B1 | 10 | 1.045420555 | 1.045420555 | 0.064083431 | RecName: Full=Sodium/potassium-transporting ATPase subunit beta-1; AltName: Full=Sodium/potassium-dependent ATPase subunit beta-1 |
| gi2498425 | GRB2 | 3 | 1.044700645 | 1.044700645 | 0.063089603 | RecName: Full=Growth factor receptor-bound protein 2; AltName: Full=Adapter protein GRB2; AltName: Full=SH2/SH3 adapter GRB2 |
| gi2829840 | ATP5J | 3 | 1.044444781 | 1.044444781 | 0.06273622 | RecName: Full=ATP synthase-coupling factor 6, mitochondrial; Short=ATPase subunit F6; Flags: Precursor |
| gi342179357 | SHANK1 | 2 | 1.044441426 | 1.044441426 | 0.062731587 | RecName: Full=SH3 and multiple ankyrin repeat domains protein 1; Short=Shank1 |
| gi81916755 | GLOD4 | 3 | 1.044415078 | 1.044415078 | 0.06269519 | RecName: Full=Glyoxalase domain-containing protein 4 |
| gi341940868 | KIF21B | 3 | 1.044333161 | 1.044333161 | 0.062582031 | RecName: Full=Kinesin-like protein KIF21B; AltName: Full=Kinesin-like protein KIF6 |
| gi81894342 | KIF27 | 2 | 1.044333161 | 1.044333161 | 0.062582031 | RecName: Full=Kinesin-like protein KIF27 |
| gi32172431 | DNM1 | 36 | 1.042745405 | 1.042745405 | 0.060386955 | RecName: Full=Dynamin-1 |
| gi3334247 | C1QBP | 2 | 1.042061853 | 1.042061853 | 0.059440913 | RecName: Full=Complement component 1 Q subcomponent-binding protein, mitochondrial; AltName: Full=GC1q-R protein; AltName: Full=Glycoprotein gC1qBP; Short=C1qBP; Flags: Precursor |
| gi78103425 | WASF1 | 3 | 1.041263839 | 1.041263839 | 0.05833567 | RecName: Full=Wiskott-Aldrich syndrome protein family member 1; Short=WASP family protein member 1; AltName: Full=Protein WAVE-1 |
| gi51317303 | GNB1 | 10 | 1.04086097 | 1.04086097 | 0.057777378 | RecName: Full=Guanine nucleotide-binding protein G(I)/G(S)/G(T) subunit beta-1; AltName: Full=Transducin beta chain 1 |
| gi61216668 | RTN1 (includes EG:104001) | 6 | 1.040630669 | 1.040630669 | 0.057458132 | RecName: Full=Reticulon-1; AltName: Full=Neuroendocrine-specific protein |
| gi51702253 | CPLX1 | 5 | 1.039804005 | 1.039804005 | 0.056311617 | RecName: Full=Complexin-1; AltName: Full=921-S; AltName: Full=Complexin I; Short=CPX I; AltName: Full=Synaphin-2 |
| gi122066700 | VAPA | 2 | 1.039566437 | 1.039566437 | 0.055981961 | RecName: Full=Vesicle-associated membrane protein-associated protein A; Short=VAMP-A; Short=VAMP-associated protein A; Short=VAP-A; AltName: Full=33 kDa VAMP-associated protein; Short=VAP-33 |
| gi2498751 | PEA15 | 3 | 1.038821051 | 1.038821051 | 0.054947155 | RecName: Full=Astrocytic phosphoprotein PEA-15; AltName: Full=15 kDa phosphoprotein enriched in astrocytes |
| gi41018346 | SYNJ1 | 7 | 1.038341617 | 1.038341617 | 0.054281172 | RecName: Full=Synaptojanin-1; AltName: Full=Synaptic inositol-1,4,5-trisphosphate 5-phosphatase 1 |
| gi8928560 | COX7A2 | 2 | 1.037818153 | 1.037818153 | 0.053553676 | RecName: Full=Cytochrome c oxidase subunit 7A2, mitochondrial; AltName: Full=Cytochrome c oxidase subunit VIIa-liver/heart; Short=Cytochrome c oxidase subunit VIIa-L; Flags: Precursor |
| gi117104 | Cox5b | 4 | 1.037566839 | 1.037566839 | 0.053204277 | RecName: Full=Cytochrome c oxidase subunit 5B, mitochondrial; AltName: Full=Cytochrome c oxidase polypeptide Vb; Flags: Precursor |
| gi18202309 | OPA1 | 8 | 1.03756102 | 1.03756102 | 0.053196185 | RecName: Full=Dynamin-like 120 kDa protein, mitochondrial; AltName: Full=Large GTP-binding protein; Short=LargeG; AltName: Full=Optic atrophy protein 1 homolog; Contains: RecName: Full=Dynamin-like 120 kDa protein, form S1; Flags: Precursor |
| gi20140777 | UQCRQ | 3 | 1.037248699 | 1.037248699 | 0.052761848 | RecName: Full=Cytochrome b-c1 complex subunit 8; AltName: Full=Complex III subunit 8; AltName: Full=Complex III subunit VIII; AltName: Full=Ubiquinol-cytochrome c reductase complex 9.5 kDa protein; AltName: Full=Ubiquinol-cytochrome c reductase complex ubiquinone-binding protein QP-C |
| gi341940634 | BSN | 13 | 1.035983173 | 1.035983173 | 0.05100057 | RecName: Full=Protein bassoon |
| gi47116573 | ACTR3 | 5 | 1.035562598 | 1.035562598 | 0.050414765 | RecName: Full=Actin-related protein 3; AltName: Full=Actin-like protein 3 |
| gi20455479 | ATP5B | 23 | 1.035421542 | 1.035421542 | 0.050218239 | RecName: Full=ATP synthase subunit beta, mitochondrial; Flags: Precursor |
| gi56404944 | PARK7 | 5 | 1.035359089 | 1.035359089 | 0.050131218 | RecName: Full=Protein DJ-1; AltName: Full=Parkinson disease protein 7 homolog; Flags: Precursor |
| gi341940470 | DNAH5 | 3 | 1.035193377 | 1.035193377 | 0.049900292 | RecName: Full=Dynein heavy chain 5, axonemal; AltName: Full=Axonemal beta dynein heavy chain 5; Short=mDNAH5; AltName: Full=Ciliary dynein heavy chain 5 |
| gi81881914 | SV2A | 6 | 1.035184566 | 1.035184566 | 0.049888013 | RecName: Full=Synaptic vesicle glycoprotein 2A; Short=Synaptic vesicle protein 2; Short=Synaptic vesicle protein 2A; AltName: Full=Calcium regulator SV2A |
| gi30316201 | HNRNPA3 | 2 | 1.034907306 | 1.034907306 | 0.049501555 | RecName: Full=Heterogeneous nuclear ribonucleoprotein A3; Short=hnRNP A3 |
| gi22653628 | AKR1A1 | 2 | 1.034838442 | 1.034838442 | 0.049405554 | RecName: Full=Alcohol dehydrogenase [NADP+]; AltName: Full=Aldehyde reductase; AltName: Full=Aldo-keto reductase family 1 member A1 |
| gi68565610 | IDH3A | 10 | 1.034023929 | 1.034023929 | 0.048269573 | RecName: Full=Isocitrate dehydrogenase [NAD] subunit alpha, mitochondrial; AltName: Full=Isocitric dehydrogenase subunit alpha; AltName: Full=NAD(+)-specific ICDH subunit alpha; Flags: Precursor |
| gi1170384 | HSP90AA1 | 25 | 1.033789952 | 1.033789952 | 0.047943085 | RecName: Full=Heat shock protein HSP 90-alpha; AltName: Full=Heat shock 86 kDa; Short=HSP 86; Short=HSP86; AltName: Full=Tumor-specific transplantation 86 kDa antigen; Short=TSTA |
| gi81911483 | CAMK2D | 10 | 1.033755319 | 1.033755319 | 0.047894753 | RecName: Full=Calcium/calmodulin-dependent protein kinase type II subunit delta; Short=CaM kinase II subunit delta; Short=CaMK-II subunit delta |
| gi108935875 | PPME1 | 2 | 1.033430403 | 1.033430403 | 0.047441232 | RecName: Full=Protein phosphatase methylesterase 1; Short=PME-1 |
| gi313104120 | ITPR1 | 2 | 1.033397578 | 1.033397578 | 0.047395407 | RecName: Full=Inositol 1,4,5-trisphosphate receptor type 1; AltName: Full=IP3 receptor isoform 1; Short=IP3R 1; Short=InsP3R1; AltName: Full=Inositol 1,4,5-trisphosphate-binding protein P400; AltName: Full=Protein PCD-6; AltName: Full=Purkinje cell protein 1; AltName: Full=Type 1 inositol 1,4,5-trisphosphate receptor; Short=Type 1 InsP3 receptor |
| gi31077176 | HSPA4L | 4 | 1.03288083 | 1.03288083 | 0.046673811 | RecName: Full=Heat shock 70 kDa protein 4L; AltName: Full=Heat shock 70-related protein APG-1; AltName: Full=Osmotic stress protein 94 |
| gi51316996 | CPLX2 | 6 | 1.032771296 | 1.032771296 | 0.046520809 | RecName: Full=Complexin-2; AltName: Full=921-L; AltName: Full=Complexin II; Short=CPX II; AltName: Full=Synaphin-1 |
| gi54036156 | PRKAR1A | 2 | 1.031829011 | 1.031829011 | 0.045203915 | RecName: Full=cAMP-dependent protein kinase type I-alpha regulatory subunit |
| gi146345423 | GFAP | 8 | 1.03158994 | 1.03158994 | 0.044869609 | RecName: Full=Glial fibrillary acidic protein; Short=GFAP |
| gi20137987 | DCLK1 | 3 | 1.031458255 | 1.031458255 | 0.044685434 | RecName: Full=Serine/threonine-protein kinase DCLK1; AltName: Full=Doublecortin-like and CAM kinase-like 1; AltName: Full=Doublecortin-like kinase 1 |
| gi117029 | COX2 (includes EG:140540) | 3 | 1.030907696 | 1.030907696 | 0.043915164 | RecName: Full=Cytochrome c oxidase subunit 2; AltName: Full=Cytochrome c oxidase polypeptide II |
| gi126752 | MARCKS | 2 | 1.029833285 | 1.029833285 | 0.042410805 | RecName: Full=Myristoylated alanine-rich C-kinase substrate; Short=MARCKS |
| gi67460546 | CPNE4 | 2 | 1.029739108 | 1.029739108 | 0.042278866 | RecName: Full=Copine-4; AltName: Full=Copine IV |
| gi47117166 | NDUFA12 | 4 | 1.029683959 | 1.029683959 | 0.042201599 | RecName: Full=NADH dehydrogenase [ubiquinone] 1 alpha subcomplex subunit 12; AltName: Full=Complex I-B17.2; Short=CI-B17.2; Short=CIB17.2; AltName: Full=NADH-ubiquinone oxidoreductase subunit B17.2 |
| gi52788305 | SUCLA2 | 6 | 1.029632425 | 1.029632425 | 0.042129392 | RecName: Full=Succinyl-CoA ligase [ADP-forming] subunit beta, mitochondrial; AltName: Full=ATP-specific succinyl-CoA synthetase subunit beta; AltName: Full=Succinyl-CoA synthetase beta-A chain; Short=SCS-betaA; Flags: Precursor |
| gi81905373 | LRGUK | 2 | 1.029519686 | 1.029519686 | 0.041971417 | RecName: Full=Leucine-rich repeat and guanylate kinase domain-containing protein |
| gi20178348 | SEPT6 | 7 | 1.028829552 | 1.028829552 | 0.041003989 | RecName: Full=Septin-6 |
| gi12585517 | ATP6V1G2 | 3 | 1.027464856 | 1.027464856 | 0.039089048 | RecName: Full=V-type proton ATPase subunit G 2; Short=V-ATPase subunit G 2; AltName: Full=V-ATPase 13 kDa subunit 2; AltName: Full=Vacuolar proton pump subunit G 2 |
| gi113334 | AP2A1 | 12 | 1.027213242 | 1.027213242 | 0.038735706 | RecName: Full=AP-2 complex subunit alpha-1; AltName: Full=100 kDa coated vesicle protein A; AltName: Full=Adapter-related protein complex 2 alpha-1 subunit; AltName: Full=Adaptor protein complex AP-2 subunit alpha-1; AltName: Full=Alpha-adaptin A; AltName: Full=Alpha1-adaptin; AltName: Full=Clathrin assembly protein complex 2 alpha-A large chain; AltName: Full=Plasma membrane adaptor HA2/AP2 adaptin alpha A subunit |
| gi32363386 | NDUFA10 | 5 | 1.027182854 | 1.027182854 | 0.038693025 | RecName: Full=NADH dehydrogenase [ubiquinone] 1 alpha subcomplex subunit 10, mitochondrial; AltName: Full=Complex I-42kD; Short=CI-42kD; AltName: Full=NADH-ubiquinone oxidoreductase 42 kDa subunit; Flags: Precursor |
| gi81873664 | SV2B | 4 | 1.027135809 | 1.027135809 | 0.03862695 | RecName: Full=Synaptic vesicle glycoprotein 2B; Short=Synaptic vesicle protein 2B |
| gi97535655 | ANK1 | 3 | 1.026480985 | 1.026480985 | 0.037706903 | RecName: Full=Ankyrin-1; Short=ANK-1; AltName: Full=Erythrocyte ankyrin |
| gi51702142 | UBE2V2 | 2 | 1.026311883 | 1.026311883 | 0.037469214 | RecName: Full=Ubiquitin-conjugating enzyme E2 variant 2; AltName: Full=Ubc-like protein MMS2 |
| gi266608 | NME2 | 5 | 1.026288628 | 1.026288628 | 0.037436523 | RecName: Full=Nucleoside diphosphate kinase B; Short=NDK B; Short=NDP kinase B; AltName: Full=Histidine protein kinase NDKB; AltName: Full=P18; AltName: Full=nm23-M2 |
| gi2493731 | CLTA | 6 | 1.026206882 | 1.026206882 | 0.037321606 | RecName: Full=Clathrin light chain A; Short=Lca |
| gi20978552 | PSMD6 | 2 | 1.026045874 | 1.026045874 | 0.037095235 | RecName: Full=26S proteasome non-ATPase regulatory subunit 6; AltName: Full=26S proteasome regulatory subunit RPN7; AltName: Full=26S proteasome regulatory subunit S10; AltName: Full=p42A |
| gi1709255 | NCAN | 2 | 1.025690731 | 1.025690731 | 0.036595792 | RecName: Full=Neurocan core protein; AltName: Full=Chondroitin sulfate proteoglycan 3; Flags: Precursor |
| gi2507330 | EIF4A2 | 5 | 1.025371383 | 1.025371383 | 0.03614654 | RecName: Full=Eukaryotic initiation factor 4A-II; Short=eIF-4A-II; Short=eIF4A-II; AltName: Full=ATP-dependent RNA helicase eIF4A-2 |
| gi341940472 | DYNC1H1 | 24 | 1.025137582 | 1.025137582 | 0.035817545 | RecName: Full=Cytoplasmic dynein 1 heavy chain 1; AltName: Full=Cytoplasmic dynein heavy chain 1; AltName: Full=Dynein heavy chain, cytosolic |
| gi46577116 | RAB1B | 4 | 1.024533521 | 1.024533521 | 0.034967188 | RecName: Full=Ras-related protein Rab-1B |
| gi13638404 | RAB6A | 3 | 1.023441987 | 1.023441987 | 0.033429326 | RecName: Full=Ras-related protein Rab-6A; Short=Rab-6 |
| gi15214055 | EIF4H | 2 | 1.023315011 | 1.023315011 | 0.033250324 | RecName: Full=Eukaryotic translation initiation factor 4H; Short=eIF-4H; AltName: Full=Williams-Beuren syndrome chromosomal region 1 protein homolog |
| gi20141789 | PRDX5 | 9 | 1.023141382 | 1.023141382 | 0.033005517 | RecName: Full=Peroxiredoxin-5, mitochondrial; AltName: Full=Antioxidant enzyme B166; Short=AOEB166; AltName: Full=Liver tissue 2D-page spot 2D-0014IV; AltName: Full=PLP; AltName: Full=Peroxiredoxin V; Short=Prx-V; AltName: Full=Peroxisomal antioxidant enzyme; AltName: Full=Thioredoxin peroxidase PMP20; AltName: Full=Thioredoxin reductase; Flags: Precursor |
| gi52000877 | UQCRFS1 | 3 | 1.023122258 | 1.023122258 | 0.03297855 | RecName: Full=Cytochrome b-c1 complex subunit Rieske, mitochondrial; AltName: Full=Complex III subunit 5; AltName: Full=Cytochrome b-c1 complex subunit 5; AltName: Full=Rieske iron-sulfur protein; Short=RISP; AltName: Full=Ubiquinol-cytochrome c reductase iron-sulfur subunit; Contains: RecName: Full=Cytochrome b-c1 complex subunit 11; AltName: Full=Complex III subunit IX; AltName: Full=Ubiquinol-cytochrome c reductase 8 kDa protein; Flags: Precursor |
| gi146345422 | GPI | 15 | 1.023032288 | 1.023032288 | 0.032851679 | RecName: Full=Glucose-6-phosphate isomerase; Short=GPI; AltName: Full=Autocrine motility factor; Short=AMF; AltName: Full=Neuroleukin; Short=NLK; AltName: Full=Phosphoglucose isomerase; Short=PGI; AltName: Full=Phosphohexose isomerase; Short=PHI |
| gi20454881 | DBN1 | 7 | 1.02271386 | 1.02271386 | 0.032402557 | RecName: Full=Drebrin; AltName: Full=Developmentally-regulated brain protein |
| gi2506545 | HSPA5 | 11 | 1.022191293 | 1.022191293 | 0.031665207 | RecName: Full=78 kDa glucose-regulated protein; Short=GRP-78; AltName: Full=Heat shock 70 kDa protein 5; AltName: Full=Immunoglobulin heavy chain-binding protein; Short=BiP; Flags: Precursor |
| gi50401075 | ITPKA | 2 | 1.021012813 | 1.021012813 | 0.030000971 | RecName: Full=Inositol-trisphosphate 3-kinase A; AltName: Full=Inositol 1,4,5-trisphosphate 3-kinase A; Short=IP3 3-kinase A; Short=IP3K A; Short=InsP 3-kinase A |
| gi94730407 | PCLO | 2 | 1.02080918 | 1.02080918 | 0.029713208 | RecName: Full=Protein piccolo; AltName: Full=Aczonin; AltName: Full=Brain-derived HLMN protein; AltName: Full=Multidomain presynaptic cytomatrix protein |
| gi56749655 | SEPT9 | 2 | 1.0204793 | 1.0204793 | 0.029246918 | RecName: Full=Septin-9; AltName: Full=SL3-3 integration site 1 protein |
| gi3913897 | ATPIF1 | 3 | 1.020359316 | 1.020359316 | 0.029077282 | RecName: Full=ATPase inhibitor, mitochondrial; AltName: Full=Inhibitor of F(1)F(o)-ATPase; Short=IF(1); Short=IF1; Flags: Precursor |
| gi41019466 | SYP | 5 | 1.01997613 | 1.01997613 | 0.02853539 | RecName: Full=Synaptophysin; AltName: Full=BM89 antigen; AltName: Full=Major synaptic vesicle protein p38 |
| gi172044688 | EHD3 | 5 | 1.019892133 | 1.019892133 | 0.028416576 | RecName: Full=EH domain-containing protein 3 |
| gi48428148 | COX6C | 2 | 1.019877501 | 1.019877501 | 0.028395879 | RecName: Full=Cytochrome c oxidase subunit 6C; AltName: Full=Cytochrome c oxidase polypeptide VIc |
| gi81898160 | DNM3 | 12 | 1.019612603 | 1.019612603 | 0.028021111 | RecName: Full=Dynamin-3 |
| gi124056467 | CAMK2A | 16 | 1.01934686 | 1.01934686 | 0.02764505 | RecName: Full=Calcium/calmodulin-dependent protein kinase type II subunit alpha; Short=CaM kinase II subunit alpha; Short=CaMK-II subunit alpha |
| gi46397725 | SNAP25 | 9 | 1.019329033 | 1.019329033 | 0.02761982 | RecName: Full=Synaptosomal-associated protein 25; Short=SNAP-25; AltName: Full=Super protein; Short=SUP; AltName: Full=Synaptosomal-associated 25 kDa protein |
| gi78099818 | ATAD3A/ATAD3B | 3 | 1.019226191 | 1.019226191 | 0.027474256 | RecName: Full=ATPase family AAA domain-containing protein 3; AltName: Full=AAA-ATPase TOB3 |
| gi13959400 | AK1 | 5 | 1.018500834 | 1.018500834 | 0.026447161 | RecName: Full=Adenylate kinase isoenzyme 1; Short=AK 1; AltName: Full=ATP-AMP transphosphorylase 1; AltName: Full=Myokinase |
| gi29840839 | PEBP1 | 6 | 1.018083489 | 1.018083489 | 0.025855876 | RecName: Full=Phosphatidylethanolamine-binding protein 1; Short=PEBP-1; AltName: Full=HCNPpp; Contains: RecName: Full=Hippocampal cholinergic neurostimulating peptide; Short=HCNP |
| gi119362 | HSP90B1 | 4 | 1.017929912 | 1.017929912 | 0.025638231 | RecName: Full=Endoplasmin; AltName: Full=94 kDa glucose-regulated protein; Short=GRP-94; AltName: Full=Endoplasmic reticulum resident protein 99; Short=ERp99; AltName: Full=Heat shock protein 90 kDa beta member 1; AltName: Full=Polymorphic tumor rejection antigen 1; AltName: Full=Tumor rejection antigen gp96; Flags: Precursor |
| gi8928249 | PTGES3 | 2 | 1.017176287 | 1.017176287 | 0.024569734 | RecName: Full=Prostaglandin E synthase 3; AltName: Full=Cytosolic prostaglandin E2 synthase; Short=cPGES; AltName: Full=Hsp90 co-chaperone; AltName: Full=Progesterone receptor complex p23; AltName: Full=Sid 3177; AltName: Full=Telomerase-binding protein p23 |
| gi38258239 | RAB3B | 4 | 1.017027971 | 1.017027971 | 0.024359358 | RecName: Full=Ras-related protein Rab-3B |
| gi341941063 | HK1 | 25 | 1.016780398 | 1.016780398 | 0.024008122 | RecName: Full=Hexokinase-1; AltName: Full=Hexokinase type I; Short=HK I; AltName: Full=Hexokinase, tumor isozyme |
| gi66773801 | CLTC | 59 | 1.01644187 | 1.01644187 | 0.023527711 | RecName: Full=Clathrin heavy chain 1 |
| gi38258917 | RAB5C | 3 | 1.016427855 | 1.016427855 | 0.023507818 | RecName: Full=Ras-related protein Rab-5C |
| gi62286986 | NUDC | 2 | 1.016237124 | 1.016237124 | 0.023237073 | RecName: Full=Nuclear migration protein nudC; AltName: Full=Nuclear distribution protein C homolog; AltName: Full=Silica-induced gene 92 protein; Short=SIG-92 |
| gi60391192 | YWHAE | 14 | 1.015511706 | 1.015511706 | 0.022206869 | RecName: Full=14-3-3 protein epsilon; Short=14-3-3E |
| gi71152120 | HGS | 2 | 1.015131655 | 1.015131655 | 0.021666846 | RecName: Full=Hepatocyte growth factor-regulated tyrosine kinase substrate |
| gi23831273 | PPP1R1B | 3 | 1.014617529 | 1.014617529 | 0.020935991 | RecName: Full=Protein phosphatase 1 regulatory subunit 1B; AltName: Full=DARPP-32; AltName: Full=Dopamine- and cAMP-regulated neuronal phosphoprotein |
| gi119348 | ENO2 | 18 | 1.014242348 | 1.014242348 | 0.020402418 | RecName: Full=Gamma-enolase; AltName: Full=2-phospho-D-glycerate hydro-lyase; AltName: Full=Enolase 2; AltName: Full=Neural enolase; AltName: Full=Neuron-specific enolase; Short=NSE |
| gi46577103 | RAB14 | 2 | 1.013661998 | 1.013661998 | 0.019576672 | RecName: Full=Ras-related protein Rab-14 |
| gi51702798 | RAB3A | 10 | 1.013419763 | 1.013419763 | 0.019231869 | RecName: Full=Ras-related protein Rab-3A |
| gi12643287 | ATP6V0D1 | 3 | 1.012997988 | 1.012997988 | 0.018631308 | RecName: Full=V-type proton ATPase subunit d 1; Short=V-ATPase subunit d 1; AltName: Full=P39; AltName: Full=Physophilin; AltName: Full=V-ATPase 40 kDa accessory protein; AltName: Full=V-ATPase AC39 subunit; AltName: Full=Vacuolar proton pump subunit d 1 |
| gi327478516 | HYDIN | 4 | 1.012288608 | 1.012288608 | 0.017620667 | RecName: Full=Hydrocephalus-inducing protein; AltName: Full=Protein Hy-3 |
| gi50402098 | EEF1A2 | 10 | 1.012043584 | 1.012043584 | 0.017271422 | RecName: Full=Elongation factor 1-alpha 2; Short=EF-1-alpha-2; AltName: Full=Eukaryotic elongation factor 1 A-2; Short=eEF1A-2; AltName: Full=Statin-S1 |
| gi17380333 | UQCRB | 8 | 1.011803295 | 1.011803295 | 0.016928843 | RecName: Full=Cytochrome b-c1 complex subunit 7; AltName: Full=Complex III subunit 7; AltName: Full=Complex III subunit VII; AltName: Full=Ubiquinol-cytochrome c reductase complex 14 kDa protein |
| gi54036445 | STIP1 | 5 | 1.011708473 | 1.011708473 | 0.016793632 | RecName: Full=Stress-induced-phosphoprotein 1; Short=STI1; Short=mSTI1; AltName: Full=Hsc70/Hsp90-organizing protein; Short=Hop |
| gi130914 | PRNP | 2 | 1.011582277 | 1.011582277 | 0.016613666 | RecName: Full=Major prion protein; Short=PrP; AltName: Full=PrP27-30; AltName: Full=PrP33-35C; AltName: CD_antigen=CD230; Flags: Precursor |
| gi416677 | ATP5A1 | 22 | 1.011512156 | 1.011512156 | 0.016513657 | RecName: Full=ATP synthase subunit alpha, mitochondrial; Flags: Precursor |
| gi46577689 | RAB21 | 3 | 1.011423362 | 1.011423362 | 0.016387008 | RecName: Full=Ras-related protein Rab-21; AltName: Full=Rab-12; Flags: Precursor |
| gi81902298 | BRK1 | 2 | 1.011248561 | 1.011248561 | 0.016137649 | RecName: Full=Protein BRICK1; Short=BRK1 |
| gi341941233 | PCCB | 2 | 1.011196688 | 1.011196688 | 0.016063644 | RecName: Full=Propionyl-CoA carboxylase beta chain, mitochondrial; Short=PCCase subunit beta; AltName: Full=Propanoyl-CoA:carbon dioxide ligase subunit beta; Flags: Precursor |
| gi42558958 | Ppfia3 | 2 | 1.010693299 | 1.010693299 | 0.015345269 | RecName: Full=Liprin-alpha-3; AltName: Full=Protein tyrosine phosphatase receptor type f polypeptide-interacting protein alpha-3; Short=PTPRF-interacting protein alpha-3 |
| gi2492687 | SNAP91 | 7 | 1.010436261 | 1.010436261 | 0.014978319 | RecName: Full=Clathrin coat assembly protein AP180; AltName: Full=91 kDa synaptosomal-associated protein; AltName: Full=Clathrin coat-associated protein AP180; AltName: Full=Phosphoprotein F1-20 |
| gi20981714 | SYNGR1 | 2 | 1.010376046 | 1.010376046 | 0.014892342 | RecName: Full=Synaptogyrin-1 |
| gi66773992 | ATP1A2 | 34 | 1.00985228 | 1.00985228 | 0.014144273 | RecName: Full=Sodium/potassium-transporting ATPase subunit alpha-2; Short=Na(+)/K(+) ATPase alpha-2 subunit; AltName: Full=Na(+)/K(+) ATPase alpha(+) subunit; AltName: Full=Sodium pump subunit alpha-2; Flags: Precursor |
| gi13124470 | PFN2 | 5 | 1.00931385 | 1.00931385 | 0.013374855 | RecName: Full=Profilin-2; AltName: Full=Profilin II |
| gi73621117 | KIAA1045 | 5 | 1.009220905 | 1.009220905 | 0.013241996 | RecName: Full=Protein KIAA1045 |
| gi1174545 | SYT1 (includes EG:20979) | 11 | 1.008974214 | 1.008974214 | 0.012889305 | RecName: Full=Synaptotagmin-1; AltName: Full=Synaptotagmin I; Short=SytI; AltName: Full=p65 |
| gi341942004 | SLC6A11 | 3 | 1.008850003 | 1.008850003 | 0.012711689 | RecName: Full=Sodium- and chloride-dependent GABA transporter 3; Short=GAT-3; AltName: Full=Sodium- and chloride-dependent GABA transporter 4; Short=GAT-4; AltName: Full=Solute carrier family 6 member 11 |
| gi97536358 | HSPH1 | 2 | 1.008809533 | 1.008809533 | 0.012653814 | RecName: Full=Heat shock protein 105 kDa; AltName: Full=42 degrees C-HSP; AltName: Full=Heat shock 110 kDa protein; AltName: Full=Heat shock-related 100 kDa protein E7I; Short=HSP-E7I |
| gi122028 | HIST1H2BN | 8 | 1.008065898 | 1.008065898 | 0.011589952 | RecName: Full=Histone H2B type 1-M; AltName: Full=H2B 291B |
| gi212288549 | USP24 | 2 | 1.008009835 | 1.008009835 | 0.011509715 | RecName: Full=Ubiquitin carboxyl-terminal hydrolase 24; AltName: Full=Deubiquitinating enzyme 24; AltName: Full=Ubiquitin thiolesterase 24; AltName: Full=Ubiquitin-specific-processing protease 24 |
| gi73920802 | SYN1 | 24 | 1.007723497 | 1.007723497 | 0.011099841 | PRKACB |
| gi462064 | FABP5 | 2 | 1.007330342 | 1.007330342 | 0.010536875 | RecName: Full=Fatty acid-binding protein, epidermal; AltName: Full=Epidermal-type fatty acid-binding protein; Short=E-FABP; AltName: Full=Fatty acid-binding protein 5; AltName: Full=Keratinocyte lipid-binding protein; AltName: Full=Psoriasis-associated fatty acid-binding protein homolog; Short=PA-FABP |
| gi223634732 | SUCLG1 | 5 | 1.007011988 | 1.007011988 | 0.010080858 | RecName: Full=Succinyl-CoA ligase [ADP/GDP-forming] subunit alpha, mitochondrial; AltName: Full=Succinyl-CoA synthetase subunit alpha; Short=SCS-alpha; Flags: Precursor |
| gi5915682 | ALB | 15 | 1.006169563 | 1.006169563 | 0.008873453 | RecName: Full=Serum albumin; Flags: Precursor |
| gi54039385 | RPS18 | 4 | 1.005941738 | 1.005941738 | 0.00854675 | RecName: Full=40S ribosomal protein S18; AltName: Full=Ke-3; Short=Ke3 |
| gi113607 | ALDOA | 18 | 1.005576231 | 1.005576231 | 0.008022454 | RecName: Full=Fructose-bisphosphate aldolase A; AltName: Full=Aldolase 1; AltName: Full=Muscle-type aldolase |
| gi417489 | PCMT1 | 4 | 1.005570816 | 1.005570816 | 0.008014685 | RecName: Full=Protein-L-isoaspartate(D-aspartate) O-methyltransferase; Short=PIMT; AltName: Full=L-isoaspartyl protein carboxyl methyltransferase; AltName: Full=Protein L-isoaspartyl/D-aspartyl methyltransferase; AltName: Full=Protein-beta-aspartate methyltransferase |
| gi10719868 | ADD1 | 8 | 1.004861046 | 1.004861046 | 0.006996016 | RecName: Full=Alpha-adducin; AltName: Full=Erythrocyte adducin subunit alpha |
| gi84029467 | SLC4A4 | 3 | 1.004102554 | 1.004102554 | 0.005906626 | RecName: Full=Electrogenic sodium bicarbonate cotransporter 1; Short=Sodium bicarbonate cotransporter; AltName: Full=Na(+)/HCO3(-) cotransporter; AltName: Full=Solute carrier family 4 member 4 |
| gi51703328 | TPT1 (includes EG:100043703) | 2 | 1.003865451 | 1.003865451 | 0.005565916 | RecName: Full=Translationally-controlled tumor protein; Short=TCTP; AltName: Full=21 kDa polypeptide; AltName: Full=p21; AltName: Full=p23 |
| gi48428722 | YWHAG | 13 | 1.003806661 | 1.003806661 | 0.005481425 | RecName: Full=14-3-3 protein gamma; Contains: RecName: Full=14-3-3 protein gamma, N-terminally processed |
| gi9789726 | SEPT7 | 16 | 1.003530644 | 1.003530644 | 0.005084672 | RecName: Full=Septin-7; AltName: Full=CDC10 protein homolog |
| gi21759002 | NDUFAB1 | 2 | 1.003528253 | 1.003528253 | 0.005081234 | RecName: Full=Acyl carrier protein, mitochondrial; Short=ACP; AltName: Full=CI-SDAP; AltName: Full=NADH-ubiquinone oxidoreductase 9.6 kDa subunit; Flags: Precursor |
| gi48427970 | ABAT | 14 | 1.002671443 | 1.002671443 | 0.003848938 | RecName: Full=4-aminobutyrate aminotransferase, mitochondrial; AltName: Full=(S)-3-amino-2-methylpropionate transaminase; AltName: Full=GABA aminotransferase; Short=GABA-AT; AltName: Full=Gamma-amino-N-butyrate transaminase; Short=GABA transaminase; Short=GABA-T; AltName: Full=L-AIBAT; Flags: Precursor |
| gi60390207 | MAPRE2 | 3 | 1.002606652 | 1.002606652 | 0.003755711 | RecName: Full=Microtubule-associated protein RP/EB family member 2; AltName: Full=APC-binding protein EB2; AltName: Full=End-binding protein 2; Short=EB2 |
| gi97537229 | SPTBN1 | 58 | 1.002602377 | 1.002602377 | 0.00374956 | RecName: Full=Spectrin beta chain, brain 1; AltName: Full=Beta-II spectrin; AltName: Full=Embryonic liver fodrin; AltName: Full=Fodrin beta chain; AltName: Full=Spectrin, non-erythroid beta chain 1 |
| gi22002044 | DNM2 | 8 | 1.0025844 | 1.0025844 | 0.003723691 | RecName: Full=Dynamin-2; AltName: Full=Dynamin UDNM |
| gi52000885 | YWHAZ | 15 | 1.001889468 | 1.001889468 | 0.002723354 | RecName: Full=14-3-3 protein zeta/delta; AltName: Full=Protein kinase C inhibitor protein 1; Short=KCIP-1; AltName: Full=SEZ-2 |
| gi547881 | GPM6A | 5 | 1.001711562 | 1.001711562 | 0.002467152 | RecName: Full=Neuronal membrane glycoprotein M6-a; Short=M6a |
| gi19862081 | UQCRHL | 3 | 1.000314253 | 1.000314253 | 0.0004533 | RecName: Full=Cytochrome b-c1 complex subunit 6, mitochondrial; AltName: Full=Complex III subunit 6; AltName: Full=Complex III subunit VIII; AltName: Full=Cytochrome c1 non-heme 11 kDa protein; AltName: Full=Mitochondrial hinge protein; AltName: Full=Ubiquinol-cytochrome c reductase complex 11 kDa protein; Flags: Precursor |
| gi146345457 | MDH2 (includes EG:17448) | 15 | 1.000296486 | 1.000296486 | 0.000427675 | RecName: Full=Malate dehydrogenase, mitochondrial; Flags: Precursor |
| gi125987813 | COL6A2 | 2 | 1.000192951 | 1.000192951 | 0.000278342 | RecName: Full=Collagen alpha-2(VI) chain; Flags: Precursor |
| gi300669660 | NPEPPS | 2 | 1.000139284 | 1.000139284 | 0.00020093 | RecName: Full=Puromycin-sensitive aminopeptidase; Short=PSA; AltName: Full=Cytosol alanyl aminopeptidase; Short=AAP-S |
| gi14917005 | HSPA9 | 9 | 0.9999715 | 0.9999715 | -4.11171E-05 | RecName: Full=Stress-70 protein, mitochondrial; AltName: Full=75 kDa glucose-regulated protein; Short=GRP-75; AltName: Full=Heat shock 70 kDa protein 9; AltName: Full=Mortalin; AltName: Full=Peptide-binding protein 74; Short=PBP74; AltName: Full=p66 MOT; Flags: Precursor |
| gi146291096 | ATP6V1C1 | 6 | 0.999927612 | 0.999927612 | -0.000104437 | RecName: Full=V-type proton ATPase subunit C 1; Short=V-ATPase subunit C 1; AltName: Full=Vacuolar proton pump subunit C 1 |
| gi62510439 | CLTB | 5 | 0.999583245 | 0.999583245 | -0.000601375 | RecName: Full=Clathrin light chain B; Short=Lcb |
| gi81885902 | CYFIP1 | 4 | 0.999533808 | 0.999533808 | -0.000672729 | RecName: Full=Cytoplasmic FMR1-interacting protein 1; AltName: Full=Specifically Rac1-associated protein 1; Short=Sra-1 |
| gi6093770 | VDAC3 | 6 | 0.99919655 | 0.99919655 | -0.0011596 | RecName: Full=Voltage-dependent anion-selective channel protein 3; Short=VDAC-3; Short=mVDAC3; AltName: Full=Outer mitochondrial membrane protein porin 3 |
| gi2829482 | SLC8A1 | 3 | 0.998661097 | 0.998661097 | -0.001932923 | RecName: Full=Sodium/calcium exchanger 1; AltName: Full=Na(+)/Ca(2+)-exchange protein 1; Flags: Precursor |
| gi341941003 | MFN1 | 2 | 0.998527037 | 0.998527037 | -0.002126603 | RecName: Full=Mitofusin-1; AltName: Full=Transmembrane GTPase MFN1 |
| gi38605093 | HOMER1 | 4 | 0.998524099 | 0.998524099 | -0.002130848 | RecName: Full=Homer protein homolog 1; Short=Homer-1; AltName: Full=VASP/Ena-related gene up-regulated during seizure and LTP 1; Short=Vesl-1 |
| gi135831 | THY1 | 3 | 0.998232816 | 0.998232816 | -0.002551763 | RecName: Full=Thy-1 membrane glycoprotein; AltName: Full=Thy-1 antigen; AltName: CD_antigen=CD90; Flags: Precursor |
| gi146345497 | ATIC | 4 | 0.997990585 | 0.997990585 | -0.00290189 | RecName: Full=Bifunctional purine biosynthesis protein PURH; Includes: RecName: Full=Phosphoribosylaminoimidazolecarboxamide formyltransferase; AltName: Full=5-aminoimidazole-4-carboxamide ribonucleotide formyltransferase; AltName: Full=AICAR transformylase; Includes: RecName: Full=IMP cyclohydrolase; AltName: Full=ATIC; AltName: Full=IMP synthase; AltName: Full=Inosinicase |
| gi2500528 | DDX3X | 2 | 0.997818585 | 0.997818585 | -0.003150555 | RecName: Full=ATP-dependent RNA helicase DDX3X; AltName: Full=D1Pas1-related sequence 2; AltName: Full=DEAD box RNA helicase DEAD3; Short=mDEAD3; AltName: Full=DEAD box protein 3, X-chromosomal; AltName: Full=Embryonic RNA helicase |
| gi341940935 | MAP2 | 26 | 0.997486883 | 0.997486883 | -0.003630225 | RecName: Full=Microtubule-associated protein 2; Short=MAP-2 |
| gi52783073 | LSAMP | 4 | 0.996913914 | 0.996913914 | -0.004459166 | RecName: Full=Limbic system-associated membrane protein; Short=LSAMP; Flags: Precursor |
| gi21759079 | CYC1 | 4 | 0.996892715 | 0.996892715 | -0.004489843 | RecName: Full=Cytochrome c1, heme protein, mitochondrial; AltName: Full=Complex III subunit 4; AltName: Full=Complex III subunit IV; AltName: Full=Cytochrome b-c1 complex subunit 4; AltName: Full=Ubiquinol-cytochrome-c reductase complex cytochrome c1 subunit; Short=Cytochrome c-1; Flags: Precursor |
| gi32363402 | NDUFA8 | 3 | 0.9966944 | 0.9966944 | -0.004776872 | RecName: Full=NADH dehydrogenase [ubiquinone] 1 alpha subcomplex subunit 8; AltName: Full=Complex I-19kD; Short=CI-19kD; AltName: Full=Complex I-PGIV; Short=CI-PGIV; AltName: Full=NADH-ubiquinone oxidoreductase 19 kDa subunit |
| gi44888264 | OTUB1 | 3 | 0.996555719 | 0.996555719 | -0.004977625 | RecName: Full=Ubiquitin thioesterase OTUB1; AltName: Full=Deubiquitinating enzyme OTUB1; AltName: Full=OTU domain-containing ubiquitin aldehyde-binding protein 1; AltName: Full=Otubain-1; AltName: Full=Ubiquitin-specific-processing protease OTUB1 |
| gi73920803 | SYN2 | 13 | 0.996524187 | 0.996524187 | -0.005023273 | RecName: Full=Synapsin-2; AltName: Full=Synapsin II |
| gi51702275 | HSPA8 | 31 | 0.996446321 | 0.996446321 | -0.005136007 | RecName: Full=Heat shock cognate 71 kDa protein; AltName: Full=Heat shock 70 kDa protein 8 |
| gi20138335 | GNG12 | 4 | 0.996410346 | 0.996410346 | -0.005188094 | RecName: Full=Guanine nucleotide-binding protein G(I)/G(S)/G(O) subunit gamma-12; Flags: Precursor |
| gi3219774 | PRDX6 | 9 | 0.996409287 | 0.996409287 | -0.005189626 | RecName: Full=Peroxiredoxin-6; AltName: Full=1-Cys peroxiredoxin; Short=1-Cys PRX; AltName: Full=Acidic calcium-independent phospholipase A2; Short=aiPLA2; AltName: Full=Antioxidant protein 2; AltName: Full=Non-selenium glutathione peroxidase; Short=NSGPx |
| gi146345480 | PDIA3 | 7 | 0.996240029 | 0.996240029 | -0.005434714 | RecName: Full=Protein disulfide-isomerase A3; AltName: Full=58 kDa glucose-regulated protein; AltName: Full=58 kDa microsomal protein; Short=p58; AltName: Full=Disulfide isomerase ER-60; AltName: Full=Endoplasmic reticulum resident protein 57; Short=ER protein 57; Short=ERp57; AltName: Full=Endoplasmic reticulum resident protein 60; Short=ER protein 60; Short=ERp60; Flags: Precursor |
| gi134614 | SOD1 | 7 | 0.996215735 | 0.996215735 | -0.005469897 | RecName: Full=Superoxide dismutase [Cu-Zn] |
| gi9910833 | PSMA1 | 2 | 0.995776641 | 0.995776641 | -0.006105921 | RecName: Full=Proteasome subunit alpha type-1; AltName: Full=Macropain subunit C2; AltName: Full=Multicatalytic endopeptidase complex subunit C2; AltName: Full=Proteasome component C2; AltName: Full=Proteasome nu chain |
| gi122066202 | SPTAN1 | 99 | 0.995629587 | 0.995629587 | -0.006318991 | RecName: Full=Spectrin alpha chain, brain; AltName: Full=Alpha-II spectrin; AltName: Full=Fodrin alpha chain; AltName: Full=Spectrin, non-erythroid alpha chain |
| gi341941800 | RASAL1 | 4 | 0.995565512 | 0.995565512 | -0.006411841 | RecName: Full=RasGAP-activating-like protein 1 |
| gi146345470 | NSF | 31 | 0.99548923 | 0.99548923 | -0.006522387 | RecName: Full=Vesicle-fusing ATPase; AltName: Full=N-ethylmaleimide-sensitive fusion protein; Short=NEM-sensitive fusion protein; AltName: Full=Suppressor of K(+) transport growth defect 2; Short=Protein SKD2; AltName: Full=Vesicular-fusion protein NSF |
| gi81906751 | HYOU1 | 2 | 0.995475625 | 0.995475625 | -0.006542104 | RecName: Full=Hypoxia up-regulated protein 1; Short=GRP-170; AltName: Full=140 kDa Ca(2+)-binding protein; Short=CBP-140; Flags: Precursor |
| gi145559539 | ATP6V1A | 17 | 0.995386266 | 0.995386266 | -0.006671613 | RecName: Full=V-type proton ATPase catalytic subunit A; Short=V-ATPase subunit A; AltName: Full=V-ATPase 69 kDa subunit; AltName: Full=Vacuolar proton pump subunit alpha |
| gi46397464 | EIF4A1 | 3 | 0.994969798 | -1.005055632 | -0.007275361 | RecName: Full=Eukaryotic initiation factor 4A-I; Short=eIF-4A-I; Short=eIF4A-I; AltName: Full=ATP-dependent RNA helicase eIF4A-1 |
| gi21903382 | SLC25A4 | 14 | 0.994954024 | -1.005071567 | -0.007298233 | RecName: Full=ADP/ATP translocase 1; AltName: Full=ADP,ATP carrier protein 1; AltName: Full=ADP,ATP carrier protein, heart/skeletal muscle isoform T1; AltName: Full=Adenine nucleotide translocator 1; Short=ANT 1; AltName: Full=Solute carrier family 25 member 4; AltName: Full=mANC1 |
| gi46396900 | PYGB | 17 | 0.994710836 | -1.005317288 | -0.007650902 | RecName: Full=Glycogen phosphorylase, brain form |
| gi3122052 | DPYSL3 | 5 | 0.994569055 | -1.005460601 | -0.00785655 | RecName: Full=Dihydropyrimidinase-related protein 3; Short=DRP-3; AltName: Full=Unc-33-like phosphoprotein 1; Short=ULIP-1 |
| gi51702234 | CYCS | 5 | 0.994397998 | -1.005633561 | -0.008104703 | RecName: Full=Cytochrome c, somatic |
| gi254763295 | FLOT2 | 2 | 0.994202702 | -1.005831103 | -0.008388071 | RecName: Full=Flotillin-2; AltName: Full=Epidermal surface antigen; Short=ESA; AltName: Full=Membrane component chromosome 17 surface marker 1 homolog |
| gi341940739 | GPHN | 2 | 0.994074141 | -1.005961184 | -0.008574638 | RecName: Full=Gephyrin; Includes: RecName: Full=Molybdopterin adenylyltransferase; Short=MPT adenylyltransferase; AltName: Full=Domain G; Includes: RecName: Full=Molybdopterin molybdenumtransferase; Short=MPT Mo-transferase; AltName: Full=Domain E |
| gi292630942 | SYNE1 | 3 | 0.993977704 | -1.006058784 | -0.008714604 | RecName: Full=Nesprin-1; AltName: Full=Enaptin; AltName: Full=Myocyte nuclear envelope protein 1; Short=Myne-1; AltName: Full=Nuclear envelope spectrin repeat protein 1; AltName: Full=Synaptic nuclear envelope protein 1; Short=Syne-1 |
| gi464569 | RAB3D | 5 | 0.993684239 | -1.006355904 | -0.009140613 | RecName: Full=Ras-related protein Rab-3D |
| gi68566306 | DNM1L | 7 | 0.993517533 | -1.006524764 | -0.009382667 | RecName: Full=Dynamin-1-like protein; AltName: Full=Dynamin family member proline-rich carboxyl-terminal domain less; Short=Dymple; AltName: Full=Dynamin-related protein 1 |
| gi23503090 | NDUFA4 | 4 | 0.99335907 | -1.006685327 | -0.009612791 | RecName: Full=NADH dehydrogenase [ubiquinone] 1 alpha subcomplex subunit 4; AltName: Full=Complex I-MLRQ; Short=CI-MLRQ; AltName: Full=NADH-ubiquinone oxidoreductase MLRQ subunit |
| gi52001076 | MAPK1 | 7 | 0.993310949 | -1.006734096 | -0.009682681 | RecName: Full=Mitogen-activated protein kinase 1; Short=MAP kinase 1; Short=MAPK 1; AltName: Full=ERT1; AltName: Full=Extracellular signal-regulated kinase 2; Short=ERK-2; AltName: Full=MAP kinase isoform p42; Short=p42-MAPK; AltName: Full=Mitogen-activated protein kinase 2; Short=MAP kinase 2; Short=MAPK 2 |
| gi158518557 | FMN1 | 3 | 0.993227232 | -1.006818952 | -0.009804278 | RecName: Full=Formin-1; AltName: Full=Limb deformity protein |
| gi51704260 | BSG (includes EG:12215) | 2 | 0.993011499 | -1.007037684 | -0.010117671 | RecName: Full=Basigin; AltName: Full=Basic immunoglobulin superfamily; AltName: Full=HT7 antigen; AltName: Full=Membrane glycoprotein gp42; AltName: CD_antigen=CD147; Flags: Precursor |
| gi12643614 | ATP2A2 | 7 | 0.992851804 | -1.007199661 | -0.010349702 | RecName: Full=Sarcoplasmic/endoplasmic reticulum calcium ATPase 2; Short=SERCA2; Short=SR Ca(2+)-ATPase 2; AltName: Full=Calcium pump 2; AltName: Full=Calcium-transporting ATPase sarcoplasmic reticulum type, slow twitch skeletal muscle isoform; AltName: Full=Endoplasmic reticulum class 1/2 Ca(2+) ATPase |
| gi47117311 | NDUFS7 | 2 | 0.992792556 | -1.007259768 | -0.010435797 | RecName: Full=NADH dehydrogenase [ubiquinone] iron-sulfur protein 7, mitochondrial; AltName: Full=Complex I-20kD; Short=CI-20kD; AltName: Full=NADH-ubiquinone oxidoreductase 20 kDa subunit; Flags: Precursor |
| gi416827 | COX4I1 | 4 | 0.992660167 | -1.007394105 | -0.010628193 | RecName: Full=Cytochrome c oxidase subunit 4 isoform 1, mitochondrial; AltName: Full=Cytochrome c oxidase polypeptide IV; AltName: Full=Cytochrome c oxidase subunit IV isoform 1; Short=COX IV-1; Flags: Precursor |
| gi27151644 | NTM | 2 | 0.992640983 | -1.007413574 | -0.010656075 | RecName: Full=Neurotrimin; Flags: Precursor |
| gi549060 | CCT7 | 2 | 0.992592695 | -1.007462583 | -0.010726258 | RecName: Full=T-complex protein 1 subunit eta; Short=TCP-1-eta; AltName: Full=CCT-eta |
| gi52000925 | KCNA2 | 4 | 0.992546116 | -1.007509862 | -0.01079396 | RecName: Full=Potassium voltage-gated channel subfamily A member 2; AltName: Full=MK2; AltName: Full=Voltage-gated potassium channel subunit Kv1.2 |
| gi114041 | APOE | 5 | 0.992487183 | -1.007569687 | -0.010879624 | RecName: Full=Apolipoprotein E; Short=Apo-E; Flags: Precursor |
| gi1350822 | HNRNPA1 | 4 | 0.992432071 | -1.007625639 | -0.010959737 | RecName: Full=Heterogeneous nuclear ribonucleoprotein A1; Short=hnRNP A1; AltName: Full=HDP-1; AltName: Full=Helix-destabilizing protein; AltName: Full=Single-strand-binding protein; AltName: Full=Topoisomerase-inhibitor suppressed; AltName: Full=hnRNP core protein A1 |
| gi32469605 | CAP2 | 4 | 0.992256763 | -1.007803662 | -0.011214604 | RecName: Full=Adenylyl cyclase-associated protein 2; Short=CAP 2 |
| gi116849 | CFL1 | 9 | 0.992241104 | -1.007819567 | -0.011237372 | RecName: Full=Cofilin-1; AltName: Full=Cofilin, non-muscle isoform |
| gi1709998 | RAB2A | 4 | 0.992118503 | -1.007944108 | -0.011415642 | RecName: Full=Ras-related protein Rab-2A |
| gi47117855 | GDI1 | 15 | 0.991837848 | -1.008229321 | -0.011823816 | RecName: Full=Rab GDP dissociation inhibitor alpha; Short=Rab GDI alpha; AltName: Full=Guanosine diphosphate dissociation inhibitor 1; Short=GDI-1 |
| gi47605479 | SLC25A12 | 11 | 0.991404287 | -1.00867024 | -0.012454598 | RecName: Full=Calcium-binding mitochondrial carrier protein Aralar1; AltName: Full=Mitochondrial aspartate glutamate carrier 1; AltName: Full=Solute carrier family 25 member 12 |
| gi118542 | GLUD1 | 13 | 0.991387902 | -1.00868691 | -0.012478441 | RecName: Full=Glutamate dehydrogenase 1, mitochondrial; Short=GDH 1; Flags: Precursor |
| gi62286597 | DIRAS2 | 2 | 0.990796193 | -1.009289304 | -0.013339769 | RecName: Full=GTP-binding protein Di-Ras2; AltName: Full=Distinct subgroup of the Ras family member 2; Flags: Precursor |
| gi118572640 | DLD | 6 | 0.990694911 | -1.009392487 | -0.013487254 | RecName: Full=Dihydrolipoyl dehydrogenase, mitochondrial; AltName: Full=Dihydrolipoamide dehydrogenase; Flags: Precursor |
| gi146345421 | FSCN1 | 6 | 0.990497418 | -1.009593747 | -0.013774881 | RecName: Full=Fascin; AltName: Full=Singed-like protein |
| gi52782750 | ATP5L | 2 | 0.990470124 | -1.009621568 | -0.013814635 | RecName: Full=ATP synthase subunit g, mitochondrial; Short=ATPase subunit g |
| gi52782785 | SDHA (includes EG:157074) | 3 | 0.990134274 | -1.009964029 | -0.01430391 | RecName: Full=Succinate dehydrogenase [ubiquinone] flavoprotein subunit, mitochondrial; AltName: Full=Flavoprotein subunit of complex II; Short=Fp; Flags: Precursor |
| gi17380130 | NIPSNAP1 | 4 | 0.989262482 | -1.010854064 | -0.015574732 | RecName: Full=Protein NipSnap homolog 1; Short=NipSnap1 |
| gi342187037 | TNR | 9 | 0.989062908 | -1.011058034 | -0.01586581 | RecName: Full=Tenascin-R; Short=TN-R; AltName: Full=Janusin; AltName: Full=Neural recognition molecule J1-160/180; AltName: Full=Restrictin; Flags: Precursor |
| gi28376967 | CEND1 | 3 | 0.988981394 | -1.011141369 | -0.015984716 | RecName: Full=Cell cycle exit and neuronal differentiation protein 1; AltName: Full=BM88 antigen |
| gi81875980 | ASRGL1 | 2 | 0.988838919 | -1.011287057 | -0.016192569 | RecName: Full=L-asparaginase; AltName: Full=Asparaginase-like protein 1; AltName: Full=L-asparagine amidohydrolase |
| gi20178035 | PGAM1 | 8 | 0.988806938 | -1.011319765 | -0.01623923 | RecName: Full=Phosphoglycerate mutase 1; AltName: Full=BPG-dependent PGAM 1; AltName: Full=Phosphoglycerate mutase isozyme B; Short=PGAM-B |
| gi146345462 | NDUFS3 | 8 | 0.988183083 | -1.011958227 | -0.017149737 | RecName: Full=NADH dehydrogenase [ubiquinone] iron-sulfur protein 3, mitochondrial; AltName: Full=Complex I-30kD; Short=CI-30kD; AltName: Full=NADH-ubiquinone oxidoreductase 30 kDa subunit; Flags: Precursor |
| gi94730399 | NEFH | 10 | 0.987950555 | -1.012196405 | -0.017489256 | RecName: Full=Neurofilament heavy polypeptide; Short=NF-H; AltName: Full=200 kDa neurofilament protein; AltName: Full=Neurofilament triplet H protein |
| gi146345472 | OGDH | 13 | 0.987731637 | -1.012420745 | -0.017808974 | RecName: Full=2-oxoglutarate dehydrogenase, mitochondrial; AltName: Full=2-oxoglutarate dehydrogenase complex component E1; Short=OGDC-E1; AltName: Full=Alpha-ketoglutarate dehydrogenase; Flags: Precursor |
| gi3023546 | COX6B1 | 4 | 0.987198812 | -1.012967183 | -0.018587436 | RecName: Full=Cytochrome c oxidase subunit 6B1; AltName: Full=Cytochrome c oxidase subunit VIb isoform 1; Short=COX VIb-1 |
| gi110287952 | SLC4A10 | 2 | 0.986744902 | -1.013433155 | -0.019250934 | RecName: Full=Sodium-driven chloride bicarbonate exchanger; AltName: Full=Solute carrier family 4 member 10 |
| gi341940560 | CADPS | 5 | 0.986693004 | -1.01348646 | -0.019326815 | RecName: Full=Calcium-dependent secretion activator 1; AltName: Full=Calcium-dependent activator protein for secretion 1; Short=CAPS-1 |
| gi55976751 | ATP1A1 | 31 | 0.986689645 | -1.013489911 | -0.019331727 | RecName: Full=Sodium/potassium-transporting ATPase subunit alpha-1; Short=Na(+)/K(+) ATPase alpha-1 subunit; AltName: Full=Sodium pump subunit alpha-1; Flags: Precursor |
| gi52782731 | PPP2R1A | 9 | 0.986328371 | -1.013861133 | -0.019860063 | RecName: Full=Serine/threonine-protein phosphatase 2A 65 kDa regulatory subunit A alpha isoform; AltName: Full=PP2A subunit A isoform PR65-alpha; AltName: Full=PP2A subunit A isoform R1-alpha |
| gi55977063 | H33 | 4 | 0.985629178 | -1.014580354 | -0.02088313 | RecName: Full=Histone H3.3 |
| gi49065658 | TIMM10 | 2 | 0.985167834 | -1.015055471 | -0.021558571 | RecName: Full=Mitochondrial import inner membrane translocase subunit Tim10 |
| gi66773932 | BASP1 | 8 | 0.984952264 | -1.01527763 | -0.021874289 | RecName: Full=Brain acid soluble protein 1; AltName: Full=22 kDa neuronal tissue-enriched acidic protein; AltName: Full=Neuronal axonal membrane protein NAP-22 |
| gi25453098 | CISD1 | 3 | 0.984911663 | -1.015319483 | -0.02193376 | RecName: Full=CDGSH iron-sulfur domain-containing protein 1; AltName: Full=MitoNEET |
| gi120975 | GNAO1 | 13 | 0.984709865 | -1.015527553 | -0.022229384 | RecName: Full=Guanine nucleotide-binding protein G(o) subunit alpha |
| gi34098594 | EPPK1 | 2 | 0.984399017 | -1.015848231 | -0.022684877 | RecName: Full=Epiplakin |
| gi112804 | SLC3A2 | 5 | 0.983643837 | -1.016628136 | -0.023792064 | RecName: Full=4F2 cell-surface antigen heavy chain; Short=4F2hc; AltName: CD_antigen=CD98 |
| gi120223 | FKBP1A | 2 | 0.983573521 | -1.016700814 | -0.023895198 | RecName: Full=Peptidyl-prolyl cis-trans isomerase FKBP1A; Short=PPIase FKBP1A; AltName: Full=12 kDa FK506-binding protein; Short=12 kDa FKBP; Short=FKBP-12; AltName: Full=FK506-binding protein 1A; Short=FKBP-1A; AltName: Full=Immunophilin FKBP12; AltName: Full=Rotamase |
| gi47117763 | RAP2B | 2 | 0.982916405 | -1.017380517 | -0.024859371 | RecName: Full=Ras-related protein Rap-2b; Flags: Precursor |
| gi51316454 | HPCAL4 | 6 | 0.981278962 | -1.019078202 | -0.027264765 | RecName: Full=Hippocalcin-like protein 4; AltName: Full=Neural visinin-like protein 2; Short=NVP-2 |
| gi353526354 | TPI1 | 10 | 0.980904507 | -1.019467229 | -0.027815401 | RecName: Full=Triosephosphate isomerase; Short=TIM; AltName: Full=Triose-phosphate isomerase |
| gi54037693 | PRKCG | 8 | 0.980739775 | -1.019638466 | -0.028057706 | RecName: Full=Protein kinase C gamma type; Short=PKC-gamma |
| gi134047752 | EPB41L1 | 2 | 0.980627024 | -1.019755702 | -0.028223575 | RecName: Full=Band 4.1-like protein 1; AltName: Full=Neuronal protein 4.1; Short=4.1N |
| gi67460966 | NONO | 7 | 0.980563 | -1.019822286 | -0.02831777 | RecName: Full=Non-POU domain-containing octamer-binding protein; Short=NonO protein |
| gi94717662 | NDUFB6 | 2 | 0.980352406 | -1.020041358 | -0.028627649 | RecName: Full=NADH dehydrogenase [ubiquinone] 1 beta subcomplex subunit 6; AltName: Full=Complex I-B17; Short=CI-B17; AltName: Full=NADH-ubiquinone oxidoreductase B17 subunit |
| gi30581069 | SFXN5 | 3 | 0.979943659 | -1.020466831 | -0.02922929 | RecName: Full=Sideroflexin-5 |
| gi122065897 | PLEC | 10 | 0.979772909 | -1.020644673 | -0.029480694 | RecName: Full=Plectin; Short=PCN; Short=PLTN; AltName: Full=Plectin-1; AltName: Full=Plectin-6 |
| gi21759257 | SLC25A3 | 10 | 0.979745806 | -1.020672907 | -0.029520603 | RecName: Full=Phosphate carrier protein, mitochondrial; AltName: Full=Phosphate transport protein; Short=PTP; AltName: Full=Solute carrier family 25 member 3; Flags: Precursor |
| gi51317340 | H4 | 5 | 0.979625904 | -1.020797833 | -0.029697172 | RecName: Full=Histone H4 |
| gi20138778 | EIF3I | 2 | 0.979025729 | -1.021423616 | -0.03058132 | RecName: Full=Eukaryotic translation initiation factor 3 subunit I; Short=eIF3i; AltName: Full=Eukaryotic translation initiation factor 3 subunit 2; AltName: Full=TGF-beta receptor-interacting protein 1; Short=TRIP-1; AltName: Full=eIF-3-beta; AltName: Full=eIF3 p36 |
| gi126048 | LDHA | 12 | 0.97866487 | -1.021800241 | -0.031113181 | RecName: Full=L-lactate dehydrogenase A chain; Short=LDH-A; AltName: Full=LDH muscle subunit; Short=LDH-M |
| gi205830666 | NCAM1 | 8 | 0.977944393 | -1.022553028 | -0.032175661 | RecName: Full=Neural cell adhesion molecule 1; Short=N-CAM-1; Short=NCAM-1; AltName: CD_antigen=CD56; Flags: Precursor |
| gi82592512 | NCKAP1 | 2 | 0.97783302 | -1.022669495 | -0.032339972 | RecName: Full=Nck-associated protein 1; Short=NAP 1; AltName: Full=Brain protein H19; AltName: Full=MH19; AltName: Full=Membrane-associated protein HEM-2; AltName: Full=p125Nap1 |
| gi51702779 | PFN1 | 2 | 0.977785432 | -1.022719267 | -0.032410184 | RecName: Full=Profilin-1; AltName: Full=Profilin I |
| gi73917637 | BAIAP2 | 2 | 0.977697501 | -1.022811247 | -0.03253993 | RecName: Full=Brain-specific angiogenesis inhibitor 1-associated protein 2; Short=BAI-associated protein 2; Short=BAI1-associated protein 2; AltName: Full=Insulin receptor substrate protein of 53 kDa; Short=IRSp53; Short=Insulin receptor substrate p53; AltName: Full=Insulin receptor tyrosine kinase 53 kDa substrate |
| gi81870005 | NCDN | 14 | 0.977572717 | -1.022941805 | -0.032724073 | RecName: Full=Neurochondrin; AltName: Full=M-Sema F-associating protein of 75 kDa; AltName: Full=Norbin |
| gi33112324 | HSPA12A | 11 | 0.977549661 | -1.022965932 | -0.032758099 | RecName: Full=Heat shock 70 kDa protein 12A |
| gi112984 | GOT2 | 14 | 0.977323848 | -1.02320229 | -0.033091398 | RecName: Full=Aspartate aminotransferase, mitochondrial; Short=mAspAT; AltName: Full=Fatty acid-binding protein; Short=FABP-1; AltName: Full=Glutamate oxaloacetate transaminase 2; AltName: Full=Plasma membrane-associated fatty acid-binding protein; Short=FABPpm; AltName: Full=Transaminase A; Flags: Precursor |
| gi47117767 | STX1B | 10 | 0.976975841 | -1.023566764 | -0.033605208 | RecName: Full=Syntaxin-1B |
| gi51317305 | GNB2 | 9 | 0.976918403 | -1.023626945 | -0.033690029 | RecName: Full=Guanine nucleotide-binding protein G(I)/G(S)/G(T) subunit beta-2; AltName: Full=G protein subunit beta-2; AltName: Full=Transducin beta chain 2 |
| gi81894883 | AHCYL1 | 4 | 0.976568095 | -1.023994134 | -0.03420745 | RecName: Full=Putative adenosylhomocysteinase 2; Short=AdoHcyase 2; AltName: Full=IP3R-binding protein released with inositol 1,4,5-trisphosphate; AltName: Full=S-adenosyl-L-homocysteine hydrolase 2; AltName: Full=S-adenosylhomocysteine hydrolase-like protein 1 |
| gi18202587 | ICAM5 | 3 | 0.976345735 | -1.024227345 | -0.034535982 | RecName: Full=Intercellular adhesion molecule 5; Short=ICAM-5; AltName: Full=Telencephalin; Flags: Precursor |
| gi49065818 | PSMC1 | 2 | 0.976274538 | -1.024302039 | -0.03464119 | RecName: Full=26S protease regulatory subunit 4; Short=P26s4; AltName: Full=26S proteasome AAA-ATPase subunit RPT2; AltName: Full=Proteasome 26S subunit ATPase 1 |
| gi48429206 | STXBP1 | 23 | 0.976145154 | -1.024437806 | -0.034832401 | RecName: Full=Syntaxin-binding protein 1; AltName: Full=Protein unc-18 homolog 1; Short=Unc18-1; AltName: Full=Protein unc-18 homolog A; Short=Unc-18A |
| gi52782768 | C21orf33 | 5 | 0.976118793 | -1.024465472 | -0.034871361 | RecName: Full=ES1 protein homolog, mitochondrial; Flags: Precursor |
| gi143811473 | ATP6V1E1 | 7 | 0.975981425 | -1.024609665 | -0.035074405 | RecName: Full=V-type proton ATPase subunit E 1; Short=V-ATPase subunit E 1; AltName: Full=V-ATPase 31 kDa subunit; Short=p31; AltName: Full=Vacuolar proton pump subunit E 1 |
| gi9910725 | GDA | 3 | 0.975910072 | -1.024684577 | -0.035179882 | RecName: Full=Guanine deaminase; Short=Guanase; Short=Guanine aminase; AltName: Full=Guanine aminohydrolase; Short=GAH |
| gi108935831 | VCAN | 4 | 0.975793101 | -1.02480741 | -0.035352812 | RecName: Full=Versican core protein; AltName: Full=Chondroitin sulfate proteoglycan core protein 2; Short=Chondroitin sulfate proteoglycan 2; AltName: Full=Large fibroblast proteoglycan; AltName: Full=PG-M; Flags: Precursor |
| gi78099820 | ATP5D | 4 | 0.975044696 | -1.025594011 | -0.036459742 | RecName: Full=ATP synthase subunit delta, mitochondrial; AltName: Full=F-ATPase delta subunit; Flags: Precursor |
| gi20454828 | ATP5F1 | 6 | 0.97466302 | -1.025995631 | -0.037024588 | RecName: Full=ATP synthase subunit b, mitochondrial; Short=ATPase subunit b; Flags: Precursor |
| gi48429104 | HNRNPK | 8 | 0.973699921 | -1.027010456 | -0.03845087 | RecName: Full=Heterogeneous nuclear ribonucleoprotein K; Short=hnRNP K |
| gi54038800 | PPP3CA | 15 | 0.972640845 | -1.028128733 | -0.040020918 | RecName: Full=Serine/threonine-protein phosphatase 2B catalytic subunit alpha isoform; AltName: Full=CAM-PRP catalytic subunit; AltName: Full=Calmodulin-dependent calcineurin A subunit alpha isoform |
| gi114978 | SEPT4 | 4 | 0.972351795 | -1.028434364 | -0.040449722 | RecName: Full=Septin-4; AltName: Full=Brain protein H5; AltName: Full=Peanut-like protein 2 |
| gi47117625 | ATP6V1F | 4 | 0.972116483 | -1.028683309 | -0.040798901 | RecName: Full=V-type proton ATPase subunit F; Short=V-ATPase subunit F; AltName: Full=V-ATPase 14 kDa subunit; AltName: Full=Vacuolar proton pump subunit F |
| gi13432217 | SNCA | 5 | 0.972003604 | -1.02880277 | -0.040966432 | RecName: Full=Alpha-synuclein; AltName: Full=Non-A beta component of AD amyloid; AltName: Full=Non-A4 component of amyloid precursor; Short=NACP |
| gi363805626 | SYGP1 | 6 | 0.971968479 | -1.028839948 | -0.041018567 | RecName: Full=Ras/Rap GTPase-activating protein SynGAP; AltName: Full=Neuronal RasGAP; AltName: Full=Synaptic Ras GTPase-activating protein 1; Short=Synaptic Ras-GAP 1 |
| gi120702 | GAPDH | 12 | 0.971917653 | -1.028893752 | -0.04109401 | RecName: Full=Glyceraldehyde-3-phosphate dehydrogenase; Short=GAPDH; AltName: Full=Peptidyl-cysteine S-nitrosylase GAPDH |
| gi57013390 | KCNA6 | 2 | 0.971695778 | -1.029128687 | -0.041423394 | RecName: Full=Potassium voltage-gated channel subfamily A member 6; AltName: Full=MK1.6; AltName: Full=Voltage-gated potassium channel subunit Kv1.6 |
| gi3024764 | USP5 | 4 | 0.971058346 | -1.029804238 | -0.042370112 | RecName: Full=Ubiquitin carboxyl-terminal hydrolase 5; AltName: Full=Deubiquitinating enzyme 5; AltName: Full=Isopeptidase T; AltName: Full=Ubiquitin thiolesterase 5; AltName: Full=Ubiquitin-specific-processing protease 5 |
| gi51338761 | CNP | 20 | 0.970454065 | -1.030445475 | -0.043268168 | RecName: Full=2',3'-cyclic-nucleotide 3'-phosphodiesterase; Short=CNP; Short=CNPase |
| gi94730376 | DPYSL2 | 23 | 0.970374349 | -1.030530126 | -0.043386681 | RecName: Full=Dihydropyrimidinase-related protein 2; Short=DRP-2; AltName: Full=Unc-33-like phosphoprotein 2; Short=ULIP-2 |
| gi18203409 | DSTN | 3 | 0.969457315 | -1.03150493 | -0.044750716 | RecName: Full=Destrin; AltName: Full=Actin-depolymerizing factor; Short=ADF; AltName: Full=Sid 23 |
| gi52782789 | FAM49B | 4 | 0.969067719 | -1.031919628 | -0.04533061 | RecName: Full=Protein FAM49B |
| gi341940718 | KHSRP | 2 | 0.968888382 | -1.032110632 | -0.045597621 | RecName: Full=Far upstream element-binding protein 2; Short=FUSE-binding protein 2; AltName: Full=KH type-splicing regulatory protein; Short=KSRP |
| gi146291078 | VCP | 9 | 0.968731406 | -1.032277878 | -0.04583138 | RecName: Full=Transitional endoplasmic reticulum ATPase; Short=TER ATPase; AltName: Full=15S Mg(2+)-ATPase p97 subunit; AltName: Full=Valosin-containing protein; Short=VCP |
| gi52000832 | ACAT1 | 4 | 0.968609591 | -1.032407699 | -0.046012806 | RecName: Full=Acetyl-CoA acetyltransferase, mitochondrial; AltName: Full=Acetoacetyl-CoA thiolase; Flags: Precursor |
| gi22653923 | PALM | 6 | 0.966963124 | -1.034165601 | -0.048467223 | RecName: Full=Paralemmin-1; AltName: Full=Paralemmin; Flags: Precursor |
| gi60390186 | MAPRE3 | 3 | 0.966920073 | -1.034211646 | -0.048531456 | RecName: Full=Microtubule-associated protein RP/EB family member 3; AltName: Full=EB1 protein family member 3; Short=EBF3; AltName: Full=End-binding protein 3; Short=EB3; AltName: Full=RP3 |
| gi341941093 | FH | 4 | 0.96685978 | -1.034276139 | -0.048621419 | RecName: Full=Fumarate hydratase, mitochondrial; Short=Fumarase; AltName: Full=EF-3; Flags: Precursor |
| gi2495342 | HSPA4 | 8 | 0.9667057 | -1.034440989 | -0.048851347 | RecName: Full=Heat shock 70 kDa protein 4; AltName: Full=Heat shock 70-related protein APG-2 |
| gi124028629 | HNRNPA2B1 | 11 | 0.966177217 | -1.035006811 | -0.049640261 | RecName: Full=Heterogeneous nuclear ribonucleoproteins A2/B1; Short=hnRNP A2/B1 |
| gi160177562 | RTTN | 2 | 0.966006338 | -1.035189895 | -0.04989544 | RecName: Full=Rotatin |
| gi341942096 | DARS | 2 | 0.965218484 | -1.036034863 | -0.051072552 | RecName: Full=Aspartyl-tRNA synthetase, cytoplasmic; AltName: Full=Aspartate--tRNA ligase; Short=AspRS |
| gi18202285 | EEF2 | 9 | 0.965209623 | -1.036044374 | -0.051085796 | RecName: Full=Elongation factor 2; Short=EF-2 |
| gi51338706 | ATP6V1B2 | 17 | 0.964794648 | -1.036489995 | -0.051706191 | RecName: Full=V-type proton ATPase subunit B, brain isoform; Short=V-ATPase subunit B 2; AltName: Full=Endomembrane proton pump 58 kDa subunit; AltName: Full=Vacuolar proton pump subunit B 2 |
| gi341941780 | UQCRC1 | 14 | 0.964335775 | -1.036983202 | -0.052392525 | RecName: Full=Cytochrome b-c1 complex subunit 1, mitochondrial; AltName: Full=Complex III subunit 1; AltName: Full=Core protein I; AltName: Full=Ubiquinol-cytochrome-c reductase complex core protein 1; Flags: Precursor |
| gi10720404 | VDAC1 | 12 | 0.964201671 | -1.037127429 | -0.052593165 | RecName: Full=Voltage-dependent anion-selective channel protein 1; Short=VDAC-1; Short=mVDAC1; AltName: Full=Outer mitochondrial membrane protein porin 1; AltName: Full=Plasmalemmal porin; AltName: Full=Voltage-dependent anion-selective channel protein 5; Short=VDAC-5; Short=mVDAC5 |
| gi50401037 | ENDOD1 | 2 | 0.963952323 | -1.037395706 | -0.052966303 | RecName: Full=Endonuclease domain-containing 1 protein; Flags: Precursor |
| gi47605546 | ATP5O | 5 | 0.963578305 | -1.037798376 | -0.053526183 | RecName: Full=ATP synthase subunit O, mitochondrial; AltName: Full=Oligomycin sensitivity conferral protein; Short=OSCP; Flags: Precursor |
| gi3334470 | MAG | 7 | 0.963263861 | -1.03813715 | -0.053997054 | RecName: Full=Myelin-associated glycoprotein; AltName: Full=Siglec-4a; Flags: Precursor |
| gi22096313 | GRIA2 | 2 | 0.96302451 | -1.038395171 | -0.054355579 | RecName: Full=Glutamate receptor 2; Short=GluR-2; AltName: Full=AMPA-selective glutamate receptor 2; AltName: Full=GluR-B; AltName: Full=GluR-K2; AltName: Full=Glutamate receptor ionotropic, AMPA 2; Short=GluA2; Flags: Precursor |
| gi47117288 | RAP1B | 3 | 0.962891464 | -1.038538649 | -0.054554906 | RecName: Full=Ras-related protein Rap-1b; AltName: Full=GTP-binding protein smg p21B; Flags: Precursor |
| gi2495231 | HINT1 | 3 | 0.962818578 | -1.038617267 | -0.054664116 | RecName: Full=Histidine triad nucleotide-binding protein 1; AltName: Full=Adenosine 5'-monophosphoramidase; AltName: Full=Protein kinase C inhibitor 1; AltName: Full=Protein kinase C-interacting protein 1; Short=PKCI-1 |
| gi464506 | PC | 7 | 0.962554952 | -1.038901725 | -0.055059188 | RecName: Full=Pyruvate carboxylase, mitochondrial; AltName: Full=Pyruvic carboxylase; Short=PCB; Flags: Precursor |
| gi42559891 | SRCIN1 | 3 | 0.961959546 | -1.039544755 | -0.055951871 | RecName: Full=SRC kinase signaling inhibitor 1; AltName: Full=SNAP-25-interacting protein; Short=SNIP; AltName: Full=p130Cas-associated protein; AltName: Full=p140Cap |
| gi51702252 | HSPD1 | 14 | 0.961487965 | -1.04005462 | -0.056659296 | RecName: Full=60 kDa heat shock protein, mitochondrial; AltName: Full=60 kDa chaperonin; AltName: Full=Chaperonin 60; Short=CPN60; AltName: Full=HSP-65; AltName: Full=Heat shock protein 60; Short=HSP-60; Short=Hsp60; AltName: Full=Mitochondrial matrix protein P1; Flags: Precursor |
| gi81906185 | APOO | 2 | 0.961368829 | -1.040183507 | -0.056838068 | RecName: Full=Apolipoprotein O; AltName: Full=Protein FAM121B |
| gi81882085 | MORC1 | 2 | 0.961228228 | -1.040335657 | -0.057049078 | RecName: Full=MORC family CW-type zinc finger protein 1; AltName: Full=Protein microrchidia |
| gi81879780 | SNCB | 6 | 0.960890235 | -1.040701595 | -0.057556457 | RecName: Full=Beta-synuclein |
| gi47117658 | ARF3 | 7 | 0.96031749 | -1.041322282 | -0.058416641 | RecName: Full=ADP-ribosylation factor 3 |
| gi51316977 | AP2M1 | 9 | 0.959915304 | -1.041758576 | -0.059020976 | RecName: Full=AP-2 complex subunit mu; AltName: Full=AP-2 mu chain; AltName: Full=Adapter-related protein complex 2 mu subunit; AltName: Full=Adaptor protein complex AP-2 subunit mu; AltName: Full=Clathrin assembly protein complex 2 medium chain; AltName: Full=Clathrin coat assembly protein AP50; AltName: Full=Clathrin coat-associated protein AP50; AltName: Full=Mu2-adaptin; AltName: Full=Plasma membrane adaptor AP-2 50 kDa protein |
| gi2497501 | DLG4 | 3 | 0.959753983 | -1.041933681 | -0.059263453 | RecName: Full=Disks large homolog 4; AltName: Full=Postsynaptic density protein 95; Short=PSD-95; AltName: Full=Synapse-associated protein 90; Short=SAP-90; Short=SAP90 |
| gi27734459 | RALB | 2 | 0.959622295 | -1.042076665 | -0.059461419 | RecName: Full=Ras-related protein Ral-B; Flags: Precursor |
| gi46396655 | SET | 4 | 0.959411636 | -1.042305475 | -0.059778159 | RecName: Full=Protein SET; AltName: Full=Phosphatase 2A inhibitor I2PP2A; Short=I-2PP2A; AltName: Full=Template-activating factor I; Short=TAF-I |
| gi121747 | GSTP1 (includes others)* | 4 | 0.959052607 | -1.042695669 | -0.060318141 | RecName: Full=Glutathione S-transferase P 1; Short=Gst P1; AltName: Full=GST YF-YF; AltName: Full=GST class-pi; AltName: Full=GST-piB; AltName: Full=Preadipocyte growth factor |
| gi3122030 | CRMP1 | 12 | 0.959007009 | -1.042745247 | -0.060386736 | RecName: Full=Dihydropyrimidinase-related protein 1; Short=DRP-1; AltName: Full=Collapsin response mediator protein 1; Short=CRMP-1; AltName: Full=Unc-33-like phosphoprotein 3; Short=ULIP-3 |
| gi341941159 | NDUFA9 | 6 | 0.958825991 | -1.042942108 | -0.060659078 | RecName: Full=NADH dehydrogenase [ubiquinone] 1 alpha subcomplex subunit 9, mitochondrial; AltName: Full=Complex I-39kD; Short=CI-39kD; AltName: Full=NADH-ubiquinone oxidoreductase 39 kDa subunit; Flags: Precursor |
| gi46397808 | CORO1A | 2 | 0.958816964 | -1.042951927 | -0.060672661 | RecName: Full=Coronin-1A; AltName: Full=Coronin-like protein A; Short=Clipin-A; AltName: Full=Coronin-like protein p57; AltName: Full=Tryptophan aspartate-containing coat protein; Short=TACO |
| gi30316333 | GPM6B | 4 | 0.958778409 | -1.042993866 | -0.060730674 | RecName: Full=Neuronal membrane glycoprotein M6-b; Short=M6b |
| gi81862737 | ATP13A4 | 2 | 0.958035749 | -1.043802385 | -0.061848603 | RecName: Full=Probable cation-transporting ATPase 13A4; AltName: Full=P5-ATPase isoform 4 |
| gi52000687 | ATP1A3 (includes EG:232975) | 45 | 0.957641338 | -1.044232282 | -0.062442665 | RecName: Full=Sodium/potassium-transporting ATPase subunit alpha-3; Short=Na(+)/K(+) ATPase alpha-3 subunit; AltName: Full=Na(+)/K(+) ATPase alpha(III) subunit; AltName: Full=Sodium pump subunit alpha-3 |
| gi68566157 | YWHAQ | 11 | 0.957496112 | -1.044390664 | -0.062661466 | RecName: Full=14-3-3 protein theta; AltName: Full=14-3-3 protein tau |
| gi13638207 | PFKM | 11 | 0.957470883 | -1.044418183 | -0.06269948 | RecName: Full=6-phosphofructokinase, muscle type; AltName: Full=Phosphofructo-1-kinase isozyme A; Short=PFK-A; Short=Phosphofructokinase-M; AltName: Full=Phosphofructokinase 1; AltName: Full=Phosphohexokinase |
| gi298286902 | NPTN | 6 | 0.956983391 | -1.044950214 | -0.063434208 | RecName: Full=Neuroplastin; AltName: Full=Stromal cell-derived receptor 1; Short=SDR-1; Flags: Precursor |
| gi1345668 | CAPZB | 2 | 0.956893368 | -1.045048522 | -0.063569929 | RecName: Full=F-actin-capping protein subunit beta; AltName: Full=CapZ beta |
| gi115395 | CALB1 | 3 | 0.955354197 | -1.0467322 | -0.065892385 | RecName: Full=Calbindin; AltName: Full=Calbindin D28; AltName: Full=D-28K; AltName: Full=PCD-29; AltName: Full=Spot 35 protein; AltName: Full=Vitamin D-dependent calcium-binding protein, avian-type |
| gi21362640 | GLO1 | 4 | 0.955258072 | -1.046837529 | -0.066037551 | RecName: Full=Lactoylglutathione lyase; AltName: Full=Aldoketomutase; AltName: Full=Glyoxalase I; Short=Glx I; AltName: Full=Ketone-aldehyde mutase; AltName: Full=Methylglyoxalase; AltName: Full=S-D-lactoylglutathione methylglyoxal lyase |
| gi116961 | Serpina3k (includes others) | 2 | 0.954931952 | -1.047195036 | -0.066530163 | RecName: Full=Serine protease inhibitor A3K; Short=Serpin A3K; AltName: Full=Contrapsin; AltName: Full=SPI-2; Flags: Precursor |
| gi20138079 | EPB41L3 | 8 | 0.95460181 | -1.0475572 | -0.067029022 | RecName: Full=Band 4.1-like protein 3; AltName: Full=4.1B; AltName: Full=Differentially expressed in adenocarcinoma of the lung protein 1; Short=DAL-1; Short=DAL1P; Short=mDAL-1 |
| gi730956 | TKT | 6 | 0.954546555 | -1.04761784 | -0.067112533 | RecName: Full=Transketolase; Short=TK; AltName: Full=P68 |
| gi45477158 | ACSL6 | 4 | 0.954511072 | -1.047656784 | -0.067166162 | RecName: Full=Long-chain-fatty-acid--CoA ligase 6; AltName: Full=Long-chain acyl-CoA synthetase 6; Short=LACS 6 |
| gi341941270 | PLCB1 | 6 | 0.954419573 | -1.047757222 | -0.067304465 | RecName: Full=1-phosphatidylinositol-4,5-bisphosphate phosphodiesterase beta-1; AltName: Full=PLC-154; AltName: Full=Phosphoinositide phospholipase C-beta-1; AltName: Full=Phospholipase C-beta-1; Short=PLC-beta-1 |
| gi51338599 | RAN | 4 | 0.954398882 | -1.047779936 | -0.067335741 | RecName: Full=GTP-binding nuclear protein Ran; AltName: Full=GTPase Ran; AltName: Full=Ras-like protein TC4; AltName: Full=Ras-related nuclear protein |
| gi147744591 | SEPT3 | 3 | 0.95433874 | -1.047845967 | -0.067426657 | RecName: Full=Neuronal-specific septin-3 |
| gi46397704 | Rps20 | 2 | 0.953757325 | -1.048484739 | -0.068305863 | RecName: Full=40S ribosomal protein S20 |
| gi71153505 | DHX9 | 2 | 0.953022603 | -1.049293057 | -0.069417664 | RecName: Full=ATP-dependent RNA helicase A; AltName: Full=DEAH box protein 9; Short=mHEL-5; AltName: Full=Nuclear DNA helicase II; Short=NDH II |
| gi46395611 | ANP32A | 3 | 0.95221898 | -1.050178604 | -0.070634709 | RecName: Full=Acidic leucine-rich nuclear phosphoprotein 32 family member A; AltName: Full=Acidic nuclear phosphoprotein pp32; AltName: Full=Leucine-rich acidic nuclear protein; Short=LANP; AltName: Full=Potent heat-stable protein phosphatase 2A inhibitor I1PP2A |
| gi52783422 | SLC38A3 | 2 | 0.95178486 | -1.050657603 | -0.071292589 | RecName: Full=Sodium-coupled neutral amino acid transporter 3; AltName: Full=N-system amino acid transporter 1; AltName: Full=Na(+)-coupled neutral amino acid transporter 3; AltName: Full=Solute carrier family 38 member 3; Short=mNAT; AltName: Full=System N amino acid transporter 1 |
| gi138536 | VIM | 4 | 0.951546233 | -1.050921086 | -0.07165434 | RecName: Full=Vimentin |
| gi54036535 | SKP1/SKP1P2 | 5 | 0.950495629 | -1.052082692 | -0.073248103 | RecName: Full=S-phase kinase-associated protein 1; AltName: Full=Cyclin-A/CDK2-associated protein p19; AltName: Full=S-phase kinase-associated protein 1A; AltName: Full=p19A; AltName: Full=p19skp1 |
| gi115311320 | CAMKV | 9 | 0.950476342 | -1.05210404 | -0.073277377 | RecName: Full=CaM kinase-like vesicle-associated protein |
| gi94730353 | INA | 21 | 0.949705439 | -1.052958064 | -0.074447979 | RecName: Full=Alpha-internexin; Short=Alpha-Inx; AltName: Full=66 kDa neurofilament protein; Short=NF-66; Short=Neurofilament-66 |
| gi81881592 | ARPC5L | 2 | 0.949059113 | -1.053675147 | -0.075430146 | RecName: Full=Actin-related protein 2/3 complex subunit 5-like protein; AltName: Full=Arp2/3 complex 16 kDa subunit 2; Short=ARC16-2 |
| gi3024089 | LASP1 | 2 | 0.948752487 | -1.054015682 | -0.075896333 | RecName: Full=LIM and SH3 domain protein 1; Short=LASP-1; AltName: Full=Metastatic lymph node gene 50 protein; Short=MLN 50 |
| gi145559476 | GLUL | 11 | 0.94786077 | -1.055007267 | -0.077252936 | RecName: Full=Glutamine synthetase; Short=GS; AltName: Full=Glutamate decarboxylase; AltName: Full=Glutamate--ammonia ligase |
| gi51701351 | AP2B1 | 16 | 0.946680556 | -1.056322529 | -0.079050403 | RecName: Full=AP-2 complex subunit beta; AltName: Full=AP105B; AltName: Full=Adapter-related protein complex 2 beta subunit; AltName: Full=Adaptor protein complex AP-2 subunit beta; AltName: Full=Beta-2-adaptin; AltName: Full=Beta-adaptin; AltName: Full=Clathrin assembly protein complex 2 beta large chain; AltName: Full=Plasma membrane adaptor HA2/AP2 adaptin beta subunit |
| gi14285350 | ATP2B2 | 12 | 0.946505139 | -1.0565183 | -0.079317756 | RecName: Full=Plasma membrane calcium-transporting ATPase 2; Short=PMCA2; AltName: Full=Plasma membrane calcium ATPase isoform 2; AltName: Full=Plasma membrane calcium pump isoform 2 |
| gi21759130 | ARHGDIA | 4 | 0.946275503 | -1.056774689 | -0.079667817 | RecName: Full=Rho GDP-dissociation inhibitor 1; Short=Rho GDI 1; AltName: Full=GDI-1; AltName: Full=Rho-GDI alpha |
| gi47117273 | NDUFS2 | 5 | 0.9459288 | -1.057162018 | -0.080196498 | RecName: Full=NADH dehydrogenase [ubiquinone] iron-sulfur protein 2, mitochondrial; AltName: Full=Complex I-49kD; Short=CI-49kD; AltName: Full=NADH-ubiquinone oxidoreductase 49 kDa subunit; Flags: Precursor |
| gi150438864 | CADM2 | 5 | 0.945670675 | -1.057450576 | -0.080590235 | RecName: Full=Cell adhesion molecule 2; AltName: Full=Immunoglobulin superfamily member 4D; Short=IgSF4D; AltName: Full=Nectin-like protein 3; Short=NECL-3; Flags: Precursor |
| gi548409 | PDHA1 | 9 | 0.945162096 | -1.058019575 | -0.08136632 | RecName: Full=Pyruvate dehydrogenase E1 component subunit alpha, somatic form, mitochondrial; AltName: Full=PDHE1-A type I; Flags: Precursor |
| gi1170151 | HIST1H1E | 4 | 0.944940798 | -1.058267356 | -0.08170415 | RecName: Full=Histone H1.4; AltName: Full=H1 VAR.2; AltName: Full=H1e |
| gi21431839 | RPH3A | 3 | 0.944440465 | -1.058827991 | -0.08246824 | RecName: Full=Rabphilin-3A; AltName: Full=Exophilin-1 |
| gi20141252 | ATP5J2 | 2 | 0.944379002 | -1.058896903 | -0.082562131 | RecName: Full=ATP synthase subunit f, mitochondrial |
| gi81912821 | GSTM2 | 4 | 0.943846744 | -1.059494041 | -0.083375473 | RecName: Full=Glutathione S-transferase Mu 7; AltName: Full=GST class-mu 7; Short=GSTM-7 |
| gi41019154 | PLP1 (includes EG:18823) | 8 | 0.943636305 | -1.059730316 | -0.083697169 | RecName: Full=Myelin proteolipid protein; Short=PLP; AltName: Full=Lipophilin |
| gi94730370 | CTNNA2 | 2 | 0.943482102 | -1.059903519 | -0.083932945 | RecName: Full=Catenin alpha-2; AltName: Full=Alpha N-catenin |
| gi116918 | CNTN1 | 16 | 0.943353807 | -1.060047664 | -0.084129136 | RecName: Full=Contactin-1; AltName: Full=Neural cell surface protein F3; Flags: Precursor |
| gi1169460 | SLC1A2 | 13 | 0.943253235 | -1.06016069 | -0.084282953 | RecName: Full=Excitatory amino acid transporter 2; AltName: Full=GLT-1; AltName: Full=Sodium-dependent glutamate/aspartate transporter 2; AltName: Full=Solute carrier family 1 member 2 |
| gi32363396 | NDUFA2 | 2 | 0.942722735 | -1.060757276 | -0.085094574 | RecName: Full=NADH dehydrogenase [ubiquinone] 1 alpha subcomplex subunit 2; AltName: Full=Complex I-B8; Short=CI-B8; AltName: Full=NADH-ubiquinone oxidoreductase B8 subunit |
| gi126042 | LDHB | 14 | 0.942300466 | -1.061232628 | -0.085740938 | RecName: Full=L-lactate dehydrogenase B chain; Short=LDH-B; AltName: Full=LDH heart subunit; Short=LDH-H |
| gi92087001 | MDH1 | 13 | 0.941486374 | -1.062150264 | -0.08698788 | RecName: Full=Malate dehydrogenase, cytoplasmic; AltName: Full=Cytosolic malate dehydrogenase |
| gi110825706 | ARPC2 | 3 | 0.941373526 | -1.062277589 | -0.087160814 | RecName: Full=Actin-related protein 2/3 complex subunit 2; AltName: Full=Arp2/3 complex 34 kDa subunit; Short=p34-ARC |
| gi68565118 | CNRIP1 | 2 | 0.93925325 | -1.064675581 | -0.090413891 | RecName: Full=CB1 cannabinoid receptor-interacting protein 1; Short=CRIP-1 |
| gi41688724 | MRPS36 | 4 | 0.939151538 | -1.064790888 | -0.090570131 | RecName: Full=28S ribosomal protein S36, mitochondrial; Short=MRP-S36; Short=S36mt |
| gi417208 | CKB | 20 | 0.938983183 | -1.0649818 | -0.090828776 | RecName: Full=Creatine kinase B-type; AltName: Full=B-CK; AltName: Full=Creatine kinase B chain |
| gi146345481 | PGK1 | 16 | 0.938901203 | -1.065074789 | -0.090954739 | RecName: Full=Phosphoglycerate kinase 1 |
| gi49037483 | CALM | 7 | 0.938585398 | -1.065433153 | -0.091440079 | RecName: Full=Calmodulin; Short=CaM |
| gi25089776 | ATP5H (includes EG:100039281) | 8 | 0.938391934 | -1.065652809 | -0.091737483 | RecName: Full=ATP synthase subunit d, mitochondrial; Short=ATPase subunit d |
| gi1170099 | GSTP1 (includes others)* | 3 | 0.937360204 | -1.066825747 | -0.093324549 | RecName: Full=Glutathione S-transferase P 2; Short=Gst P2; AltName: Full=GST YF-YF; AltName: Full=GST class-pi; AltName: Full=GST-piA |
| gi14548302 | UQCRC2 | 15 | 0.937130769 | -1.067086935 | -0.093677716 | RecName: Full=Cytochrome b-c1 complex subunit 2, mitochondrial; AltName: Full=Complex III subunit 2; AltName: Full=Core protein II; AltName: Full=Ubiquinol-cytochrome-c reductase complex core protein 2; Flags: Precursor |
| gi146345428 | GPD2 | 9 | 0.936166293 | -1.06818629 | -0.095163273 | RecName: Full=Glycerol-3-phosphate dehydrogenase, mitochondrial; Short=GPD-M; Short=GPDH-M; AltName: Full=Protein TISP38; Flags: Precursor |
| gi1709737 | PITPNA | 3 | 0.935714567 | -1.068701968 | -0.095859581 | RecName: Full=Phosphatidylinositol transfer protein alpha isoform; Short=PI-TP-alpha; Short=PtdIns transfer protein alpha; Short=PtdInsTP alpha |
| gi2493662 | HSPE1 | 7 | 0.935635077 | -1.068792764 | -0.095982146 | RecName: Full=10 kDa heat shock protein, mitochondrial; Short=Hsp10; AltName: Full=10 kDa chaperonin; AltName: Full=Chaperonin 10; Short=CPN10 |
| gi302595876 | UBB | 7 | 0.935437931 | -1.069018015 | -0.096286165 | RecName: Full=Polyubiquitin-B; Contains: RecName: Full=Ubiquitin; Flags: Precursor |
| gi267190 | UBA1 | 14 | 0.935048814 | -1.069462883 | -0.096886413 | RecName: Full=Ubiquitin-like modifier-activating enzyme 1; AltName: Full=Ubiquitin-activating enzyme E1; AltName: Full=Ubiquitin-activating enzyme E1 X; AltName: Full=Ubiquitin-like modifier-activating enzyme 1 X |
| gi55584180 | PHGDH | 3 | 0.934585911 | -1.069992591 | -0.097600807 | RecName: Full=D-3-phosphoglycerate dehydrogenase; Short=3-PGDH; AltName: Full=A10 |
| gi341940828 | KPNB1 | 5 | 0.934376032 | -1.070232931 | -0.097924827 | RecName: Full=Importin subunit beta-1; AltName: Full=Karyopherin subunit beta-1; AltName: Full=Nuclear factor p97; AltName: Full=Pore targeting complex 97 kDa subunit; Short=PTAC97; AltName: Full=SCG |
| gi122065442 | MAP1A | 21 | 0.934158869 | -1.070481728 | -0.098260171 | RecName: Full=Microtubule-associated protein 1A; Short=MAP-1A; Contains: RecName: Full=MAP1 light chain LC2 |
| gi81879424 | HNRNPU | 5 | 0.933994573 | -1.070670033 | -0.098513928 | RecName: Full=Heterogeneous nuclear ribonucleoprotein U; Short=hnRNP U; AltName: Full=Scaffold attachment factor A; Short=SAF-A |
| gi462602 | MIF | 3 | 0.93313234 | -1.071659353 | -0.099846391 | RecName: Full=Macrophage migration inhibitory factor; Short=MIF; AltName: Full=Delayed early response protein 6; Short=DER6; AltName: Full=Glycosylation-inhibiting factor; Short=GIF; AltName: Full=L-dopachrome isomerase; AltName: Full=L-dopachrome tautomerase; AltName: Full=Phenylpyruvate tautomerase |
| gi251757493 | GNAQ | 2 | 0.933071917 | -1.071728751 | -0.099939813 | RecName: Full=Guanine nucleotide-binding protein G(q) subunit alpha; AltName: Full=Guanine nucleotide-binding protein alpha-q |
| gi547923 | PRDX1 | 8 | 0.932958159 | -1.07185943 | -0.100115714 | RecName: Full=Peroxiredoxin-1; AltName: Full=Macrophage 23 kDa stress protein; AltName: Full=Osteoblast-specific factor 3; Short=OSF-3; AltName: Full=Thioredoxin peroxidase 2; AltName: Full=Thioredoxin-dependent peroxide reductase 2 |
| gi47117909 | RPL15 | 2 | 0.932927881 | -1.071894217 | -0.100162536 | RecName: Full=60S ribosomal protein L15 |
| gi81885886 | NAP1L4 | 2 | 0.932117417 | -1.072826214 | -0.101416395 | RecName: Full=Nucleosome assembly protein 1-like 4 |
| gi54039315 | RPS10 | 2 | 0.931716135 | -1.073288271 | -0.102037617 | RecName: Full=40S ribosomal protein S10 |
| gi1711560 | STMN1 | 4 | 0.931346474 | -1.07371427 | -0.102610124 | RecName: Full=Stathmin; AltName: Full=Leukemia-associated gene protein; AltName: Full=Leukemia-associated phosphoprotein p18; AltName: Full=Metablastin; AltName: Full=Oncoprotein 18; Short=Op18; AltName: Full=Phosphoprotein p19; Short=pp19; AltName: Full=Prosolin; AltName: Full=Protein Pr22; AltName: Full=pp17 |
| gi46396509 | PDHB | 7 | 0.930864399 | -1.074270324 | -0.103357072 | RecName: Full=Pyruvate dehydrogenase E1 component subunit beta, mitochondrial; Short=PDHE1-B; Flags: Precursor |
| gi341941247 | BCAN | 3 | 0.930669242 | -1.074495594 | -0.103659567 | RecName: Full=Brevican core protein; Flags: Precursor |
| gi108935937 | TCEAL3 | 3 | 0.93043623 | -1.074764683 | -0.104020821 | RecName: Full=Transcription elongation factor A protein-like 3; Short=TCEA-like protein 3; AltName: Full=Transcription elongation factor S-II protein-like 3 |
| gi30913117 | SCRN1 | 5 | 0.929785066 | -1.075517382 | -0.105030842 | RecName: Full=Secernin-1 |
| gi93139504 | ADSS | 2 | 0.929121114 | -1.076285949 | -0.106061426 | RecName: Full=Adenylosuccinate synthetase isozyme 2; Short=AMPSase 2; Short=AdSS 2; AltName: Full=Adenylosuccinate synthetase, acidic isozyme; AltName: Full=Adenylosuccinate synthetase, liver isozyme; Short=L-type adenylosuccinate synthetase; AltName: Full=IMP--aspartate ligase 2 |
| gi57012952 | PDHX | 2 | 0.928620895 | -1.07686571 | -0.106838351 | RecName: Full=Pyruvate dehydrogenase protein X component, mitochondrial; AltName: Full=Dihydrolipoamide dehydrogenase-binding protein of pyruvate dehydrogenase complex; AltName: Full=Lipoyl-containing pyruvate dehydrogenase complex component X; Flags: Precursor |
| gi60391212 | ACO2 (includes EG:11429) | 22 | 0.928257859 | -1.077286866 | -0.10740247 | RecName: Full=Aconitate hydratase, mitochondrial; Short=Aconitase; AltName: Full=Citrate hydro-lyase; Flags: Precursor |
| gi20138723 | SLC25A11 | 2 | 0.927524772 | -1.078138321 | -0.108542282 | RecName: Full=Mitochondrial 2-oxoglutarate/malate carrier protein; Short=OGCP; AltName: Full=Solute carrier family 25 member 11 |
| gi59797879 | DLG2 | 2 | 0.926077981 | -1.079822673 | -0.110794414 | RecName: Full=Disks large homolog 2; AltName: Full=Channel-associated protein of synapse-110; Short=Chapsyn-110; AltName: Full=Postsynaptic density protein PSD-93 |
| gi2829481 | IDH3G | 4 | 0.926052569 | -1.079852303 | -0.110834001 | RecName: Full=Isocitrate dehydrogenase [NAD] subunit gamma 1, mitochondrial; AltName: Full=Isocitric dehydrogenase subunit gamma; AltName: Full=NAD(+)-specific ICDH subunit gamma; Flags: Precursor |
| gi62510833 | DLST | 3 | 0.925350502 | -1.080671592 | -0.111928166 | RecName: Full=Dihydrolipoyllysine-residue succinyltransferase component of 2-oxoglutarate dehydrogenase complex, mitochondrial; AltName: Full=2-oxoglutarate dehydrogenase complex component E2; Short=OGDC-E2; AltName: Full=Dihydrolipoamide succinyltransferase component of 2-oxoglutarate dehydrogenase complex; AltName: Full=E2K; Flags: Precursor |
| gi48474314 | RAB5B | 2 | 0.924438615 | -1.08173759 | -0.11335057 | RecName: Full=Ras-related protein Rab-5B |
| gi20140237 | EFHD2 | 3 | 0.923061468 | -1.083351472 | -0.115501373 | RecName: Full=EF-hand domain-containing protein D2; AltName: Full=Swiprosin-1 |
| gi47117291 | NDUFS5 | 2 | 0.922834045 | -1.083618453 | -0.115856866 | RecName: Full=NADH dehydrogenase [ubiquinone] iron-sulfur protein 5; AltName: Full=Complex I-15 kDa; Short=CI-15 kDa; AltName: Full=NADH-ubiquinone oxidoreductase 15 kDa subunit |
| gi55976655 | YWHAH | 10 | 0.92130306 | -1.085419167 | -0.118252289 | RecName: Full=14-3-3 protein eta |
| gi338817898 | GOT1 | 13 | 0.920379629 | -1.086508185 | -0.119699042 | RecName: Full=Aspartate aminotransferase, cytoplasmic; AltName: Full=Glutamate oxaloacetate transaminase 1; AltName: Full=Transaminase A |
| gi55583934 | TXLNG | 2 | 0.920185047 | -1.086737937 | -0.120004082 | RecName: Full=Gamma-taxilin; AltName: Full=Factor inhibiting ATF4-mediated transcription; Short=FIAT; AltName: Full=Lipopolysaccharide-responsive gene protein |
| gi347595817 | PRKCB | 3 | 0.919283056 | -1.087804233 | -0.121418945 | RecName: Full=Protein kinase C beta type; Short=PKC-B; Short=PKC-beta |
| gi146345463 | NDUFV2 | 2 | 0.918878957 | -1.088282621 | -0.122053266 | RecName: Full=NADH dehydrogenase [ubiquinone] flavoprotein 2, mitochondrial; AltName: Full=NADH-ubiquinone oxidoreductase 24 kDa subunit; Flags: Precursor |
| gi12585446 | ATP6V1D (includes EG:299159) | 2 | 0.917307683 | -1.090146762 | -0.124522372 | RecName: Full=V-type proton ATPase subunit D; Short=V-ATPase subunit D; AltName: Full=V-ATPase 28 kDa accessory protein; AltName: Full=Vacuolar proton pump subunit D |
| gi2497313 | MOG | 5 | 0.917268343 | -1.090193516 | -0.124584245 | RecName: Full=Myelin-oligodendrocyte glycoprotein; Flags: Precursor |
| gi341940933 | MAP1B | 9 | 0.916569916 | -1.091024244 | -0.125683161 | RecName: Full=Microtubule-associated protein 1B; Short=MAP-1B; AltName: Full=MAP1(X); AltName: Full=MAP1.2; Contains: RecName: Full=MAP1 light chain LC1 |
| gi37999666 | ATL1 | 2 | 0.916498062 | -1.091109781 | -0.125796265 | RecName: Full=Atlastin-1; AltName: Full=Spastic paraplegia 3A homolog |
| gi146345448 | PKM2 | 27 | 0.916394347 | -1.09123327 | -0.125959535 | RecName: Full=Pyruvate kinase isozymes M1/M2; AltName: Full=Pyruvate kinase muscle isozyme |
| gi28376965 | ACOT7 | 4 | 0.916286711 | -1.091361457 | -0.126128999 | RecName: Full=Cytosolic acyl coenzyme A thioester hydrolase; AltName: Full=Acyl-CoA thioesterase 7; AltName: Full=Brain acyl-CoA hydrolase; Short=BACH; AltName: Full=CTE-IIa; Short=CTE-II; AltName: Full=Long chain acyl-CoA thioester hydrolase |
| gi41016844 | AMPH | 13 | 0.915836534 | -1.091897913 | -0.126837978 | RecName: Full=Amphiphysin |
| gi205830863 | MAP6 | 10 | 0.914409656 | -1.093601751 | -0.129087457 | RecName: Full=Microtubule-associated protein 6; Short=MAP-6; AltName: Full=Stable tubule-only polypeptide; Short=STOP |
| gi17432986 | EPB41L2 | 4 | 0.914223899 | -1.093823953 | -0.129380561 | RecName: Full=Band 4.1-like protein 2; AltName: Full=Generally expressed protein 4.1; Short=4.1G |
| gi29427844 | NLRP3 | 2 | 0.910928582 | -1.097780901 | -0.134590145 | RecName: Full=NACHT, LRR and PYD domains-containing protein 3; AltName: Full=Cold autoinflammatory syndrome 1 protein homolog; AltName: Full=Cryopyrin; AltName: Full=Mast cell maturation-associated-inducible protein 1; AltName: Full=PYRIN-containing APAF1-like protein 1 |
| gi160406731 | SH3GL2 | 11 | 0.910829583 | -1.09790022 | -0.134746945 | RecName: Full=Endophilin-A1; AltName: Full=Endophilin-1; AltName: Full=SH3 domain protein 2A; AltName: Full=SH3 domain-containing GRB2-like protein 2; AltName: Full=SH3p4 |
| gi20141407 | SLC1A3 | 4 | 0.910024514 | -1.098871497 | -0.136022687 | RecName: Full=Excitatory amino acid transporter 1; AltName: Full=Glial high affinity glutamate transporter; AltName: Full=High-affinity neuronal glutamate transporter; AltName: Full=Sodium-dependent glutamate/aspartate transporter 1; Short=GLAST-1; AltName: Full=Solute carrier family 1 member 3 |
| gi59797853 | DLG1 | 2 | 0.90779237 | -1.101573479 | -0.139565732 | RecName: Full=Disks large homolog 1; AltName: Full=Embryo-dlg/synapse-associated protein 97; Short=E-dlg/SAP97; AltName: Full=Synapse-associated protein 97; Short=SAP-97; Short=SAP97 |
| gi341942279 | EPRS | 2 | 0.907752249 | -1.101622168 | -0.139629496 | RecName: Full=Bifunctional aminoacyl-tRNA synthetase; Includes: RecName: Full=Glutamyl-tRNA synthetase; AltName: Full=Glutamate--tRNA ligase; Short=GluRS; Includes: RecName: Full=Prolyl-tRNA synthetase; AltName: Full=Proline--tRNA ligase; Short=ProRS |
| gi67460396 | TUFM | 6 | 0.901727513 | -1.108982464 | -0.149236553 | RecName: Full=Elongation factor Tu, mitochondrial; Flags: Precursor |
| gi47117859 | AQP4 | 2 | 0.900396908 | -1.110621317 | -0.151366992 | RecName: Full=Aquaporin-4; Short=AQP-4; AltName: Full=Mercurial-insensitive water channel; Short=MIWC; AltName: Full=WCH4 |
| gi41017503 | NSFL1C | 2 | 0.899968074 | -1.111150528 | -0.152054272 | RecName: Full=NSFL1 cofactor p47; AltName: Full=p97 cofactor p47 |
| gi48428679 | OXCT1 | 3 | 0.899614729 | -1.111586958 | -0.152620813 | RecName: Full=Succinyl-CoA:3-ketoacid-coenzyme A transferase 1, mitochondrial; AltName: Full=3-oxoacid-CoA transferase 1; AltName: Full=Somatic-type succinyl-CoA:3-oxoacid CoA-transferase; Short=SCOT-s; Flags: Precursor |
| gi400275 | MAP2K1 | 4 | 0.898966538 | -1.112388457 | -0.153660679 | RecName: Full=Dual specificity mitogen-activated protein kinase kinase 1; Short=MAP kinase kinase 1; Short=MAPKK 1; AltName: Full=ERK activator kinase 1; AltName: Full=MAPK/ERK kinase 1; Short=MEK 1 |
| gi38372626 | ARPC4 | 2 | 0.898720549 | -1.112692929 | -0.154055505 | RecName: Full=Actin-related protein 2/3 complex subunit 4; AltName: Full=Arp2/3 complex 20 kDa subunit; Short=p20-ARC |
| gi146345468 | NEFM | 20 | 0.898523674 | -1.112936731 | -0.154371579 | RecName: Full=Neurofilament medium polypeptide; Short=NF-M; AltName: Full=160 kDa neurofilament protein; AltName: Full=Neurofilament 3; AltName: Full=Neurofilament triplet M protein |
| gi147644461 | GPD1L | 2 | 0.89796618 | -1.113627688 | -0.155266986 | RecName: Full=Glycerol-3-phosphate dehydrogenase 1-like protein |
| gi67460420 | CDS2 | 2 | 0.896199295 | -1.115823239 | -0.158108504 | RecName: Full=Phosphatidate cytidylyltransferase 2; AltName: Full=CDP-DAG synthase 2; AltName: Full=CDP-DG synthase 2; AltName: Full=CDP-diacylglycerol synthase 2; Short=CDS 2; AltName: Full=CDP-diglyceride pyrophosphorylase 2; AltName: Full=CDP-diglyceride synthase 2; AltName: Full=CTP:phosphatidate cytidylyltransferase 2 |
| gi20140196 | PSAT1 | 3 | 0.895535864 | -1.116649863 | -0.159176885 | RecName: Full=Phosphoserine aminotransferase; Short=PSAT; AltName: Full=Endometrial progesterone-induced protein; Short=EPIP; AltName: Full=Phosphohydroxythreonine aminotransferase |
| gi13637776 | ENO1 | 23 | 0.894921007 | -1.117417059 | -0.16016775 | RecName: Full=Alpha-enolase; AltName: Full=2-phospho-D-glycerate hydro-lyase; AltName: Full=Enolase 1; AltName: Full=Non-neural enolase; Short=NNE |
| gi146345384 | PPP3R1 | 7 | 0.894662818 | -1.117739533 | -0.160584036 | RecName: Full=Calcineurin subunit B type 1; AltName: Full=Protein phosphatase 2B regulatory subunit 1; AltName: Full=Protein phosphatase 3 regulatory subunit B alpha isoform 1 |
| gi158931128 | KIF2A | 2 | 0.891586245 | -1.121596487 | -0.165553736 | RecName: Full=Kinesin-like protein KIF2A; AltName: Full=Kinesin-2 |
| gi56405010 | Eef1a1 | 6 | 0.89017664 | -1.123372548 | -0.167836453 | RecName: Full=Elongation factor 1-alpha 1; Short=EF-1-alpha-1; AltName: Full=Elongation factor Tu; Short=EF-Tu; AltName: Full=Eukaryotic elongation factor 1 A-1; Short=eEF1A-1 |
| gi13432200 | MAPT | 6 | 0.886398902 | -1.128160242 | -0.173972001 | RecName: Full=Microtubule-associated protein tau; AltName: Full=Neurofibrillary tangle protein; AltName: Full=Paired helical filament-tau; Short=PHF-tau |
| gi334305788 | GNAI1 | 4 | 0.886068892 | -1.128580418 | -0.174509223 | RecName: Full=Guanine nucleotide-binding protein G(i) subunit alpha-1; AltName: Full=Adenylate cyclase-inhibiting G alpha protein |
| gi52001083 | PCP4 | 3 | 0.885092492 | -1.129825424 | -0.176099871 | RecName: Full=Purkinje cell protein 4; AltName: Full=Brain-specific antigen PCP-4; AltName: Full=Brain-specific polypeptide PEP-19 |
| gi46577661 | UBE2N | 4 | 0.885079972 | -1.129841407 | -0.176120279 | RecName: Full=Ubiquitin-conjugating enzyme E2 N; AltName: Full=Bendless-like ubiquitin-conjugating enzyme; AltName: Full=Ubc13; AltName: Full=Ubiquitin carrier protein N; AltName: Full=Ubiquitin-protein ligase N |
| gi46577330 | HNRNPH2 | 5 | 0.884784132 | -1.130219184 | -0.176602583 | RecName: Full=Heterogeneous nuclear ribonucleoprotein H2; Short=hnRNP H2; AltName: Full=Heterogeneous nuclear ribonucleoprotein H'; Short=hnRNP H' |
| gi341940932 | ME1 | 3 | 0.883593259 | -1.131742451 | -0.178545683 | RecName: Full=NADP-dependent malic enzyme; Short=NADP-ME; AltName: Full=Malic enzyme 1 |
| gi25091206 | SETDB1 | 2 | 0.880002804 | -1.136360015 | -0.184419974 | RecName: Full=Histone-lysine N-methyltransferase SETDB1; AltName: Full=ERG-associated protein with SET domain; Short=ESET; AltName: Full=SET domain bifurcated 1 |
| gi18202836 | YWHAB | 13 | 0.879622386 | -1.136851467 | -0.185043774 | RecName: Full=14-3-3 protein beta/alpha; AltName: Full=Protein kinase C inhibitor protein 1; Short=KCIP-1; Contains: RecName: Full=14-3-3 protein beta/alpha, N-terminally processed |
| gi1708288 | HPRT1 | 3 | 0.877081599 | -1.140144772 | -0.189217025 | RecName: Full=Hypoxanthine-guanine phosphoribosyltransferase; Short=HGPRT; Short=HGPRTase; AltName: Full=HPRT B |
| gi8928084 | FUS | 3 | 0.876303438 | -1.141157226 | -0.190497576 | RecName: Full=RNA-binding protein FUS; AltName: Full=Protein pigpen |
| gi51338605 | RAB3C (includes EG:115827) | 6 | 0.875813622 | -1.141795441 | -0.191304206 | RecName: Full=Ras-related protein Rab-3C |
| gi1346207 | GSTM3 | 5 | 0.875758193 | -1.141867707 | -0.191395514 | RecName: Full=Glutathione S-transferase Mu 5; AltName: Full=Fibrous sheath component 2; Short=Fsc2; AltName: Full=GST class-mu 5 |
| gi127982 | NME1 (includes EG:18102) | 6 | 0.87320142 | -1.145211147 | -0.195613618 | RecName: Full=Nucleoside diphosphate kinase A; Short=NDK A; Short=NDP kinase A; AltName: Full=Metastasis inhibition factor NM23; AltName: Full=NDPK-A; AltName: Full=Tumor metastatic process-associated protein; AltName: Full=nm23-M1 |
| gi294862498 | OXR1 (includes EG:117520) | 4 | 0.872648801 | -1.145936371 | -0.196526939 | RecName: Full=Oxidation resistance protein 1; AltName: Full=Protein C7 |
| gi81896595 | NCEH1 | 2 | 0.872032751 | -1.146745921 | -0.197545776 | RecName: Full=Neutral cholesterol ester hydrolase 1; Short=NCEH; AltName: Full=Arylacetamide deacetylase-like 1; AltName: Full=Chlorpyrifos oxon-binding protein; Short=CPO-BP |
| gi121557 | GPD1 | 2 | 0.871275755 | -1.147742255 | -0.198798697 | RecName: Full=Glycerol-3-phosphate dehydrogenase [NAD+], cytoplasmic; Short=GPD-C; Short=GPDH-C |
| gi146325018 | DLAT | 4 | 0.870508254 | -1.148754185 | -0.200070117 | RecName: Full=Dihydrolipoyllysine-residue acetyltransferase component of pyruvate dehydrogenase complex, mitochondrial; AltName: Full=Dihydrolipoamide acetyltransferase component of pyruvate dehydrogenase complex; AltName: Full=Pyruvate dehydrogenase complex component E2; Short=PDC-E2; Short=PDCE2; Flags: Precursor |
| gi146345383 | CA2 | 8 | 0.87011429 | -1.149274309 | -0.200723182 | RecName: Full=Carbonic anhydrase 2; AltName: Full=Carbonate dehydratase II; AltName: Full=Carbonic anhydrase II; Short=CA-II |
| gi56748875 | DYNLL2 | 2 | 0.869096466 | -1.150620257 | -0.202411775 | RecName: Full=Dynein light chain 2, cytoplasmic; AltName: Full=8 kDa dynein light chain b; Short=DLC8; Short=DLC8b; AltName: Full=Dynein light chain LC8-type 2 |
| gi37999865 | MLL3 | 2 | 0.869058493 | -1.150670534 | -0.202474813 | RecName: Full=Histone-lysine N-methyltransferase MLL3; AltName: Full=Myeloid/lymphoid or mixed-lineage leukemia protein 3 homolog |
| gi97536879 | NEFL | 20 | 0.867625213 | -1.152571392 | -0.204856117 | RecName: Full=Neurofilament light polypeptide; Short=NF-L; AltName: Full=68 kDa neurofilament protein; AltName: Full=Neurofilament triplet L protein |
| gi20177853 | CTNND2 | 2 | 0.866513652 | -1.154049907 | -0.206705615 | RecName: Full=Catenin delta-2; AltName: Full=Neural plakophilin-related ARM-repeat protein; Short=NPRAP; AltName: Full=Neurojungin |
| gi8928228 | NDRG2 | 5 | 0.866221135 | -1.154439622 | -0.207192722 | RecName: Full=Protein NDRG2; AltName: Full=Protein Ndr2 |
| gi14916528 | ADD2 | 5 | 0.865907169 | -1.154858207 | -0.207715729 | RecName: Full=Beta-adducin; AltName: Full=Add97; AltName: Full=Erythrocyte adducin subunit beta |
| gi341941734 | PSMC4 | 2 | 0.864812111 | -1.156320532 | -0.209541368 | RecName: Full=26S protease regulatory subunit 6B; AltName: Full=26S proteasome AAA-ATPase subunit RPT3; AltName: Full=CIP21; AltName: Full=MB67-interacting protein; AltName: Full=MIP224; AltName: Full=Proteasome 26S subunit ATPase 4; AltName: Full=Tat-binding protein 7; Short=TBP-7 |
| gi51702788 | RAC1 | 2 | 0.859689454 | -1.163210733 | -0.218112486 | RecName: Full=Ras-related C3 botulinum toxin substrate 1; AltName: Full=p21-Rac1; Flags: Precursor |
| gi81881569 | SH2D4A | 2 | 0.858674958 | -1.164585028 | -0.219815977 | RecName: Full=SH2 domain-containing protein 4A |
| gi21362402 | SDHC | 2 | 0.858518714 | -1.164796974 | -0.220078513 | RecName: Full=Succinate dehydrogenase cytochrome b560 subunit, mitochondrial; AltName: Full=Integral membrane protein CII-3; AltName: Full=QPs-1; Short=QPs1; Flags: Precursor |
| gi20177955 | CAMK2G | 8 | 0.858165131 | -1.165276896 | -0.220672812 | RecName: Full=Calcium/calmodulin-dependent protein kinase type II subunit gamma; Short=CaM kinase II subunit gamma; Short=CaMK-II subunit gamma |
| gi30581015 | APP | 2 | 0.857483874 | -1.16620269 | -0.221818556 | RecName: Full=Amyloid beta A4 protein; AltName: Full=ABPP; Short=APP; AltName: Full=Alzheimer disease amyloid A4 protein homolog; AltName: Full=Amyloidogenic glycoprotein; Short=AG; Contains: RecName: Full=N-APP; Contains: RecName: Full=Soluble APP-alpha; Short=S-APP-alpha; Contains: RecName: Full=Soluble APP-beta; Short=S-APP-beta; Contains: RecName: Full=C99; AltName: Full=APP-C99; Contains: RecName: Full=Beta-amyloid protein 42; AltName: Full=Beta-APP42; Contains: RecName: Full=Beta-amyloid protein 40; AltName: Full=Beta-APP40; Contains: RecName: Full=C83; Contains: RecName: Full=P3(42); Contains: RecName: Full=P3(40); Contains: RecName: Full=C80; Contains: RecName: Full=Gamma-secretase C-terminal fragment 59; AltName: Full=APP-C59; AltName: Full=Amyloid intracellular domain 59; Short=AID(59); AltName: Full=Gamma-CTF(59); Contains: RecName: Full=Gamma-secretase C-terminal fragment 57; AltName: Full=APP-C57; AltName: Full=Amyloid intracellular domain 57; Short=AID(57); AltName: Full=Gamma-CTF(57); Contains: RecName: Full=Gamma-secretase C-terminal fragment 50; AltName: Full=Amyloid intracellular domain 50; Short=AID(50); AltName: Full=Gamma-CTF(50); Contains: RecName: Full=C31; Flags: Precursor |
| gi20140091 | SFXN3 | 5 | 0.856546659 | -1.167478723 | -0.223396258 | RecName: Full=Sideroflexin-3 |
| gi32470593 | ALDOC | 11 | 0.850084539 | -1.176353591 | -0.234321774 | RecName: Full=Fructose-bisphosphate aldolase C; AltName: Full=Aldolase 3; AltName: Full=Brain-type aldolase; AltName: Full=Scrapie-responsive protein 2; AltName: Full=Zebrin II |
| gi17378829 | MBP | 9 | 0.849192298 | -1.177589578 | -0.235836809 | RecName: Full=Myelin basic protein; Short=MBP; AltName: Full=Myelin A1 protein |
| gi341940637 | CAP1 | 3 | 0.848904603 | -1.177988666 | -0.236325658 | RecName: Full=Adenylyl cyclase-associated protein 1; Short=CAP 1 |
| gi52783095 | PPA1 | 3 | 0.847658445 | -1.179720447 | -0.238445031 | RecName: Full=Inorganic pyrophosphatase; AltName: Full=Pyrophosphate phospho-hydrolase; Short=PPase |
| gi32363497 | EZR | 2 | 0.844490626 | -1.184145767 | -0.243846686 | RecName: Full=Ezrin; AltName: Full=Cytovillin; AltName: Full=Villin-2; AltName: Full=p81 |
| gi3913376 | CRYM | 5 | 0.839634512 | -1.190994398 | -0.252166628 | RecName: Full=Thiomorpholine-carboxylate dehydrogenase; AltName: Full=Mu-crystallin homolog; AltName: Full=NADP-regulated thyroid-hormone-binding protein; AltName: Full=ketimine reductase |
| gi8928304 | PTK6 | 2 | 0.832342705 | -1.201428203 | -0.264750436 | RecName: Full=Protein-tyrosine kinase 6; AltName: Full=SRC-related intestinal kinase |
| gi1710815 | S100B | 3 | 0.830264726 | -1.204435127 | -0.268356689 | RecName: Full=Protein S100-B; AltName: Full=S-100 protein beta chain; AltName: Full=S-100 protein subunit beta; AltName: Full=S100 calcium-binding protein B |
| gi549057 | CCT4 | 2 | 0.824722674 | -1.212528808 | -0.278019024 | RecName: Full=T-complex protein 1 subunit delta; Short=TCP-1-delta; AltName: Full=A45; AltName: Full=CCT-delta |
| gi549058 | CCT5 | 2 | 0.82464038 | -1.21264981 | -0.278162988 | RecName: Full=T-complex protein 1 subunit epsilon; Short=TCP-1-epsilon; AltName: Full=CCT-epsilon |
| gi38258618 | SIRT2 | 3 | 0.823916081 | -1.213715842 | -0.279430693 | RecName: Full=NAD-dependent deacetylase sirtuin-2; AltName: Full=SIR2-like protein 2; Short=mSIR2L2 |
| gi341941148 | GNAI2 | 5 | 0.818150236 | -1.222269402 | -0.289562307 | RecName: Full=Guanine nucleotide-binding protein G(i) subunit alpha-2; AltName: Full=Adenylate cyclase-inhibiting G alpha protein |
| gi3914939 | PSAP | 3 | 0.816365669 | -1.22494127 | -0.292712581 | RecName: Full=Sulfated glycoprotein 1; Short=SGP-1; AltName: Full=Prosaposin; Flags: Precursor |
| gi52000963 | GNAS (mouse) | 3 | 0.811153936 | -1.232811622 | -0.301952368 | RecName: Full=Guanine nucleotide-binding protein G(s) subunit alpha isoforms short; AltName: Full=Adenylate cyclase-stimulating G alpha protein |
| gi341940808 | HSPA2 | 13 | 0.810278553 | -1.234143489 | -0.303510141 | RecName: Full=Heat shock-related 70 kDa protein 2; Short=Heat shock protein 70.2 |
| gi54040727 | FASN | 5 | 0.808660798 | -1.236612436 | -0.30639342 | RecName: Full=Fatty acid synthase; Includes: RecName: Full=[Acyl-carrier-protein] S-acetyltransferase; Includes: RecName: Full=[Acyl-carrier-protein] S-malonyltransferase; Includes: RecName: Full=3-oxoacyl-[acyl-carrier-protein] synthase; Includes: RecName: Full=3-oxoacyl-[acyl-carrier-protein] reductase; Includes: RecName: Full=3-hydroxypalmitoyl-[acyl-carrier-protein] dehydratase; Includes: RecName: Full=Enoyl-[acyl-carrier-protein] reductase; Includes: RecName: Full=Oleoyl-[acyl-carrier-protein] hydrolase |
| gi51317403 | HPCAL1 | 6 | 0.807666344 | -1.238135038 | -0.308168672 | RecName: Full=Hippocalcin-like protein 1; AltName: Full=Neural visinin-like protein 3; Short=NVL-3; Short=NVP-3; AltName: Full=Visinin-like protein 3; Short=VILIP-3 |
| gi51317407 | HPCA | 9 | 0.807116098 | -1.238979129 | -0.309151886 | RecName: Full=Neuron-specific calcium-binding protein hippocalcin |
| gi81888798 | ERMN | 2 | 0.804332335 | -1.243267187 | -0.314136375 | RecName: Full=Ermin; AltName: Full=Juxtanodin; Short=JN |
| gi81900953 | COQ9 (includes EG:246650) | 2 | 0.800247212 | -1.24961385 | -0.321482349 | RecName: Full=Ubiquinone biosynthesis protein COQ9, mitochondrial; Flags: Precursor |
| gi6685313 | CLDN11 | 2 | 0.793264232 | -1.260614004 | -0.334126594 | RecName: Full=Claudin-11; AltName: Full=Oligodendrocyte transmembrane protein; AltName: Full=Oligodendrocyte-specific protein |
| gi47606758 | NCALD | 8 | 0.789655799 | -1.266374541 | -0.340704157 | RecName: Full=Neurocalcin-delta |
| gi81908472 | F5 | 3 | 0.787429125 | -1.269955566 | -0.34477802 | RecName: Full=Coagulation factor V; AltName: Full=Activated protein C cofactor; Contains: RecName: Full=Coagulation factor V heavy chain; Contains: RecName: Full=Coagulation factor V light chain; Flags: Precursor |
| gi327488098 | EXOC6B | 2 | 0.784079985 | -1.275380087 | -0.350927261 | RecName: Full=Exocyst complex component 6B; AltName: Full=Exocyst complex component Sec15B; AltName: Full=SEC15-like protein 2 |
| gi341942067 | CTTN | 2 | 0.776450678 | -1.287911813 | -0.365033811 | RecName: Full=Src substrate cortactin |
| gi266683 | OAT | 2 | 0.764581375 | -1.307905257 | -0.387258038 | RecName: Full=Ornithine aminotransferase, mitochondrial; AltName: Full=Ornithine--oxo-acid aminotransferase; Flags: Precursor |
| gi73920250 | VBP1 | 2 | 0.734340201 | -1.361766656 | -0.445479513 | RecName: Full=Prefoldin subunit 3; AltName: Full=Von Hippel-Lindau-binding protein 1; Short=VBP-1; Short=VHL-binding protein 1 |
| gi47605401 | CSRP1 | 3 | 0.732230909 | -1.365689412 | -0.44962942 | RecName: Full=Cysteine and glycine-rich protein 1; AltName: Full=Cysteine-rich protein 1; Short=CRP; Short=CRP1 |
| gi44888257 | PICALM | 2 | 0.681953814 | -1.466374966 | -0.552254061 | RecName: Full=Phosphatidylinositol-binding clathrin assembly protein; AltName: Full=Clathrin assembly lymphoid myeloid leukemia; Short=CALM |
| gi398990 | DBI | 2 | 0.668320308 | -1.496288514 | -0.581388382 | RecName: Full=Acyl-CoA-binding protein; Short=ACBP; AltName: Full=Diazepam-binding inhibitor; Short=DBI; AltName: Full=Endozepine; Short=EP |
| gi17380463 | NUCB1 | 2 | 0.613043457 | -1.631205729 | -0.705938748 | RecName: Full=Nucleobindin-1; AltName: Full=CALNUC; Flags: Precursor |
| gi18202239 | CAPNS1 | 2 | 0.569331644 | -1.756445493 | -0.812658807 | RecName: Full=Calpain small subunit 1; Short=CSS1; AltName: Full=Calcium-activated neutral proteinase small subunit; Short=CANP small subunit; AltName: Full=Calcium-dependent protease small subunit; Short=CDPS; AltName: Full=Calcium-dependent protease small subunit 1; AltName: Full=Calpain regulatory subunit |
| gi52783214 | TOMM20 | 2 | 0.549479084 | -1.819905488 | -0.86386353 | RecName: Full=Mitochondrial import receptor subunit TOM20 homolog; AltName: Full=Mitochondrial 20 kDa outer membrane protein; AltName: Full=Outer mitochondrial membrane receptor Tom20 |
| gi52001483 | MAPK3 | 2 | 0.52115543 | -1.918813356 | -0.940214387 | RecName: Full=Mitogen-activated protein kinase 3; Short=MAP kinase 3; Short=MAPK 3; AltName: Full=ERT2; AltName: Full=Extracellular signal-regulated kinase 1; Short=ERK-1; AltName: Full=Insulin-stimulated MAP2 kinase; AltName: Full=MAP kinase isoform p44; Short=p44-MAPK; AltName: Full=MNK1; AltName: Full=Microtubule-associated protein 2 kinase; AltName: Full=Mitogen-activated protein kinase 1; Short=MAP kinase 1; Short=MAPK 1; AltName: Full=p44-ERK1 |
