# Supplementary material for: The effects of aging on the BTBR mouse model of autism spectrum disorder
Source: Front Aging Neurosci. 2014 Sep 1;6:225. doi: 10.3389/fnagi.2014.00225 (PMC4150363; doi:10.3389/fnagi.2014.00225)
Supplement: Supplementary file 4 [file Table2.DOCX]

**Table S2. Identified and quantified proteins from BTBR hippocampus.** iTRAQ expression ratios (BTBR:WT), Fold Changes and Log_2_ transformed iTRAQ ratios were generated for proteins extracted from hippocampal BTBR tissues compared to WT controls.

| **Accession** | **Symbol** | **Σ# Peptides** | **Hippo BTBR:WT** | **Fold Change** | **log2 ratio** | **Description** |
| --- | --- | --- | --- | --- | --- | --- |
| gi81914749 | WDR33 | 3 | 2.510567518 | 2.510567518 | 1.328013525 | RecName: Full=pre-mRNA 3' end processing protein WDR33; AltName: Full=WD repeat-containing protein 33; AltName: Full=WD repeat-containing protein WDC146 |
| gi378522303 | PRRT2 | 2 | 2.381953722 | 2.381953722 | 1.252145384 | RecName: Full=Proline-rich transmembrane protein 2; AltName: Full=Dispanin subfamily B member 3 |
| gi81876946 | PCYOX1L | 2 | 1.860882083 | 1.860882083 | 0.89598664 | RecName: Full=Prenylcysteine oxidase-like; Flags: Precursor |
| gi61219108 | AP3B2 | 2 | 1.789312776 | 1.789312776 | 0.839405596 | RecName: Full=AP-3 complex subunit beta-2; AltName: Full=Adapter-related protein complex 3 subunit beta-2; AltName: Full=Adaptor protein complex AP-3 subunit beta-2; AltName: Full=Beta-3B-adaptin; AltName: Full=Clathrin assembly protein complex 3 beta-2 large chain |
| gi20138800 | ITSN1 | 3 | 1.774926636 | 1.774926636 | 0.827759394 | RecName: Full=Intersectin-1; AltName: Full=EH and SH3 domains protein 1 |
| gi52783579 | DYNLL1 | 2 | 1.737545789 | 1.737545789 | 0.797050997 | RecName: Full=Dynein light chain 1, cytoplasmic; AltName: Full=8 kDa dynein light chain; Short=DLC8; AltName: Full=Dynein light chain LC8-type 1; AltName: Full=Protein inhibitor of neuronal nitric oxide synthase; Short=PIN; Short=mPIN |
| gi62286986 | NUDC | 2 | 1.732009523 | 1.732009523 | 0.792446862 | RecName: Full=Nuclear migration protein nudC; AltName: Full=Nuclear distribution protein C homolog; AltName: Full=Silica-induced gene 92 protein; Short=SIG-92 |
| gi47117763 | RAP2B | 2 | 1.726355193 | 1.726355193 | 0.787729326 | RecName: Full=Ras-related protein Rap-2b; Flags: Precursor |
| gi158931128 | KIF2A | 2 | 1.663032474 | 1.663032474 | 0.733816341 | RecName: Full=Kinesin-like protein KIF2A; AltName: Full=Kinesin-2 |
| gi25091206 | SETDB1 | 2 | 1.648556725 | 1.648556725 | 0.721203529 | RecName: Full=Histone-lysine N-methyltransferase SETDB1; AltName: Full=ERG-associated protein with SET domain; Short=ESET; AltName: Full=SET domain bifurcated 1 |
| gi12585446 | ATP6V1D (includes EG:299159) | 2 | 1.640964877 | 1.640964877 | 0.71454436 | RecName: Full=V-type proton ATPase subunit D; Short=V-ATPase subunit D; AltName: Full=V-ATPase 28 kDa accessory protein; AltName: Full=Vacuolar proton pump subunit D |
| gi341940931 | ME3 | 2 | 1.62730561 | 1.62730561 | 0.702485216 | RecName: Full=NADP-dependent malic enzyme, mitochondrial; Short=NADP-ME; AltName: Full=Malic enzyme 3; Flags: Precursor |
| gi294862512 | GOLGA2 (includes EG:2801) | 2 | 1.60040989 | 1.60040989 | 0.678441449 | RecName: Full=Golgin subfamily A member 2; AltName: Full=130 kDa cis-Golgi matrix protein; Short=GM130 |
| gi20981714 | SYNGR1 | 2 | 1.58532711 | 1.58532711 | 0.664780551 | RecName: Full=Synaptogyrin-1 |
| gi3122052 | DPYSL3 | 5 | 1.582802831 | 1.582802831 | 0.662481551 | RecName: Full=Dihydropyrimidinase-related protein 3; Short=DRP-3; AltName: Full=Unc-33-like phosphoprotein 1; Short=ULIP-1 |
| gi1709255 | NCAN | 2 | 1.571585688 | 1.571585688 | 0.652220935 | RecName: Full=Neurocan core protein; AltName: Full=Chondroitin sulfate proteoglycan 3; Flags: Precursor |
| gi20177853 | CTNND2 | 2 | 1.562506309 | 1.562506309 | 0.643862015 | RecName: Full=Catenin delta-2; AltName: Full=Neural plakophilin-related ARM-repeat protein; Short=NPRAP; AltName: Full=Neurojungin |
| gi68565118 | CNRIP1 | 2 | 1.554254382 | 1.554254382 | 0.636222646 | RecName: Full=CB1 cannabinoid receptor-interacting protein 1; Short=CRIP-1 |
| gi62510597 | UTP20 | 2 | 1.544855143 | 1.544855143 | 0.627471567 | RecName: Full=Small subunit processome component 20 homolog; AltName: Full=Down-regulated in metastasis protein |
| gi341940932 | ME1 | 3 | 1.478800627 | 1.478800627 | 0.56442756 | RecName: Full=NADP-dependent malic enzyme; Short=NADP-ME; AltName: Full=Malic enzyme 1 |
| gi51702779 | PFN1 | 2 | 1.477145399 | 1.477145399 | 0.562811841 | RecName: Full=Profilin-1; AltName: Full=Profilin I |
| gi108935875 | PPME1 | 2 | 1.455765369 | 1.455765369 | 0.54177785 | RecName: Full=Protein phosphatase methylesterase 1; Short=PME-1 |
| gi30581015 | APP | 2 | 1.455465641 | 1.455465641 | 0.541480782 | RecName: Full=Amyloid beta A4 protein; AltName: Full=ABPP; Short=APP; AltName: Full=Alzheimer disease amyloid A4 protein homolog; AltName: Full=Amyloidogenic glycoprotein; Short=AG; Contains: RecName: Full=N-APP; Contains: RecName: Full=Soluble APP-alpha; Short=S-APP-alpha; Contains: RecName: Full=Soluble APP-beta; Short=S-APP-beta; Contains: RecName: Full=C99; AltName: Full=APP-C99; Contains: RecName: Full=Beta-amyloid protein 42; AltName: Full=Beta-APP42; Contains: RecName: Full=Beta-amyloid protein 40; AltName: Full=Beta-APP40; Contains: RecName: Full=C83; Contains: RecName: Full=P3(42); Contains: RecName: Full=P3(40); Contains: RecName: Full=C80; Contains: RecName: Full=Gamma-secretase C-terminal fragment 59; AltName: Full=APP-C59; AltName: Full=Amyloid intracellular domain 59; Short=AID(59); AltName: Full=Gamma-CTF(59); Contains: RecName: Full=Gamma-secretase C-terminal fragment 57; AltName: Full=APP-C57; AltName: Full=Amyloid intracellular domain 57; Short=AID(57); AltName: Full=Gamma-CTF(57); Contains: RecName: Full=Gamma-secretase C-terminal fragment 50; AltName: Full=Amyloid intracellular domain 50; Short=AID(50); AltName: Full=Gamma-CTF(50); Contains: RecName: Full=C31; Flags: Precursor |
| gi47116926 | LANCL2 | 2 | 1.455132031 | 1.455132031 | 0.541150062 | RecName: Full=LanC-like protein 2; AltName: Full=Testis-specific adriamycin sensitivity protein |
| gi118595720 | CEP290 | 2 | 1.454207016 | 1.454207016 | 0.540232662 | RecName: Full=Centrosomal protein of 290 kDa; Short=Cep290; AltName: Full=Bardet-Biedl syndrome 14 protein homolog; AltName: Full=Nephrocystin-6 |
| gi251757493 | GNAQ | 2 | 1.453050806 | 1.453050806 | 0.539085148 | RecName: Full=Guanine nucleotide-binding protein G(q) subunit alpha; AltName: Full=Guanine nucleotide-binding protein alpha-q |
| gi81881592 | ARPC5L | 2 | 1.451224208 | 1.451224208 | 0.537270427 | RecName: Full=Actin-related protein 2/3 complex subunit 5-like protein; AltName: Full=Arp2/3 complex 16 kDa subunit 2; Short=ARC16-2 |
| gi2499469 | PRDX2 | 5 | 1.448929334 | 1.448929334 | 0.534987234 | RecName: Full=Peroxiredoxin-2; AltName: Full=Thiol-specific antioxidant protein; Short=TSA; AltName: Full=Thioredoxin peroxidase 1; AltName: Full=Thioredoxin-dependent peroxide reductase 1 |
| gi81885902 | CYFIP1 | 4 | 1.432347642 | 1.432347642 | 0.518381688 | RecName: Full=Cytoplasmic FMR1-interacting protein 1; AltName: Full=Specifically Rac1-associated protein 1; Short=Sra-1 |
| gi341940808 | HSPA2 | 13 | 1.431503207 | 1.431503207 | 0.517530903 | RecName: Full=Heat shock-related 70 kDa protein 2; Short=Heat shock protein 70.2 |
| gi68566057 | SFPQ | 5 | 1.427372534 | 1.427372534 | 0.513361917 | RecName: Full=Splicing factor, proline- and glutamine-rich; AltName: Full=DNA-binding p52/p100 complex, 100 kDa subunit; AltName: Full=Polypyrimidine tract-binding protein-associated-splicing factor; Short=PSF; Short=PTB-associated-splicing factor |
| gi55583934 | TXLNG | 2 | 1.42119969 | 1.42119969 | 0.507109279 | RecName: Full=Gamma-taxilin; AltName: Full=Factor inhibiting ATF4-mediated transcription; Short=FIAT; AltName: Full=Lipopolysaccharide-responsive gene protein |
| gi2498425 | GRB2 | 3 | 1.4157442 | 1.4157442 | 0.501560619 | RecName: Full=Growth factor receptor-bound protein 2; AltName: Full=Adapter protein GRB2; AltName: Full=SH2/SH3 adapter GRB2 |
| gi10719959 | CPNE6 | 3 | 1.414165286 | 1.414165286 | 0.49995075 | RecName: Full=Copine-6; AltName: Full=Copine VI; AltName: Full=Neuronal-copine; Short=N-copine |
| gi44888293 | PDXP | 3 | 1.410098436 | 1.410098436 | 0.495795877 | RecName: Full=Pyridoxal phosphate phosphatase; Short=PLP phosphatase; AltName: Full=Chronophin |
| gi51317305 | GNB2 | 9 | 1.408516442 | 1.408516442 | 0.494176404 | RecName: Full=Guanine nucleotide-binding protein G(I)/G(S)/G(T) subunit beta-2; AltName: Full=G protein subunit beta-2; AltName: Full=Transducin beta chain 2 |
| gi52783066 | VAT1L | 2 | 1.401775589 | 1.401775589 | 0.487255406 | RecName: Full=Synaptic vesicle membrane protein VAT-1 homolog-like |
| gi81916755 | GLOD4 | 3 | 1.391614359 | 1.391614359 | 0.47675947 | RecName: Full=Glyoxalase domain-containing protein 4 |
| gi294862498 | OXR1 (includes EG:117520) | 4 | 1.380069445 | 1.380069445 | 0.464740865 | RecName: Full=Oxidation resistance protein 1; AltName: Full=Protein C7 |
| gi51704193 | VAMP2 | 3 | 1.373709575 | 1.373709575 | 0.458077026 | RecName: Full=Vesicle-associated membrane protein 2; Short=VAMP-2; AltName: Full=Synaptobrevin-2 |
| gi56757667 | HSPA1A/HSPA1B | 9 | 1.373028035 | 1.373028035 | 0.457361083 | RecName: Full=Heat shock 70 kDa protein 1A; AltName: Full=Heat shock 70 kDa protein 3; Short=HSP70.3; AltName: Full=Hsp68 |
| gi160419228 | PSD3 | 2 | 1.371411227 | 1.371411227 | 0.455661238 | RecName: Full=PH and SEC7 domain-containing protein 3; AltName: Full=Exchange factor for ADP-ribosylation factor guanine nucleotide factor 6; AltName: Full=Pleckstrin homology and SEC7 domain-containing protein 3 |
| gi51704260 | BSG (includes EG:12215) | 2 | 1.369172074 | 1.369172074 | 0.453303772 | RecName: Full=Basigin; AltName: Full=Basic immunoglobulin superfamily; AltName: Full=HT7 antigen; AltName: Full=Membrane glycoprotein gp42; AltName: CD_antigen=CD147; Flags: Precursor |
| gi13124470 | PFN2 | 5 | 1.368469072 | 1.368469072 | 0.45256283 | RecName: Full=Profilin-2; AltName: Full=Profilin II |
| gi20141252 | ATP5J2 | 2 | 1.368321641 | 1.368321641 | 0.452407393 | RecName: Full=ATP synthase subunit f, mitochondrial |
| gi51317303 | GNB1 | 10 | 1.366417736 | 1.366417736 | 0.450398607 | RecName: Full=Guanine nucleotide-binding protein G(I)/G(S)/G(T) subunit beta-1; AltName: Full=Transducin beta chain 1 |
| gi2497501 | DLG4 | 3 | 1.363981591 | 1.363981591 | 0.447824173 | RecName: Full=Disks large homolog 4; AltName: Full=Postsynaptic density protein 95; Short=PSD-95; AltName: Full=Synapse-associated protein 90; Short=SAP-90; Short=SAP90 |
| gi160409932 | SH3GLB2 | 3 | 1.36363306 | 1.36363306 | 0.447455482 | RecName: Full=Endophilin-B2; AltName: Full=SH3 domain-containing GRB2-like protein B2 |
| gi81862978 | KTN1 | 2 | 1.362629072 | 1.362629072 | 0.446392893 | RecName: Full=Kinectin |
| gi266683 | OAT | 2 | 1.362629072 | 1.362629072 | 0.446392893 | RecName: Full=Ornithine aminotransferase, mitochondrial; AltName: Full=Ornithine--oxo-acid aminotransferase; Flags: Precursor |
| gi51702798 | RAB3A | 10 | 1.362173026 | 1.362173026 | 0.445909969 | RecName: Full=Ras-related protein Rab-3A |
| gi549060 | CCT7 | 2 | 1.3567651 | 1.3567651 | 0.440170965 | RecName: Full=T-complex protein 1 subunit eta; Short=TCP-1-eta; AltName: Full=CCT-eta |
| gi21362402 | SDHC | 2 | 1.347654394 | 1.347654394 | 0.430450564 | RecName: Full=Succinate dehydrogenase cytochrome b560 subunit, mitochondrial; AltName: Full=Integral membrane protein CII-3; AltName: Full=QPs-1; Short=QPs1; Flags: Precursor |
| gi341941247 | BCAN | 3 | 1.342898167 | 1.342898167 | 0.425349908 | RecName: Full=Brevican core protein; Flags: Precursor |
| gi97536358 | HSPH1 | 2 | 1.337373319 | 1.337373319 | 0.419402241 | RecName: Full=Heat shock protein 105 kDa; AltName: Full=42 degrees C-HSP; AltName: Full=Heat shock 110 kDa protein; AltName: Full=Heat shock-related 100 kDa protein E7I; Short=HSP-E7I |
| gi14916528 | ADD2 | 5 | 1.336930818 | 1.336930818 | 0.418924812 | RecName: Full=Beta-adducin; AltName: Full=Add97; AltName: Full=Erythrocyte adducin subunit beta |
| gi341940231 | AP2A2 | 12 | 1.335723865 | 1.335723865 | 0.417621789 | RecName: Full=AP-2 complex subunit alpha-2; AltName: Full=100 kDa coated vesicle protein C; AltName: Full=Adapter-related protein complex 2 alpha-2 subunit; AltName: Full=Adaptor protein complex AP-2 subunit alpha-2; AltName: Full=Alpha-adaptin C; AltName: Full=Alpha2-adaptin; AltName: Full=Clathrin assembly protein complex 2 alpha-C large chain; AltName: Full=Plasma membrane adaptor HA2/AP2 adaptin alpha C subunit |
| gi56748875 | DYNLL2 | 2 | 1.322647505 | 1.322647505 | 0.403428625 | RecName: Full=Dynein light chain 2, cytoplasmic; AltName: Full=8 kDa dynein light chain b; Short=DLC8; Short=DLC8b; AltName: Full=Dynein light chain LC8-type 2 |
| gi47115588 | LANCL1 | 2 | 1.320074872 | 1.320074872 | 0.400619759 | RecName: Full=LanC-like protein 1; AltName: Full=40 kDa erythrocyte membrane protein; Short=p40 |
| gi9910725 | GDA | 3 | 1.312825433 | 1.312825433 | 0.392675093 | RecName: Full=Guanine deaminase; Short=Guanase; Short=Guanine aminase; AltName: Full=Guanine aminohydrolase; Short=GAH |
| gi730956 | TKT | 6 | 1.3105561 | 1.3105561 | 0.390179112 | RecName: Full=Transketolase; Short=TK; AltName: Full=P68 |
| gi8928560 | COX7A2 | 2 | 1.308575621 | 1.308575621 | 0.387997299 | RecName: Full=Cytochrome c oxidase subunit 7A2, mitochondrial; AltName: Full=Cytochrome c oxidase subunit VIIa-liver/heart; Short=Cytochrome c oxidase subunit VIIa-L; Flags: Precursor |
| gi13432217 | SNCA | 5 | 1.305570043 | 1.305570043 | 0.38467986 | RecName: Full=Alpha-synuclein; AltName: Full=Non-A beta component of AD amyloid; AltName: Full=Non-A4 component of amyloid precursor; Short=NACP |
| gi73917637 | BAIAP2 | 2 | 1.303596608 | 1.303596608 | 0.382497503 | RecName: Full=Brain-specific angiogenesis inhibitor 1-associated protein 2; Short=BAI-associated protein 2; Short=BAI1-associated protein 2; AltName: Full=Insulin receptor substrate protein of 53 kDa; Short=IRSp53; Short=Insulin receptor substrate p53; AltName: Full=Insulin receptor tyrosine kinase 53 kDa substrate |
| gi24638218 | SH3BGRL3 | 3 | 1.302583328 | 1.302583328 | 0.381375667 | RecName: Full=SH3 domain-binding glutamic acid-rich-like protein 3 |
| gi2500582 | NPM1 | 3 | 1.301978732 | 1.301978732 | 0.380705882 | RecName: Full=Nucleophosmin; Short=NPM; AltName: Full=Nucleolar phosphoprotein B23; AltName: Full=Nucleolar protein NO38; AltName: Full=Numatrin |
| gi17380130 | NIPSNAP1 | 4 | 1.299090793 | 1.299090793 | 0.377502263 | RecName: Full=Protein NipSnap homolog 1; Short=NipSnap1 |
| gi38372626 | ARPC4 | 2 | 1.296939524 | 1.296939524 | 0.375111209 | RecName: Full=Actin-related protein 2/3 complex subunit 4; AltName: Full=Arp2/3 complex 20 kDa subunit; Short=p20-ARC |
| gi17380315 | NAPA (includes EG:108124) | 3 | 1.292138971 | 1.292138971 | 0.369761242 | RecName: Full=Alpha-soluble NSF attachment protein; Short=SNAP-alpha; AltName: Full=N-ethylmaleimide-sensitive factor attachment protein alpha |
| gi115395 | CALB1 | 3 | 1.290893833 | 1.290893833 | 0.368370354 | RecName: Full=Calbindin; AltName: Full=Calbindin D28; AltName: Full=D-28K; AltName: Full=PCD-29; AltName: Full=Spot 35 protein; AltName: Full=Vitamin D-dependent calcium-binding protein, avian-type |
| gi115311320 | CAMKV | 9 | 1.286413462 | 1.286413462 | 0.363354409 | RecName: Full=CaM kinase-like vesicle-associated protein |
| gi48428148 | COX6C | 2 | 1.286388519 | 1.286388519 | 0.363326436 | RecName: Full=Cytochrome c oxidase subunit 6C; AltName: Full=Cytochrome c oxidase polypeptide VIc |
| gi117104 | COX5B | 4 | 1.285714649 | 1.285714649 | 0.362570487 | RecName: Full=Cytochrome c oxidase subunit 5B, mitochondrial; AltName: Full=Cytochrome c oxidase polypeptide Vb; Flags: Precursor |
| gi341941159 | NDUFA9 | 6 | 1.285358088 | 1.285358088 | 0.362170336 | RecName: Full=NADH dehydrogenase [ubiquinone] 1 alpha subcomplex subunit 9, mitochondrial; AltName: Full=Complex I-39kD; Short=CI-39kD; AltName: Full=NADH-ubiquinone oxidoreductase 39 kDa subunit; Flags: Precursor |
| gi12643287 | ATP6V0D1 | 3 | 1.275603048 | 1.275603048 | 0.35117945 | RecName: Full=V-type proton ATPase subunit d 1; Short=V-ATPase subunit d 1; AltName: Full=P39; AltName: Full=Physophilin; AltName: Full=V-ATPase 40 kDa accessory protein; AltName: Full=V-ATPase AC39 subunit; AltName: Full=Vacuolar proton pump subunit d 1 |
| gi41017503 | NSFL1C | 2 | 1.274526051 | 1.274526051 | 0.349960862 | RecName: Full=NSFL1 cofactor p47; AltName: Full=p97 cofactor p47 |
| gi126048 | LDHA | 12 | 1.269135901 | 1.269135901 | 0.343846564 | RecName: Full=L-lactate dehydrogenase A chain; Short=LDH-A; AltName: Full=LDH muscle subunit; Short=LDH-M |
| gi47117625 | ATP6V1F | 4 | 1.266693513 | 1.266693513 | 0.341067494 | RecName: Full=V-type proton ATPase subunit F; Short=V-ATPase subunit F; AltName: Full=V-ATPase 14 kDa subunit; AltName: Full=Vacuolar proton pump subunit F |
| gi62510439 | CLTB | 5 | 1.265664013 | 1.265664013 | 0.339894473 | RecName: Full=Clathrin light chain B; Short=Lcb |
| gi20978758 | SRSF4 | 3 | 1.261593119 | 1.261593119 | 0.335246697 | RecName: Full=Serine/arginine-rich splicing factor 4; AltName: Full=Splicing factor, arginine/serine-rich 4 |
| gi81905373 | LRGUK | 2 | 1.260891479 | 1.260891479 | 0.334444113 | RecName: Full=Leucine-rich repeat and guanylate kinase domain-containing protein |
| gi47117242 | NDUFS8 | 4 | 1.260447912 | 1.260447912 | 0.3339365 | RecName: Full=NADH dehydrogenase [ubiquinone] iron-sulfur protein 8, mitochondrial; AltName: Full=Complex I-23kD; Short=CI-23kD; AltName: Full=NADH-ubiquinone oxidoreductase 23 kDa subunit; Flags: Precursor |
| gi50402237 | PPP2CB | 5 | 1.259397109 | 1.259397109 | 0.332733261 | RecName: Full=Serine/threonine-protein phosphatase 2A catalytic subunit beta isoform; Short=PP2A-beta |
| gi416827 | COX4I1 | 4 | 1.256849924 | 1.256849924 | 0.329812392 | RecName: Full=Cytochrome c oxidase subunit 4 isoform 1, mitochondrial; AltName: Full=Cytochrome c oxidase polypeptide IV; AltName: Full=Cytochrome c oxidase subunit IV isoform 1; Short=COX IV-1; Flags: Precursor |
| gi81911483 | CAMK2D | 10 | 1.256848229 | 1.256848229 | 0.329810447 | RecName: Full=Calcium/calmodulin-dependent protein kinase type II subunit delta; Short=CaM kinase II subunit delta; Short=CaMK-II subunit delta |
| gi109818808 | DNAJC6 | 3 | 1.255985647 | 1.255985647 | 0.328819978 | RecName: Full=Putative tyrosine-protein phosphatase auxilin; AltName: Full=DnaJ homolog subfamily C member 6 |
| gi125987813 | COL6A2 | 2 | 1.253698992 | 1.253698992 | 0.326191005 | RecName: Full=Collagen alpha-2(VI) chain; Flags: Precursor |
| gi3913376 | CRYM | 5 | 1.251866833 | 1.251866833 | 0.324081104 | RecName: Full=Thiomorpholine-carboxylate dehydrogenase; AltName: Full=Mu-crystallin homolog; AltName: Full=NADP-regulated thyroid-hormone-binding protein; AltName: Full=ketimine reductase |
| gi54038800 | PPP3CA | 15 | 1.249125255 | 1.249125255 | 0.320918149 | RecName: Full=Serine/threonine-protein phosphatase 2B catalytic subunit alpha isoform; AltName: Full=CAM-PRP catalytic subunit; AltName: Full=Calmodulin-dependent calcineurin A subunit alpha isoform |
| gi341941123 | SLC2A1 | 2 | 1.24839376 | 1.24839376 | 0.320073051 | RecName: Full=Solute carrier family 2, facilitated glucose transporter member 1; AltName: Full=Glucose transporter type 1, erythrocyte/brain; Short=GLUT-1; Short=GT1 |
| gi51315842 | DBNL | 4 | 1.24548243 | 1.24548243 | 0.31670467 | RecName: Full=Drebrin-like protein; AltName: Full=Actin-binding protein 1; AltName: Full=SH3 domain-containing protein 7 |
| gi42558958 | PPFIA3 | 2 | 1.244596343 | 1.244596343 | 0.315677912 | RecName: Full=Liprin-alpha-3; AltName: Full=Protein tyrosine phosphatase receptor type f polypeptide-interacting protein alpha-3; Short=PTPRF-interacting protein alpha-3 |
| gi124056467 | CAMK2A | 16 | 1.241519397 | 1.241519397 | 0.312106802 | RecName: Full=Calcium/calmodulin-dependent protein kinase type II subunit alpha; Short=CaM kinase II subunit alpha; Short=CaMK-II subunit alpha |
| gi41019466 | SYP | 5 | 1.237859198 | 1.237859198 | 0.307847222 | RecName: Full=Synaptophysin; AltName: Full=BM89 antigen; AltName: Full=Major synaptic vesicle protein p38 |
| gi51701351 | AP2B1 | 16 | 1.237413188 | 1.237413188 | 0.307327315 | RecName: Full=AP-2 complex subunit beta; AltName: Full=AP105B; AltName: Full=Adapter-related protein complex 2 beta subunit; AltName: Full=Adaptor protein complex AP-2 subunit beta; AltName: Full=Beta-2-adaptin; AltName: Full=Beta-adaptin; AltName: Full=Clathrin assembly protein complex 2 beta large chain; AltName: Full=Plasma membrane adaptor HA2/AP2 adaptin beta subunit |
| gi212288549 | USP24 | 2 | 1.233820239 | 1.233820239 | 0.303132217 | RecName: Full=Ubiquitin carboxyl-terminal hydrolase 24; AltName: Full=Deubiquitinating enzyme 24; AltName: Full=Ubiquitin thiolesterase 24; AltName: Full=Ubiquitin-specific-processing protease 24 |
| gi462064 | FABP5 | 2 | 1.233314773 | 1.233314773 | 0.302541059 | RecName: Full=Fatty acid-binding protein, epidermal; AltName: Full=Epidermal-type fatty acid-binding protein; Short=E-FABP; AltName: Full=Fatty acid-binding protein 5; AltName: Full=Keratinocyte lipid-binding protein; AltName: Full=Psoriasis-associated fatty acid-binding protein homolog; Short=PA-FABP |
| gi56749655 | SEPT9 | 2 | 1.226916013 | 1.226916013 | 0.295036495 | RecName: Full=Septin-9; AltName: Full=SL3-3 integration site 1 protein |
| gi18203578 | EHD1 | 2 | 1.226344591 | 1.226344591 | 0.294364419 | RecName: Full=EH domain-containing protein 1; AltName: Full=PAST homolog 1; Short=mPAST1 |
| gi32469605 | CAP2 | 4 | 1.223807885 | 1.223807885 | 0.2913771 | RecName: Full=Adenylyl cyclase-associated protein 2; Short=CAP 2 |
| gi21759257 | SLC25A3 | 10 | 1.221941931 | 1.221941931 | 0.289175727 | RecName: Full=Phosphate carrier protein, mitochondrial; AltName: Full=Phosphate transport protein; Short=PTP; AltName: Full=Solute carrier family 25 member 3; Flags: Precursor |
| gi94730394 | CAMK2B | 15 | 1.22058499 | 1.22058499 | 0.287572755 | RecName: Full=Calcium/calmodulin-dependent protein kinase type II subunit beta; Short=CaM kinase II subunit beta; Short=CaMK-II subunit beta |
| gi8928249 | PTGES3 | 2 | 1.216665914 | 1.216665914 | 0.282933071 | RecName: Full=Prostaglandin E synthase 3; AltName: Full=Cytosolic prostaglandin E2 synthase; Short=cPGES; AltName: Full=Hsp90 co-chaperone; AltName: Full=Progesterone receptor complex p23; AltName: Full=Sid 3177; AltName: Full=Telomerase-binding protein p23 |
| gi547923 | PRDX1 | 8 | 1.215985447 | 1.215985447 | 0.282125962 | RecName: Full=Peroxiredoxin-1; AltName: Full=Macrophage 23 kDa stress protein; AltName: Full=Osteoblast-specific factor 3; Short=OSF-3; AltName: Full=Thioredoxin peroxidase 2; AltName: Full=Thioredoxin-dependent peroxide reductase 2 |
| gi461586 | ATP5I | 3 | 1.215521395 | 1.215521395 | 0.281575287 | RecName: Full=ATP synthase subunit e, mitochondrial; Short=ATPase subunit e |
| gi146345497 | ATIC | 4 | 1.213849668 | 1.213849668 | 0.279589759 | RecName: Full=Bifunctional purine biosynthesis protein PURH; Includes: RecName: Full=Phosphoribosylaminoimidazolecarboxamide formyltransferase; AltName: Full=5-aminoimidazole-4-carboxamide ribonucleotide formyltransferase; AltName: Full=AICAR transformylase; Includes: RecName: Full=IMP cyclohydrolase; AltName: Full=ATIC; AltName: Full=IMP synthase; AltName: Full=Inosinicase |
| gi121716 | GSTM5 | 13 | 1.213704207 | 1.213704207 | 0.279416864 | RecName: Full=Glutathione S-transferase Mu 1; AltName: Full=GST 1-1; AltName: Full=GST class-mu 1; AltName: Full=Glutathione S-transferase GT8.7; AltName: Full=pmGT10 |
| gi47117311 | NDUFS7 | 2 | 1.211966939 | 1.211966939 | 0.277350344 | RecName: Full=NADH dehydrogenase [ubiquinone] iron-sulfur protein 7, mitochondrial; AltName: Full=Complex I-20kD; Short=CI-20kD; AltName: Full=NADH-ubiquinone oxidoreductase 20 kDa subunit; Flags: Precursor |
| gi20137987 | DCLK1 | 3 | 1.207992461 | 1.207992461 | 0.272611451 | RecName: Full=Serine/threonine-protein kinase DCLK1; AltName: Full=Doublecortin-like and CAM kinase-like 1; AltName: Full=Doublecortin-like kinase 1 |
| gi147644461 | GPD1L | 2 | 1.207286149 | 1.207286149 | 0.271767662 | RecName: Full=Glycerol-3-phosphate dehydrogenase 1-like protein |
| gi215273994 | HNRNPL | 3 | 1.205847041 | 1.205847041 | 0.270046916 | RecName: Full=Heterogeneous nuclear ribonucleoprotein L; Short=hnRNP L |
| gi47117840 | NAPB | 11 | 1.202833155 | 1.202833155 | 0.26643654 | RecName: Full=Beta-soluble NSF attachment protein; Short=SNAP-beta; AltName: Full=Brain protein I47; AltName: Full=N-ethylmaleimide-sensitive factor attachment protein beta |
| gi94730421 | RTN4 (includes EG:57142) | 2 | 1.201798491 | 1.201798491 | 0.265195016 | RecName: Full=Reticulon-4; AltName: Full=Neurite outgrowth inhibitor; Short=Nogo protein |
| gi341941800 | RASAL1 | 4 | 1.199346747 | 1.199346747 | 0.262248822 | RecName: Full=RasGAP-activating-like protein 1 |
| gi10720404 | VDAC1 | 12 | 1.198310899 | 1.198310899 | 0.26100226 | RecName: Full=Voltage-dependent anion-selective channel protein 1; Short=VDAC-1; Short=mVDAC1; AltName: Full=Outer mitochondrial membrane protein porin 1; AltName: Full=Plasmalemmal porin; AltName: Full=Voltage-dependent anion-selective channel protein 5; Short=VDAC-5; Short=mVDAC5 |
| gi548409 | PDHA1 | 9 | 1.197585092 | 1.197585092 | 0.260128167 | RecName: Full=Pyruvate dehydrogenase E1 component subunit alpha, somatic form, mitochondrial; AltName: Full=PDHE1-A type I; Flags: Precursor |
| gi47117306 | RPS19 | 2 | 1.197082231 | 1.197082231 | 0.259522258 | RecName: Full=40S ribosomal protein S19 |
| gi48428722 | YWHAG | 13 | 1.195911878 | 1.195911878 | 0.258111087 | RecName: Full=14-3-3 protein gamma; Contains: RecName: Full=14-3-3 protein gamma, N-terminally processed |
| gi73920803 | SYN2 | 13 | 1.195781187 | 1.195781187 | 0.257953418 | RecName: Full=Synapsin-2; AltName: Full=Synapsin II |
| gi313104120 | ITPR1 | 2 | 1.195481925 | 1.195481925 | 0.257592317 | RecName: Full=Inositol 1,4,5-trisphosphate receptor type 1; AltName: Full=IP3 receptor isoform 1; Short=IP3R 1; Short=InsP3R1; AltName: Full=Inositol 1,4,5-trisphosphate-binding protein P400; AltName: Full=Protein PCD-6; AltName: Full=Purkinje cell protein 1; AltName: Full=Type 1 inositol 1,4,5-trisphosphate receptor; Short=Type 1 InsP3 receptor |
| gi114978 | SEPT4 | 4 | 1.189916668 | 1.189916668 | 0.250860543 | RecName: Full=Septin-4; AltName: Full=Brain protein H5; AltName: Full=Peanut-like protein 2 |
| gi47117658 | ARF3 | 7 | 1.18917473 | 1.18917473 | 0.249960711 | RecName: Full=ADP-ribosylation factor 3 |
| gi94730370 | CTNNA2 | 2 | 1.188718216 | 1.188718216 | 0.249406767 | RecName: Full=Catenin alpha-2; AltName: Full=Alpha N-catenin |
| gi150438864 | CADM2 | 5 | 1.188645366 | 1.188645366 | 0.249318349 | RecName: Full=Cell adhesion molecule 2; AltName: Full=Immunoglobulin superfamily member 4D; Short=IgSF4D; AltName: Full=Nectin-like protein 3; Short=NECL-3; Flags: Precursor |
| gi78103425 | WASF1 | 3 | 1.18853099 | 1.18853099 | 0.249179521 | RecName: Full=Wiskott-Aldrich syndrome protein family member 1; Short=WASP family protein member 1; AltName: Full=Protein WAVE-1 |
| gi42559891 | SRCIN1 | 3 | 1.187826056 | 1.187826056 | 0.248323584 | RecName: Full=SRC kinase signaling inhibitor 1; AltName: Full=SNAP-25-interacting protein; Short=SNIP; AltName: Full=p130Cas-associated protein; AltName: Full=p140Cap |
| gi68566306 | DNM1L | 7 | 1.187275246 | 1.187275246 | 0.247654434 | RecName: Full=Dynamin-1-like protein; AltName: Full=Dynamin family member proline-rich carboxyl-terminal domain less; Short=Dymple; AltName: Full=Dynamin-related protein 1 |
| gi117502 | CALR | 6 | 1.187081915 | 1.187081915 | 0.247419492 | RecName: Full=Calreticulin; AltName: Full=CRP55; AltName: Full=Calregulin; AltName: Full=Endoplasmic reticulum resident protein 60; Short=ERp60; AltName: Full=HACBP; Flags: Precursor |
| gi341942067 | CTTN | 2 | 1.186233968 | 1.186233968 | 0.246388589 | RecName: Full=Src substrate cortactin |
| gi61216668 | RTN1 (includes EG:104001) | 6 | 1.184480697 | 1.184480697 | 0.244254687 | RecName: Full=Reticulon-1; AltName: Full=Neuroendocrine-specific protein |
| gi47117649 | ACTR2 | 3 | 1.184408327 | 1.184408327 | 0.244166539 | RecName: Full=Actin-related protein 2; AltName: Full=Actin-like protein 2 |
| gi10719868 | ADD1 | 8 | 1.183320548 | 1.183320548 | 0.242840936 | RecName: Full=Alpha-adducin; AltName: Full=Erythrocyte adducin subunit alpha |
| gi52001076 | MAPK1 | 7 | 1.183281505 | 1.183281505 | 0.242793335 | RecName: Full=Mitogen-activated protein kinase 1; Short=MAP kinase 1; Short=MAPK 1; AltName: Full=ERT1; AltName: Full=Extracellular signal-regulated kinase 2; Short=ERK-2; AltName: Full=MAP kinase isoform p42; Short=p42-MAPK; AltName: Full=Mitogen-activated protein kinase 2; Short=MAP kinase 2; Short=MAPK 2 |
| gi22653628 | AKR1A1 | 2 | 1.183245172 | 1.183245172 | 0.242749035 | RecName: Full=Alcohol dehydrogenase [NADP+]; AltName: Full=Aldehyde reductase; AltName: Full=Aldo-keto reductase family 1 member A1 |
| gi12230747 | WDR1 | 2 | 1.181041473 | 1.181041473 | 0.240059627 | RecName: Full=WD repeat-containing protein 1; AltName: Full=Actin-interacting protein 1; Short=AIP1 |
| gi30316201 | HNRNPA3 | 2 | 1.176022508 | 1.176022508 | 0.233915672 | RecName: Full=Heterogeneous nuclear ribonucleoprotein A3; Short=hnRNP A3 |
| gi46396509 | PDHB | 7 | 1.175206486 | 1.175206486 | 0.232914263 | RecName: Full=Pyruvate dehydrogenase E1 component subunit beta, mitochondrial; Short=PDHE1-B; Flags: Precursor |
| gi20137942 | C14orf166 | 2 | 1.172352647 | 1.172352647 | 0.229406601 | RecName: Full=UPF0568 protein C14orf166 homolog |
| gi20138335 | GNG12 | 4 | 1.171371714 | 1.171371714 | 0.228198962 | RecName: Full=Guanine nucleotide-binding protein G(I)/G(S)/G(O) subunit gamma-12; Flags: Precursor |
| gi54037693 | PRKCG | 8 | 1.171027706 | 1.171027706 | 0.22777521 | RecName: Full=Protein kinase C gamma type; Short=PKC-gamma |
| gi47117909 | RPL15 | 2 | 1.16672429 | 1.16672429 | 0.222463676 | RecName: Full=60S ribosomal protein L15 |
| gi15214055 | EIF4H | 2 | 1.165344613 | 1.165344613 | 0.220756648 | RecName: Full=Eukaryotic translation initiation factor 4H; Short=eIF-4H; AltName: Full=Williams-Beuren syndrome chromosomal region 1 protein homolog |
| gi51338706 | ATP6V1B2 | 17 | 1.164795643 | 1.164795643 | 0.220076865 | RecName: Full=V-type proton ATPase subunit B, brain isoform; Short=V-ATPase subunit B 2; AltName: Full=Endomembrane proton pump 58 kDa subunit; AltName: Full=Vacuolar proton pump subunit B 2 |
| gi38258917 | RAB5C | 3 | 1.164278601 | 1.164278601 | 0.219436323 | RecName: Full=Ras-related protein Rab-5C |
| gi52000885 | YWHAZ | 15 | 1.164030132 | 1.164030132 | 0.219128404 | RecName: Full=14-3-3 protein zeta/delta; AltName: Full=Protein kinase C inhibitor protein 1; Short=KCIP-1; AltName: Full=SEZ-2 |
| gi1352004 | ATP1B2 | 2 | 1.163687158 | 1.163687158 | 0.21870326 | RecName: Full=Sodium/potassium-transporting ATPase subunit beta-2; AltName: Full=AMOG; AltName: Full=Glial cell adhesion molecule; AltName: Full=Sodium/potassium-dependent ATPase subunit beta-2 |
| gi81873664 | SV2B | 4 | 1.163393175 | 1.163393175 | 0.218338746 | RecName: Full=Synaptic vesicle glycoprotein 2B; Short=Synaptic vesicle protein 2B |
| gi81881569 | SH2D4A | 2 | 1.160803348 | 1.160803348 | 0.215123586 | RecName: Full=SH2 domain-containing protein 4A |
| gi2500577 | PCBP2 | 3 | 1.160371337 | 1.160371337 | 0.214586564 | RecName: Full=Poly(rC)-binding protein 2; AltName: Full=Alpha-CP2; AltName: Full=CTBP; Short=CBP; AltName: Full=Putative heterogeneous nuclear ribonucleoprotein X; Short=hnRNP X |
| gi124028629 | HNRNPA2B1 | 11 | 1.159389795 | 1.159389795 | 0.213365693 | RecName: Full=Heterogeneous nuclear ribonucleoproteins A2/B1; Short=hnRNP A2/B1 |
| gi341941063 | HK1 | 25 | 1.15923674 | 1.15923674 | 0.213175225 | RecName: Full=Hexokinase-1; AltName: Full=Hexokinase type I; Short=HK I; AltName: Full=Hexokinase, tumor isozyme |
| gi60392581 | KCNAB2 | 2 | 1.156637066 | 1.156637066 | 0.209936241 | RecName: Full=Voltage-gated potassium channel subunit beta-2; AltName: Full=K(+) channel subunit beta-2; AltName: Full=Kv-beta-2; AltName: Full=Neuroimmune protein F5 |
| gi50401037 | ENDOD1 | 2 | 1.156014894 | 1.156014894 | 0.209159986 | RecName: Full=Endonuclease domain-containing 1 protein; Flags: Precursor |
| gi22653715 | DPP6 | 2 | 1.155874502 | 1.155874502 | 0.208984767 | RecName: Full=Dipeptidyl aminopeptidase-like protein 6; AltName: Full=DPPX; AltName: Full=Dipeptidyl aminopeptidase-related protein; AltName: Full=Dipeptidyl peptidase 6; AltName: Full=Dipeptidyl peptidase IV-like protein; AltName: Full=Dipeptidyl peptidase VI; Short=DPP VI |
| gi128101 | GAP43 | 5 | 1.155341789 | 1.155341789 | 0.208319712 | RecName: Full=Neuromodulin; AltName: Full=Axonal membrane protein GAP-43; AltName: Full=Calmodulin-binding protein P-57; AltName: Full=Growth-associated protein 43 |
| gi46577116 | RAB1B | 4 | 1.154814412 | 1.154814412 | 0.207661018 | RecName: Full=Ras-related protein Rab-1B |
| gi1350822 | HNRNPA1 | 4 | 1.153831098 | 1.153831098 | 0.206432052 | RecName: Full=Heterogeneous nuclear ribonucleoprotein A1; Short=hnRNP A1; AltName: Full=HDP-1; AltName: Full=Helix-destabilizing protein; AltName: Full=Single-strand-binding protein; AltName: Full=Topoisomerase-inhibitor suppressed; AltName: Full=hnRNP core protein A1 |
| gi48474314 | RAB5B | 2 | 1.15360005 | 1.15360005 | 0.206143132 | RecName: Full=Ras-related protein Rab-5B |
| gi21431839 | RPH3A | 3 | 1.150624008 | 1.150624008 | 0.202416478 | RecName: Full=Rabphilin-3A; AltName: Full=Exophilin-1 |
| gi81898160 | DNM3 | 12 | 1.149230668 | 1.149230668 | 0.200668397 | RecName: Full=Dynamin-3 |
| gi46395721 | ACTN1 | 14 | 1.145458316 | 1.145458316 | 0.195924959 | RecName: Full=Alpha-actinin-1; AltName: Full=Alpha-actinin cytoskeletal isoform; AltName: Full=F-actin cross-linking protein; AltName: Full=Non-muscle alpha-actinin-1 |
| gi52788305 | SUCLA2 | 6 | 1.145192708 | 1.145192708 | 0.195590389 | RecName: Full=Succinyl-CoA ligase [ADP-forming] subunit beta, mitochondrial; AltName: Full=ATP-specific succinyl-CoA synthetase subunit beta; AltName: Full=Succinyl-CoA synthetase beta-A chain; Short=SCS-betaA; Flags: Precursor |
| gi54039385 | RPS18 | 4 | 1.144956611 | 1.144956611 | 0.195292928 | RecName: Full=40S ribosomal protein S18; AltName: Full=Ke-3; Short=Ke3 |
| gi2495231 | HINT1 | 3 | 1.144677389 | 1.144677389 | 0.194941053 | RecName: Full=Histidine triad nucleotide-binding protein 1; AltName: Full=Adenosine 5'-monophosphoramidase; AltName: Full=Protein kinase C inhibitor 1; AltName: Full=Protein kinase C-interacting protein 1; Short=PKCI-1 |
| gi113334 | AP2A1 | 12 | 1.144512712 | 1.144512712 | 0.194733486 | RecName: Full=AP-2 complex subunit alpha-1; AltName: Full=100 kDa coated vesicle protein A; AltName: Full=Adapter-related protein complex 2 alpha-1 subunit; AltName: Full=Adaptor protein complex AP-2 subunit alpha-1; AltName: Full=Alpha-adaptin A; AltName: Full=Alpha1-adaptin; AltName: Full=Clathrin assembly protein complex 2 alpha-A large chain; AltName: Full=Plasma membrane adaptor HA2/AP2 adaptin alpha A subunit |
| gi51316976 | AP2S1 | 4 | 1.143762404 | 1.143762404 | 0.19378739 | RecName: Full=AP-2 complex subunit sigma; AltName: Full=Adapter-related protein complex 2 sigma subunit; AltName: Full=Adaptor protein complex AP-2 subunit sigma; AltName: Full=Clathrin assembly protein 2 small chain; AltName: Full=Clathrin coat assembly protein AP17; AltName: Full=Clathrin coat-associated protein AP17; AltName: Full=Plasma membrane adaptor AP-2 17 kDa protein; AltName: Full=Sigma-adaptin 3b; AltName: Full=Sigma2-adaptin |
| gi341940668 | EXOC5 | 2 | 1.142628263 | 1.142628263 | 0.19235612 | RecName: Full=Exocyst complex component 5; AltName: Full=Exocyst complex component Sec10 |
| gi145559476 | GLUL | 11 | 1.141290159 | 1.141290159 | 0.190665626 | RecName: Full=Glutamine synthetase; Short=GS; AltName: Full=Glutamate decarboxylase; AltName: Full=Glutamate--ammonia ligase |
| gi94730407 | PCLO | 2 | 1.141213536 | 1.141213536 | 0.190568764 | RecName: Full=Protein piccolo; AltName: Full=Aczonin; AltName: Full=Brain-derived HLMN protein; AltName: Full=Multidomain presynaptic cytomatrix protein |
| gi21363012 | TF | 2 | 1.140675676 | 1.140675676 | 0.189888654 | RecName: Full=Serotransferrin; Short=Transferrin; AltName: Full=Beta-1 metal-binding globulin; AltName: Full=Siderophilin; Flags: Precursor |
| gi52000963 | GNAS | 3 | 1.140383061 | 1.140383061 | 0.189518515 | RecName: Full=Guanine nucleotide-binding protein G(s) subunit alpha isoforms short; AltName: Full=Adenylate cyclase-stimulating G alpha protein |
| gi341940470 | DNAH5 | 3 | 1.139894152 | 1.139894152 | 0.188899865 | RecName: Full=Dynein heavy chain 5, axonemal; AltName: Full=Axonemal beta dynein heavy chain 5; Short=mDNAH5; AltName: Full=Ciliary dynein heavy chain 5 |
| gi67460546 | CPNE4 | 2 | 1.137995791 | 1.137995791 | 0.186495221 | RecName: Full=Copine-4; AltName: Full=Copine IV |
| gi8134322 | ATP8A1 | 3 | 1.13676643 | 1.13676643 | 0.184935857 | RecName: Full=Probable phospholipid-transporting ATPase IA; AltName: Full=ATPase class I type 8A member 1; AltName: Full=Chromaffin granule ATPase II |
| gi81879780 | SNCB | 6 | 1.135508242 | 1.135508242 | 0.183338178 | RecName: Full=Beta-synuclein |
| gi46577103 | RAB14 | 2 | 1.135077249 | 1.135077249 | 0.182790485 | RecName: Full=Ras-related protein Rab-14 |
| gi20141319 | GNAZ | 2 | 1.134386345 | 1.134386345 | 0.181912072 | RecName: Full=Guanine nucleotide-binding protein G(z) subunit alpha; AltName: Full=G(x) alpha chain; AltName: Full=Gz-alpha |
| gi22096313 | GRIA2 | 2 | 1.134159319 | 1.134159319 | 0.181623315 | RecName: Full=Glutamate receptor 2; Short=GluR-2; AltName: Full=AMPA-selective glutamate receptor 2; AltName: Full=GluR-B; AltName: Full=GluR-K2; AltName: Full=Glutamate receptor ionotropic, AMPA 2; Short=GluA2; Flags: Precursor |
| gi182636954 | SLC12A5 | 7 | 1.133167674 | 1.133167674 | 0.180361351 | RecName: Full=Solute carrier family 12 member 5; AltName: Full=Electroneutral potassium-chloride cotransporter 2; AltName: Full=K-Cl cotransporter 2; Short=mKCC2; AltName: Full=Neuronal K-Cl cotransporter |
| gi1174545 | SYT1 (includes EG:20979) | 11 | 1.132114331 | 1.132114331 | 0.179019662 | RecName: Full=Synaptotagmin-1; AltName: Full=Synaptotagmin I; Short=SytI; AltName: Full=p65 |
| gi143811473 | ATP6V1E1 | 7 | 1.131649914 | 1.131649914 | 0.178427717 | RecName: Full=V-type proton ATPase subunit E 1; Short=V-ATPase subunit E 1; AltName: Full=V-ATPase 31 kDa subunit; Short=p31; AltName: Full=Vacuolar proton pump subunit E 1 |
| gi22095581 | DCTN2 | 3 | 1.130752002 | 1.130752002 | 0.17728255 | RecName: Full=Dynactin subunit 2; AltName: Full=50 kDa dynein-associated polypeptide; AltName: Full=Dynactin complex 50 kDa subunit; Short=DCTN-50; AltName: Full=Growth cone membrane protein 23-48K; Short=GMP23-48K; AltName: Full=p50 dynamitin |
| gi20140777 | UQCRQ | 3 | 1.129789069 | 1.129789069 | 0.176053447 | RecName: Full=Cytochrome b-c1 complex subunit 8; AltName: Full=Complex III subunit 8; AltName: Full=Complex III subunit VIII; AltName: Full=Ubiquinol-cytochrome c reductase complex 9.5 kDa protein; AltName: Full=Ubiquinol-cytochrome c reductase complex ubiquinone-binding protein QP-C |
| gi20141407 | SLC1A3 | 4 | 1.128533802 | 1.128533802 | 0.174449632 | RecName: Full=Excitatory amino acid transporter 1; AltName: Full=Glial high affinity glutamate transporter; AltName: Full=High-affinity neuronal glutamate transporter; AltName: Full=Sodium-dependent glutamate/aspartate transporter 1; Short=GLAST-1; AltName: Full=Solute carrier family 1 member 3 |
| gi59797879 | DLG2 | 2 | 1.128497573 | 1.128497573 | 0.174403316 | RecName: Full=Disks large homolog 2; AltName: Full=Channel-associated protein of synapse-110; Short=Chapsyn-110; AltName: Full=Postsynaptic density protein PSD-93 |
| gi62286597 | DIRAS2 | 2 | 1.127550794 | 1.127550794 | 0.173192425 | RecName: Full=GTP-binding protein Di-Ras2; AltName: Full=Distinct subgroup of the Ras family member 2; Flags: Precursor |
| gi6093768 | VDAC2 | 4 | 1.126884012 | 1.126884012 | 0.17233903 | RecName: Full=Voltage-dependent anion-selective channel protein 2; Short=VDAC-2; Short=mVDAC2; AltName: Full=Outer mitochondrial membrane protein porin 2; AltName: Full=Voltage-dependent anion-selective channel protein 6; Short=VDAC-6; Short=mVDAC6 |
| gi78099820 | ATP5D | 4 | 1.126105647 | 1.126105647 | 0.171342181 | RecName: Full=ATP synthase subunit delta, mitochondrial; AltName: Full=F-ATPase delta subunit; Flags: Precursor |
| gi298286902 | NPTN | 6 | 1.125988393 | 1.125988393 | 0.171191956 | RecName: Full=Neuroplastin; AltName: Full=Stromal cell-derived receptor 1; Short=SDR-1; Flags: Precursor |
| gi3334247 | C1QBP | 2 | 1.125626169 | 1.125626169 | 0.170727774 | RecName: Full=Complement component 1 Q subcomponent-binding protein, mitochondrial; AltName: Full=GC1q-R protein; AltName: Full=Glycoprotein gC1qBP; Short=C1qBP; Flags: Precursor |
| gi55976655 | YWHAH | 10 | 1.124101302 | 1.124101302 | 0.168772054 | RecName: Full=14-3-3 protein eta |
| gi2500528 | DDX3X | 2 | 1.123138001 | 1.123138001 | 0.167535203 | RecName: Full=ATP-dependent RNA helicase DDX3X; AltName: Full=D1Pas1-related sequence 2; AltName: Full=DEAD box RNA helicase DEAD3; Short=mDEAD3; AltName: Full=DEAD box protein 3, X-chromosomal; AltName: Full=Embryonic RNA helicase |
| gi146345384 | PPP3R1 | 7 | 1.121000585 | 1.121000585 | 0.164787031 | RecName: Full=Calcineurin subunit B type 1; AltName: Full=Protein phosphatase 2B regulatory subunit 1; AltName: Full=Protein phosphatase 3 regulatory subunit B alpha isoform 1 |
| gi464569 | RAB3D | 5 | 1.11869233 | 1.11869233 | 0.161813312 | RecName: Full=Ras-related protein Rab-3D |
| gi462602 | MIF | 3 | 1.118560853 | 1.118560853 | 0.161643746 | RecName: Full=Macrophage migration inhibitory factor; Short=MIF; AltName: Full=Delayed early response protein 6; Short=DER6; AltName: Full=Glycosylation-inhibiting factor; Short=GIF; AltName: Full=L-dopachrome isomerase; AltName: Full=L-dopachrome tautomerase; AltName: Full=Phenylpyruvate tautomerase |
| gi1169460 | SLC1A2 | 13 | 1.116822357 | 1.116822357 | 0.159399728 | RecName: Full=Excitatory amino acid transporter 2; AltName: Full=GLT-1; AltName: Full=Sodium-dependent glutamate/aspartate transporter 2; AltName: Full=Solute carrier family 1 member 2 |
| gi32363497 | EZR | 2 | 1.114045018 | 1.114045018 | 0.155807532 | RecName: Full=Ezrin; AltName: Full=Cytovillin; AltName: Full=Villin-2; AltName: Full=p81 |
| gi76364169 | DDX17 | 3 | 1.113904806 | 1.113904806 | 0.155625946 | RecName: Full=Probable ATP-dependent RNA helicase DDX17; AltName: Full=DEAD box protein 17 |
| gi46397834 | RAB7A | 3 | 1.113608915 | 1.113608915 | 0.155242666 | RecName: Full=Ras-related protein Rab-7a |
| gi34222626 | SLC25A22 | 4 | 1.113502295 | 1.113502295 | 0.155104532 | RecName: Full=Mitochondrial glutamate carrier 1; Short=GC-1; AltName: Full=Glutamate/H(+) symporter 1; AltName: Full=Solute carrier family 25 member 22 |
| gi146325018 | DLAT | 4 | 1.113300583 | 1.113300583 | 0.154843162 | RecName: Full=Dihydrolipoyllysine-residue acetyltransferase component of pyruvate dehydrogenase complex, mitochondrial; AltName: Full=Dihydrolipoamide acetyltransferase component of pyruvate dehydrogenase complex; AltName: Full=Pyruvate dehydrogenase complex component E2; Short=PDC-E2; Short=PDCE2; Flags: Precursor |
| gi363805626 | SYGP1 | 6 | 1.112876809 | 1.112876809 | 0.1542939 | RecName: Full=Ras/Rap GTPase-activating protein SynGAP; AltName: Full=Neuronal RasGAP; AltName: Full=Synaptic Ras GTPase-activating protein 1; Short=Synaptic Ras-GAP 1 |
| gi20140091 | SFXN3 | 5 | 1.111277029 | 1.111277029 | 0.152218509 | RecName: Full=Sideroflexin-3 |
| gi6093647 | PAK1 | 6 | 1.111045779 | 1.111045779 | 0.151918262 | RecName: Full=Serine/threonine-protein kinase PAK 1; AltName: Full=Alpha-PAK; AltName: Full=CDC42/RAC effector kinase PAK-A; AltName: Full=p21-activated kinase 1; Short=PAK-1; AltName: Full=p65-PAK |
| gi51317407 | HPCA | 9 | 1.110393776 | 1.110393776 | 0.151071386 | RecName: Full=Neuron-specific calcium-binding protein hippocalcin |
| gi1709998 | RAB2A | 4 | 1.109337504 | 1.109337504 | 0.149698357 | RecName: Full=Ras-related protein Rab-2A |
| gi2829840 | ATP5J | 3 | 1.108886437 | 1.108886437 | 0.149111624 | RecName: Full=ATP synthase-coupling factor 6, mitochondrial; Short=ATPase subunit F6; Flags: Precursor |
| gi12585517 | ATP6V1G2 | 3 | 1.108755676 | 1.108755676 | 0.14894149 | RecName: Full=V-type proton ATPase subunit G 2; Short=V-ATPase subunit G 2; AltName: Full=V-ATPase 13 kDa subunit 2; AltName: Full=Vacuolar proton pump subunit G 2 |
| gi41016844 | AMPH | 13 | 1.108749605 | 1.108749605 | 0.14893359 | RecName: Full=Amphiphysin |
| gi341940935 | MAP2 | 26 | 1.108381544 | 1.108381544 | 0.148454593 | RecName: Full=Microtubule-associated protein 2; Short=MAP-2 |
| gi56404944 | PARK7 | 5 | 1.107447966 | 1.107447966 | 0.147238915 | RecName: Full=Protein DJ-1; AltName: Full=Parkinson disease protein 7 homolog; Flags: Precursor |
| gi146345462 | NDUFS3 | 8 | 1.107308057 | 1.107308057 | 0.14705664 | RecName: Full=NADH dehydrogenase [ubiquinone] iron-sulfur protein 3, mitochondrial; AltName: Full=Complex I-30kD; Short=CI-30kD; AltName: Full=NADH-ubiquinone oxidoreductase 30 kDa subunit; Flags: Precursor |
| gi417489 | PCMT1 | 4 | 1.107247812 | 1.107247812 | 0.146978146 | RecName: Full=Protein-L-isoaspartate(D-aspartate) O-methyltransferase; Short=PIMT; AltName: Full=L-isoaspartyl protein carboxyl methyltransferase; AltName: Full=Protein L-isoaspartyl/D-aspartyl methyltransferase; AltName: Full=Protein-beta-aspartate methyltransferase |
| gi3913897 | ATPIF1 | 3 | 1.107089341 | 1.107089341 | 0.146771651 | RecName: Full=ATPase inhibitor, mitochondrial; AltName: Full=Inhibitor of F(1)F(o)-ATPase; Short=IF(1); Short=IF1; Flags: Precursor |
| gi22653923 | PALM | 6 | 1.106520538 | 1.106520538 | 0.146030229 | RecName: Full=Paralemmin-1; AltName: Full=Paralemmin; Flags: Precursor |
| gi300669660 | NPEPPS | 2 | 1.106277093 | 1.106277093 | 0.145712788 | RecName: Full=Puromycin-sensitive aminopeptidase; Short=PSA; AltName: Full=Cytosol alanyl aminopeptidase; Short=AAP-S |
| gi81881319 | RUFY3 | 3 | 1.106173637 | 1.106173637 | 0.145577865 | RecName: Full=Protein RUFY3; AltName: Full=Rap2-interacting protein x; Short=RIPx; AltName: Full=Single axon-regulated protein; Short=Singar |
| gi146345470 | NSF | 31 | 1.106063864 | 1.106063864 | 0.145434689 | RecName: Full=Vesicle-fusing ATPase; AltName: Full=N-ethylmaleimide-sensitive fusion protein; Short=NEM-sensitive fusion protein; AltName: Full=Suppressor of K(+) transport growth defect 2; Short=Protein SKD2; AltName: Full=Vesicular-fusion protein NSF |
| gi347595817 | PRKCB | 3 | 1.105855491 | 1.105855491 | 0.145162872 | RecName: Full=Protein kinase C beta type; Short=PKC-B; Short=PKC-beta |
| gi13959400 | AK1 | 5 | 1.10336356 | 1.10336356 | 0.141908239 | RecName: Full=Adenylate kinase isoenzyme 1; Short=AK 1; AltName: Full=ATP-AMP transphosphorylase 1; AltName: Full=Myokinase |
| gi22256950 | PACSIN1 | 17 | 1.103304634 | 1.103304634 | 0.141831189 | RecName: Full=Protein kinase C and casein kinase substrate in neurons protein 1 |
| gi57015413 | NCKIPSD | 2 | 1.099920877 | 1.099920877 | 0.137399747 | RecName: Full=NCK-interacting protein with SH3 domain; AltName: Full=54 kDa VacA-interacting protein; Short=VIP54; AltName: Full=90 kDa N-WASP-interacting protein; AltName: Full=90 kDa SH3 protein interacting with Nck; AltName: Full=SH3 adapter protein SPIN90; AltName: Full=WASP-interacting SH3-domain protein; Short=WISH; AltName: Full=Wiskott-Aldrich syndrome protein-binding protein; Short=N-WASP-binding protein |
| gi31077176 | HSPA4L | 4 | 1.099543561 | 1.099543561 | 0.136904761 | RecName: Full=Heat shock 70 kDa protein 4L; AltName: Full=Heat shock 70-related protein APG-1; AltName: Full=Osmotic stress protein 94 |
| gi46577689 | RAB21 | 3 | 1.098697642 | 1.098697642 | 0.135794417 | RecName: Full=Ras-related protein Rab-21; AltName: Full=Rab-12; Flags: Precursor |
| gi22002044 | DNM2 | 8 | 1.097971639 | 1.097971639 | 0.134840789 | RecName: Full=Dynamin-2; AltName: Full=Dynamin UDNM |
| gi341940868 | KIF21B | 3 | 1.097884181 | 1.097884181 | 0.134725868 | RecName: Full=Kinesin-like protein KIF21B; AltName: Full=Kinesin-like protein KIF6 |
| gi81894342 | KIF27 | 2 | 1.097884181 | 1.097884181 | 0.134725868 | RecName: Full=Kinesin-like protein KIF27 |
| gi146345463 | NDUFV2 | 2 | 1.09784061 | 1.09784061 | 0.134668612 | RecName: Full=NADH dehydrogenase [ubiquinone] flavoprotein 2, mitochondrial; AltName: Full=NADH-ubiquinone oxidoreductase 24 kDa subunit; Flags: Precursor |
| gi81910611 | ACTR3B | 3 | 1.097152086 | 1.097152086 | 0.133763525 | RecName: Full=Actin-related protein 3B; AltName: Full=ARP3-beta; AltName: Full=Actin-like protein 3B |
| gi12643614 | ATP2A2 | 7 | 1.096178715 | 1.096178715 | 0.132483026 | RecName: Full=Sarcoplasmic/endoplasmic reticulum calcium ATPase 2; Short=SERCA2; Short=SR Ca(2+)-ATPase 2; AltName: Full=Calcium pump 2; AltName: Full=Calcium-transporting ATPase sarcoplasmic reticulum type, slow twitch skeletal muscle isoform; AltName: Full=Endoplasmic reticulum class 1/2 Ca(2+) ATPase |
| gi549059 | CCT3 | 2 | 1.094126841 | 1.094126841 | 0.129779997 | RecName: Full=T-complex protein 1 subunit gamma; Short=TCP-1-gamma; AltName: Full=CCT-gamma; AltName: Full=Matricin; AltName: Full=mTRiC-P5 |
| gi341941093 | FH | 4 | 1.0935489 | 1.0935489 | 0.129017735 | RecName: Full=Fumarate hydratase, mitochondrial; Short=Fumarase; AltName: Full=EF-3; Flags: Precursor |
| gi62510833 | DLST | 3 | 1.093143261 | 1.093143261 | 0.128482485 | RecName: Full=Dihydrolipoyllysine-residue succinyltransferase component of 2-oxoglutarate dehydrogenase complex, mitochondrial; AltName: Full=2-oxoglutarate dehydrogenase complex component E2; Short=OGDC-E2; AltName: Full=Dihydrolipoamide succinyltransferase component of 2-oxoglutarate dehydrogenase complex; AltName: Full=E2K; Flags: Precursor |
| gi66773932 | BASP1 | 8 | 1.092398492 | 1.092398492 | 0.127499228 | RecName: Full=Brain acid soluble protein 1; AltName: Full=22 kDa neuronal tissue-enriched acidic protein; AltName: Full=Neuronal axonal membrane protein NAP-22 |
| gi46577330 | HNRNPH2 | 5 | 1.090346664 | 1.090346664 | 0.124786897 | RecName: Full=Heterogeneous nuclear ribonucleoprotein H2; Short=hnRNP H2; AltName: Full=Heterogeneous nuclear ribonucleoprotein H'; Short=hnRNP H' |
| gi32172431 | DNM1 | 36 | 1.088566458 | 1.088566458 | 0.122429488 | RecName: Full=Dynamin-1 |
| gi46397808 | CORO1A | 2 | 1.08755583 | 1.08755583 | 0.121089464 | RecName: Full=Coronin-1A; AltName: Full=Coronin-like protein A; Short=Clipin-A; AltName: Full=Coronin-like protein p57; AltName: Full=Tryptophan aspartate-containing coat protein; Short=TACO |
| gi66773801 | CLTC | 59 | 1.086230545 | 1.086230545 | 0.119330338 | RecName: Full=Clathrin heavy chain 1 |
| gi14285350 | ATP2B2 | 12 | 1.085261134 | 1.085261134 | 0.118042223 | RecName: Full=Plasma membrane calcium-transporting ATPase 2; Short=PMCA2; AltName: Full=Plasma membrane calcium ATPase isoform 2; AltName: Full=Plasma membrane calcium pump isoform 2 |
| gi2506246 | SPTB | 6 | 1.084919486 | 1.084919486 | 0.117587981 | RecName: Full=Spectrin beta chain, erythrocyte; AltName: Full=Beta-I spectrin |
| gi51702252 | HSPD1 | 14 | 1.084845102 | 1.084845102 | 0.117489064 | RecName: Full=60 kDa heat shock protein, mitochondrial; AltName: Full=60 kDa chaperonin; AltName: Full=Chaperonin 60; Short=CPN60; AltName: Full=HSP-65; AltName: Full=Heat shock protein 60; Short=HSP-60; Short=Hsp60; AltName: Full=Mitochondrial matrix protein P1; Flags: Precursor |
| gi342187037 | TNR | 9 | 1.083628433 | 1.083628433 | 0.115870154 | RecName: Full=Tenascin-R; Short=TN-R; AltName: Full=Janusin; AltName: Full=Neural recognition molecule J1-160/180; AltName: Full=Restrictin; Flags: Precursor |
| gi46577661 | UBE2N | 4 | 1.08179429 | 1.08179429 | 0.113426188 | RecName: Full=Ubiquitin-conjugating enzyme E2 N; AltName: Full=Bendless-like ubiquitin-conjugating enzyme; AltName: Full=Ubc13; AltName: Full=Ubiquitin carrier protein N; AltName: Full=Ubiquitin-protein ligase N |
| gi1703188 | SLC25A6 | 12 | 1.080948525 | 1.080948525 | 0.112297823 | RecName: Full=ADP/ATP translocase 2; AltName: Full=ADP,ATP carrier protein 2; AltName: Full=Adenine nucleotide translocator 2; Short=ANT 2; AltName: Full=Solute carrier family 25 member 5 |
| gi146345422 | GPI | 15 | 1.080916991 | 1.080916991 | 0.112255736 | RecName: Full=Glucose-6-phosphate isomerase; Short=GPI; AltName: Full=Autocrine motility factor; Short=AMF; AltName: Full=Neuroleukin; Short=NLK; AltName: Full=Phosphoglucose isomerase; Short=PGI; AltName: Full=Phosphohexose isomerase; Short=PHI |
| gi81881914 | SV2A | 6 | 1.080912775 | 1.080912775 | 0.112250108 | RecName: Full=Synaptic vesicle glycoprotein 2A; Short=Synaptic vesicle protein 2; Short=Synaptic vesicle protein 2A; AltName: Full=Calcium regulator SV2A |
| gi60391212 | ACO2 (includes EG:11429) | 22 | 1.080886512 | 1.080886512 | 0.112215055 | RecName: Full=Aconitate hydratase, mitochondrial; Short=Aconitase; AltName: Full=Citrate hydro-lyase; Flags: Precursor |
| gi25453322 | VPS35 | 2 | 1.080217152 | 1.080217152 | 0.111321361 | RecName: Full=Vacuolar protein sorting-associated protein 35; AltName: Full=Maternal-embryonic 3; AltName: Full=Vesicle protein sorting 35 |
| gi29427667 | SIRPA | 4 | 1.079716658 | 1.079716658 | 0.110652766 | RecName: Full=Tyrosine-protein phosphatase non-receptor type substrate 1; Short=SHP substrate 1; Short=SHPS-1; AltName: Full=Brain Ig-like molecule with tyrosine-based activation motifs; Short=Bit; AltName: Full=CD172 antigen-like family member A; AltName: Full=Inhibitory receptor SHPS-1; AltName: Full=MyD-1 antigen; AltName: Full=Signal-regulatory protein alpha-1; Short=Sirp-alpha-1; Short=mSIRP-alpha1; AltName: Full=p84; AltName: CD_antigen=CD172a; Flags: Precursor |
| gi3023546 | COX6B1 | 4 | 1.079557208 | 1.079557208 | 0.110439697 | RecName: Full=Cytochrome c oxidase subunit 6B1; AltName: Full=Cytochrome c oxidase subunit VIb isoform 1; Short=COX VIb-1 |
| gi47116573 | ACTR3 | 5 | 1.076305527 | 1.076305527 | 0.106087668 | RecName: Full=Actin-related protein 3; AltName: Full=Actin-like protein 3 |
| gi81906185 | APOO | 2 | 1.076175067 | 1.076175067 | 0.105912787 | RecName: Full=Apolipoprotein O; AltName: Full=Protein FAM121B |
| gi67460489 | CAND1 | 6 | 1.07553481 | 1.07553481 | 0.105054219 | RecName: Full=Cullin-associated NEDD8-dissociated protein 1; AltName: Full=Cullin-associated and neddylation-dissociated protein 1; AltName: Full=p120 CAND1 |
| gi342187059 | TOMM70A | 2 | 1.074041263 | 1.074041263 | 0.10304942 | RecName: Full=Mitochondrial import receptor subunit TOM70; AltName: Full=Mitochondrial precursor proteins import receptor; AltName: Full=Translocase of outer membrane 70 kDa subunit |
| gi341941003 | MFN1 | 2 | 1.073563088 | 1.073563088 | 0.102406974 | RecName: Full=Mitofusin-1; AltName: Full=Transmembrane GTPase MFN1 |
| gi14548302 | UQCRC2 | 15 | 1.072543819 | 1.072543819 | 0.101036591 | RecName: Full=Cytochrome b-c1 complex subunit 2, mitochondrial; AltName: Full=Complex III subunit 2; AltName: Full=Core protein II; AltName: Full=Ubiquinol-cytochrome-c reductase complex core protein 2; Flags: Precursor |
| gi97537229 | SPTBN1 | 58 | 1.070473794 | 1.070473794 | 0.098249478 | RecName: Full=Spectrin beta chain, brain 1; AltName: Full=Beta-II spectrin; AltName: Full=Embryonic liver fodrin; AltName: Full=Fodrin beta chain; AltName: Full=Spectrin, non-erythroid beta chain 1 |
| gi547881 | GPM6A | 5 | 1.069880085 | 1.069880085 | 0.097449104 | RecName: Full=Neuronal membrane glycoprotein M6-a; Short=M6a |
| gi341940229 | AP1B1 | 10 | 1.069406038 | 1.069406038 | 0.096809728 | RecName: Full=AP-1 complex subunit beta-1; AltName: Full=Adapter-related protein complex 1 subunit beta-1; AltName: Full=Adaptor protein complex AP-1 subunit beta-1; AltName: Full=Beta-1-adaptin; AltName: Full=Beta-adaptin 1; AltName: Full=Clathrin assembly protein complex 1 beta large chain; AltName: Full=Golgi adaptor HA1/AP1 adaptin beta subunit |
| gi122066202 | SPTAN1 | 99 | 1.069039309 | 1.069039309 | 0.096314902 | RecName: Full=Spectrin alpha chain, brain; AltName: Full=Alpha-II spectrin; AltName: Full=Fodrin alpha chain; AltName: Full=Spectrin, non-erythroid alpha chain |
| gi21759130 | ARHGDIA | 4 | 1.067299411 | 1.067299411 | 0.093964954 | RecName: Full=Rho GDP-dissociation inhibitor 1; Short=Rho GDI 1; AltName: Full=GDI-1; AltName: Full=Rho-GDI alpha |
| gi97535655 | ANK1 | 3 | 1.067142873 | 1.067142873 | 0.093753342 | RecName: Full=Ankyrin-1; Short=ANK-1; AltName: Full=Erythrocyte ankyrin |
| gi108935831 | VCAN | 4 | 1.066742377 | 1.066742377 | 0.0932118 | RecName: Full=Versican core protein; AltName: Full=Chondroitin sulfate proteoglycan core protein 2; Short=Chondroitin sulfate proteoglycan 2; AltName: Full=Large fibroblast proteoglycan; AltName: Full=PG-M; Flags: Precursor |
| gi341941148 | GNAI2 | 5 | 1.064608393 | 1.064608393 | 0.090322846 | RecName: Full=Guanine nucleotide-binding protein G(i) subunit alpha-2; AltName: Full=Adenylate cyclase-inhibiting G alpha protein |
| gi145559539 | ATP6V1A | 17 | 1.064271157 | 1.064271157 | 0.08986577 | RecName: Full=V-type proton ATPase catalytic subunit A; Short=V-ATPase subunit A; AltName: Full=V-ATPase 69 kDa subunit; AltName: Full=Vacuolar proton pump subunit alpha |
| gi33112324 | HSPA12A | 11 | 1.064171919 | 1.064171919 | 0.08973124 | RecName: Full=Heat shock 70 kDa protein 12A |
| gi3024089 | LASP1 | 2 | 1.064027144 | 1.064027144 | 0.089534955 | RecName: Full=LIM and SH3 domain protein 1; Short=LASP-1; AltName: Full=Metastatic lymph node gene 50 protein; Short=MLN 50 |
| gi81870005 | NCDN | 14 | 1.06290164 | 1.06290164 | 0.088008097 | RecName: Full=Neurochondrin; AltName: Full=M-Sema F-associating protein of 75 kDa; AltName: Full=Norbin |
| gi60391192 | YWHAE | 14 | 1.062644966 | 1.062644966 | 0.087659667 | RecName: Full=14-3-3 protein epsilon; Short=14-3-3E |
| gi25089776 | ATP5H (includes EG:100039281) | 8 | 1.062075326 | 1.062075326 | 0.086886091 | RecName: Full=ATP synthase subunit d, mitochondrial; Short=ATPase subunit d |
| gi82592512 | NCKAP1 | 2 | 1.060961123 | 1.060961123 | 0.085371792 | RecName: Full=Nck-associated protein 1; Short=NAP 1; AltName: Full=Brain protein H19; AltName: Full=MH19; AltName: Full=Membrane-associated protein HEM-2; AltName: Full=p125Nap1 |
| gi416677 | ATP5A1 | 22 | 1.06051779 | 1.06051779 | 0.084768822 | RecName: Full=ATP synthase subunit alpha, mitochondrial; Flags: Precursor |
| gi116849 | CFL1 | 9 | 1.060287978 | 1.060287978 | 0.084456159 | RecName: Full=Cofilin-1; AltName: Full=Cofilin, non-muscle isoform |
| gi17432986 | EPB41L2 | 4 | 1.060159799 | 1.060159799 | 0.08428174 | RecName: Full=Band 4.1-like protein 2; AltName: Full=Generally expressed protein 4.1; Short=4.1G |
| gi21759079 | CYC1 | 4 | 1.059762294 | 1.059762294 | 0.083740703 | RecName: Full=Cytochrome c1, heme protein, mitochondrial; AltName: Full=Complex III subunit 4; AltName: Full=Complex III subunit IV; AltName: Full=Cytochrome b-c1 complex subunit 4; AltName: Full=Ubiquinol-cytochrome-c reductase complex cytochrome c1 subunit; Short=Cytochrome c-1; Flags: Precursor |
| gi126986 | PRDX3 | 2 | 1.059541315 | 1.059541315 | 0.083439844 | RecName: Full=Thioredoxin-dependent peroxide reductase, mitochondrial; AltName: Full=Antioxidant protein 1; Short=AOP-1; AltName: Full=PRX III; AltName: Full=Perioredoxin-3; AltName: Full=Protein MER5; Flags: Precursor |
| gi32363403 | NDUFB10 | 3 | 1.059500674 | 1.059500674 | 0.083384506 | RecName: Full=NADH dehydrogenase [ubiquinone] 1 beta subcomplex subunit 10; AltName: Full=Complex I-PDSW; Short=CI-PDSW; AltName: Full=NADH-ubiquinone oxidoreductase PDSW subunit |
| gi83305642 | SEPT5 | 8 | 1.059499112 | 1.059499112 | 0.083382378 | RecName: Full=Septin-5; AltName: Full=Cell division control-related protein 1; Short=CDCrel-1; AltName: Full=Peanut-like protein 1 |
| gi127982 | NME1 (includes EG:18102) | 6 | 1.059465397 | 1.059465397 | 0.083336468 | RecName: Full=Nucleoside diphosphate kinase A; Short=NDK A; Short=NDP kinase A; AltName: Full=Metastasis inhibition factor NM23; AltName: Full=NDPK-A; AltName: Full=Tumor metastatic process-associated protein; AltName: Full=nm23-M1 |
| gi46577639 | RAB10 | 3 | 1.058641732 | 1.058641732 | 0.082214432 | RecName: Full=Ras-related protein Rab-10 |
| gi20141789 | PRDX5 | 9 | 1.058061366 | 1.058061366 | 0.081423304 | RecName: Full=Peroxiredoxin-5, mitochondrial; AltName: Full=Antioxidant enzyme B166; Short=AOEB166; AltName: Full=Liver tissue 2D-page spot 2D-0014IV; AltName: Full=PLP; AltName: Full=Peroxiredoxin V; Short=Prx-V; AltName: Full=Peroxisomal antioxidant enzyme; AltName: Full=Thioredoxin peroxidase PMP20; AltName: Full=Thioredoxin reductase; Flags: Precursor |
| gi21903382 | SLC25A4 | 14 | 1.057510178 | 1.057510178 | 0.080671548 | RecName: Full=ADP/ATP translocase 1; AltName: Full=ADP,ATP carrier protein 1; AltName: Full=ADP,ATP carrier protein, heart/skeletal muscle isoform T1; AltName: Full=Adenine nucleotide translocator 1; Short=ANT 1; AltName: Full=Solute carrier family 25 member 4; AltName: Full=mANC1 |
| gi51316977 | AP2M1 | 9 | 1.057396239 | 1.057396239 | 0.0805161 | RecName: Full=AP-2 complex subunit mu; AltName: Full=AP-2 mu chain; AltName: Full=Adapter-related protein complex 2 mu subunit; AltName: Full=Adaptor protein complex AP-2 subunit mu; AltName: Full=Clathrin assembly protein complex 2 medium chain; AltName: Full=Clathrin coat assembly protein AP50; AltName: Full=Clathrin coat-associated protein AP50; AltName: Full=Mu2-adaptin; AltName: Full=Plasma membrane adaptor AP-2 50 kDa protein |
| gi13638404 | RAB6A | 3 | 1.057260726 | 1.057260726 | 0.080331197 | RecName: Full=Ras-related protein Rab-6A; Short=Rab-6 |
| gi341940297 | CAB39 | 2 | 1.056451408 | 1.056451408 | 0.079226412 | RecName: Full=Calcium-binding protein 39; AltName: Full=MO25alpha; AltName: Full=Protein Mo25 |
| gi21263432 | ATP5C1 | 3 | 1.056448702 | 1.056448702 | 0.079222716 | RecName: Full=ATP synthase subunit gamma, mitochondrial; AltName: Full=F-ATPase gamma subunit; Flags: Precursor |
| gi52782789 | FAM49B | 4 | 1.055584685 | 1.055584685 | 0.078042325 | RecName: Full=Protein FAM49B |
| gi146345428 | GPD2 | 9 | 1.054601394 | 1.054601394 | 0.076697809 | RecName: Full=Glycerol-3-phosphate dehydrogenase, mitochondrial; Short=GPD-M; Short=GPDH-M; AltName: Full=Protein TISP38; Flags: Precursor |
| gi30316333 | GPM6B | 4 | 1.053242389 | 1.053242389 | 0.07483749 | RecName: Full=Neuronal membrane glycoprotein M6-b; Short=M6b |
| gi20454881 | DBN1 | 7 | 1.051981464 | 1.051981464 | 0.073109285 | RecName: Full=Drebrin; AltName: Full=Developmentally-regulated brain protein |
| gi119348 | ENO2 | 18 | 1.051343555 | 1.051343555 | 0.072234186 | RecName: Full=Gamma-enolase; AltName: Full=2-phospho-D-glycerate hydro-lyase; AltName: Full=Enolase 2; AltName: Full=Neural enolase; AltName: Full=Neuron-specific enolase; Short=NSE |
| gi29427692 | IMMT | 9 | 1.048967423 | 1.048967423 | 0.068969874 | RecName: Full=Mitochondrial inner membrane protein; AltName: Full=Mitofilin |
| gi2493662 | HSPE1 | 7 | 1.046893421 | 1.046893421 | 0.066114576 | RecName: Full=10 kDa heat shock protein, mitochondrial; Short=Hsp10; AltName: Full=10 kDa chaperonin; AltName: Full=Chaperonin 10; Short=CPN10 |
| gi121747 | GSTP1 (includes others)* | 4 | 1.04414224 | 1.04414224 | 0.062318259 | RecName: Full=Glutathione S-transferase P 1; Short=Gst P1; AltName: Full=GST YF-YF; AltName: Full=GST class-pi; AltName: Full=GST-piB; AltName: Full=Preadipocyte growth factor |
| gi51316454 | HPCAL4 | 6 | 1.0437167 | 1.0437167 | 0.061730169 | RecName: Full=Hippocalcin-like protein 4; AltName: Full=Neural visinin-like protein 2; Short=NVP-2 |
| gi94717662 | NDUFB6 | 2 | 1.043637734 | 1.043637734 | 0.061621013 | RecName: Full=NADH dehydrogenase [ubiquinone] 1 beta subcomplex subunit 6; AltName: Full=Complex I-B17; Short=CI-B17; AltName: Full=NADH-ubiquinone oxidoreductase B17 subunit |
| gi400275 | MAP2K1 | 4 | 1.042677189 | 1.042677189 | 0.060292571 | RecName: Full=Dual specificity mitogen-activated protein kinase kinase 1; Short=MAP kinase kinase 1; Short=MAPKK 1; AltName: Full=ERK activator kinase 1; AltName: Full=MAPK/ERK kinase 1; Short=MEK 1 |
| gi54036535 | SKP1/SKP1P2 | 5 | 1.04095656 | 1.04095656 | 0.057909865 | RecName: Full=S-phase kinase-associated protein 1; AltName: Full=Cyclin-A/CDK2-associated protein p19; AltName: Full=S-phase kinase-associated protein 1A; AltName: Full=p19A; AltName: Full=p19skp1 |
| gi49065658 | TIMM10 | 2 | 1.040300114 | 1.040300114 | 0.056999789 | RecName: Full=Mitochondrial import inner membrane translocase subunit Tim10 |
| gi122065442 | MAP1A | 21 | 1.03973954 | 1.03973954 | 0.056222171 | RecName: Full=Microtubule-associated protein 1A; Short=MAP-1A; Contains: RecName: Full=MAP1 light chain LC2 |
| gi342187144 | ATP6V0A1 | 10 | 1.03876165 | 1.03876165 | 0.054864658 | RecName: Full=V-type proton ATPase 116 kDa subunit a isoform 1; Short=V-ATPase 116 kDa isoform a1; AltName: Full=Clathrin-coated vesicle/synaptic vesicle proton pump 116 kDa subunit; AltName: Full=Vacuolar adenosine triphosphatase subunit Ac116; AltName: Full=Vacuolar proton pump subunit 1; AltName: Full=Vacuolar proton translocating ATPase 116 kDa subunit a isoform 1 |
| gi47605546 | ATP5O | 5 | 1.038437947 | 1.038437947 | 0.054415009 | RecName: Full=ATP synthase subunit O, mitochondrial; AltName: Full=Oligomycin sensitivity conferral protein; Short=OSCP; Flags: Precursor |
| gi54040727 | FASN | 5 | 1.038384405 | 1.038384405 | 0.054340621 | RecName: Full=Fatty acid synthase; Includes: RecName: Full=[Acyl-carrier-protein] S-acetyltransferase; Includes: RecName: Full=[Acyl-carrier-protein] S-malonyltransferase; Includes: RecName: Full=3-oxoacyl-[acyl-carrier-protein] synthase; Includes: RecName: Full=3-oxoacyl-[acyl-carrier-protein] reductase; Includes: RecName: Full=3-hydroxypalmitoyl-[acyl-carrier-protein] dehydratase; Includes: RecName: Full=Enoyl-[acyl-carrier-protein] reductase; Includes: RecName: Full=Oleoyl-[acyl-carrier-protein] hydrolase |
| gi23503090 | NDUFA4 | 4 | 1.03832342 | 1.03832342 | 0.054255888 | RecName: Full=NADH dehydrogenase [ubiquinone] 1 alpha subcomplex subunit 4; AltName: Full=Complex I-MLRQ; Short=CI-MLRQ; AltName: Full=NADH-ubiquinone oxidoreductase MLRQ subunit |
| gi2498751 | PEA15 | 3 | 1.037869368 | 1.037869368 | 0.05362487 | RecName: Full=Astrocytic phosphoprotein PEA-15; AltName: Full=15 kDa phosphoprotein enriched in astrocytes |
| gi6093770 | VDAC3 | 6 | 1.037430405 | 1.037430405 | 0.053014558 | RecName: Full=Voltage-dependent anion-selective channel protein 3; Short=VDAC-3; Short=mVDAC3; AltName: Full=Outer mitochondrial membrane protein porin 3 |
| gi113607 | ALDOA | 18 | 1.035871306 | 1.035871306 | 0.050844777 | RecName: Full=Fructose-bisphosphate aldolase A; AltName: Full=Aldolase 1; AltName: Full=Muscle-type aldolase |
| gi146345481 | PGK1 | 16 | 1.034539465 | 1.034539465 | 0.048988682 | RecName: Full=Phosphoglycerate kinase 1 |
| gi78099814 | ATP6V1H | 7 | 1.033078642 | 1.033078642 | 0.046950081 | RecName: Full=V-type proton ATPase subunit H; Short=V-ATPase subunit H; AltName: Full=Vacuolar proton pump subunit H |
| gi81902298 | BRK1 | 2 | 1.032631612 | 1.032631612 | 0.04632567 | RecName: Full=Protein BRICK1; Short=BRK1 |
| gi267190 | UBA1 | 14 | 1.032375628 | 1.032375628 | 0.045967989 | RecName: Full=Ubiquitin-like modifier-activating enzyme 1; AltName: Full=Ubiquitin-activating enzyme E1; AltName: Full=Ubiquitin-activating enzyme E1 X; AltName: Full=Ubiquitin-like modifier-activating enzyme 1 X |
| gi20141656 | STX1A | 7 | 1.031376196 | 1.031376196 | 0.044570653 | RecName: Full=Syntaxin-1A; AltName: Full=Neuron-specific antigen HPC-1 |
| gi146345480 | PDIA3 | 7 | 1.030773372 | 1.030773372 | 0.043727173 | RecName: Full=Protein disulfide-isomerase A3; AltName: Full=58 kDa glucose-regulated protein; AltName: Full=58 kDa microsomal protein; Short=p58; AltName: Full=Disulfide isomerase ER-60; AltName: Full=Endoplasmic reticulum resident protein 57; Short=ER protein 57; Short=ERp57; AltName: Full=Endoplasmic reticulum resident protein 60; Short=ER protein 60; Short=ERp60; Flags: Precursor |
| gi52782750 | ATP5L | 2 | 1.029937669 | 1.029937669 | 0.042557029 | RecName: Full=ATP synthase subunit g, mitochondrial; Short=ATPase subunit g |
| gi9978489 | HAPLN1 | 2 | 1.028081597 | 1.028081597 | 0.039954773 | RecName: Full=Hyaluronan and proteoglycan link protein 1; AltName: Full=Cartilage-linking protein 1; Short=Cartilage-link protein; AltName: Full=Proteoglycan link protein; Flags: Precursor |
| gi338817898 | GOT1 | 13 | 1.026495243 | 1.026495243 | 0.037726942 | RecName: Full=Aspartate aminotransferase, cytoplasmic; AltName: Full=Glutamate oxaloacetate transaminase 1; AltName: Full=Transaminase A |
| gi3041732 | SOD2 | 6 | 1.026220882 | 1.026220882 | 0.037341287 | RecName: Full=Superoxide dismutase [Mn], mitochondrial; Flags: Precursor |
| gi73920802 | SYN1 | 24 | 1.025070537 | 1.025070537 | 0.035723188 | PRKACB |
| gi45476974 | DDAH1 | 2 | 1.024845167 | 1.024845167 | 0.035405965 | RecName: Full=N(G),N(G)-dimethylarginine dimethylaminohydrolase 1; Short=DDAH-1; Short=Dimethylarginine dimethylaminohydrolase 1; AltName: Full=DDAHI; AltName: Full=Dimethylargininase-1 |
| gi32363396 | NDUFA2 | 2 | 1.023995103 | 1.023995103 | 0.034208816 | RecName: Full=NADH dehydrogenase [ubiquinone] 1 alpha subcomplex subunit 2; AltName: Full=Complex I-B8; Short=CI-B8; AltName: Full=NADH-ubiquinone oxidoreductase B8 subunit |
| gi52782731 | PPP2R1A | 9 | 1.023317284 | 1.023317284 | 0.033253528 | RecName: Full=Serine/threonine-protein phosphatase 2A 65 kDa regulatory subunit A alpha isoform; AltName: Full=PP2A subunit A isoform PR65-alpha; AltName: Full=PP2A subunit A isoform R1-alpha |
| gi118105 | PPIA | 11 | 1.020659731 | 1.020659731 | 0.029501978 | RecName: Full=Peptidyl-prolyl cis-trans isomerase A; Short=PPIase A; AltName: Full=Cyclophilin A; AltName: Full=Cyclosporin A-binding protein; AltName: Full=Rotamase A; AltName: Full=SP18 |
| gi28376967 | CEND1 | 3 | 1.019608367 | 1.019608367 | 0.028015117 | RecName: Full=Cell cycle exit and neuronal differentiation protein 1; AltName: Full=BM88 antigen |
| gi52783422 | SLC38A3 | 2 | 1.019281651 | 1.019281651 | 0.027552757 | RecName: Full=Sodium-coupled neutral amino acid transporter 3; AltName: Full=N-system amino acid transporter 1; AltName: Full=Na(+)-coupled neutral amino acid transporter 3; AltName: Full=Solute carrier family 38 member 3; Short=mNAT; AltName: Full=System N amino acid transporter 1 |
| gi116918 | CNTN1 | 16 | 1.018667355 | 1.018667355 | 0.026683017 | RecName: Full=Contactin-1; AltName: Full=Neural cell surface protein F3; Flags: Precursor |
| gi1345668 | CAPZB | 2 | 1.018504825 | 1.018504825 | 0.026452814 | RecName: Full=F-actin-capping protein subunit beta; AltName: Full=CapZ beta |
| gi160406731 | SH3GL2 | 11 | 1.018112626 | 1.018112626 | 0.025897164 | RecName: Full=Endophilin-A1; AltName: Full=Endophilin-1; AltName: Full=SH3 domain protein 2A; AltName: Full=SH3 domain-containing GRB2-like protein 2; AltName: Full=SH3p4 |
| gi51338697 | VSNL1 | 8 | 1.016809073 | 1.016809073 | 0.024048809 | RecName: Full=Visinin-like protein 1; Short=VILIP; AltName: Full=Neural visinin-like protein 1; Short=NVL-1; Short=NVP-1 |
| gi2495342 | HSPA4 | 8 | 1.014946276 | 1.014946276 | 0.021403364 | RecName: Full=Heat shock 70 kDa protein 4; AltName: Full=Heat shock 70-related protein APG-2 |
| gi231557 | APOA1 | 2 | 1.011047092 | 1.011047092 | 0.015850196 | RecName: Full=Apolipoprotein A-I; Short=Apo-AI; Short=ApoA-I; AltName: Full=Apolipoprotein A1; Flags: Precursor |
| gi1346207 | GSTM3 | 5 | 1.010393535 | 1.010393535 | 0.014917313 | RecName: Full=Glutathione S-transferase Mu 5; AltName: Full=Fibrous sheath component 2; Short=Fsc2; AltName: Full=GST class-mu 5 |
| gi20178035 | PGAM1 | 8 | 1.010278628 | 1.010278628 | 0.014753233 | RecName: Full=Phosphoglycerate mutase 1; AltName: Full=BPG-dependent PGAM 1; AltName: Full=Phosphoglycerate mutase isozyme B; Short=PGAM-B |
| gi122066700 | VAPA | 2 | 1.010270653 | 1.010270653 | 0.014741845 | RecName: Full=Vesicle-associated membrane protein-associated protein A; Short=VAMP-A; Short=VAMP-associated protein A; Short=VAP-A; AltName: Full=33 kDa VAMP-associated protein; Short=VAP-33 |
| gi223634732 | SUCLG1 | 5 | 1.010016556 | 1.010016556 | 0.014378941 | RecName: Full=Succinyl-CoA ligase [ADP/GDP-forming] subunit alpha, mitochondrial; AltName: Full=Succinyl-CoA synthetase subunit alpha; Short=SCS-alpha; Flags: Precursor |
| gi60390186 | MAPRE3 | 3 | 1.009406346 | 1.009406346 | 0.013507062 | RecName: Full=Microtubule-associated protein RP/EB family member 3; AltName: Full=EB1 protein family member 3; Short=EBF3; AltName: Full=End-binding protein 3; Short=EB3; AltName: Full=RP3 |
| gi341941780 | UQCRC1 | 14 | 1.006958954 | 1.006958954 | 0.010004877 | RecName: Full=Cytochrome b-c1 complex subunit 1, mitochondrial; AltName: Full=Complex III subunit 1; AltName: Full=Core protein I; AltName: Full=Ubiquinol-cytochrome-c reductase complex core protein 1; Flags: Precursor |
| gi20138723 | SLC25A11 | 2 | 1.006513495 | 1.006513495 | 0.009366515 | RecName: Full=Mitochondrial 2-oxoglutarate/malate carrier protein; Short=OGCP; AltName: Full=Solute carrier family 25 member 11 |
| gi81875980 | ASRGL1 | 2 | 1.006037467 | 1.006037467 | 0.008684035 | RecName: Full=L-asparaginase; AltName: Full=Asparaginase-like protein 1; AltName: Full=L-asparagine amidohydrolase |
| gi52783095 | PPA1 | 3 | 1.005081357 | 1.005081357 | 0.007312286 | RecName: Full=Inorganic pyrophosphatase; AltName: Full=Pyrophosphate phospho-hydrolase; Short=PPase |
| gi3122030 | CRMP1 | 12 | 1.004515014 | 1.004515014 | 0.006499128 | RecName: Full=Dihydropyrimidinase-related protein 1; Short=DRP-1; AltName: Full=Collapsin response mediator protein 1; Short=CRMP-1; AltName: Full=Unc-33-like phosphoprotein 3; Short=ULIP-3 |
| gi122065897 | PLEC | 10 | 1.004398052 | 1.004398052 | 0.006331136 | RecName: Full=Plectin; Short=PCN; Short=PLTN; AltName: Full=Plectin-1; AltName: Full=Plectin-6 |
| gi146291087 | TMOD2 | 6 | 1.003447409 | 1.003447409 | 0.004965007 | RecName: Full=Tropomodulin-2; AltName: Full=Neuronal tropomodulin; Short=N-Tmod |
| gi57012721 | CS | 12 | 1.003327873 | 1.003327873 | 0.004793134 | RecName: Full=Citrate synthase, mitochondrial; Flags: Precursor |
| gi302595876 | UBB | 7 | 1.003287386 | 1.003287386 | 0.004734917 | RecName: Full=Polyubiquitin-B; Contains: RecName: Full=Ubiquitin; Flags: Precursor |
| gi38605093 | HOMER1 | 4 | 1.000595509 | 1.000595509 | 0.000858883 | RecName: Full=Homer protein homolog 1; Short=Homer-1; AltName: Full=VASP/Ena-related gene up-regulated during seizure and LTP 1; Short=Vesl-1 |
| gi47117859 | AQP4 | 2 | 1.000039663 | 1.000039663 | 5.7221E-05 | RecName: Full=Aquaporin-4; Short=AQP-4; AltName: Full=Mercurial-insensitive water channel; Short=MIWC; AltName: Full=WCH4 |
| gi52000832 | ACAT1 | 4 | 0.999400288 | 0.999400288 | -0.000865461 | RecName: Full=Acetyl-CoA acetyltransferase, mitochondrial; AltName: Full=Acetoacetyl-CoA thiolase; Flags: Precursor |
| gi124028616 | LAP3 | 2 | 0.99913902 | 0.99913902 | -0.001242666 | RecName: Full=Cytosol aminopeptidase; AltName: Full=Leucine aminopeptidase 3; Short=LAP-3; AltName: Full=Leucyl aminopeptidase; AltName: Full=Proline aminopeptidase; AltName: Full=Prolyl aminopeptidase |
| gi21759002 | NDUFAB1 | 2 | 0.999071908 | 0.999071908 | -0.001339575 | RecName: Full=Acyl carrier protein, mitochondrial; Short=ACP; AltName: Full=CI-SDAP; AltName: Full=NADH-ubiquinone oxidoreductase 9.6 kDa subunit; Flags: Precursor |
| gi341940933 | MAP1B | 9 | 0.998859548 | 0.998859548 | -0.001646264 | RecName: Full=Microtubule-associated protein 1B; Short=MAP-1B; AltName: Full=MAP1(X); AltName: Full=MAP1.2; Contains: RecName: Full=MAP1 light chain LC1 |
| gi29840839 | PEBP1 | 6 | 0.998443341 | 0.998443341 | -0.002247534 | RecName: Full=Phosphatidylethanolamine-binding protein 1; Short=PEBP-1; AltName: Full=HCNPpp; Contains: RecName: Full=Hippocampal cholinergic neurostimulating peptide; Short=HCNP |
| gi147744591 | SEPT3 | 3 | 0.996777349 | 0.996777349 | -0.00465681 | RecName: Full=Neuronal-specific septin-3 |
| gi122028 | HIST1H2BN | 8 | 0.996721717 | 0.996721717 | -0.004737333 | RecName: Full=Histone H2B type 1-M; AltName: Full=H2B 291B |
| gi2506545 | HSPA5 | 11 | 0.996616776 | 0.996616776 | -0.004889236 | RecName: Full=78 kDa glucose-regulated protein; Short=GRP-78; AltName: Full=Heat shock 70 kDa protein 5; AltName: Full=Immunoglobulin heavy chain-binding protein; Short=BiP; Flags: Precursor |
| gi158705915 | GRM2 | 3 | 0.994163819 | -1.005870442 | -0.008444495 | RecName: Full=Metabotropic glutamate receptor 2; Short=mGluR2; Flags: Precursor |
| gi112984 | GOT2 | 14 | 0.994091009 | -1.005944114 | -0.008550158 | RecName: Full=Aspartate aminotransferase, mitochondrial; Short=mAspAT; AltName: Full=Fatty acid-binding protein; Short=FABP-1; AltName: Full=Glutamate oxaloacetate transaminase 2; AltName: Full=Plasma membrane-associated fatty acid-binding protein; Short=FABPpm; AltName: Full=Transaminase A; Flags: Precursor |
| gi92087001 | MDH1 | 13 | 0.993794226 | -1.006244526 | -0.008980935 | RecName: Full=Malate dehydrogenase, cytoplasmic; AltName: Full=Cytosolic malate dehydrogenase |
| gi9789726 | SEPT7 | 16 | 0.993367215 | -1.006677073 | -0.009600963 | RecName: Full=Septin-7; AltName: Full=CDC10 protein homolog |
| gi29427844 | NLRP3 | 2 | 0.992840275 | -1.007211356 | -0.010366455 | RecName: Full=NACHT, LRR and PYD domains-containing protein 3; AltName: Full=Cold autoinflammatory syndrome 1 protein homolog; AltName: Full=Cryopyrin; AltName: Full=Mast cell maturation-associated-inducible protein 1; AltName: Full=PYRIN-containing APAF1-like protein 1 |
| gi120223 | FKBP1A | 2 | 0.992001993 | -1.00806249 | -0.011585075 | RecName: Full=Peptidyl-prolyl cis-trans isomerase FKBP1A; Short=PPIase FKBP1A; AltName: Full=12 kDa FK506-binding protein; Short=12 kDa FKBP; Short=FKBP-12; AltName: Full=FK506-binding protein 1A; Short=FKBP-1A; AltName: Full=Immunophilin FKBP12; AltName: Full=Rotamase |
| gi146345457 | MDH2 (includes EG:17448) | 15 | 0.99150728 | -1.008565464 | -0.012304729 | RecName: Full=Malate dehydrogenase, mitochondrial; Flags: Precursor |
| gi51316996 | CPLX2 | 6 | 0.990989332 | -1.009092599 | -0.013058568 | RecName: Full=Complexin-2; AltName: Full=921-L; AltName: Full=Complexin II; Short=CPX II; AltName: Full=Synaphin-1 |
| gi1350769 | RPL7 | 2 | 0.99013 | -1.009968388 | -0.014310137 | RecName: Full=60S ribosomal protein L7 |
| gi55976751 | ATP1A1 | 31 | 0.989369878 | -1.010744335 | -0.015418118 | RecName: Full=Sodium/potassium-transporting ATPase subunit alpha-1; Short=Na(+)/K(+) ATPase alpha-1 subunit; AltName: Full=Sodium pump subunit alpha-1; Flags: Precursor |
| gi71153505 | DHX9 | 2 | 0.988286882 | -1.011851942 | -0.016998204 | RecName: Full=ATP-dependent RNA helicase A; AltName: Full=DEAH box protein 9; Short=mHEL-5; AltName: Full=Nuclear DNA helicase II; Short=NDH II |
| gi81879424 | HNRNPU | 5 | 0.988180463 | -1.01196091 | -0.017153562 | RecName: Full=Heterogeneous nuclear ribonucleoprotein U; Short=hnRNP U; AltName: Full=Scaffold attachment factor A; Short=SAF-A |
| gi108935937 | TCEAL3 | 3 | 0.987504393 | -1.012653722 | -0.018140928 | RecName: Full=Transcription elongation factor A protein-like 3; Short=TCEA-like protein 3; AltName: Full=Transcription elongation factor S-II protein-like 3 |
| gi51317340 | H4 | 5 | 0.986885704 | -1.013288566 | -0.019045085 | RecName: Full=Histone H4 |
| gi334305788 | GNAI1 | 4 | 0.985715595 | -1.014491406 | -0.020756644 | RecName: Full=Guanine nucleotide-binding protein G(i) subunit alpha-1; AltName: Full=Adenylate cyclase-inhibiting G alpha protein |
| gi17380314 | NAPG | 6 | 0.985388749 | -1.014827905 | -0.021235096 | RecName: Full=Gamma-soluble NSF attachment protein; Short=SNAP-gamma; AltName: Full=N-ethylmaleimide-sensitive factor attachment protein gamma |
| gi37999865 | MLL3 | 2 | 0.984698354 | -1.015539425 | -0.022246249 | RecName: Full=Histone-lysine N-methyltransferase MLL3; AltName: Full=Myeloid/lymphoid or mixed-lineage leukemia protein 3 homolog |
| gi81871239 | HIST1H2AC | 5 | 0.983747967 | -1.016520525 | -0.023639346 | RecName: Full=Histone H2A type 1-H |
| gi46396175 | NRGN | 4 | 0.983325751 | -1.016956994 | -0.024258671 | RecName: Full=Neurogranin; Short=Ng; AltName: Full=RC3; Contains: RecName: Full=NEUG(55-78) |
| gi172044688 | EHD3 | 5 | 0.982885587 | -1.017412417 | -0.024904606 | RecName: Full=EH domain-containing protein 3 |
| gi46397464 | EIF4A1 | 3 | 0.982500009 | -1.017811695 | -0.025470674 | RecName: Full=Eukaryotic initiation factor 4A-I; Short=eIF-4A-I; Short=eIF4A-I; AltName: Full=ATP-dependent RNA helicase eIF4A-1 |
| gi112804 | SLC3A2 | 5 | 0.982201204 | -1.018121334 | -0.025909503 | RecName: Full=4F2 cell-surface antigen heavy chain; Short=4F2hc; AltName: CD_antigen=CD98 |
| gi48428679 | OXCT1 | 3 | 0.981683802 | -1.018657941 | -0.026669685 | RecName: Full=Succinyl-CoA:3-ketoacid-coenzyme A transferase 1, mitochondrial; AltName: Full=3-oxoacid-CoA transferase 1; AltName: Full=Somatic-type succinyl-CoA:3-oxoacid CoA-transferase; Short=SCOT-s; Flags: Precursor |
| gi1170384 | HSP90AA1 | 25 | 0.981179043 | -1.01918198 | -0.027411675 | RecName: Full=Heat shock protein HSP 90-alpha; AltName: Full=Heat shock 86 kDa; Short=HSP 86; Short=HSP86; AltName: Full=Tumor-specific transplantation 86 kDa antigen; Short=TSTA |
| gi114041 | APOE | 5 | 0.981102037 | -1.019261975 | -0.027524906 | RecName: Full=Apolipoprotein E; Short=Apo-E; Flags: Precursor |
| gi81916660 | CADM3 | 2 | 0.97754634 | -1.022969407 | -0.032763001 | RecName: Full=Cell adhesion molecule 3; AltName: Full=Immunoglobulin superfamily member 4B; Short=IgSF4B; AltName: Full=Nectin-like protein 1; Short=NECL-1; AltName: Full=Synaptic cell adhesion molecule 3; AltName: Full=TSLC1-like protein 1; Flags: Precursor |
| gi52000687 | ATP1A3 (includes EG:232975) | 45 | 0.977336661 | -1.023188876 | -0.033072485 | RecName: Full=Sodium/potassium-transporting ATPase subunit alpha-3; Short=Na(+)/K(+) ATPase alpha-3 subunit; AltName: Full=Na(+)/K(+) ATPase alpha(III) subunit; AltName: Full=Sodium pump subunit alpha-3 |
| gi81894883 | AHCYL1 | 4 | 0.977250386 | -1.023279207 | -0.033199846 | RecName: Full=Putative adenosylhomocysteinase 2; Short=AdoHcyase 2; AltName: Full=IP3R-binding protein released with inositol 1,4,5-trisphosphate; AltName: Full=S-adenosyl-L-homocysteine hydrolase 2; AltName: Full=S-adenosylhomocysteine hydrolase-like protein 1 |
| gi83305135 | NDUFS6 | 3 | 0.977202483 | -1.023329369 | -0.033270566 | RecName: Full=NADH dehydrogenase [ubiquinone] iron-sulfur protein 6, mitochondrial; AltName: Full=Complex I-13kD-A; Short=CI-13kD-A; AltName: Full=NADH-ubiquinone oxidoreductase 13 kDa-A subunit; Flags: Precursor |
| gi1345696 | CAPZA2 | 4 | 0.977053155 | -1.023485769 | -0.033491043 | RecName: Full=F-actin-capping protein subunit alpha-2; AltName: Full=CapZ alpha-2 |
| gi341940828 | KPNB1 | 5 | 0.976565321 | -1.023997042 | -0.034211547 | RecName: Full=Importin subunit beta-1; AltName: Full=Karyopherin subunit beta-1; AltName: Full=Nuclear factor p97; AltName: Full=Pore targeting complex 97 kDa subunit; Short=PTAC97; AltName: Full=SCG |
| gi41688568 | SLC9A3R1 | 2 | 0.975790465 | -1.024810177 | -0.035356708 | RecName: Full=Na(+)/H(+) exchange regulatory cofactor NHE-RF1; Short=NHERF-1; AltName: Full=Ezrin-radixin-moesin-binding phosphoprotein 50; Short=EBP50; AltName: Full=Regulatory cofactor of Na(+)/H(+) exchanger; AltName: Full=Sodium-hydrogen exchanger regulatory factor 1; AltName: Full=Solute carrier family 9 isoform A3 regulatory factor 1 |
| gi47117291 | NDUFS5 | 2 | 0.975254406 | -1.025373476 | -0.036149484 | RecName: Full=NADH dehydrogenase [ubiquinone] iron-sulfur protein 5; AltName: Full=Complex I-15 kDa; Short=CI-15 kDa; AltName: Full=NADH-ubiquinone oxidoreductase 15 kDa subunit |
| gi19862081 | UQCRHL | 3 | 0.974016758 | -1.026676381 | -0.037981501 | RecName: Full=Cytochrome b-c1 complex subunit 6, mitochondrial; AltName: Full=Complex III subunit 6; AltName: Full=Complex III subunit VIII; AltName: Full=Cytochrome c1 non-heme 11 kDa protein; AltName: Full=Mitochondrial hinge protein; AltName: Full=Ubiquinol-cytochrome c reductase complex 11 kDa protein; Flags: Precursor |
| gi2492687 | SNAP91 | 7 | 0.973910343 | -1.026788562 | -0.038139129 | RecName: Full=Clathrin coat assembly protein AP180; AltName: Full=91 kDa synaptosomal-associated protein; AltName: Full=Clathrin coat-associated protein AP180; AltName: Full=Phosphoprotein F1-20 |
| gi20454828 | ATP5F1 | 6 | 0.973900198 | -1.026799257 | -0.038154157 | RecName: Full=ATP synthase subunit b, mitochondrial; Short=ATPase subunit b; Flags: Precursor |
| gi146345448 | PKM2 | 27 | 0.972402301 | -1.028380948 | -0.040374787 | RecName: Full=Pyruvate kinase isozymes M1/M2; AltName: Full=Pyruvate kinase muscle isozyme |
| gi2493731 | CLTA | 6 | 0.970905422 | -1.029966439 | -0.042597329 | RecName: Full=Clathrin light chain A; Short=Lca |
| gi81908472 | F5 | 3 | 0.969905897 | -1.031027859 | -0.044083316 | RecName: Full=Coagulation factor V; AltName: Full=Activated protein C cofactor; Contains: RecName: Full=Coagulation factor V heavy chain; Contains: RecName: Full=Coagulation factor V light chain; Flags: Precursor |
| gi1711560 | STMN1 | 4 | 0.969764586 | -1.031178096 | -0.044293524 | RecName: Full=Stathmin; AltName: Full=Leukemia-associated gene protein; AltName: Full=Leukemia-associated phosphoprotein p18; AltName: Full=Metablastin; AltName: Full=Oncoprotein 18; Short=Op18; AltName: Full=Phosphoprotein p19; Short=pp19; AltName: Full=Prosolin; AltName: Full=Protein Pr22; AltName: Full=pp17 |
| gi117029 | COX2 (includes EG:140540) | 3 | 0.968767975 | -1.032238912 | -0.045776921 | RecName: Full=Cytochrome c oxidase subunit 2; AltName: Full=Cytochrome c oxidase polypeptide II |
| gi353526354 | TPI1 | 10 | 0.968377021 | -1.032655648 | -0.04635925 | RecName: Full=Triosephosphate isomerase; Short=TIM; AltName: Full=Triose-phosphate isomerase |
| gi48429206 | STXBP1 | 23 | 0.967718118 | -1.033358765 | -0.047341222 | RecName: Full=Syntaxin-binding protein 1; AltName: Full=Protein unc-18 homolog 1; Short=Unc18-1; AltName: Full=Protein unc-18 homolog A; Short=Unc-18A |
| gi3023203 | 2010107E04Rik | 2 | 0.967259121 | -1.03384913 | -0.048025667 | RecName: Full=6.8 kDa mitochondrial proteolipid |
| gi341940718 | KHSRP | 2 | 0.96673349 | -1.034411252 | -0.048809874 | RecName: Full=Far upstream element-binding protein 2; Short=FUSE-binding protein 2; AltName: Full=KH type-splicing regulatory protein; Short=KSRP |
| gi17380333 | UQCRB | 8 | 0.966220432 | -1.03496052 | -0.049575735 | RecName: Full=Cytochrome b-c1 complex subunit 7; AltName: Full=Complex III subunit 7; AltName: Full=Complex III subunit VII; AltName: Full=Ubiquinol-cytochrome c reductase complex 14 kDa protein |
| gi399310 | CTNNB1 | 6 | 0.965956349 | -1.035243467 | -0.049970099 | RecName: Full=Catenin beta-1; AltName: Full=Beta-catenin |
| gi13432200 | MAPT | 6 | 0.965534196 | -1.035696099 | -0.050600739 | RecName: Full=Microtubule-associated protein tau; AltName: Full=Neurofibrillary tangle protein; AltName: Full=Paired helical filament-tau; Short=PHF-tau |
| gi76364091 | BAG6 | 2 | 0.96530077 | -1.035946547 | -0.050949565 | RecName: Full=Large proline-rich protein BAG6; AltName: Full=BAG family molecular chaperone regulator 6; AltName: Full=BCL2-associated athanogene 6; Short=BAG-6; Short=BAG6; AltName: Full=HLA-B-associated transcript 3; AltName: Full=Protein Scythe |
| gi20455479 | ATP5B | 23 | 0.964689595 | -1.036602867 | -0.051863289 | RecName: Full=ATP synthase subunit beta, mitochondrial; Flags: Precursor |
| gi3219774 | PRDX6 | 9 | 0.96441891 | -1.036893812 | -0.052268155 | RecName: Full=Peroxiredoxin-6; AltName: Full=1-Cys peroxiredoxin; Short=1-Cys PRX; AltName: Full=Acidic calcium-independent phospholipase A2; Short=aiPLA2; AltName: Full=Antioxidant protein 2; AltName: Full=Non-selenium glutathione peroxidase; Short=NSGPx |
| gi18202836 | YWHAB | 13 | 0.963188382 | -1.038218503 | -0.054110104 | RecName: Full=14-3-3 protein beta/alpha; AltName: Full=Protein kinase C inhibitor protein 1; Short=KCIP-1; Contains: RecName: Full=14-3-3 protein beta/alpha, N-terminally processed |
| gi62901088 | RAB35 | 2 | 0.962457391 | -1.039007035 | -0.055205423 | RecName: Full=Ras-related protein Rab-35 |
| gi205830666 | NCAM1 | 8 | 0.96089758 | -1.040693639 | -0.057545429 | RecName: Full=Neural cell adhesion molecule 1; Short=N-CAM-1; Short=NCAM-1; AltName: CD_antigen=CD56; Flags: Precursor |
| gi341940560 | CADPS | 5 | 0.960094318 | -1.041564335 | -0.058751954 | RecName: Full=Calcium-dependent secretion activator 1; AltName: Full=Calcium-dependent activator protein for secretion 1; Short=CAPS-1 |
| gi9910790 | TAGLN3 | 2 | 0.959981771 | -1.041686447 | -0.058921084 | RecName: Full=Transgelin-3; AltName: Full=Neuronal protein NP25 |
| gi81862370 | CYFIP2 | 6 | 0.959081007 | -1.042664794 | -0.06027542 | RecName: Full=Cytoplasmic FMR1-interacting protein 2; AltName: Full=p53-inducible protein 121 |
| gi30581069 | SFXN5 | 3 | 0.9588445 | -1.042921976 | -0.060631229 | RecName: Full=Sideroflexin-5 |
| gi38257686 | GDAP1L1 | 2 | 0.95730135 | -1.044603143 | -0.06295495 | RecName: Full=Ganglioside-induced differentiation-associated protein 1-like 1; Short=GDAP1-L1 |
| gi46397704 | RPS20 | 2 | 0.956950373 | -1.04498627 | -0.063483986 | RecName: Full=40S ribosomal protein S20 |
| gi78099818 | ATAD3A/ATAD3B | 3 | 0.95528055 | -1.046812897 | -0.066003605 | RecName: Full=ATPase family AAA domain-containing protein 3; AltName: Full=AAA-ATPase TOB3 |
| gi13637776 | ENO1 | 23 | 0.95522679 | -1.046871812 | -0.066084797 | RecName: Full=Alpha-enolase; AltName: Full=2-phospho-D-glycerate hydro-lyase; AltName: Full=Enolase 1; AltName: Full=Non-neural enolase; Short=NNE |
| gi8928228 | NDRG2 | 5 | 0.955088981 | -1.047022864 | -0.066292947 | RecName: Full=Protein NDRG2; AltName: Full=Protein Ndr2 |
| gi223634791 | ANK2 | 7 | 0.954400376 | -1.047778297 | -0.067333484 | RecName: Full=Ankyrin-2; Short=ANK-2; AltName: Full=Brain ankyrin |
| gi120975 | GNAO1 | 13 | 0.953404475 | -1.048872778 | -0.068839698 | RecName: Full=Guanine nucleotide-binding protein G(o) subunit alpha |
| gi38372295 | NFASC | 7 | 0.952812394 | -1.04952455 | -0.069735915 | RecName: Full=Neurofascin; Flags: Precursor |
| gi47117296 | NDUFC2 | 4 | 0.952786248 | -1.049553351 | -0.069775504 | RecName: Full=NADH dehydrogenase [ubiquinone] 1 subunit C2; AltName: Full=Complex I-B14.5b; Short=CI-B14.5b; AltName: Full=NADH-ubiquinone oxidoreductase subunit B14.5b |
| gi32363386 | NDUFA10 | 5 | 0.952255767 | -1.050138035 | -0.070578975 | RecName: Full=NADH dehydrogenase [ubiquinone] 1 alpha subcomplex subunit 10, mitochondrial; AltName: Full=Complex I-42kD; Short=CI-42kD; AltName: Full=NADH-ubiquinone oxidoreductase 42 kDa subunit; Flags: Precursor |
| gi254763295 | FLOT2 | 2 | 0.950207813 | -1.052401366 | -0.073685026 | RecName: Full=Flotillin-2; AltName: Full=Epidermal surface antigen; Short=ESA; AltName: Full=Membrane component chromosome 17 surface marker 1 homolog |
| gi52783073 | LSAMP | 4 | 0.94957939 | -1.053097836 | -0.074639473 | RecName: Full=Limbic system-associated membrane protein; Short=LSAMP; Flags: Precursor |
| gi110287952 | SLC4A10 | 2 | 0.948964393 | -1.053780318 | -0.07557414 | RecName: Full=Sodium-driven chloride bicarbonate exchanger; AltName: Full=Solute carrier family 4 member 10 |
| gi50402098 | EEF1A2 | 10 | 0.948739714 | -1.054029873 | -0.075915756 | RecName: Full=Elongation factor 1-alpha 2; Short=EF-1-alpha-2; AltName: Full=Eukaryotic elongation factor 1 A-2; Short=eEF1A-2; AltName: Full=Statin-S1 |
| gi114393 | ATP1B1 | 10 | 0.948175188 | -1.054657423 | -0.076774455 | RecName: Full=Sodium/potassium-transporting ATPase subunit beta-1; AltName: Full=Sodium/potassium-dependent ATPase subunit beta-1 |
| gi20177955 | CAMK2G | 8 | 0.947909367 | -1.054953179 | -0.077178971 | RecName: Full=Calcium/calmodulin-dependent protein kinase type II subunit gamma; Short=CaM kinase II subunit gamma; Short=CaMK-II subunit gamma |
| gi54036445 | STIP1 | 5 | 0.947822001 | -1.05505042 | -0.077311946 | RecName: Full=Stress-induced-phosphoprotein 1; Short=STI1; Short=mSTI1; AltName: Full=Hsc70/Hsp90-organizing protein; Short=Hop |
| gi46395611 | ANP32A | 3 | 0.947554581 | -1.055348177 | -0.077719047 | RecName: Full=Acidic leucine-rich nuclear phosphoprotein 32 family member A; AltName: Full=Acidic nuclear phosphoprotein pp32; AltName: Full=Leucine-rich acidic nuclear protein; Short=LANP; AltName: Full=Potent heat-stable protein phosphatase 2A inhibitor I1PP2A |
| gi14916635 | PYGM | 5 | 0.947189312 | -1.055755157 | -0.078275294 | RecName: Full=Glycogen phosphorylase, muscle form; AltName: Full=Myophosphorylase |
| gi3914438 | PSMA3 | 2 | 0.947112021 | -1.055841313 | -0.078393023 | RecName: Full=Proteasome subunit alpha type-3; AltName: Full=Macropain subunit C8; AltName: Full=Multicatalytic endopeptidase complex subunit C8; AltName: Full=Proteasome component C8; AltName: Full=Proteasome subunit K |
| gi13626886 | GDI2 | 8 | 0.94620166 | -1.056857161 | -0.079780404 | RecName: Full=Rab GDP dissociation inhibitor beta; Short=Rab GDI beta; AltName: Full=GDI-3; AltName: Full=Guanosine diphosphate dissociation inhibitor 2; Short=GDI-2 |
| gi120702 | GAPDH | 12 | 0.945653767 | -1.057469483 | -0.08061603 | RecName: Full=Glyceraldehyde-3-phosphate dehydrogenase; Short=GAPDH; AltName: Full=Peptidyl-cysteine S-nitrosylase GAPDH |
| gi59798463 | LIN7A | 3 | 0.944845322 | -1.058374293 | -0.081849926 | RecName: Full=Protein lin-7 homolog A; Short=Lin-7A; Short=mLin-7; AltName: Full=Mammalian lin-seven protein 1; Short=MALS-1; AltName: Full=Vertebrate lin-7 homolog 1; Short=Veli-1 |
| gi1709737 | PITPNA | 3 | 0.944813113 | -1.058410374 | -0.081899107 | RecName: Full=Phosphatidylinositol transfer protein alpha isoform; Short=PI-TP-alpha; Short=PtdIns transfer protein alpha; Short=PtdInsTP alpha |
| gi152031595 | DMXL2 | 8 | 0.944577469 | -1.058674415 | -0.082258971 | RecName: Full=DmX-like protein 2; AltName: Full=Rabconnectin-3 |
| gi2851596 | TALDO1 | 4 | 0.943710414 | -1.059647096 | -0.083583871 | RecName: Full=Transaldolase |
| gi205830867 | NFU1 | 2 | 0.943573323 | -1.059801052 | -0.083793465 | RecName: Full=NFU1 iron-sulfur cluster scaffold homolog, mitochondrial; AltName: Full=HIRA-interacting protein 5; Short=mHIRIP5; Flags: Precursor |
| gi341941160 | NDUFS1 | 9 | 0.942465279 | -1.061047046 | -0.085488625 | RecName: Full=NADH-ubiquinone oxidoreductase 75 kDa subunit, mitochondrial; AltName: Full=Complex I-75kD; Short=CI-75kD; Flags: Precursor |
| gi71152120 | HGS | 2 | 0.941505481 | -1.062128708 | -0.086958602 | RecName: Full=Hepatocyte growth factor-regulated tyrosine kinase substrate |
| gi18202309 | OPA1 | 8 | 0.939368487 | -1.064544973 | -0.090236899 | RecName: Full=Dynamin-like 120 kDa protein, mitochondrial; AltName: Full=Large GTP-binding protein; Short=LargeG; AltName: Full=Optic atrophy protein 1 homolog; Contains: RecName: Full=Dynamin-like 120 kDa protein, form S1; Flags: Precursor |
| gi20178348 | SEPT6 | 7 | 0.938941482 | -1.065029099 | -0.090892848 | RecName: Full=Septin-6 |
| gi47117288 | RAP1B | 3 | 0.938462902 | -1.065572222 | -0.091628379 | RecName: Full=Ras-related protein Rap-1b; AltName: Full=GTP-binding protein smg p21B; Flags: Precursor |
| gi126042 | LDHB | 14 | 0.938153017 | -1.065924196 | -0.092104843 | RecName: Full=L-lactate dehydrogenase B chain; Short=LDH-B; AltName: Full=LDH heart subunit; Short=LDH-H |
| gi49065818 | PSMC1 | 2 | 0.93799755 | -1.066100865 | -0.09234394 | RecName: Full=26S protease regulatory subunit 4; Short=P26s4; AltName: Full=26S proteasome AAA-ATPase subunit RPT2; AltName: Full=Proteasome 26S subunit ATPase 1 |
| gi66773992 | ATP1A2 | 34 | 0.937301237 | -1.066892862 | -0.093415308 | RecName: Full=Sodium/potassium-transporting ATPase subunit alpha-2; Short=Na(+)/K(+) ATPase alpha-2 subunit; AltName: Full=Na(+)/K(+) ATPase alpha(+) subunit; AltName: Full=Sodium pump subunit alpha-2; Flags: Precursor |
| gi123778087 | HNRNPUL2 | 2 | 0.936066201 | -1.06830051 | -0.095317531 | RecName: Full=Heterogeneous nuclear ribonucleoprotein U-like protein 2; AltName: Full=MLF1-associated nuclear protein |
| gi14917005 | HSPA9 | 9 | 0.936033201 | -1.068338173 | -0.095368391 | RecName: Full=Stress-70 protein, mitochondrial; AltName: Full=75 kDa glucose-regulated protein; Short=GRP-75; AltName: Full=Heat shock 70 kDa protein 9; AltName: Full=Mortalin; AltName: Full=Peptide-binding protein 74; Short=PBP74; AltName: Full=p66 MOT; Flags: Precursor |
| gi146291078 | VCP | 9 | 0.935135025 | -1.069364288 | -0.096753403 | RecName: Full=Transitional endoplasmic reticulum ATPase; Short=TER ATPase; AltName: Full=15S Mg(2+)-ATPase p97 subunit; AltName: Full=Valosin-containing protein; Short=VCP |
| gi47606041 | PHYHIP | 4 | 0.934477945 | -1.070116213 | -0.09776748 | RecName: Full=Phytanoyl-CoA hydroxylase-interacting protein; AltName: Full=Phytanoyl-CoA hydroxylase-associated protein 1; Short=PAHX-AP1; Short=PAHXAP1 |
| gi44888264 | OTUB1 | 3 | 0.934144121 | -1.070498629 | -0.098282947 | RecName: Full=Ubiquitin thioesterase OTUB1; AltName: Full=Deubiquitinating enzyme OTUB1; AltName: Full=OTU domain-containing ubiquitin aldehyde-binding protein 1; AltName: Full=Otubain-1; AltName: Full=Ubiquitin-specific-processing protease OTUB1 |
| gi81885886 | NAP1L4 | 2 | 0.932554233 | -1.072323694 | -0.100740467 | RecName: Full=Nucleosome assembly protein 1-like 4 |
| gi266414 | CKMT1A/CKMT1B | 14 | 0.932099798 | -1.072846493 | -0.101443665 | RecName: Full=Creatine kinase U-type, mitochondrial; AltName: Full=Acidic-type mitochondrial creatine kinase; Short=Mia-CK; AltName: Full=Ubiquitous mitochondrial creatine kinase; Short=U-MtCK; Flags: Precursor |
| gi49037483 | CALM | 7 | 0.932096914 | -1.072849814 | -0.10144813 | RecName: Full=Calmodulin; Short=CaM |
| gi46576640 | GMFB | 2 | 0.931618796 | -1.073400413 | -0.102188348 | RecName: Full=Glia maturation factor beta; Short=GMF-beta |
| gi2507330 | EIF4A2 | 5 | 0.930999231 | -1.074114743 | -0.103148118 | RecName: Full=Eukaryotic initiation factor 4A-II; Short=eIF-4A-II; Short=eIF4A-II; AltName: Full=ATP-dependent RNA helicase eIF4A-2 |
| gi5915682 | ALB | 15 | 0.930365175 | -1.074846766 | -0.104130999 | RecName: Full=Serum albumin; Flags: Precursor |
| gi34098594 | EPPK1 | 2 | 0.930176468 | -1.075064823 | -0.104423653 | RecName: Full=Epiplakin |
| gi146345423 | GFAP | 8 | 0.927838401 | -1.077773887 | -0.108054538 | RecName: Full=Glial fibrillary acidic protein; Short=GFAP |
| gi18203410 | UCHL1 | 8 | 0.9275264 | -1.078136429 | -0.10853975 | RecName: Full=Ubiquitin carboxyl-terminal hydrolase isozyme L1; Short=UCH-L1; AltName: Full=Neuron cytoplasmic protein 9.5; AltName: Full=PGP 9.5; Short=PGP9.5; AltName: Full=Ubiquitin thioesterase L1 |
| gi81912821 | GSTM2 | 4 | 0.92723981 | -1.078469657 | -0.108985586 | RecName: Full=Glutathione S-transferase Mu 7; AltName: Full=GST class-mu 7; Short=GSTM-7 |
| gi341940634 | BSN | 13 | 0.927014268 | -1.078732048 | -0.10933655 | RecName: Full=Protein bassoon |
| gi135831 | THY1 | 3 | 0.926777698 | -1.079007406 | -0.109704767 | RecName: Full=Thy-1 membrane glycoprotein; AltName: Full=Thy-1 antigen; AltName: CD_antigen=CD90; Flags: Precursor |
| gi68566157 | YWHAQ | 11 | 0.926282008 | -1.079584825 | -0.110476604 | RecName: Full=14-3-3 protein theta; AltName: Full=14-3-3 protein tau |
| gi13626388 | EEF1G | 2 | 0.926240367 | -1.079633361 | -0.110541462 | RecName: Full=Elongation factor 1-gamma; Short=EF-1-gamma; AltName: Full=eEF-1B gamma |
| gi46397725 | SNAP25 | 9 | 0.925969234 | -1.079949488 | -0.110963836 | RecName: Full=Synaptosomal-associated protein 25; Short=SNAP-25; AltName: Full=Super protein; Short=SUP; AltName: Full=Synaptosomal-associated 25 kDa protein |
| gi341941065 | HSP90AB1 | 23 | 0.925798999 | -1.080148067 | -0.111229091 | RecName: Full=Heat shock protein HSP 90-beta; AltName: Full=Heat shock 84 kDa; Short=HSP 84; Short=HSP84; AltName: Full=Tumor-specific transplantation 84 kDa antigen; Short=TSTA |
| gi121557 | GPD1 | 2 | 0.925007618 | -1.081072178 | -0.112462848 | RecName: Full=Glycerol-3-phosphate dehydrogenase [NAD+], cytoplasmic; Short=GPD-C; Short=GPDH-C |
| gi24418394 | ALDH1L1 | 2 | 0.924825942 | -1.081284547 | -0.112746227 | RecName: Full=Cytosolic 10-formyltetrahydrofolate dehydrogenase; Short=10-FTHFDH; Short=FDH; AltName: Full=Aldehyde dehydrogenase family 1 member L1 |
| gi47605479 | SLC25A12 | 11 | 0.923988117 | -1.082265001 | -0.114053797 | RecName: Full=Calcium-binding mitochondrial carrier protein Aralar1; AltName: Full=Mitochondrial aspartate glutamate carrier 1; AltName: Full=Solute carrier family 25 member 12 |
| gi47117855 | GDI1 | 15 | 0.921966343 | -1.084638293 | -0.11721401 | RecName: Full=Rab GDP dissociation inhibitor alpha; Short=Rab GDI alpha; AltName: Full=Guanosine diphosphate dissociation inhibitor 1; Short=GDI-1 |
| gi44888257 | PICALM | 2 | 0.919497517 | -1.087550517 | -0.121082416 | RecName: Full=Phosphatidylinositol-binding clathrin assembly protein; AltName: Full=Clathrin assembly lymphoid myeloid leukemia; Short=CALM |
| gi55584163 | PPP3CB | 7 | 0.919463464 | -1.087590795 | -0.121135846 | RecName: Full=Serine/threonine-protein phosphatase 2B catalytic subunit beta isoform; AltName: Full=CAM-PRP catalytic subunit; AltName: Full=Calmodulin-dependent calcineurin A subunit beta isoform |
| gi47117166 | NDUFA12 | 4 | 0.917833554 | -1.089522164 | -0.123695546 | RecName: Full=NADH dehydrogenase [ubiquinone] 1 alpha subcomplex subunit 12; AltName: Full=Complex I-B17.2; Short=CI-B17.2; Short=CIB17.2; AltName: Full=NADH-ubiquinone oxidoreductase subunit B17.2 |
| gi54037163 | GANAB | 2 | 0.914939232 | -1.092968763 | -0.128252169 | RecName: Full=Neutral alpha-glucosidase AB; AltName: Full=Alpha-glucosidase 2; AltName: Full=Glucosidase II subunit alpha; Flags: Precursor |
| gi30913117 | SCRN1 | 5 | 0.91358482 | -1.094589116 | -0.130389416 | RecName: Full=Secernin-1 |
| gi341942096 | DARS | 2 | 0.913419519 | -1.094787202 | -0.130650475 | RecName: Full=Aspartyl-tRNA synthetase, cytoplasmic; AltName: Full=Aspartate--tRNA ligase; Short=AspRS |
| gi391359280 | GLSK | 4 | 0.912920811 | -1.09538526 | -0.131438372 | RecName: Full=Glutaminase kidney isoform, mitochondrial; Short=GLS; Flags: Precursor |
| gi55584180 | PHGDH | 3 | 0.912433702 | -1.095970039 | -0.13220836 | RecName: Full=D-3-phosphoglycerate dehydrogenase; Short=3-PGDH; AltName: Full=A10 |
| gi543921 | CANX | 10 | 0.910804608 | -1.097930326 | -0.134786504 | RecName: Full=Calnexin; Flags: Precursor |
| gi55977063 | H33 | 4 | 0.910080924 | -1.098803385 | -0.135933259 | RecName: Full=Histone H3.3 |
| gi20140196 | PSAT1 | 3 | 0.909238725 | -1.099821172 | -0.137268964 | RecName: Full=Phosphoserine aminotransferase; Short=PSAT; AltName: Full=Endometrial progesterone-induced protein; Short=EPIP; AltName: Full=Phosphohydroxythreonine aminotransferase |
| gi51338605 | RAB3C (includes EG:115827) | 6 | 0.907837413 | -1.101518825 | -0.139494151 | RecName: Full=Ras-related protein Rab-3C |
| gi46396900 | PYGB | 17 | 0.906284404 | -1.103406387 | -0.141964236 | RecName: Full=Glycogen phosphorylase, brain form |
| gi48429104 | HNRNPK | 8 | 0.905693742 | -1.10412599 | -0.142904805 | RecName: Full=Heterogeneous nuclear ribonucleoprotein K; Short=hnRNP K |
| gi341942263 | PRPS1 | 3 | 0.904718668 | -1.105315979 | -0.144458855 | RecName: Full=Ribose-phosphate pyrophosphokinase 1; AltName: Full=Phosphoribosyl pyrophosphate synthase I; Short=PRS-I |
| gi59797853 | DLG1 | 2 | 0.902147554 | -1.108466122 | -0.148564677 | RecName: Full=Disks large homolog 1; AltName: Full=Embryo-dlg/synapse-associated protein 97; Short=E-dlg/SAP97; AltName: Full=Synapse-associated protein 97; Short=SAP-97; Short=SAP97 |
| gi84029467 | SLC4A4 | 3 | 0.901616658 | -1.109118816 | -0.149413925 | RecName: Full=Electrogenic sodium bicarbonate cotransporter 1; Short=Sodium bicarbonate cotransporter; AltName: Full=Na(+)/HCO3(-) cotransporter; AltName: Full=Solute carrier family 4 member 4 |
| gi146345421 | FSCN1 | 6 | 0.900987302 | -1.109893556 | -0.150421322 | RecName: Full=Fascin; AltName: Full=Singed-like protein |
| gi47117767 | STX1B | 10 | 0.899791104 | -1.111369067 | -0.152337992 | RecName: Full=Syntaxin-1B |
| gi327478516 | HYDIN | 4 | 0.89732118 | -1.114428169 | -0.15630363 | RecName: Full=Hydrocephalus-inducing protein; AltName: Full=Protein Hy-3 |
| gi110825706 | ARPC2 | 3 | 0.896598053 | -1.115326982 | -0.157466729 | RecName: Full=Actin-related protein 2/3 complex subunit 2; AltName: Full=Arp2/3 complex 34 kDa subunit; Short=p34-ARC |
| gi25453098 | CISD1 | 3 | 0.896459904 | -1.115498859 | -0.157689038 | RecName: Full=CDGSH iron-sulfur domain-containing protein 1; AltName: Full=MitoNEET |
| gi51702142 | UBE2V2 | 2 | 0.896108784 | -1.115935942 | -0.158254215 | RecName: Full=Ubiquitin-conjugating enzyme E2 variant 2; AltName: Full=Ubc-like protein MMS2 |
| gi119362 | HSP90B1 | 4 | 0.894943882 | -1.117388498 | -0.160130875 | RecName: Full=Endoplasmin; AltName: Full=94 kDa glucose-regulated protein; Short=GRP-94; AltName: Full=Endoplasmic reticulum resident protein 99; Short=ERp99; AltName: Full=Heat shock protein 90 kDa beta member 1; AltName: Full=Polymorphic tumor rejection antigen 1; AltName: Full=Tumor rejection antigen gp96; Flags: Precursor |
| gi46576352 | CACNA2D1 | 2 | 0.893913263 | -1.118676769 | -0.161793243 | RecName: Full=Voltage-dependent calcium channel subunit alpha-2/delta-1; AltName: Full=Voltage-gated calcium channel subunit alpha-2/delta-1; Contains: RecName: Full=Voltage-dependent calcium channel subunit alpha-2-1; Contains: RecName: Full=Voltage-dependent calcium channel subunit delta-1; Flags: Precursor |
| gi51702234 | CYCS | 5 | 0.893593905 | -1.119076568 | -0.16230875 | RecName: Full=Cytochrome c, somatic |
| gi292630943 | SYNE2 | 2 | 0.890311452 | -1.123202446 | -0.167617982 | RecName: Full=Nesprin-2; AltName: Full=Nuclear envelope spectrin repeat protein 2; AltName: Full=Nucleus and actin connecting element protein; Short=Protein NUANCE; AltName: Full=Synaptic nuclear envelope protein 2; Short=Syne-2 |
| gi1170099 | GSTP1 (includes others)* | 3 | 0.889554499 | -1.124158217 | -0.168845099 | RecName: Full=Glutathione S-transferase P 2; Short=Gst P2; AltName: Full=GST YF-YF; AltName: Full=GST class-pi; AltName: Full=GST-piA |
| gi27734459 | RALB | 2 | 0.889434526 | -1.124309851 | -0.169039686 | RecName: Full=Ras-related protein Ral-B; Flags: Precursor |
| gi51702275 | HSPA8 | 31 | 0.887490568 | -1.126772539 | -0.172196308 | RecName: Full=Heat shock cognate 71 kDa protein; AltName: Full=Heat shock 70 kDa protein 8 |
| gi52783085 | PPA2 (includes EG:27068) | 3 | 0.887285228 | -1.127033301 | -0.172530145 | RecName: Full=Inorganic pyrophosphatase 2, mitochondrial; AltName: Full=Pyrophosphate phospho-hydrolase 2; Short=PPase 2; Flags: Precursor |
| gi20138778 | EIF3I | 2 | 0.883168148 | -1.132287213 | -0.179239954 | RecName: Full=Eukaryotic translation initiation factor 3 subunit I; Short=eIF3i; AltName: Full=Eukaryotic translation initiation factor 3 subunit 2; AltName: Full=TGF-beta receptor-interacting protein 1; Short=TRIP-1; AltName: Full=eIF-3-beta; AltName: Full=eIF3 p36 |
| gi8928084 | FUS | 3 | 0.881440463 | -1.134506574 | -0.182064969 | RecName: Full=RNA-binding protein FUS; AltName: Full=Protein pigpen |
| gi54036156 | PRKAR1A | 2 | 0.880188975 | -1.136119662 | -0.184114795 | RecName: Full=cAMP-dependent protein kinase type I-alpha regulatory subunit |
| gi28376965 | ACOT7 | 4 | 0.878684287 | -1.13806519 | -0.186583199 | RecName: Full=Cytosolic acyl coenzyme A thioester hydrolase; AltName: Full=Acyl-CoA thioesterase 7; AltName: Full=Brain acyl-CoA hydrolase; Short=BACH; AltName: Full=CTE-IIa; Short=CTE-II; AltName: Full=Long chain acyl-CoA thioester hydrolase |
| gi54041237 | PRKAR2B | 2 | 0.877690009 | -1.13935443 | -0.18821661 | RecName: Full=cAMP-dependent protein kinase type II-beta regulatory subunit |
| gi41688584 | HOOK3 | 2 | 0.873165487 | -1.145258275 | -0.195672987 | RecName: Full=Protein Hook homolog 3; Short=mHK3 |
| gi549057 | CCT4 | 2 | 0.870396604 | -1.148901542 | -0.200255167 | RecName: Full=T-complex protein 1 subunit delta; Short=TCP-1-delta; AltName: Full=A45; AltName: Full=CCT-delta |
| gi341940472 | DYNC1H1 | 24 | 0.870058987 | -1.14934736 | -0.20081488 | RecName: Full=Cytoplasmic dynein 1 heavy chain 1; AltName: Full=Cytoplasmic dynein heavy chain 1; AltName: Full=Dynein heavy chain, cytosolic |
| gi341942254 | PGM1 | 5 | 0.869947583 | -1.149494544 | -0.200999618 | RecName: Full=Phosphoglucomutase-1; Short=PGM 1; AltName: Full=Glucose phosphomutase 1 |
| gi118572640 | DLD | 6 | 0.869761755 | -1.149740137 | -0.201307822 | RecName: Full=Dihydrolipoyl dehydrogenase, mitochondrial; AltName: Full=Dihydrolipoamide dehydrogenase; Flags: Precursor |
| gi6685313 | CLDN11 | 2 | 0.868983091 | -1.150770377 | -0.202599989 | RecName: Full=Claudin-11; AltName: Full=Oligodendrocyte transmembrane protein; AltName: Full=Oligodendrocyte-specific protein |
| gi138536 | VIM | 4 | 0.868761658 | -1.151063691 | -0.202967663 | RecName: Full=Vimentin |
| gi1170151 | HIST1H1E | 4 | 0.868494935 | -1.151417192 | -0.203410659 | RecName: Full=Histone H1.4; AltName: Full=H1 VAR.2; AltName: Full=H1e |
| gi399833 | SLC2A3 | 3 | 0.865899783 | -1.154868057 | -0.207728034 | RecName: Full=Solute carrier family 2, facilitated glucose transporter member 3; AltName: Full=Glucose transporter type 3, brain; Short=GLUT-3 |
| gi18202285 | EEF2 | 9 | 0.865754599 | -1.155061725 | -0.207969949 | RecName: Full=Elongation factor 2; Short=EF-2 |
| gi341940423 | DYNC1I1 | 4 | 0.864588917 | -1.156619037 | -0.209913752 | RecName: Full=Cytoplasmic dynein 1 intermediate chain 1; AltName: Full=Cytoplasmic dynein intermediate chain 1; AltName: Full=Dynein intermediate chain 1, cytosolic; Short=DH IC-1 |
| gi73920250 | VBP1 | 2 | 0.862865815 | -1.15892875 | -0.212791873 | RecName: Full=Prefoldin subunit 3; AltName: Full=Von Hippel-Lindau-binding protein 1; Short=VBP-1; Short=VHL-binding protein 1 |
| gi146291096 | ATP6V1C1 | 6 | 0.862775794 | -1.159049671 | -0.212942394 | RecName: Full=V-type proton ATPase subunit C 1; Short=V-ATPase subunit C 1; AltName: Full=Vacuolar proton pump subunit C 1 |
| gi158518416 | IDH2 | 2 | 0.862111526 | -1.159942733 | -0.214053581 | RecName: Full=Isocitrate dehydrogenase [NADP], mitochondrial; Short=IDH; AltName: Full=ICD-M; AltName: Full=IDP; AltName: Full=NADP(+)-specific ICDH; AltName: Full=Oxalosuccinate decarboxylase; Flags: Precursor |
| gi1729865 | TCP1 | 3 | 0.862102006 | -1.159955543 | -0.214069513 | RecName: Full=T-complex protein 1 subunit alpha; Short=TCP-1-alpha; AltName: Full=CCT-alpha; AltName: Full=Tailless complex polypeptide 1A; Short=TCP-1-A; AltName: Full=Tailless complex polypeptide 1B; Short=TCP-1-B |
| gi9910833 | PSMA1 | 2 | 0.861939104 | -1.160174767 | -0.214342148 | RecName: Full=Proteasome subunit alpha type-1; AltName: Full=Macropain subunit C2; AltName: Full=Multicatalytic endopeptidase complex subunit C2; AltName: Full=Proteasome component C2; AltName: Full=Proteasome nu chain |
| gi134614 | SOD1 | 7 | 0.861896183 | -1.160232543 | -0.214413991 | RecName: Full=Superoxide dismutase [Cu-Zn] |
| gi51338599 | RAN | 4 | 0.86182806 | -1.160324252 | -0.214528023 | RecName: Full=GTP-binding nuclear protein Ran; AltName: Full=GTPase Ran; AltName: Full=Ras-like protein TC4; AltName: Full=Ras-related nuclear protein |
| gi130914 | PRNP | 2 | 0.861630826 | -1.160589861 | -0.214858231 | RecName: Full=Major prion protein; Short=PrP; AltName: Full=PrP27-30; AltName: Full=PrP33-35C; AltName: CD_antigen=CD230; Flags: Precursor |
| gi1351942 | ANXA5 | 3 | 0.861619015 | -1.16060577 | -0.214878006 | RecName: Full=Annexin A5; AltName: Full=Anchorin CII; AltName: Full=Annexin V; AltName: Full=Annexin-5; AltName: Full=Calphobindin I; Short=CBP-I; AltName: Full=Endonexin II; AltName: Full=Lipocortin V; AltName: Full=Placental anticoagulant protein 4; Short=PP4; AltName: Full=Placental anticoagulant protein I; Short=PAP-I; AltName: Full=Thromboplastin inhibitor; AltName: Full=Vascular anticoagulant-alpha; Short=VAC-alpha |
| gi13629369 | PFKP | 13 | 0.85977949 | -1.163088921 | -0.217961399 | RecName: Full=6-phosphofructokinase type C; AltName: Full=Phosphofructo-1-kinase isozyme C; Short=PFK-C; AltName: Full=Phosphofructokinase 1; AltName: Full=Phosphohexokinase |
| gi52782768 | C21orf33 | 5 | 0.859189468 | -1.163887637 | -0.218951785 | RecName: Full=ES1 protein homolog, mitochondrial; Flags: Precursor |
| gi81913084 | NECAB1 | 2 | 0.858512354 | -1.164805603 | -0.2200892 | RecName: Full=N-terminal EF-hand calcium-binding protein 1; Short=EF-hand calcium-binding protein 1 |
| gi20140237 | EFHD2 | 3 | 0.857668556 | -1.165951571 | -0.221507866 | RecName: Full=EF-hand domain-containing protein D2; AltName: Full=Swiprosin-1 |
| gi54039315 | RPS10 | 2 | 0.856491012 | -1.167554575 | -0.223489988 | RecName: Full=40S ribosomal protein S10 |
| gi22654291 | CCT2 | 4 | 0.855442268 | -1.16898596 | -0.225257602 | RecName: Full=T-complex protein 1 subunit beta; Short=TCP-1-beta; AltName: Full=CCT-beta |
| gi341940637 | CAP1 | 3 | 0.853688671 | -1.171387222 | -0.228218062 | RecName: Full=Adenylyl cyclase-associated protein 1; Short=CAP 1 |
| gi20138079 | EPB41L3 | 8 | 0.852133651 | -1.173524832 | -0.23084837 | RecName: Full=Band 4.1-like protein 3; AltName: Full=4.1B; AltName: Full=Differentially expressed in adenocarcinoma of the lung protein 1; Short=DAL-1; Short=DAL1P; Short=mDAL-1 |
| gi131884 | NRAS | 2 | 0.852048432 | -1.173642205 | -0.230992657 | RecName: Full=GTPase NRas; AltName: Full=Transforming protein N-Ras; Flags: Precursor |
| gi41019154 | PLP1 (includes EG:18823) | 8 | 0.85189163 | -1.173858229 | -0.231258179 | RecName: Full=Myelin proteolipid protein; Short=PLP; AltName: Full=Lipophilin |
| gi81892818 | PPP1R9B | 2 | 0.851875832 | -1.173879998 | -0.231284934 | RecName: Full=Neurabin-2; AltName: Full=Neurabin-II; AltName: Full=Protein phosphatase 1 regulatory subunit 9B; AltName: Full=Spinophilin |
| gi118542 | GLUD1 | 13 | 0.851852506 | -1.173912142 | -0.231324438 | RecName: Full=Glutamate dehydrogenase 1, mitochondrial; Short=GDH 1; Flags: Precursor |
| gi32470593 | ALDOC | 11 | 0.851267882 | -1.174718348 | -0.232314896 | RecName: Full=Fructose-bisphosphate aldolase C; AltName: Full=Aldolase 3; AltName: Full=Brain-type aldolase; AltName: Full=Scrapie-responsive protein 2; AltName: Full=Zebrin II |
| gi158518557 | FMN1 | 3 | 0.850634463 | -1.175593094 | -0.233388789 | RecName: Full=Formin-1; AltName: Full=Limb deformity protein |
| gi54038837 | PHB | 6 | 0.84843545 | -1.178640049 | -0.237123194 | RecName: Full=Prohibitin; AltName: Full=B-cell receptor-associated protein 32; Short=BAP 32 |
| gi160177562 | RTTN | 2 | 0.846625055 | -1.181160413 | -0.24020491 | RecName: Full=Rotatin |
| gi341941734 | PSMC4 | 2 | 0.843103871 | -1.186093475 | -0.246217712 | RecName: Full=26S protease regulatory subunit 6B; AltName: Full=26S proteasome AAA-ATPase subunit RPT3; AltName: Full=CIP21; AltName: Full=MB67-interacting protein; AltName: Full=MIP224; AltName: Full=Proteasome 26S subunit ATPase 4; AltName: Full=Tat-binding protein 7; Short=TBP-7 |
| gi51338761 | CNP | 20 | 0.841370324 | -1.188537284 | -0.249187161 | RecName: Full=2',3'-cyclic-nucleotide 3'-phosphodiesterase; Short=CNP; Short=CNPase |
| gi13638207 | PFKM | 11 | 0.838900377 | -1.192036655 | -0.2534286 | RecName: Full=6-phosphofructokinase, muscle type; AltName: Full=Phosphofructo-1-kinase isozyme A; Short=PFK-A; Short=Phosphofructokinase-M; AltName: Full=Phosphofructokinase 1; AltName: Full=Phosphohexokinase |
| gi52000925 | KCNA2 | 4 | 0.837918856 | -1.193432983 | -0.255117554 | RecName: Full=Potassium voltage-gated channel subfamily A member 2; AltName: Full=MK2; AltName: Full=Voltage-gated potassium channel subunit Kv1.2 |
| gi94730399 | NEFH | 10 | 0.837253422 | -1.194381502 | -0.256263727 | RecName: Full=Neurofilament heavy polypeptide; Short=NF-H; AltName: Full=200 kDa neurofilament protein; AltName: Full=Neurofilament triplet H protein |
| gi146345472 | OGDH | 13 | 0.837081507 | -1.194626798 | -0.25655999 | RecName: Full=2-oxoglutarate dehydrogenase, mitochondrial; AltName: Full=2-oxoglutarate dehydrogenase complex component E1; Short=OGDC-E1; AltName: Full=Alpha-ketoglutarate dehydrogenase; Flags: Precursor |
| gi32130449 | RPL12 | 2 | 0.837032338 | -1.194696972 | -0.256644734 | RecName: Full=60S ribosomal protein L12 |
| gi73621117 | KIAA1045 | 5 | 0.836750071 | -1.195099988 | -0.257131327 | RecName: Full=Protein KIAA1045 |
| gi68565610 | IDH3A | 10 | 0.834965961 | -1.197653614 | -0.260210711 | RecName: Full=Isocitrate dehydrogenase [NAD] subunit alpha, mitochondrial; AltName: Full=Isocitric dehydrogenase subunit alpha; AltName: Full=NAD(+)-specific ICDH subunit alpha; Flags: Precursor |
| gi37077864 | AUH (includes EG:11992) | 2 | 0.834519172 | -1.198294819 | -0.260982902 | RecName: Full=Methylglutaconyl-CoA hydratase, mitochondrial; AltName: Full=AU-specific RNA-binding enoyl-CoA hydratase; Short=AU-binding enoyl-CoA hydratase; Short=muAUH; Flags: Precursor |
| gi94730376 | DPYSL2 | 23 | 0.83357381 | -1.199653813 | -0.262618144 | RecName: Full=Dihydropyrimidinase-related protein 2; Short=DRP-2; AltName: Full=Unc-33-like phosphoprotein 2; Short=ULIP-2 |
| gi62510641 | QDPR | 2 | 0.833512665 | -1.199741818 | -0.262723974 | RecName: Full=Dihydropteridine reductase; AltName: Full=HDHPR; AltName: Full=Quinoid dihydropteridine reductase |
| gi29428127 | SLC32A1 | 2 | 0.831277255 | -1.202968076 | -0.266598357 | RecName: Full=Vesicular inhibitory amino acid transporter; AltName: Full=GABA and glycine transporter; AltName: Full=Solute carrier family 32 member 1; AltName: Full=Vesicular GABA transporter; Short=mVGAT; Short=mVIAAT |
| gi67460420 | CDS2 | 2 | 0.825578591 | -1.21127172 | -0.276522536 | RecName: Full=Phosphatidate cytidylyltransferase 2; AltName: Full=CDP-DAG synthase 2; AltName: Full=CDP-DG synthase 2; AltName: Full=CDP-diacylglycerol synthase 2; Short=CDS 2; AltName: Full=CDP-diglyceride pyrophosphorylase 2; AltName: Full=CDP-diglyceride synthase 2; AltName: Full=CTP:phosphatidate cytidylyltransferase 2 |
| gi18202587 | ICAM5 | 3 | 0.825506173 | -1.21137798 | -0.276649092 | RecName: Full=Intercellular adhesion molecule 5; Short=ICAM-5; AltName: Full=Telencephalin; Flags: Precursor |
| gi61229841 | PDXK | 3 | 0.825284135 | -1.211703894 | -0.277037188 | RecName: Full=Pyridoxal kinase; AltName: Full=Pyridoxine kinase |
| gi81906751 | HYOU1 | 2 | 0.824908011 | -1.21225638 | -0.277694847 | RecName: Full=Hypoxia up-regulated protein 1; Short=GRP-170; AltName: Full=140 kDa Ca(2+)-binding protein; Short=CBP-140; Flags: Precursor |
| gi464506 | PC | 7 | 0.824457989 | -1.212918079 | -0.278482113 | RecName: Full=Pyruvate carboxylase, mitochondrial; AltName: Full=Pyruvic carboxylase; Short=PCB; Flags: Precursor |
| gi2829482 | SLC8A1 | 3 | 0.823210152 | -1.214756642 | -0.280667321 | RecName: Full=Sodium/calcium exchanger 1; AltName: Full=Na(+)/Ca(2+)-exchange protein 1; Flags: Precursor |
| gi205830863 | MAP6 | 10 | 0.822127828 | -1.216355859 | -0.282565368 | RecName: Full=Microtubule-associated protein 6; Short=MAP-6; AltName: Full=Stable tubule-only polypeptide; Short=STOP |
| gi81900953 | COQ9 (includes EG:246650) | 2 | 0.821582281 | -1.217163543 | -0.283523027 | RecName: Full=Ubiquinone biosynthesis protein COQ9, mitochondrial; Flags: Precursor |
| gi3024764 | USP5 | 4 | 0.816683348 | -1.224464785 | -0.292151283 | RecName: Full=Ubiquitin carboxyl-terminal hydrolase 5; AltName: Full=Deubiquitinating enzyme 5; AltName: Full=Isopeptidase T; AltName: Full=Ubiquitin thiolesterase 5; AltName: Full=Ubiquitin-specific-processing protease 5 |
| gi325530087 | KIF7 | 2 | 0.815824145 | -1.225754357 | -0.293669889 | RecName: Full=Kinesin-like protein KIF7 |
| gi51702788 | RAC1 | 2 | 0.812747526 | -1.230394394 | -0.299120835 | RecName: Full=Ras-related C3 botulinum toxin substrate 1; AltName: Full=p21-Rac1; Flags: Precursor |
| gi81910752 | GIT1 (includes EG:216963) | 2 | 0.811701357 | -1.2319802 | -0.30097907 | RecName: Full=ARF GTPase-activating protein GIT1; Short=ARF GAP GIT1; AltName: Full=G protein-coupled receptor kinase-interactor 1; AltName: Full=GRK-interacting protein 1 |
| gi41018346 | SYNJ1 | 7 | 0.810864933 | -1.233251013 | -0.302466472 | RecName: Full=Synaptojanin-1; AltName: Full=Synaptic inositol-1,4,5-trisphosphate 5-phosphatase 1 |
| gi47606758 | NCALD | 8 | 0.809620301 | -1.235146893 | -0.304682629 | RecName: Full=Neurocalcin-delta |
| gi134047901 | RTN3 | 2 | 0.807816212 | -1.237905337 | -0.307900995 | RecName: Full=Reticulon-3 |
| gi125306 | CKM | 3 | 0.807694071 | -1.238092535 | -0.308119145 | RecName: Full=Creatine kinase M-type; AltName: Full=Creatine kinase M chain; AltName: Full=M-CK |
| gi47117304 | NDUFS4 | 2 | 0.805262643 | -1.241830859 | -0.312468687 | RecName: Full=NADH dehydrogenase [ubiquinone] iron-sulfur protein 4, mitochondrial; AltName: Full=Complex I-18 kDa; Short=CI-18 kDa; AltName: Full=Complex I-AQDQ; Short=CI-AQDQ; AltName: Full=NADH-ubiquinone oxidoreductase 18 kDa subunit; Flags: Precursor |
| gi117949769 | PCSK1N | 4 | 0.802907043 | -1.245474191 | -0.316695126 | RecName: Full=ProSAAS; AltName: Full=IA-4; AltName: Full=Proprotein convertase subtilisin/kexin type 1 inhibitor; Short=Proprotein convertase 1 inhibitor; AltName: Full=pro-SAAS; Contains: RecName: Full=KEP; Contains: RecName: Full=Big SAAS; Short=b-SAAS; Contains: RecName: Full=Little SAAS; Short=l-SAAS; Contains: RecName: Full=Big PEN-LEN; Short=b-PEN-LEN; AltName: Full=SAAS CT(1-49); Contains: RecName: Full=PEN; Contains: RecName: Full=PEN-20; Contains: RecName: Full=PEN-19; Contains: RecName: Full=Little LEN; Short=l-LEN; Contains: RecName: Full=Big LEN; Short=b-LEN; AltName: Full=SAAS CT(25-40); Flags: Precursor |
| gi342187361 | WDR7 | 2 | 0.802589305 | -1.245967263 | -0.317266163 | RecName: Full=WD repeat-containing protein 7; AltName: Full=TGF-beta resistance-associated protein TRAG |
| gi3334470 | MAG | 7 | 0.800667336 | -1.248958157 | -0.320725144 | RecName: Full=Myelin-associated glycoprotein; AltName: Full=Siglec-4a; Flags: Precursor |
| gi134034087 | DDB1 | 2 | 0.796804091 | -1.255013637 | -0.32770304 | RecName: Full=DNA damage-binding protein 1; AltName: Full=DDB p127 subunit; AltName: Full=Damage-specific DNA-binding protein 1; AltName: Full=UV-damaged DNA-binding factor |
| gi81881275 | MOBP | 2 | 0.795974341 | -1.256321905 | -0.32920617 | RecName: Full=Myelin-associated oligodendrocyte basic protein |
| gi1352217 | GAD2 | 3 | 0.793561839 | -1.26014124 | -0.333585444 | RecName: Full=Glutamate decarboxylase 2; AltName: Full=65 kDa glutamic acid decarboxylase; Short=GAD-65; AltName: Full=Glutamate decarboxylase 65 kDa isoform |
| gi94730353 | INA | 21 | 0.789968011 | -1.265874043 | -0.340133861 | RecName: Full=Alpha-internexin; Short=Alpha-Inx; AltName: Full=66 kDa neurofilament protein; Short=NF-66; Short=Neurofilament-66 |
| gi51703328 | TPT1 (includes EG:100043703) | 2 | 0.788227413 | -1.268669401 | -0.343316171 | RecName: Full=Translationally-controlled tumor protein; Short=TCTP; AltName: Full=21 kDa polypeptide; AltName: Full=p21; AltName: Full=p23 |
| gi2829481 | IDH3G | 4 | 0.787293071 | -1.270175029 | -0.345027313 | RecName: Full=Isocitrate dehydrogenase [NAD] subunit gamma 1, mitochondrial; AltName: Full=Isocitric dehydrogenase subunit gamma; AltName: Full=NAD(+)-specific ICDH subunit gamma; Flags: Precursor |
| gi341941233 | PCCB | 2 | 0.785617942 | -1.272883353 | -0.348100217 | RecName: Full=Propionyl-CoA carboxylase beta chain, mitochondrial; Short=PCCase subunit beta; AltName: Full=Propanoyl-CoA:carbon dioxide ligase subunit beta; Flags: Precursor |
| gi60390207 | MAPRE2 | 3 | 0.784870248 | -1.274095945 | -0.349473923 | RecName: Full=Microtubule-associated protein RP/EB family member 2; AltName: Full=APC-binding protein EB2; AltName: Full=End-binding protein 2; Short=EB2 |
| gi41688724 | MRPS36 | 4 | 0.784103004 | -1.275342647 | -0.350884909 | RecName: Full=28S ribosomal protein S36, mitochondrial; Short=MRP-S36; Short=S36mt |
| gi126752 | MARCKS | 2 | 0.780953599 | -1.280485807 | -0.356691262 | RecName: Full=Myristoylated alanine-rich C-kinase substrate; Short=MARCKS |
| gi55977306 | PSMC6 | 2 | 0.780564144 | -1.281124693 | -0.357410901 | RecName: Full=26S protease regulatory subunit 10B; AltName: Full=26S proteasome AAA-ATPase subunit RPT4; AltName: Full=Proteasome 26S subunit ATPase 6; AltName: Full=Proteasome subunit p42 |
| gi48427970 | ABAT | 14 | 0.779262228 | -1.283265073 | -0.359819206 | RecName: Full=4-aminobutyrate aminotransferase, mitochondrial; AltName: Full=(S)-3-amino-2-methylpropionate transaminase; AltName: Full=GABA aminotransferase; Short=GABA-AT; AltName: Full=Gamma-amino-N-butyrate transaminase; Short=GABA transaminase; Short=GABA-T; AltName: Full=L-AIBAT; Flags: Precursor |
| gi27151644 | NTM | 2 | 0.778643236 | -1.284285221 | -0.36096564 | RecName: Full=Neurotrimin; Flags: Precursor |
| gi146345383 | CA2 | 8 | 0.778597998 | -1.284359839 | -0.361049459 | RecName: Full=Carbonic anhydrase 2; AltName: Full=Carbonate dehydratase II; AltName: Full=Carbonic anhydrase II; Short=CA-II |
| gi51317403 | HPCAL1 | 6 | 0.778535464 | -1.284463003 | -0.361165336 | RecName: Full=Hippocalcin-like protein 1; AltName: Full=Neural visinin-like protein 3; Short=NVL-3; Short=NVP-3; AltName: Full=Visinin-like protein 3; Short=VILIP-3 |
| gi2497313 | MOG | 5 | 0.772094275 | -1.295178624 | -0.37315108 | RecName: Full=Myelin-oligodendrocyte glycoprotein; Flags: Precursor |
| gi9910829 | PSMA6 | 2 | 0.770593324 | -1.297701354 | -0.375958407 | RecName: Full=Proteasome subunit alpha type-6; AltName: Full=Macropain iota chain; AltName: Full=Multicatalytic endopeptidase complex iota chain; AltName: Full=Proteasome iota chain |
| gi52782785 | SDHA (includes EG:157074) | 3 | 0.76877223 | -1.300775393 | -0.379371871 | RecName: Full=Succinate dehydrogenase [ubiquinone] flavoprotein subunit, mitochondrial; AltName: Full=Flavoprotein subunit of complex II; Short=Fp; Flags: Precursor |
| gi13124070 | COPB1 | 2 | 0.768675583 | -1.300938943 | -0.379553253 | RecName: Full=Coatomer subunit beta; AltName: Full=Beta-coat protein; Short=Beta-COP |
| gi67460966 | NONO | 7 | 0.768583335 | -1.301095086 | -0.379726401 | RecName: Full=Non-POU domain-containing octamer-binding protein; Short=NonO protein |
| gi67460396 | TUFM | 6 | 0.762638196 | -1.311237761 | -0.390929307 | RecName: Full=Elongation factor Tu, mitochondrial; Flags: Precursor |
| gi93139504 | ADSS | 2 | 0.762460912 | -1.311542644 | -0.391264716 | RecName: Full=Adenylosuccinate synthetase isozyme 2; Short=AMPSase 2; Short=AdSS 2; AltName: Full=Adenylosuccinate synthetase, acidic isozyme; AltName: Full=Adenylosuccinate synthetase, liver isozyme; Short=L-type adenylosuccinate synthetase; AltName: Full=IMP--aspartate ligase 2 |
| gi90110719 | IGSF8 | 2 | 0.761739825 | -1.312784192 | -0.392629772 | RecName: Full=Immunoglobulin superfamily member 8; Short=IgSF8; AltName: Full=CD81 partner 3; AltName: Full=Glu-Trp-Ile EWI motif-containing protein 2; Short=EWI-2; AltName: Full=Keratinocyte-associated transmembrane protein 4; Short=KCT-4; AltName: Full=Prostaglandin regulatory-like protein; AltName: CD_antigen=CD316; Flags: Precursor |
| gi166897986 | COX5A (includes EG:12858) | 4 | 0.761292744 | -1.313555144 | -0.393476768 | RecName: Full=Cytochrome c oxidase subunit 5A, mitochondrial; AltName: Full=Cytochrome c oxidase polypeptide Va; Flags: Precursor |
| gi3122044 | DPYSL4 | 5 | 0.760779063 | -1.314442062 | -0.394450552 | RecName: Full=Dihydropyrimidinase-related protein 4; Short=DRP-4; AltName: Full=Collapsin response mediator protein 3; Short=CRMP-3; AltName: Full=UNC33-like phosphoprotein 4; Short=ULIP-4 |
| gi342187017 | SLC17A7 | 4 | 0.759990745 | -1.315805498 | -0.395946246 | RecName: Full=Vesicular glutamate transporter 1; Short=VGluT1; AltName: Full=Brain-specific Na(+)-dependent inorganic phosphate cotransporter; AltName: Full=Solute carrier family 17 member 7 |
| gi52000877 | UQCRFS1 | 3 | 0.759641434 | -1.316410553 | -0.396609496 | RecName: Full=Cytochrome b-c1 complex subunit Rieske, mitochondrial; AltName: Full=Complex III subunit 5; AltName: Full=Cytochrome b-c1 complex subunit 5; AltName: Full=Rieske iron-sulfur protein; Short=RISP; AltName: Full=Ubiquinol-cytochrome c reductase iron-sulfur subunit; Contains: RecName: Full=Cytochrome b-c1 complex subunit 11; AltName: Full=Complex III subunit IX; AltName: Full=Ubiquinol-cytochrome c reductase 8 kDa protein; Flags: Precursor |
| gi417208 | CKB | 20 | 0.75865446 | -1.318123142 | -0.398485156 | RecName: Full=Creatine kinase B-type; AltName: Full=B-CK; AltName: Full=Creatine kinase B chain |
| gi47606029 | PIP4K2B | 2 | 0.755014716 | -1.324477494 | -0.40542333 | RecName: Full=Phosphatidylinositol-5-phosphate 4-kinase type-2 beta; AltName: Full=1-phosphatidylinositol-5-phosphate 4-kinase 2-beta; AltName: Full=Diphosphoinositide kinase 2-beta; AltName: Full=Phosphatidylinositol-5-phosphate 4-kinase type II beta; Short=PI(5)P 4-kinase type II beta; Short=PIP4KII-beta; AltName: Full=PtdIns(5)P-4-kinase isoform 2-beta |
| gi146345468 | NEFM | 20 | 0.751708525 | -1.330302859 | -0.41175473 | RecName: Full=Neurofilament medium polypeptide; Short=NF-M; AltName: Full=160 kDa neurofilament protein; AltName: Full=Neurofilament 3; AltName: Full=Neurofilament triplet M protein |
| gi20978552 | PSMD6 | 2 | 0.7517035 | -1.330311752 | -0.411764373 | RecName: Full=26S proteasome non-ATPase regulatory subunit 6; AltName: Full=26S proteasome regulatory subunit RPN7; AltName: Full=26S proteasome regulatory subunit S10; AltName: Full=p42A |
| gi97536879 | NEFL | 20 | 0.743754195 | -1.344530231 | -0.427102194 | RecName: Full=Neurofilament light polypeptide; Short=NF-L; AltName: Full=68 kDa neurofilament protein; AltName: Full=Neurofilament triplet L protein |
| gi32363402 | NDUFA8 | 3 | 0.741669619 | -1.348309239 | -0.431151421 | RecName: Full=NADH dehydrogenase [ubiquinone] 1 alpha subcomplex subunit 8; AltName: Full=Complex I-19kD; Short=CI-19kD; AltName: Full=Complex I-PGIV; Short=CI-PGIV; AltName: Full=NADH-ubiquinone oxidoreductase 19 kDa subunit |
| gi14916536 | BIN1 | 9 | 0.738410262 | -1.354260702 | -0.437505492 | RecName: Full=Myc box-dependent-interacting protein 1; AltName: Full=Amphiphysin II; AltName: Full=Amphiphysin-like protein; AltName: Full=Bridging integrator 1; AltName: Full=SH3 domain-containing protein 9 |
| gi59798430 | PTMS | 2 | 0.725077744 | -1.379162453 | -0.463792404 | RecName: Full=Parathymosin |
| gi76363295 | PHB2 | 3 | 0.724106174 | -1.381012946 | -0.465726843 | RecName: Full=Prohibitin-2; AltName: Full=B-cell receptor-associated protein BAP37; AltName: Full=Repressor of estrogen receptor activity |
| gi47117273 | NDUFS2 | 5 | 0.723246298 | -1.382654847 | -0.467441061 | RecName: Full=NADH dehydrogenase [ubiquinone] iron-sulfur protein 2, mitochondrial; AltName: Full=Complex I-49kD; Short=CI-49kD; AltName: Full=NADH-ubiquinone oxidoreductase 49 kDa subunit; Flags: Precursor |
| gi150416330 | ZXDB | 2 | 0.717566234 | -1.393599577 | -0.478816091 | RecName: Full=Zinc finger X-linked protein ZXDA/ZXDB |
| gi18203409 | DSTN | 3 | 0.716091448 | -1.396469687 | -0.481784257 | RecName: Full=Destrin; AltName: Full=Actin-depolymerizing factor; Short=ADF; AltName: Full=Sid 23 |
| gi549058 | CCT5 | 2 | 0.715967467 | -1.396711507 | -0.482034061 | RecName: Full=T-complex protein 1 subunit epsilon; Short=TCP-1-epsilon; AltName: Full=CCT-epsilon |
| gi1710815 | S100B | 3 | 0.712680638 | -1.403153034 | -0.488672364 | RecName: Full=Protein S100-B; AltName: Full=S-100 protein beta chain; AltName: Full=S-100 protein subunit beta; AltName: Full=S100 calcium-binding protein B |
| gi77416393 | CLASP2 | 2 | 0.712660499 | -1.403192685 | -0.488713132 | RecName: Full=CLIP-associating protein 2; AltName: Full=Cytoplasmic linker-associated protein 2 |
| gi134047752 | EPB41L1 | 2 | 0.704480164 | -1.419486383 | -0.505369009 | RecName: Full=Band 4.1-like protein 1; AltName: Full=Neuronal protein 4.1; Short=4.1N |
| gi341941131 | PFKL | 11 | 0.702122301 | -1.424253294 | -0.510205743 | RecName: Full=6-phosphofructokinase, liver type; AltName: Full=Phosphofructo-1-kinase isozyme B; Short=PFK-B; AltName: Full=Phosphofructokinase 1; AltName: Full=Phosphohexokinase |
| gi3914939 | PSAP | 3 | 0.699565519 | -1.429458675 | -0.515468913 | RecName: Full=Sulfated glycoprotein 1; Short=SGP-1; AltName: Full=Prosaposin; Flags: Precursor |
| gi51702253 | CPLX1 | 5 | 0.695456557 | -1.437904339 | -0.523967699 | RecName: Full=Complexin-1; AltName: Full=921-S; AltName: Full=Complexin I; Short=CPX I; AltName: Full=Synaphin-2 |
| gi116961 | SPA3K | 2 | 0.683768807 | -1.462482626 | -0.548419486 | RecName: Full=Serine protease inhibitor A3K; Short=Serpin A3K; AltName: Full=Contrapsin; AltName: Full=SPI-2; Flags: Precursor |
| gi30315914 | AHSA1 | 2 | 0.674531997 | -1.482509362 | -0.568041215 | RecName: Full=Activator of 90 kDa heat shock protein ATPase homolog 1; Short=AHA1 |
| gi46396655 | SET | 4 | 0.669246114 | -1.494218612 | -0.579391237 | RecName: Full=Protein SET; AltName: Full=Phosphatase 2A inhibitor I2PP2A; Short=I-2PP2A; AltName: Full=Template-activating factor I; Short=TAF-I |
| gi37999666 | ATL1 | 2 | 0.668215483 | -1.49652324 | -0.581614683 | RecName: Full=Atlastin-1; AltName: Full=Spastic paraplegia 3A homolog |
| gi81888798 | ERMN | 2 | 0.666549353 | -1.500264003 | -0.585216395 | RecName: Full=Ermin; AltName: Full=Juxtanodin; Short=JN |
| gi59799776 | PRKACB | 4 | 0.666070907 | -1.501341659 | -0.586252327 | RecName: Full=cAMP-dependent protein kinase catalytic subunit beta; Short=PKA C-beta |
| gi266608 | NME2 | 5 | 0.665621264 | -1.502355851 | -0.587226573 | RecName: Full=Nucleoside diphosphate kinase B; Short=NDK B; Short=NDP kinase B; AltName: Full=Histidine protein kinase NDKB; AltName: Full=P18; AltName: Full=nm23-M2 |
| gi47605401 | CSRP1 | 3 | 0.662309771 | -1.509867503 | -0.594421953 | RecName: Full=Cysteine and glycine-rich protein 1; AltName: Full=Cysteine-rich protein 1; Short=CRP; Short=CRP1 |
| gi50401075 | ITPKA | 2 | 0.659802134 | -1.515605888 | -0.59989465 | RecName: Full=Inositol-trisphosphate 3-kinase A; AltName: Full=Inositol 1,4,5-trisphosphate 3-kinase A; Short=IP3 3-kinase A; Short=IP3K A; Short=InsP 3-kinase A |
| gi49036421 | NCS1 | 2 | 0.658139737 | -1.519434163 | -0.603534164 | RecName: Full=Neuronal calcium sensor 1; Short=NCS-1; AltName: Full=Frequenin homolog |
| gi56405010 | EEF1A1 | 6 | 0.654071638 | -1.528884517 | -0.612479438 | RecName: Full=Elongation factor 1-alpha 1; Short=EF-1-alpha-1; AltName: Full=Elongation factor Tu; Short=EF-Tu; AltName: Full=Eukaryotic elongation factor 1 A-1; Short=eEF1A-1 |
| gi341941270 | PLCB1 | 6 | 0.653319304 | -1.530645112 | -0.614139825 | RecName: Full=1-phosphatidylinositol-4,5-bisphosphate phosphodiesterase beta-1; AltName: Full=PLC-154; AltName: Full=Phosphoinositide phospholipase C-beta-1; AltName: Full=Phospholipase C-beta-1; Short=PLC-beta-1 |
| gi81873719 | ENPP6 | 2 | 0.648346699 | -1.542384655 | -0.625162604 | RecName: Full=Ectonucleotide pyrophosphatase/phosphodiesterase family member 6; Short=E-NPP 6; Short=NPP-6; Contains: RecName: Full=Ectonucleotide pyrophosphatase/phosphodiesterase family member 6 soluble form; Flags: Precursor |
| gi292630942 | SYNE1 | 3 | 0.647495933 | -1.544411246 | -0.627056965 | RecName: Full=Nesprin-1; AltName: Full=Enaptin; AltName: Full=Myocyte nuclear envelope protein 1; Short=Myne-1; AltName: Full=Nuclear envelope spectrin repeat protein 1; AltName: Full=Synaptic nuclear envelope protein 1; Short=Syne-1 |
| gi52001083 | PCP4 | 3 | 0.643356218 | -1.554348855 | -0.636310335 | RecName: Full=Purkinje cell protein 4; AltName: Full=Brain-specific antigen PCP-4; AltName: Full=Brain-specific polypeptide PEP-19 |
| gi17378829 | MBP | 9 | 0.631472374 | -1.583600552 | -0.663208475 | RecName: Full=Myelin basic protein; Short=MBP; AltName: Full=Myelin A1 protein |
| gi81896595 | NCEH1 | 2 | 0.620228829 | -1.612308156 | -0.689127508 | RecName: Full=Neutral cholesterol ester hydrolase 1; Short=NCEH; AltName: Full=Arylacetamide deacetylase-like 1; AltName: Full=Chlorpyrifos oxon-binding protein; Short=CPO-BP |
| gi45477158 | ACSL6 | 4 | 0.62021574 | -1.612342183 | -0.689157956 | RecName: Full=Long-chain-fatty-acid--CoA ligase 6; AltName: Full=Long-chain acyl-CoA synthetase 6; Short=LACS 6 |
| gi54037410 | EIF5A | 2 | 0.603597325 | -1.65673365 | -0.728341682 | RecName: Full=Eukaryotic translation initiation factor 5A-1; Short=eIF-5A-1; Short=eIF-5A1; AltName: Full=Eukaryotic initiation factor 5A isoform 1; Short=eIF-5A; AltName: Full=eIF-4D |
| gi21362640 | GLO1 | 4 | 0.602546916 | -1.659621805 | -0.730854517 | RecName: Full=Lactoylglutathione lyase; AltName: Full=Aldoketomutase; AltName: Full=Glyoxalase I; Short=Glx I; AltName: Full=Ketone-aldehyde mutase; AltName: Full=Methylglyoxalase; AltName: Full=S-D-lactoylglutathione methylglyoxal lyase |
| gi23831273 | PPP1R1B | 3 | 0.599315194 | -1.668571078 | -0.738613144 | RecName: Full=Protein phosphatase 1 regulatory subunit 1B; AltName: Full=DARPP-32; AltName: Full=Dopamine- and cAMP-regulated neuronal phosphoprotein |
| gi3183025 | HSD17B10 | 2 | 0.581469408 | -1.719780931 | -0.782224804 | RecName: Full=3-hydroxyacyl-CoA dehydrogenase type-2; AltName: Full=17-beta-hydroxysteroid dehydrogenase 10; Short=17-beta-HSD 10; AltName: Full=3-hydroxy-2-methylbutyryl-CoA dehydrogenase; AltName: Full=3-hydroxyacyl-CoA dehydrogenase type II; AltName: Full=Endoplasmic reticulum-associated amyloid beta-peptide-binding protein; AltName: Full=Mitochondrial ribonuclease P protein 2; Short=Mitochondrial RNase P protein 2; AltName: Full=Type II HADH |
| gi38258618 | SIRT2 | 3 | 0.562832436 | -1.776727737 | -0.829222622 | RecName: Full=NAD-dependent deacetylase sirtuin-2; AltName: Full=SIR2-like protein 2; Short=mSIR2L2 |
| gi341940739 | GPHN | 2 | 0.552411749 | -1.810243901 | -0.85618409 | RecName: Full=Gephyrin; Includes: RecName: Full=Molybdopterin adenylyltransferase; Short=MPT adenylyltransferase; AltName: Full=Domain G; Includes: RecName: Full=Molybdopterin molybdenumtransferase; Short=MPT Mo-transferase; AltName: Full=Domain E |
| gi81882085 | MORC1 | 2 | 0.539478005 | -1.853643692 | -0.890363955 | RecName: Full=MORC family CW-type zinc finger protein 1; AltName: Full=Protein microrchidia |
| gi341942004 | SLC6A11 | 3 | 0.532727317 | -1.877132951 | -0.908530835 | RecName: Full=Sodium- and chloride-dependent GABA transporter 3; Short=GAT-3; AltName: Full=Sodium- and chloride-dependent GABA transporter 4; Short=GAT-4; AltName: Full=Solute carrier family 6 member 11 |
| gi57012952 | PDHX | 2 | 0.532273827 | -1.878732243 | -0.909759468 | RecName: Full=Pyruvate dehydrogenase protein X component, mitochondrial; AltName: Full=Dihydrolipoamide dehydrogenase-binding protein of pyruvate dehydrogenase complex; AltName: Full=Lipoyl-containing pyruvate dehydrogenase complex component X; Flags: Precursor |
| gi38258077 | MMAA | 2 | 0.529694687 | -1.887879989 | -0.916767057 | RecName: Full=Methylmalonic aciduria type A homolog, mitochondrial; Flags: Precursor |
| gi8928304 | PTK6 | 2 | 0.526869463 | -1.89800334 | -0.924482531 | RecName: Full=Protein-tyrosine kinase 6; AltName: Full=SRC-related intestinal kinase |
| gi21362536 | DPYSL5 | 2 | 0.525357626 | -1.903465278 | -0.928628253 | RecName: Full=Dihydropyrimidinase-related protein 5; Short=DRP-5; AltName: Full=Collapsin response mediator protein 5; Short=CRMP-5 |
| gi398990 | DBI | 2 | 0.486164715 | -2.056916039 | -1.040482906 | RecName: Full=Acyl-CoA-binding protein; Short=ACBP; AltName: Full=Diazepam-binding inhibitor; Short=DBI; AltName: Full=Endozepine; Short=EP |
| gi1708288 | HPRT1 | 3 | 0.428615131 | -2.333095423 | -1.222245314 | RecName: Full=Hypoxanthine-guanine phosphoribosyltransferase; Short=HGPRT; Short=HGPRTase; AltName: Full=HPRT B |
| gi84028249 | PTPN11 | 2 | 0.3232305 | -3.093767449 | -1.629364757 | RecName: Full=Tyrosine-protein phosphatase non-receptor type 11; AltName: Full=Protein-tyrosine phosphatase SYP; AltName: Full=SH-PTP2; Short=SHP-2; Short=Shp2 |
| gi17380463 | NUCB1 | 2 | 0.280817949 | -3.561025937 | -1.832292944 | RecName: Full=Nucleobindin-1; AltName: Full=CALNUC; Flags: Precursor |
