# Supplementary material for: The effects of aging on the BTBR mouse model of autism spectrum disorder
Source: Front Aging Neurosci. 2014 Sep 1;6:225. doi: 10.3389/fnagi.2014.00225 (PMC4150363; doi:10.3389/fnagi.2014.00225)
Supplement: Supplementary file 5 [file Table3.DOCX]

**Table S3. *Textrous!-*based collective processing analysis of upregulated BTBR-specific cortical proteins.** Cosine similarity scores, Z-scores and probability values (p-Value) were calculated using collective processing of the upregulated (BTBR:WT iTRAQ ratio >1.2) BTBR-specific cortical proteins.

| **Word** | **Cosine Similarity** | **Z-score** | **p-Value** |
| --- | --- | --- | --- |
| metabotropic | 0.943659871 | 3.974441724 | 3.53378E-05 |
| glutamatergic | 0.824850058 | 3.475028175 | 0.000255427 |
| ionotropic | 0.81789328 | 3.445785562 | 0.000284475 |
| bursts | 0.816558085 | 3.440173112 | 0.000290857 |
| n-methyl-d-aspartic | 0.800413346 | 3.372309175 | 0.000373122 |
| bicuculline | 0.790877844 | 3.332226975 | 0.000431121 |
| excitatory | 0.785381016 | 3.309121217 | 0.000468149 |
| glutamate | 0.775817964 | 3.268923212 | 0.000539642 |
| postsynaptic | 0.753778762 | 3.176282076 | 0.000746605 |
| potentiation | 0.731919202 | 3.084396059 | 0.001021188 |
| ampa | 0.706618472 | 2.978045184 | 0.00145068 |
| transmission | 0.702601972 | 2.961161946 | 0.00153321 |
| epileptiform | 0.689528094 | 2.906206283 | 0.001830407 |
| ketamine | 0.677966592 | 2.857607853 | 0.002131602 |
| synapses | 0.676935386 | 2.853273209 | 0.002165431 |
| monocular | 0.671784668 | 2.831622317 | 0.002312892 |
| terminals | 0.662436228 | 2.792326424 | 0.002619168 |
| long-term | 0.653663207 | 2.755449287 | 0.002934607 |
| synaptic | 0.641275702 | 2.703378774 | 0.003435837 |
| propionic | 0.629099828 | 2.652197847 | 0.004000827 |
| nmda | 0.599604168 | 2.528213709 | 0.005735718 |
| spikes | 0.596402921 | 2.514757361 | 0.005951619 |
| presynaptic | 0.590554401 | 2.490173283 | 0.006387155 |
| plasticity | 0.589535531 | 2.485890493 | 0.006459398 |
| globus | 0.582692273 | 2.457125059 | 0.007005134 |
| post-synaptic | 0.580783853 | 2.449103072 | 0.007162672 |
| reticulata | 0.580056181 | 2.446044322 | 0.00722255 |
| kainate | 0.578160107 | 2.438074232 | 0.007384387 |
| occluded | 0.570579257 | 2.406208351 | 0.008064132 |
| mossy | 0.568405116 | 2.397069415 | 0.008264962 |
| amygdaloid | 0.567673594 | 2.393994483 | 0.008332874 |
| dendrites | 0.566780142 | 2.390238883 | 0.008424186 |
| n-methyl-d-aspartate | 0.562121372 | 2.370655882 | 0.008870016 |
| evoked | 0.56082815 | 2.36521986 | 0.009015035 |
| kainic | 0.558823215 | 2.356792175 | 0.009211623 |
| amygdala | 0.554213039 | 2.337413436 | 0.009719592 |
| subiculum | 0.549707529 | 2.318474658 | 0.010224661 |
| hippocampal | 0.548013057 | 2.311351995 | 0.010416427 |
| pyramidal | 0.546995649 | 2.30707535 | 0.01052741 |
| scaling | 0.544302059 | 2.295752922 | 0.010837941 |
| fragile | 0.539623704 | 2.276087597 | 0.011423005 |
| objects | 0.53452996 | 2.254676192 | 0.012066664 |
| nmdar | 0.526298843 | 2.220076935 | 0.013209384 |
| vo | 0.525392049 | 2.216265252 | 0.013345751 |
| monosynaptic | 0.519949687 | 2.193388446 | 0.01415369 |
| spines | 0.518697396 | 2.188124475 | 0.014334801 |
| ly | 0.514016133 | 2.168446926 | 0.015079343 |
| application | 0.513176139 | 2.164916036 | 0.015193843 |
| picrotoxin | 0.506145947 | 2.135364829 | 0.016380505 |
| learning | 0.505647313 | 2.133268836 | 0.016462363 |
| hypofunction | 0.505638642 | 2.133232387 | 0.016462363 |
| afferents | 0.499976162 | 2.109430322 | 0.017472291 |
| somatosensory | 0.488606457 | 2.061638099 | 0.019603868 |
| inspiratory | 0.485007639 | 2.046510574 | 0.020329042 |
| short-term | 0.484696977 | 2.045204716 | 0.020427429 |
| quantal | 0.480953957 | 2.029471042 | 0.021229145 |
| long-lasting | 0.477169039 | 2.013561253 | 0.022004767 |
| sedative | 0.47110903 | 1.98808818 | 0.023405845 |
| laminae | 0.469929381 | 1.983129563 | 0.023683715 |
| recordings | 0.466891791 | 1.970361142 | 0.024419185 |
| corticostriatal | 0.461158324 | 1.946260684 | 0.02582737 |
| excitation | 0.460251336 | 1.942448185 | 0.02606855 |
| thalamic | 0.459084749 | 1.93754447 | 0.026311611 |
| recording | 0.452824984 | 1.911231733 | 0.02800229 |
| l-glutamate | 0.451944623 | 1.90753116 | 0.028195608 |
| hemispheric | 0.45150531 | 1.905684522 | 0.028325103 |
| pallidus | 0.451349194 | 1.905028294 | 0.028390036 |
| maze | 0.449447711 | 1.897035465 | 0.028913969 |
| morris | 0.446036305 | 1.882695721 | 0.029850184 |
| hydrochloride | 0.442289924 | 1.86694792 | 0.030950794 |
| clonic | 0.437300331 | 1.845974309 | 0.032446103 |
| appetitive | 0.43447873 | 1.834113795 | 0.033326997 |
| deafferentation | 0.433978376 | 1.832010569 | 0.03347571 |
| acidin | 0.427702721 | 1.805631042 | 0.03545917 |
| terminalis | 0.425131312 | 1.794822199 | 0.03632685 |
| stereotaxic | 0.419783995 | 1.77234491 | 0.038197279 |
| high-frequency | 0.418665745 | 1.76764438 | 0.038530452 |
| pdz | 0.415956868 | 1.756257693 | 0.039544204 |
| clustering | 0.411214183 | 1.736321956 | 0.041281917 |
| transmitter | 0.411117038 | 1.735913612 | 0.041281917 |
| neostriatum | 0.406710873 | 1.717392426 | 0.042989586 |
| microvasculature | 0.405217707 | 1.711115946 | 0.043540556 |
| shell | 0.404695249 | 1.708919809 | 0.043725475 |
| burst | 0.402194962 | 1.698409926 | 0.044753881 |
| spatial | 0.397330585 | 1.677962661 | 0.04667355 |
| neurotransmitter | 0.394892157 | 1.6677128 | 0.047657863 |
| boutons | 0.391554702 | 1.653683905 | 0.049063756 |
| memories | 0.391035642 | 1.651502055 | 0.049267275 |
| receptor-mediated | 0.390228892 | 1.648110904 | 0.049676336 |
| associative | 0.389795377 | 1.646288634 | 0.04988188 |
| strength | 0.383004219 | 1.617742204 | 0.052831299 |
| miniature | 0.380921334 | 1.608986859 | 0.053808171 |
| gabaergic | 0.380724391 | 1.608159012 | 0.053917589 |
| facilities | 0.380665659 | 1.607912133 | 0.053917589 |
| hippocampus | 0.378214176 | 1.597607399 | 0.055021489 |
| reflex | 0.377850069 | 1.596076885 | 0.055244397 |
| instrumental | 0.377007036 | 1.592533221 | 0.055580096 |
| neuroplasticity | 0.375506726 | 1.586226711 | 0.056369655 |
| chelators | 0.374592171 | 1.582382407 | 0.056824785 |
| synapse | 0.371039242 | 1.567447774 | 0.058557346 |
