# Supplementary material for: The effects of aging on the BTBR mouse model of autism spectrum disorder
Source: Front Aging Neurosci. 2014 Sep 1;6:225. doi: 10.3389/fnagi.2014.00225 (PMC4150363; doi:10.3389/fnagi.2014.00225)
Supplement: Supplementary file 6 [file Table4.DOCX]

**Table S4. *Textrous!-*based collective analysis of downregulated BTBR-specific cortical proteins.** Cosine similarity scores, Z-scores and probability values (p-Value) were calculated using collective processing of the downregulated (BTBR:WT iTRAQ ratio <0.8) BTBR-specific cortical proteins.

| **Word** | **Cosine Similarity** | **Z-score** | **p-Value** |
| --- | --- | --- | --- |
| kinase | 0.694086128 | 3.064932752 | 0.00108835 |
| signal-regulated | 0.684304465 | 3.023170913 | 0.001251411 |
| kinases | 0.646215382 | 2.860553347 | 0.002111535 |
| signal-regulating | 0.538288395 | 2.399769783 | 0.008197536 |
| cascades | 0.510535657 | 2.28128222 | 0.011274223 |
| mekk | 0.458162965 | 2.057682208 | 0.019795067 |
| mixed-lineage | 0.457955704 | 2.056797328 | 0.019843113 |
| phosphorylation | 0.428497557 | 1.931028689 | 0.026741526 |
| sprague-dawley | 0.425093 | 1.916493272 | 0.027682548 |
| deprivation-induced | 0.419226877 | 1.891448443 | 0.029312172 |
| map | 0.417772185 | 1.885237781 | 0.029714919 |
| phenylephrine | 0.417253351 | 1.883022671 | 0.029850184 |
| inhibition | 0.416723533 | 1.880760661 | 0.029985959 |
| b-raf | 0.411068809 | 1.856618382 | 0.031655577 |
| c-raf | 0.404374023 | 1.828035653 | 0.033774776 |
| vasoconstrictor | 0.403285409 | 1.823387926 | 0.034151695 |
| reperfusion | 0.399914368 | 1.808995599 | 0.035225502 |
| min | 0.398988587 | 1.805043072 | 0.035537341 |
| cardioprotective | 0.39849505 | 1.802935962 | 0.035694107 |
| stress-activated | 0.392975481 | 1.779370713 | 0.037619881 |
| mitogen-activated | 0.387654161 | 1.756651862 | 0.039458905 |
| l-name | 0.387614624 | 1.756483064 | 0.039544204 |
| tractus | 0.38602205 | 1.749683729 | 0.040059157 |
| cascade | 0.382915235 | 1.736419488 | 0.041281917 |
| cyclo-oxygenase | 0.371054345 | 1.685780596 | 0.0458979 |
| mek-erk | 0.367550413 | 1.670820903 | 0.047360839 |
| intrathecal | 0.36416596 | 1.656371319 | 0.048860909 |
| pkc-epsilon | 0.362472912 | 1.649143017 | 0.049573817 |
| phosphospecific | 0.360280574 | 1.639783049 | 0.050502583 |
| endothelin | 0.359452162 | 1.636246225 | 0.050919794 |
| pkc-delta | 0.358664515 | 1.632883443 | 0.051234498 |
| activation | 0.356791224 | 1.624885617 | 0.052081279 |
| renovascular | 0.35580691 | 1.620683183 | 0.052508819 |
| phospho-specific | 0.355304343 | 1.618537522 | 0.052723632 |
| infusion | 0.350810219 | 1.599350305 | 0.054910301 |
| wistar | 0.350675575 | 1.598775457 | 0.054910301 |
| wky | 0.348126721 | 1.587893379 | 0.05614317 |
| lumbosacral | 0.345271585 | 1.575703658 | 0.057512902 |
| withdrawal-induced | 0.344919246 | 1.574199382 | 0.057743725 |
| activated | 0.343861275 | 1.569682481 | 0.058207556 |
| attenuated | 0.343845965 | 1.569617117 | 0.058207556 |
| allodynia | 0.34346189 | 1.567977347 | 0.058440566 |
| phosphorylations | 0.34309243 | 1.566399971 | 0.058674309 |
| mapk | 0.34260403 | 1.564314796 | 0.058908784 |
| compression | 0.33934475 | 1.550399624 | 0.060570758 |
| vasoconstriction | 0.338489176 | 1.546746838 | 0.060931623 |
| vasodilator | 0.337691133 | 1.54333967 | 0.061415388 |
| conscious | 0.336065846 | 1.53640067 | 0.06226919 |
| sham | 0.335192283 | 1.532671077 | 0.062637927 |
| monocrotaline | 0.334375803 | 1.529185196 | 0.063132222 |
| extracellular | 0.333856968 | 1.526970085 | 0.063380506 |
| eta | 0.333790894 | 1.526687987 | 0.063380506 |
| ischemia | 0.33246523 | 1.521028198 | 0.064129919 |
| cardiomyocyte | 0.332364502 | 1.520598148 | 0.064129919 |
| dawley | 0.331477059 | 1.516809297 | 0.064633342 |
| sprague | 0.329227451 | 1.507204821 | 0.065905329 |
| biphasic | 0.328163721 | 1.502663331 | 0.066419522 |
| mesenteric | 0.326310869 | 1.494752763 | 0.067457221 |
| ng-nitro-l-arginine | 0.321170466 | 1.472806323 | 0.070375518 |
| stimulus | 0.320296583 | 1.469075364 | 0.070916395 |
| fischer | 0.319635845 | 1.466254409 | 0.071324143 |
| natriuresis | 0.319565164 | 1.465952643 | 0.071324143 |
| mca | 0.318812776 | 1.462740398 | 0.071733689 |
| hypertonic | 0.318277617 | 1.460455592 | 0.072145037 |
| sustained | 0.315753184 | 1.449677774 | 0.07352926 |
| sapk | 0.315693234 | 1.449421823 | 0.073668791 |
| immobilization | 0.31235429 | 1.435166534 | 0.075643549 |
| shin | 0.312209449 | 1.43454815 | 0.075643549 |
| dbcamp | 0.312176308 | 1.434406658 | 0.075786131 |
| ipsilateral | 0.310591307 | 1.42763965 | 0.076645929 |
| inhibitor | 0.307845648 | 1.415917334 | 0.078387752 |
| adjuvant-induced | 0.307039708 | 1.412476452 | 0.078974981 |
| inhibitors | 0.306397066 | 1.409732757 | 0.079269841 |
| cystitis | 0.305327922 | 1.405168153 | 0.080010639 |
| pkc-alpha | 0.304754105 | 1.402718295 | 0.080308419 |
| mapkks | 0.303893834 | 1.399045455 | 0.080906492 |
| saline | 0.303723931 | 1.398320072 | 0.081056534 |
| eyelid | 0.303553656 | 1.397593099 | 0.081056534 |
| tachykinin | 0.300885089 | 1.386199916 | 0.082873452 |
| caldesmon | 0.30083194 | 1.385973003 | 0.082873452 |
| hypertrophy | 0.300047758 | 1.382625016 | 0.083332433 |
| wear | 0.298746324 | 1.37706867 | 0.084256124 |
| baroreceptor | 0.297057862 | 1.369859953 | 0.085343451 |
| capsaicin | 0.295392173 | 1.362748458 | 0.086441255 |
| stretch | 0.295330651 | 1.362485794 | 0.086598943 |
| mitogen | 0.294222159 | 1.357753198 | 0.087231842 |
| adventitial | 0.292932956 | 1.352249072 | 0.088187658 |
| pseudopodia | 0.292334302 | 1.349693178 | 0.088507991 |
| compressed | 0.291849289 | 1.347622464 | 0.088829191 |
| low-salt | 0.290678045 | 1.342621956 | 0.089635987 |
| arterioles | 0.290181539 | 1.340502172 | 0.089960226 |
| preconditioning | 0.288178772 | 1.331951555 | 0.091430094 |
| hyperalgesia | 0.287655191 | 1.329716179 | 0.091759136 |
| neurokinin | 0.28657652 | 1.3251109 | 0.092585576 |
| blocker | 0.282934774 | 1.309562829 | 0.095097918 |
| pathways | 0.281205269 | 1.302178878 | 0.096458193 |
| activations | 0.279790485 | 1.296138598 | 0.097487742 |
| erectile | 0.279277174 | 1.293947067 | 0.09783271 |
| pharmacological | 0.279032937 | 1.292904323 | 0.09800553 |
| deprivation | 0.278551151 | 1.290847382 | 0.098351839 |
