# Supplementary material for: The effects of aging on the BTBR mouse model of autism spectrum disorder
Source: Front Aging Neurosci. 2014 Sep 1;6:225. doi: 10.3389/fnagi.2014.00225 (PMC4150363; doi:10.3389/fnagi.2014.00225)
Supplement: Supplementary file 7 [file Table5.DOCX]

**Table S5. *Textrous!-*based collective analysis of upregulated BTBR-specific hippocampal proteins.** Cosine similarity scores, Z-scores and probability values (p-Value) were calculated using collective processing of the upregulated (BTBR:WT iTRAQ ratio >1.2) BTBR-specific hippocampal proteins.

| **Word** | **Cosine Similarity** | **Z-score** | **p-Value** |
| --- | --- | --- | --- |
| alpha-synuclein | 0.710909842 | 3.305774891 | 0.00047319 |
| lewy | 0.699544111 | 3.252841022 | 0.000570967 |
| misfolding | 0.672702194 | 3.127829573 | 0.000880001 |
| oligomers | 0.666393678 | 3.09844878 | 0.000974157 |
| msa | 0.665172797 | 3.092762744 | 0.000990721 |
| mptp | 0.639275984 | 2.972152948 | 0.001479333 |
| heat-shock | 0.593003078 | 2.756645124 | 0.002916718 |
| chaperones | 0.588031153 | 2.733489269 | 0.003138017 |
| heat | 0.587496028 | 2.730997019 | 0.003157124 |
| shock | 0.585172472 | 2.72017547 | 0.003264096 |
| hsp | 0.581449552 | 2.702836634 | 0.003435837 |
| parkinson | 0.578866369 | 2.690805916 | 0.00356191 |
| chaperone | 0.578580551 | 2.689474769 | 0.003583321 |
| substantia | 0.562100968 | 2.612724046 | 0.004487565 |
| parkinson's | 0.556333835 | 2.585864648 | 0.004854847 |
| refolding | 0.546174546 | 2.538549571 | 0.005558491 |
| nigra | 0.537747752 | 2.499303275 | 0.006227216 |
| dnaj | 0.524669682 | 2.438394491 | 0.007384387 |
| renaturation | 0.50607832 | 2.351808533 | 0.009336387 |
| bodies | 0.505937628 | 2.351153284 | 0.009361517 |
| solubility | 0.503235196 | 2.33856719 | 0.009667717 |
| neurotoxin | 0.500164453 | 2.32426575 | 0.010062747 |
| lb | 0.492896035 | 2.290414389 | 0.011010658 |
| folding | 0.489333405 | 2.27382207 | 0.011482993 |
| chaperonin | 0.488444394 | 2.26968166 | 0.011603792 |
| prion | 0.477246061 | 2.217527417 | 0.013277416 |
| unfolded | 0.475503291 | 2.209410775 | 0.01358732 |
| prions | 0.474382056 | 2.204188823 | 0.013762172 |
| alpha-crystallin | 0.468631198 | 2.177405228 | 0.014740284 |
| mptp-induced | 0.466291395 | 2.166508011 | 0.015117427 |
| chaperonins | 0.465040668 | 2.160682973 | 0.01534767 |
| brain-enriched | 0.453364351 | 2.106302602 | 0.017602176 |
| crowding | 0.450383751 | 2.092420989 | 0.018219258 |
| novobiocin | 0.448903502 | 2.085526995 | 0.018489312 |
| antihuman | 0.446655128 | 2.075055593 | 0.018993267 |
| inclusions | 0.443697305 | 2.061280057 | 0.01965152 |
| stripping | 0.43652044 | 2.027855085 | 0.021280124 |
| neurotoxins | 0.419789824 | 1.949935221 | 0.02558806 |
| shocks | 0.416353491 | 1.933931112 | 0.026556564 |
| spheroids | 0.416252345 | 1.933460042 | 0.026618099 |
| endoplasmic | 0.415331531 | 1.929171515 | 0.026865431 |
| stress | 0.414676881 | 1.926122599 | 0.027052187 |
| hyperthermia | 0.410666453 | 1.907444745 | 0.028260294 |
| aggregated | 0.405460827 | 1.883200465 | 0.029850184 |
| presynaptic | 0.401088313 | 1.862836259 | 0.031231133 |
| triplication | 0.392564161 | 1.823136538 | 0.034151695 |
| tegmentum | 0.386147678 | 1.793252912 | 0.036486462 |
| trimerization | 0.38254846 | 1.776490195 | 0.037866459 |
| synaptophysin | 0.379371484 | 1.761693993 | 0.039034649 |
| reticulum | 0.378153331 | 1.756020665 | 0.039544204 |
| dopaminergic | 0.376844099 | 1.749923149 | 0.040059157 |
| sn | 0.37468346 | 1.739860355 | 0.040929509 |
| zap | 0.373122278 | 1.732589429 | 0.041547834 |
| stresses | 0.369761712 | 1.716938191 | 0.042989586 |
| tunicamycin | 0.366182301 | 1.700267721 | 0.044565463 |
| cytoprotection | 0.366024752 | 1.699533965 | 0.044565463 |
| curing | 0.365590932 | 1.697513525 | 0.044753881 |
| cll | 0.364283347 | 1.69142368 | 0.045418401 |
| temperatures | 0.363685956 | 1.688641438 | 0.045609715 |
| hypothermic | 0.361219401 | 1.677153898 | 0.046771241 |
| rotenone | 0.355088089 | 1.648598404 | 0.049573817 |
| transmitter | 0.349963965 | 1.62473371 | 0.052081279 |
| lobule | 0.349889404 | 1.624386453 | 0.052187904 |
| extractions | 0.34580139 | 1.605347256 | 0.054246902 |
| cardiolipin | 0.344208414 | 1.597928253 | 0.055021489 |
| calreticulin | 0.340562964 | 1.580950218 | 0.056939019 |
| aggregate | 0.331370357 | 1.538137289 | 0.062024307 |
| m-chlorophenyl | 0.329643244 | 1.530093568 | 0.063008364 |
| cytosol | 0.329601186 | 1.529897691 | 0.063008364 |
| lymphocytic | 0.32918175 | 1.527944239 | 0.063256269 |
| fluidity | 0.328277702 | 1.5237338 | 0.063754356 |
| inert | 0.327903402 | 1.521990563 | 0.06400454 |
| chung | 0.327834912 | 1.521671584 | 0.06400454 |
| preprotein | 0.324102716 | 1.504289543 | 0.066290683 |
| reticulum-associated | 0.323530272 | 1.50162349 | 0.066548554 |
| ppiase | 0.316086057 | 1.466953381 | 0.071188028 |
| crystallin | 0.315356995 | 1.463557904 | 0.071596974 |
| prpc | 0.315000964 | 1.461899756 | 0.071870605 |
| high-frequency | 0.314153037 | 1.457950683 | 0.072420272 |
| bisphenol | 0.312648495 | 1.45094355 | 0.07338993 |
| plantaris | 0.310395169 | 1.440449083 | 0.0749337 |
| assist | 0.310358744 | 1.440279439 | 0.0749337 |
| parkinsonian | 0.309359023 | 1.435623417 | 0.075501171 |
| leprosy | 0.307655126 | 1.427687819 | 0.076645929 |
| injurious | 0.30556073 | 1.417933545 | 0.078095382 |
| tremor | 0.303441083 | 1.408061667 | 0.079565535 |
| synaptic | 0.302561865 | 1.403966865 | 0.080159425 |
| olive | 0.298462938 | 1.384876843 | 0.083026234 |
| neuropils | 0.297593235 | 1.380826353 | 0.08363948 |
| washing | 0.297496653 | 1.380376543 | 0.083793322 |
| superhelical | 0.296988578 | 1.378010271 | 0.084101644 |
| sonication | 0.295845104 | 1.372684745 | 0.084876173 |
| juvenile | 0.294056047 | 1.364352532 | 0.086283783 |
| terminals | 0.294011015 | 1.364142804 | 0.086283783 |
| immunophilins | 0.293029674 | 1.359572383 | 0.086914962 |
| calnexin | 0.292149088 | 1.355471209 | 0.087708779 |
| stressful | 0.29117468 | 1.350933078 | 0.088347716 |
| chop | 0.289769636 | 1.344389333 | 0.089474193 |
| cytosolic | 0.289567517 | 1.343447999 | 0.089635987 |
| oviposition | 0.28905709 | 1.341070779 | 0.089960226 |
