# Supplementary material for: The effects of aging on the BTBR mouse model of autism spectrum disorder
Source: Front Aging Neurosci. 2014 Sep 1;6:225. doi: 10.3389/fnagi.2014.00225 (PMC4150363; doi:10.3389/fnagi.2014.00225)
Supplement: Supplementary file 8 [file Table6.DOCX]

**Table S6. *Textrous!-*based collective analysis of downregulated BTBR-specific hippocampal proteins.** Cosine similarity scores, Z-scores and probability values (p-Value) were calculated using collective processing of the downregulated (BTBR:WT iTRAQ ratio <0.8) BTBR-specific hippocampal proteins.

| **Word** | **Cosine Similarity** | **Z-Score** | **p-Value** |
| --- | --- | --- | --- |
| myelin-associated | 0.746581109 | 2.726886136 | 0.003195652 |
| myelin | 0.723398874 | 2.638243496 | 0.004169829 |
| myelinated | 0.720504212 | 2.627175087 | 0.004307067 |
| axonal | 0.716289562 | 2.611059397 | 0.004513895 |
| hypomyelination | 0.690895817 | 2.51396054 | 0.005968522 |
| sheaths | 0.689821432 | 2.509852381 | 0.006036558 |
| quaking | 0.687522273 | 2.501061015 | 0.006192159 |
| proteolipid | 0.686935802 | 2.498818508 | 0.006227216 |
| oligodendrocytes | 0.68316172 | 2.484387431 | 0.00649579 |
| axons | 0.668568786 | 2.428587972 | 0.007570266 |
| oligodendrocyte | 0.660479068 | 2.397655065 | 0.008242433 |
| unmyelinated | 0.656477135 | 2.382352747 | 0.008609449 |
| callosum | 0.650338042 | 2.358878507 | 0.009162128 |
| ranvier | 0.64022612 | 2.320213235 | 0.010170439 |
| compact | 0.634069704 | 2.296672755 | 0.010809385 |
| pns | 0.630540954 | 2.283179761 | 0.011215184 |
| nerves | 0.627939932 | 2.273234152 | 0.01151309 |
| cns | 0.61180378 | 2.211533842 | 0.013483332 |
| fibrillary | 0.608852226 | 2.200247893 | 0.013903448 |
| neuron | 0.607901301 | 2.196611813 | 0.014010223 |
| schwann | 0.604259674 | 2.18268721 | 0.014517905 |
| motor | 0.603480337 | 2.179707233 | 0.014628731 |
| nervous | 0.589501365 | 2.126255402 | 0.01675163 |
| spinal | 0.580840815 | 2.093139785 | 0.018174577 |
| cord | 0.580798123 | 2.092976541 | 0.018174577 |
| neuropil | 0.569398833 | 2.049388721 | 0.020231057 |
| cerebellum | 0.558704493 | 2.008496437 | 0.022321646 |
| glial | 0.555527913 | 1.996350049 | 0.022966961 |
| nerve | 0.55325989 | 1.987677739 | 0.023405845 |
| neurite | 0.548218256 | 1.968399885 | 0.024534019 |
| brain | 0.538223978 | 1.93018445 | 0.026803419 |
| sheath | 0.530352976 | 1.900087855 | 0.02871656 |
| corticospinal | 0.528841512 | 1.894308423 | 0.029112505 |
| sciatic | 0.526748224 | 1.886304254 | 0.029647477 |
| neurons | 0.524134194 | 1.876308903 | 0.03032764 |
| neurofilament | 0.523253627 | 1.872941853 | 0.030534192 |
| cerebellar | 0.518681967 | 1.855461054 | 0.031798113 |
| parvalbumin | 0.514320406 | 1.838783614 | 0.032957593 |
| swellings | 0.513303274 | 1.834894377 | 0.033252845 |
| olfactory | 0.510202959 | 1.823039606 | 0.034151695 |
| projection | 0.508761873 | 1.817529277 | 0.034532066 |
| bulb | 0.505163908 | 1.803771627 | 0.035615653 |
| polysomes | 0.504773855 | 1.802280169 | 0.035772703 |
| purkinje | 0.504470398 | 1.801119833 | 0.03585144 |
| tremor | 0.503681103 | 1.798101779 | 0.036088504 |
| encephalitogenic | 0.502356744 | 1.793037788 | 0.036486462 |
| fasciculation | 0.50143462 | 1.789511833 | 0.036726956 |
| acidic | 0.49975596 | 1.783093087 | 0.037293152 |
| neurologic | 0.499303252 | 1.781362053 | 0.037456225 |
| growth-associated | 0.487853422 | 1.737580979 | 0.041105407 |
| neuropathies | 0.487207544 | 1.735111317 | 0.041370403 |
| neocortex | 0.486616695 | 1.732852068 | 0.041547834 |
| axon | 0.485381698 | 1.728129772 | 0.041994112 |
| neuronal | 0.485098179 | 1.727045672 | 0.042083832 |
| separations | 0.474752604 | 1.687486971 | 0.045801676 |
| ataxia | 0.469706884 | 1.668193494 | 0.047657863 |
| non-neuronal | 0.469673604 | 1.668066242 | 0.047657863 |
| neuron-specific | 0.469000609 | 1.665492887 | 0.047956378 |
| chromosome-linked | 0.46250039 | 1.640637797 | 0.050398708 |
| ectopia | 0.461957576 | 1.638562222 | 0.05060663 |
| horseradish | 0.461158787 | 1.63550787 | 0.050919794 |
| wallerian | 0.457664094 | 1.622145103 | 0.052401674 |
| outgrowth | 0.456637189 | 1.618218494 | 0.052831299 |
| dendrite | 0.452918428 | 1.603998952 | 0.054357026 |
| astrocytosis | 0.451292517 | 1.597781904 | 0.055021489 |
| matter | 0.450844982 | 1.596070651 | 0.055244397 |
| pyramidal | 0.44736619 | 1.582768685 | 0.056710732 |
| claw | 0.447298747 | 1.5825108 | 0.056710732 |
| olivary | 0.446972879 | 1.581264768 | 0.056939019 |
| neuropathy | 0.443605118 | 1.568387357 | 0.058440566 |
| feet | 0.443192788 | 1.566810714 | 0.058557346 |
| sg | 0.440855779 | 1.557874623 | 0.059616624 |
| cortex | 0.439485579 | 1.552635344 | 0.060211567 |
| neuroglial | 0.437554528 | 1.545251523 | 0.061173132 |
| gliosis | 0.436566967 | 1.541475358 | 0.061658393 |
| postnatal | 0.435350511 | 1.536823956 | 0.062146655 |
| dendrites | 0.43514517 | 1.536038785 | 0.06226919 |
| crush | 0.435057459 | 1.535703402 | 0.06226919 |
| labeled | 0.434747614 | 1.534518642 | 0.062391914 |
| neuromuscular | 0.433524443 | 1.529841562 | 0.063008364 |
| cortical | 0.433162628 | 1.528458079 | 0.063256269 |
| demyelinating | 0.432677661 | 1.526603696 | 0.063380506 |
| calcium-binding | 0.432395597 | 1.525525159 | 0.063504932 |
| central | 0.43129859 | 1.521330501 | 0.064129919 |
| brainstem | 0.423738296 | 1.492421965 | 0.067849573 |
| optic | 0.423490731 | 1.491475346 | 0.067980747 |
| galactosyl | 0.423338237 | 1.490892247 | 0.067980747 |
| ataxic | 0.422189282 | 1.486498953 | 0.06863956 |
| connections | 0.420242437 | 1.47905474 | 0.069570157 |
| fasciculus | 0.419382138 | 1.47576519 | 0.069971946 |
| hilus | 0.417907563 | 1.470126809 | 0.070780877 |
| neuron-glia | 0.41516538 | 1.45964144 | 0.072145037 |
| bulbs | 0.412853357 | 1.450800886 | 0.07338993 |
| entorhinal | 0.411514795 | 1.445682581 | 0.074088601 |
| zones | 0.411373308 | 1.445141575 | 0.074228943 |
| neuregulin | 0.410376865 | 1.441331443 | 0.074792341 |
| fibers | 0.406476547 | 1.426417675 | 0.076934171 |
| hippocampus | 0.406000267 | 1.424596511 | 0.077078601 |
| anterograde | 0.405134809 | 1.421287231 | 0.07765838 |
| auditory | 0.404234903 | 1.417846232 | 0.078095382 |
