# Supplementary material for: The effects of aging on the BTBR mouse model of autism spectrum disorder
Source: Front Aging Neurosci. 2014 Sep 1;6:225. doi: 10.3389/fnagi.2014.00225 (PMC4150363; doi:10.3389/fnagi.2014.00225)
Supplement: Supplementary file 9 [file Table7.DOCX]

**Table S7. Neurobiologically-relevant proteins differentially expressed between aged BTBR and WT mice.** The table indicates the reported ASD-relevant effects and clinical implications of experimentally identified cortical or hippocampal proteins altered in our BTBR model compared to WT mice.

| **Altered cortical proteins** | **Clinical implication** | **Source** |
| --- | --- | --- |
| EXOC6 | Patient with autism had mutation in exocyst complex component 6B gene; EXOC6B protein expression in patient’s blood lymphocytes and buccal smear was reduced in comparison with the control | Frühmesser et al., 2013 |
| DPP6 | Dipeptidyl peptidase 6 genetically linked with ASD phenotype | Noor et al., 2010 |
| Tomm20 | Mitochondrial protein Tomm20 has been shown to be reduced in ASD patients | Anitha et al., 2012 |
| Picalm | Associations with age at onset of Alzheimer's disease in Down syndrome with Picalm and the ApoE loci detected. | Jones et al, 2013 |
|  | Genome-wide association study of general population identified variants at Picalm was associated with Alzheimer's disease | Harold D. et al, 2013 |
| Eif5a | Eukaryotic translation initiation factor 5A-1 is a neuronal development-associated protein | Nishimura, et al., 2012 |
|  |  |  |
| **Altered hippocampal proteins** |  |  |
| Abat | Mutations in 4-aminobutyrate aminotransferase (ABAT) have been found in individuals with ASD. | Barnby et al, 2005, Hedges et al, 2010 |
| Setdb1 | Mutations in histone-lysine N-methyl transferase (SETDB1) found in people with ASD | Cukier et al, 2012 |
| App and Cyfip1 | Amyloid precursor protein (App) and cytoplasmic FMR1 interacting protein 1 (Cyfip1) have been associated with multiple forms of ASD-related pathophysiologies. | Sokol et al., 2011; Pathania et al., 2014 |
| Sfpq | RNA splicing factor Sfpq (splicing factor proline/glutamine-rich) has been associated with dysfunction nucleic acid processing in ASD. | Stamova et al, 2013 |
| PSD95 also known as Dlg4 | Dlg4 gene modulation is tightly associated with multiple ASD-related disorders. | Feyder et al., 2010 |
| Mobp | Genotypes of individuals at myelin-associated oligodendrocyte basic protein (Mobp) confer risk predominantly in ApoE ε4-positive subjects implying an interaction between ApoE and Mobp on Alzheimer disease risk | Liu et al., 2013 |
| Ina | Internexin neuronal intermediate filament protein, alpha (Ina) immunoreactivity is suspected to reflect neuronal vulnerability in the brains of individuals with Alzheimer's disease | Dickson et al., 2005 |
| Marcks | Myristoylated alanine-rich C-kinase substrate (Marcks) has been strongly linked to ASD phenotypes. | Stumpo et al., 1995, Hussain et al, 2006, Weimer et al, 2009 |
| Gad2 | Mice lacking methyl CpG binding protein 2(MeCP2) from GABA (γ-aminobutyric acid)-releasing neurons reportedly have a Rett syndrome phenotype with autistic features. MeCP2-deficient GABAergic neurons show reduced inhibitory quantal size consistent with a presynaptic reduction in glutamate decarboxylase 1 (Gad1) and glutamate decarboxylase 2 (Gad2) levels. | Chao et al, 2010 |
|  | Mice lacking Gad2 also show evidence of changes in extra synaptic receptor function | Blednov et al, 2010 |
|  | Gad2 has also recently been genetically linked to human ASD conditions. | Zhubi et al., 2014 |
|  |  |  |
| **Altered hippocampal and cortical proteins** |  |  |
| Prrt2 | Alterations in activity of Prrt2 found in the frontal cortex of individuals with autism and have also been genetically-associated with ASD. | Ji et al, 2012; Weber et al., 2013 |
| Vat1l | Genetic overlap exists between ASDs and other neurodevelopmental and neuropsychiatric diseases. | Griswold AJ, et al, 2012 |
|  | Vesicular monoamine transporters are involved in the presynaptic vesicular packaging of monoamine neurotransmitters, which have been postulated to play a role in the etiology of neuropsychiatric disease | Lohoff et al, 2008; Khalifa et al, 2012 |
| Dbi | GABAergic signaling pathways have been implicated in playing a role in the phenotype of individuals with ASD. These proteins include acyl-CoA-binding protein (Dbi), an allosteric binder of GABA receptors | Griswold AJ, et al, 2012 |
| Cep290 | Centrosomal protein 290kDa (Cep290) was recently linked to ASD phenotypes through whole-exome sequencing risk association analysis and via connections to Leber Congenital Amaurosis | Cukier et al., 2014; Coppieters et al., 2010 |
| Itsn1 | Intersectins encode a multi-domain scaffold protein proposed to facilitate interhemispheric connectivity essential for high order cognitive function; implicated in DS and AD | Hunter et al., 2011 |
|  | Itsn1 increased in mid-frontal cortex of people with DS but decreased in individuals with DS who also had AD | Hunter et al., 2011 |
| AP3B2 | In murine models neuron synaptic vesicle biogenesis mediated bycytosolic adaptor protein complexes, a ubiquitous AP-2 complex and the neuron-specific AP-3B complex | Suckow et al, 2010 |
